# A gene network of uterine luminal epithelium organizes mouse blastocyst implantation

**DOI:** 10.1002/rmb2.12435

**Published:** 2022-01-05

**Authors:** Shizu Aikawa, Yasushi Hirota, Yamato Fukui, Chihiro Ishizawa, Rei IIda, Tetsuaki Kaku, Tomoyuki Hirata, Shun Akaeda, Takehiro Hiraoka, Mitsunori Matsuo, Yutaka Osuga

**Affiliations:** ^1^ Department of Obstetrics and Gynecology Graduate School of Medicine The University of Tokyo Tokyo Japan

**Keywords:** blastocyst implantation, gene expression, laser microdissection, luminal epithelium, uterus

## Abstract

**Purpose:**

The receptive endometrium is critical for blastocyst implantation. In mice, after blastocysts enter the uterine cavities on day 4 of pregnancy (day 1 = vaginal plug), blastocyst attachment is completed within 24 h, accompanied by dynamic interactions between the uterine luminal epithelium and the blastocysts. Any failures in this process compromise subsequent pregnancy outcomes. Here, we performed comprehensive analyses of gene expression at the luminal epithelium in the peri‐implantation period.

**Methods:**

RNA‐seq combined with laser microdissection (LMD) was used to reveal unique gene expression kinetics in the epithelium.

**Results:**

We found that the prereceptive epithelium on day 3 specifically expresses cell cycle‐related genes. In addition, days 3 and 4 epithelia express glutathione pathway‐related genes, which are protective against oxidative stresses. In contrast, day 5 epithelium expresses genes involved in glycolysis and the regulation of cell proliferation. The genes highly expressed on days 3 and 4 compared to day 5 are related to progesterone receptor signaling, and the genes highly expressed on day 5 compared to days 3 and 4 are associated with the ones regulated by H3K27me3.

**Conclusions:**

These results suggest that specific gene expression patterns govern uterine functions during early pregnancy, contributing to implantation success.

## 1 INTRODUCTION

Blastocyst implantation is key for a successful pregnancy.^1^, ^2^ Upon blastocyst implantation, blastocysts directly communicate with the luminal epithelium. After mating in mice, uterine epithelial cells undergo massive apoptosis and cell proliferation, which continues until the serum progesterone (P_4_) level increases on day 3 of pregnancy (with day 1 being the presence of a vaginal plug).^3^, ^4^ The luminal epithelium then differentiates to be receptive and permits blastocyst adhesion.^4^ Uterine receptivity is strictly regulated by the balance of P_4_ and estrogen (E_2_), and this period of receptivity is defined as the “implantation window,” which normally occurs on day 4 of pregnancy in mice.^2^, ^5^ Blastocysts enter the uterine cavity on the early morning of day 4 and attach to the luminal epithelium by midnight on day 4. After blastocyst attachment, the implantation chamber is formed with the invagination of the luminal epithelia into the antimesometrial pole accompanying the initiation of decidualization on day 5.^6^

Accumulating studies have shown that anomalies in epithelial functions in the peri‐implantation period compromise subsequent pregnancy outcomes.^1^, ^2^ After coitus, epithelial cells undergo proliferation influenced by E_2_. Increasing levels of P_4_ then suppress the epithelial proliferation and initiate stromal proliferation in the receptive phase.^7^, ^8^ Previous reports have suggested that the switching of epithelial‐stromal proliferation by ovarian hormones is critical to establish the receptive endometrium to the blastocysts. P_4_ and its receptor (Pgr) signaling result in pregnancy success as revealed by the analyses using mice missing P_4_‐responsive epithelial genes such as *Hand2*, *Ihh* ^9^, ^10^ as well as epithelial *Pgr*.^11^, ^12^ *Lpar3*, a receptor for lysophosphatidic acid, is also a P_4_‐responsive gene expressed at the luminal epithelium in the receptive phase.^13^ *Lpar3* knockout (KO) females show deferred implantation and abnormal embryo spacing, causing shared placenta and reduced litter sizes.^14^ Lpar3 regulates decidualization processes by upregulating epithelial *Hb‐egf* and *Ptgs2* (the gene name of Cox‐2) which are also critical for the embryo attachment following decidualization.^15^, ^16^, ^17^ While it is believed that the early pregnancy is governed by various signaling pathways,^2^ the entire landscape of gene expression remains unclear.

In this study, we conducted RNA‐seq analyses combined with laser microdissection (LMD) to investigate gene expression during early pregnancy. We found that the levels of epithelial genes dynamically change over time. We also found that these genes are related to different transcription mechanisms at each time point.

## 2 MATERIALS AND METHODS

### 2.1 Mice

C57BL/6 wild‐type females (8–10 months old) (Japan SLC, Hamamatsu, Japan) were mated with wild‐type fertile males to induce pregnancy. Day 1 of pregnancy was defined as the day when we recognized vaginal plug. Females were kept separate from males after mating. To confirm pregnancy, oviducts (day 3 a.m.) or uterine horns (day 4 a.m. and p.m.) were flushed with saline to observe the presence of embryos. On the morning of day 5, females were injected with Chicago blue dye in saline to detect blastocyst implantation sites.^18^ These female mice were sacrificed on days 3, 4, and 5 for the collection of the uteri. The tissues were stored at −80°C until LMD. All mice used in this study were housed in the University of Tokyo Animal Care Facility according to the institutional guidelines for the use of laboratory animals. The experimental procedures were approved by the institutional animal experiment committee.

### 2.2 Laser microdissection (LMD)

Laser microdissection was performed as previously described.^4^, ^19^ To isolate the luminal epithelium, frozen sections of pregnant uteri (20 µm) were mounted on film slides and dissected using the LMD7000 System (Leica Microsystems, Wetzlar, Germany).^4^, ^19^ For each day of pregnancy, tissues from 3 females were dissected. The extracted RNA was amplified using the Ovation PicoSL WTA System V2 (Tecan, Zürich, Switzerland).

### 2.3 RNA‐seq analysis

RNA purification, cDNA library construction, and sequencing were performed at Macrogen Japan (Tokyo, Japan). RNA collected by the LMD system was then treated with DNase I and magnetic beads with Oligo(dT) to isolate mRNA. cDNA synthesis was performed using the TruSeq RNA Sample Prep Kit v2 (Illumina, San Diego, CA, USA). To check the quality and quantity of the sample library, a 2100 Bioanalyzer (Agilent, Santa Clara, CA, USA) was used. Sequencing was performed on a HiSeq 2000 (Illumina). The reads per kilobase of exon per million mapped sequence reads were counted using the featureCounts function in Subread (version 2.0.0).^20^ Genes with log2 fold change >|1| were defined as differentially expressed genes (DEGs). DEG heatmaps were created using Morpheus (https://software.broadinstitute.org/morpheus/). K‐means clustering and visualizations of the data were performed using trinityrnaseq (version 2.0.6).^21^ Genes in each cluster were then subjected to comparative analysis and gene ontology (GO) analysis in Enrichr (https://amp.pharm.mssm.edu/Enrichr/).

### 2.4 Immunostaining

Immunostaining was performed as previously reported.^22^ Briefly, 12‐µm frozen sections were fixed in 4% PFA‐PBS and then incubated with specific primary antibodies: anti‐Cox‐2 rabbit antibody (1:300; 1AA570‐598, Cayman Chemical), anti‐Ki67 rabbit antibody (1:300; SP6, Thermo Fisher Scientific), anti‐Pgr rabbit antibody (1:300; D8Q2J, Cell Signaling Technology), and anti‐cytokeratin 8 (CK8) rat antibody (1:500; TROMA‐I, DSHB). Sections were then incubated with Alexa‐conjugated antibodies for signal detection: anti‐rabbit IgG‐Alexa555 (1:500; A27039, Invitrogen) and anti‐rat IgG‐Alexa488 (1:500; A11006, Invitrogen). DAPI (2 µg/ml; D5888, TCI chemicals) was used to stain nuclei.

## 3 RESULTS

### 3.1 Gene expression kinetics in luminal epithelial cells dynamically change during the peri‐implantation period

**FIGURE 1:**
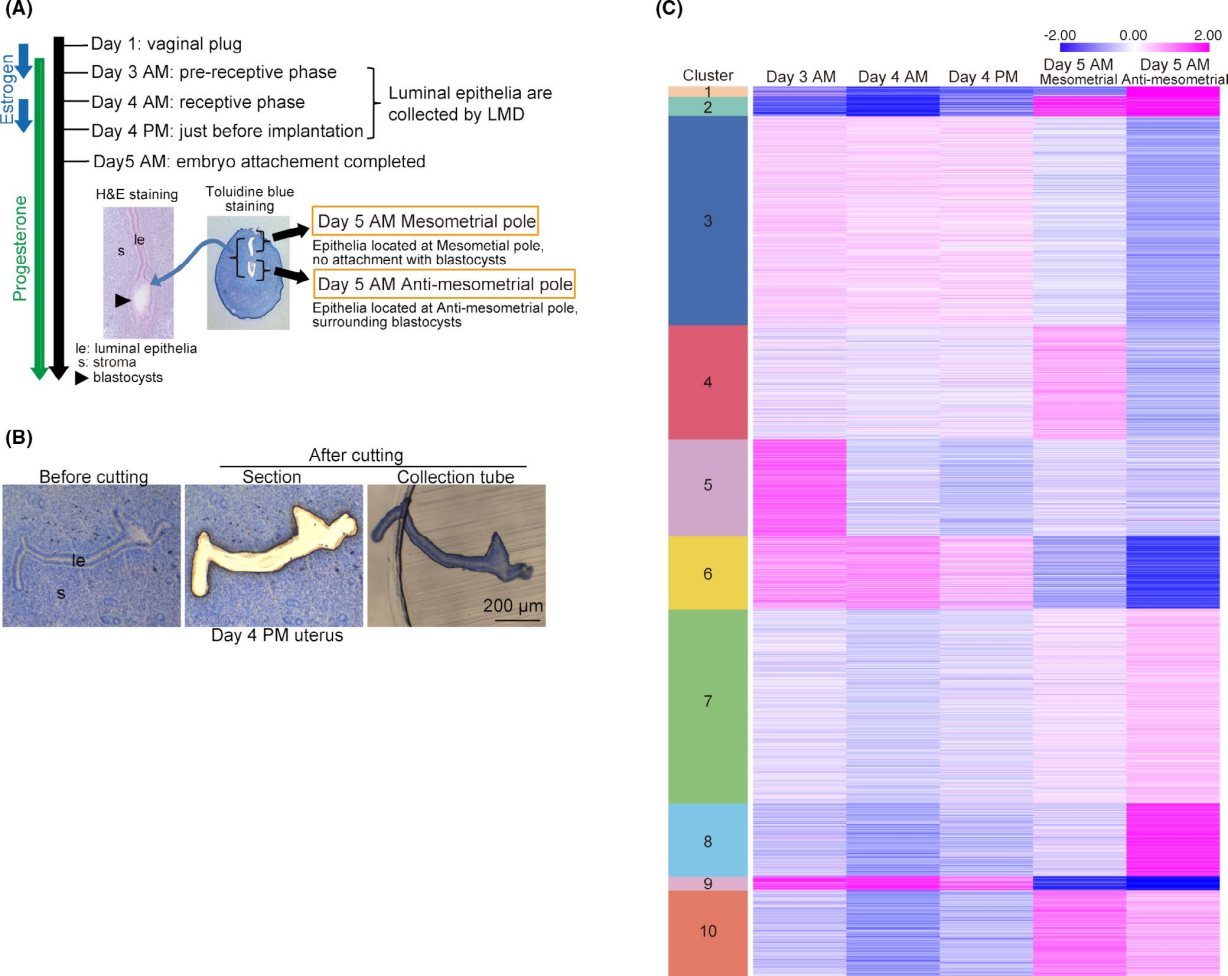
RNA‐seq analyses on the uterine luminal epithelium in the peri‐implantation period. (A) The analysis scheme of this study. Females on day 3 a.m., day 4 a.m. and p.m., and day 5 a.m. were used. (B) Representative pictures of the uterine tissue before and after the epithelial dissection using LMD. (C) Heatmaps depict log2 fold enrichment of DEGs during the peri‐implantation period. Numbers shown on the left indicate different gene clusters

To examine gene expression in luminal epithelial cells during early pregnancy, we prepared epithelial tissues using LMD and then performed RNA‐seq analyses (Figure 1A,B). Uterine tissues were collected on the morning of day 3 in (day 3 a.m.; prereceptive phase), on the morning of day 4 (day 4 a.m.; receptive phase), in the evening of day 4 (day 4 p.m.; just before blastocyst attachment), and on the morning of day 5 (day 5 a.m.; the initial stage of decidualization). In the tissues from day 5 a.m., we separately dissected the luminal epithelia at the mesometrial pole without contact to blastocysts and at the antimesometrial pole with contact to blastocysts to observe the influence of blastocysts on the luminal epithelium.

**FIGURE 2:**
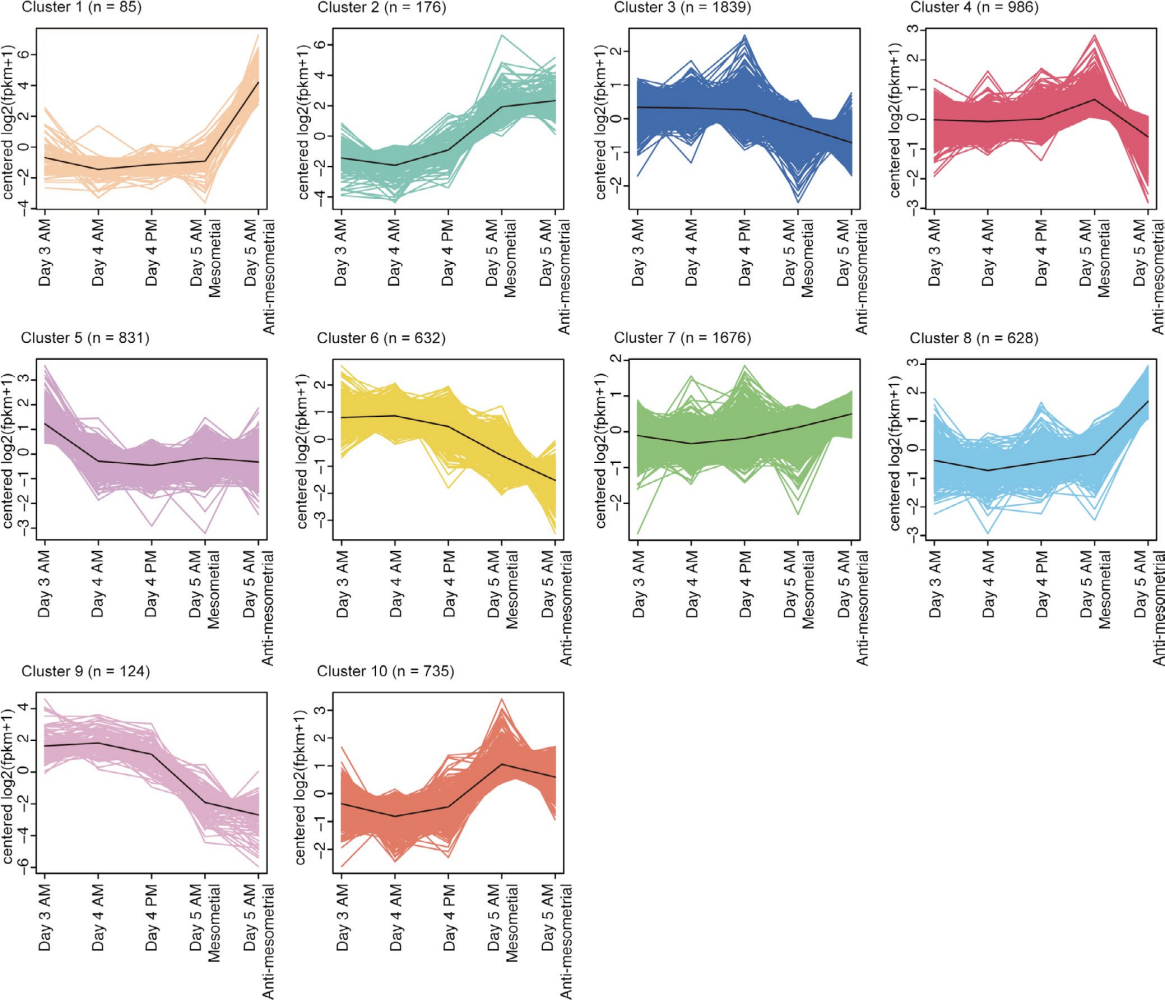
K‐means clustering shows differential gene kinetics during the peri‐implantation period. Ten different clusters were revealed by k‐means clustering. The numbers of genes in each cluster are shown on the top of each graph

To observe distinctions in gene expression kinetics at each time point, we then performed k‐means clustering (Figures 1C and 2, Tables 1, 2, 3, 4, 5, 6, 7, 8, 9, 10). K‐means clustering is an algorithm that repetitively calculates means of clusters to reallocate each gene to its closest cluster depending on Euclidean distance.^23^ We detected 6712 DEGs showing log2 fold changes >|1| in at least one time point compared to others (Figure 1C). Among 10 clusters defined by the analysis, genes in cluster 5 are highly expressed only at day 3 a.m. Clusters 6 and 9 are highly expressed from day 3 a.m. to day 4 p.m. but decrease by day 5. In contrast, genes in cluster 2 are increased in the whole luminal epithelium on day 5 a.m. We also found that cluster 4 and clusters 1/8 contain genes expressed only in blastocyst‐unattached or blastocyst‐attached epithelia on day 5 a.m., respectively. These results led us to ask which molecular and biological pathways are governed by these unique gene expression patterns on each day of pregnancy.

**TABLE 1:** Gene list of cluster 1

	D3AM	D4AM	D4AM	D5AM_mesometrial	D5AM_antimesometrial
Serpinb5	6.12710221	2.19590927	11.1117784	7.65586434	93.8596552
Dppa1	0	0	0.35974032	0	20.4137565
Ecm1	1.82002082	1.26730739	0.63596611	3.90934929	71.2726507
Cldn1	96.4996183	16.0675995	11.4211645	13.3372386	258.771866
Il11	0.10063532	0.21071857	0.03106223	0.46195828	278.43752
S100a3	0.45794726	0	0.16962074	0.6726943	216.295883
Trap1a	0	0	0	0	18.2055298
Spp1	95.457642	12.8165946	16.0249651	26.3600415	2329.77399
Pthlh	12.5506625	1.82873613	14.976434	0.1583857	799.172442
Ereg	0.14190165	0.34664631	0.68619298	0.72376442	179.388564
Hsd17b2	0	0	0.84916381	3.97572843	183.145989
Ptpla	0.90571791	1.47314855	4.19340153	10.5128505	110.321257
Krt17	11.6094934	0.10166369	2.03814301	1.22830151	403.257262
Aqp3	0.44386856	0.05808805	0.1027538	0.20375426	32.0421385
Ctsll3	0	0	0	0	33.347464
BC053393	0	0	0	0	107.865695
Amica1	0.74285082	0.20831819	0.5404677	0.90121332	58.9331234
Crisp1	0.36842762	0.0723229	0.5543819	0.12684278	19.781064
Chrna1	0.0301906	0	0	0.31875121	21.0602313
Uchl1	2.84270661	0.89284414	0.21058407	1.5137088	31.4820309
Hmox1	8.39972905	8.64240271	18.3530497	28.453347	363.646776
S100a8	0.6654254	1.56748811	3.23490975	0.91637438	127.31085
Plac1	0.24378202	0.57425733	0.4514765	0.05595308	32.184721
Adora2b	0.17682128	0.55536455	0.52394778	3.86361461	37.1809969
Sprr2d	6.6073518	0.75190325	1.77342091	0.35165811	534.161562
Arg1	4.15023617	1.17871144	1.144739	1.94566693	65.6667599
Nat8l	0	0.00784264	0	0.05501896	34.6728779
Ankrd37	9.73847134	3.34189389	1.8703313	4.50347999	119.082214
Clic4	25.0962309	7.63533331	12.8228936	37.5364744	319.266713
Dppa5a	0	0	0.85049271	0	156.690533
Slc16a3	1.72075859	0.95375295	1.00758798	1.51010343	126.233042
Crct1	0	0	0	0	131.991938
Tnfrsf23	0.23836134	0.04679069	0.03678647	0.56532549	23.733122
Sprr2f	2033.23409	189.431839	727.296951	93.6878695	8732.74127
Slc26a4	0.40188845	0	0	0.73793452	18.9164127
Obp2a	0.091461	0.64634152	2.20197691	0.92365739	168.132626
Trim15	0.06989463	0.13720421	1.19734423	1.47588985	119.413566
Degs2	3.1565815	0.97423436	0.38859949	1.22285811	65.0220552
Sprr2b	7.92989072	0	4.49215889	0.2569519	1527.57686
Fads3	0.51866134	0.61088402	0.55416013	8.27811145	115.08146
Krt16	0.08333762	0	0	0.03825546	19.9346264
Nppc	0.0625832	0	0	0	157.925788
Slpi	1.6377365	0.35071652	0	2.73378049	43.5478648
Sprr2e	4.23321632	1.18712392	0.9333073	1.38801683	355.221346
Prss56	0	0	0	0.02994987	195.775863
Cyp11a1	0	0	0	0	18.7054526
Fst	0.64179241	0.43820806	1.65367516	4.7393717	116.858096
Tmprss11d	0.06374554	0.05005338	1.71179324	1.72645053	458.312543
Guca2b	2.93452603	2.38954854	9.66159713	53.1843326	1176.48911
Lce3a	0	0	0	0	79.8615487
Areg	24.2572256	50.4786079	50.5642385	33.0557155	2104.46237
Pxdc1	1.81143582	0.38294047	2.06444383	2.0148488	32.605068
Slc7a11	20.2836836	4.42289752	1.42013017	0.60343457	64.0489176
Aldh1a3	22.9967105	0	0.28416462	0.1408701	56.2535947
2010109I03Rik	0.06911679	0	0	0	179.342431
Gjb6	1.09774	0.46592003	0	1.33467691	30.3844429
Stom	1.45892629	1.70924485	0.94351534	2.33866378	33.6815516
Gm1045	0	0	0	0	73.1191026
Cdkn1a	10.5896574	1.31423485	1.99693672	4.46216126	90.2253293
Krt20	0.47303816	0.02653089	0.03128756	0.06204124	47.9252149
Adm2	0.8112459	0.58799554	0	0.34375001	22.9032598
Upp1	1.27699131	0.70189089	0.74889991	0.54711299	43.4227926
Nrgn	0.30260645	0.27720981	1.35434221	3.56533182	67.777048
2210409E12Rik	2.24867895	15.4836226	2.24241313	0	55.1683295
Fabp3	1.7321855	0.32002882	0.18870307	0.28063965	169.428411
Tnfsf9	4.66555177	1.3321525	1.03096311	3.94264489	45.502208
Des	3.30761943	6.98136397	6.26610098	10.2801087	148.09111
Ptgs2	24.4327512	4.13837029	14.1602419	0.96773851	104.086226
Ak4	0.32845872	0.28766636	0.43282533	0.26675809	19.5282056
Egln3	2.26632553	0.26788661	0.74465785	1.52135497	62.1387021
Cryab	46.5011626	4.06180041	2.39501617	5.75962564	259.479125
Il1f6	42.9082578	1.15978735	0.13677233	3.39013549	50.4228051
Sox21	0	0	0.27035678	0.74107987	15.8547717
Krt6a	0	0	0.24068416	0	18.8617412
BC051665	0	0	0.35836785	0.04441379	116.610069
Pla2g7	12.5655945	1.47062853	2.20728394	28.6687206	457.436777
Ncmap	1.45521204	0.19044021	0	4.62035593	136.049117
Adm	2.17214896	1.55727276	8.78883518	7.3699238	100.920048
Btc	2.51686284	0.92581356	1.46973183	5.97448906	72.6080393
Tnfrsf12a	30.7474806	1.32654434	11.1383697	24.8164944	259.500147
Inhba	0.68239832	0.06697791	0.07898624	0.11746853	23.7192032
Tmem190	0	0.350117	0.20644438	0.61404916	23.7615016
Mrgprg	0	0	0	0	20.5471309
Phlda1	7.91355372	2.70049923	2.22617445	3.43339313	61.5958124
Sprr1a	15.6838241	0.12963193	5.27413134	0.90941457	139.176771

**TABLE 2:** Gene list of cluster 2

	D3AM	D4AM	D4AM	D5AM_mesometrial	D5AM_antimesometrial
Slc46a2	0.37987392	1.590822882	11.72523923	87.01464057	189.5473662
Il20ra	2.217447297	5.664878	1.735200633	55.36808901	29.98295515
Ece1	8.097298659	4.901438105	15.99901102	63.61165483	115.2137611
Tll1	0.17202759	0.072733823	1.053796359	34.60007505	28.11170198
Slc45a4	5.026791317	4.644238959	7.108579787	47.06563308	60.1277975
Glo1	31.71460713	22.10327996	32.97972975	152.9010902	194.7728222
Aig1	8.600023572	7.384346255	8.536396891	51.51983117	119.4880691
Epha2	1.144355691	0.39067608	1.428230755	6.821380256	12.77197295
Slc16a1	1.66491661	1.191612062	2.373923831	33.65477732	86.15511136
Cwh43	21.24848269	31.68910533	58.69340127	386.472422	271.1877867
Slc39a8	9.604958858	12.2106492	34.30557652	172.4708705	89.44522314
Cyp26c1	0.712009455	1.183921654	9.152765421	27.41628918	18.92947647
Sepn1	5.773458725	4.01313356	6.064790649	25.84220083	39.65194257
Me1	7.67518068	1.732110147	2.397167305	42.17832759	40.86218989
Wnt7b	9.866683717	1.333755453	5.593988143	288.4462882	259.594592
Trpm6	1.112075209	0.778807273	6.048703071	29.53478332	37.37261829
Gadd45a	15.71686962	8.534101921	118.3032573	213.9464654	106.1799408
Anxa8	0.069819796	0.986812642	2.941666684	16.89046081	8.476447335
Lrrc8b	1.331319251	0.144187572	0.977722342	5.521255089	34.13931868
Tacstd2	212.1597995	33.32363607	51.86125158	381.8565978	558.7161423
Bnip3	32.01166081	34.61268444	38.0329101	151.8906189	713.4211583
Atp6v0d2	1.198456939	2.434418207	7.527013342	883.3365751	546.1037946
Atp13a4	0	0.011429601	0.175224278	21.22170333	69.14778809
Gabrp	9.642582142	0.031222324	0.331380999	31.39513524	13.82910241
Dhx40	16.9158142	15.27613411	14.28549119	81.58947324	135.7093663
1810010H24Rik	2.076805399	1.16480008	2.582433263	80.89776367	8.321248041
Serpinb6c	0.196420752	0.308461515	3.228413949	6.942731994	34.94322641
Coch	6.684015886	32.53964318	249.3721957	86.60255899	943.6528509
Aspg	0.238695789	0.074970149	0.088411394	7.275538508	7.019941301
Gnl2	5.993925506	7.103278025	9.533118441	70.97755008	102.7136616
Depdc7	2.235612831	0.208269774	1.54383452	37.29251474	21.61957911
Gda	1.384409137	1.795989697	2.129136343	26.9010662	69.79556244
Klf7	3.758458154	2.644549547	4.565032325	25.63287535	45.76437283
Npnt	1.45538323	0.096378672	1.607451794	17.19304617	7.686157144
Dram1	3.554466868	10.25186736	37.13020271	230.9903298	155.8973221
Gpx3	9.801097702	3.847940484	1.791248643	284.470314	28.79842974
Entpd3	4.363818158	2.017331161	18.55981053	39.36976524	32.54736125
Ggt6	9.198355769	27.23230986	70.59276166	102.7841448	331.3270693
Fga	0	0	0.161745488	8.731011447	56.3167208
Snora20	0	0	0.989917738	22.57385629	2.57003792
Sphk1	1.675735924	2.007149407	8.10042442	31.73411926	23.05609076
Chst12	5.075265871	1.69152972	0.946685049	38.9522392	16.70716513
Cebpa	3.187687742	4.035831451	10.31967673	52.95039649	27.14852106
F3	48.14402452	27.56751474	29.99936208	174.6308668	242.233837
Lcn2	36.77235951	3.481673469	21.59134762	79.66261994	436.3771732
Hbegf	11.20268182	2.889018082	5.54270572	22.08250041	119.7850488
Gpc3	0.546306041	0.880502579	0.905242235	5.965861185	12.39616467
Rnf125	38.12977349	34.46907133	26.11854171	152.8160753	346.607053
Sftpd	71.06402065	6.487462094	105.7420274	630.1227964	415.3008133
Osmr	2.211937349	0.698966776	14.56233486	14.02946747	33.93761207
Glrx	8.206746158	5.556491379	6.325178978	52.60601234	281.1741561
Basp1	9.562037495	36.90596529	31.08768617	219.5656066	241.1730049
Igfbp3	5.207321646	8.009894187	46.43857341	725.8170415	1163.038281
Slc37a4	8.257019682	13.35986027	8.225100671	156.9249005	52.48300937
Nt5e	10.58323788	6.493545708	8.855339545	67.07758068	54.3156217
Tuba8	0.364311115	0	2.108414177	32.52702429	92.03439795
Pgap1	0.88148895	0.44529958	0.376728312	13.26541207	34.84981784
Phlda2	0.176965236	0.903202103	0.819334899	5.036536163	11.98304881
Ggh	30.98292942	13.39969849	27.18690368	2872.84704	524.0719506
Cybrd1	0.363121927	0.766889343	1.020329958	42.83311212	33.90512327
Cfh	37.36413185	5.783247075	10.94261645	143.2074471	101.1028777
Tmem154	1.073202551	0.199180117	0	7.91814734	13.63509446
Foxo1	10.97041031	11.27017927	26.87523246	101.5155312	73.16229525
Adamts9	0.583404777	0.330844906	4.021663814	21.79157302	8.051593283
Rnf186	5.196150561	1.713620465	6.591826724	125.6550073	232.0146955
Arc	0.085271905	0.033478007	0.532982842	11.4885807	15.3577759
Efna1	19.61910757	9.074629692	25.81623471	186.42768	501.8878151
Foxq1	0.232483742	0	0.064582868	2.945465741	23.36214862
Penk	0.738942658	1.378027789	2.138278401	34.64137443	59.5483125
Ttpa	1.901028617	0	0.237346801	209.2403174	24.27155148
Grhl1	1.200781819	0.493188893	0.461868135	11.19378562	31.7541654
Spaca7	0	0	0.238048549	31.70502824	7.553637512
Ces2b	0.550600017	0	0.095596278	22.21027141	6.783815131
H6pd	4.678618789	4.300366193	8.142224841	124.7803214	149.9134555
Eif4ebp1	39.55245186	14.35399406	16.25039272	158.2491057	288.5612029
Stx11	1.633013928	0.213708729	4.56163679	11.39425105	29.90189778
Kng1	0	0	0	16.61902869	10.07307161
Gmpr	6.965984854	2.932567842	8.393279357	193.9026172	162.9261812
Prss35	0	0	0.239338018	46.05916282	2.071242081
Guca2a	0.815146119	0.091436806	0.754812275	8.766648228	37.79332548
Prss22	2.413423555	2.93923752	15.96279737	18.94670935	115.5268279
Akap12	0.960377051	0.795077208	2.126572122	11.14592108	13.67711071
Tgfbi	14.14786842	11.414919	36.57372698	164.2954097	375.0319997
Fgf9	2.898997554	0.606191342	4.230887845	57.06362226	37.24557652
Cep120	4.75412405	3.710479096	10.68737275	38.41447837	39.89879466
C330005M16Rik	0	0	0	7.689983784	24.67485878
Il1a	0.660706074	0.02593952	1.254196689	23.08050269	172.1530483
Runx1	0.920344105	0.409970427	0.753890399	5.25658243	17.55861253
Neat1	7.77942409	8.235932166	22.7702891	58.92755493	116.1605367
Slc9a2	0.634754493	1.143418179	0.380323391	6.701714565	34.73855435
D16Ertd472e	0.571660915	0.806306383	1.186133576	9.165132239	10.47693759
Cda	5.481328663	1.645636464	17.31683045	204.1789348	319.1644001
Camk2n2	1.169944508	0.079882389	3.108743225	4.623330332	28.00379072
Cldn10	2.299997349	1.345626029	47.73169742	382.9430937	577.5422627
Pcolce2	2.236628151	0.63113937	0.809016372	28.33066829	13.30240241
Ascl2	0.889985837	0.254117179	1.423467319	11.9962426	7.715428146
Paox	27.33109383	7.782393462	10.95064441	134.8362863	76.5058515
Nceh1	9.928680086	7.283314111	8.589124664	60.59193639	53.47051431
8430408G22Rik	0.445637969	0.194398677	3.255378682	2162.586015	293.2685816
Trib3	3.720023245	2.396705367	1.089343898	19.26575487	64.64023376
Rassf4	1.665241757	0.734492378	3.083974492	27.91532898	17.80080751
Gjb2	90.49342608	8.243005267	16.30193567	804.9797471	5203.266985
Kng2	0.025857133	0	0	8.498566937	5.553129779
Car12	7.475828779	2.411412007	25.49624242	37.00778095	236.5788144
Agtr2	0.794710701	0.42789369	3.637396202	248.4221172	203.5165183
Il34	0.141918802	0.390024245	0.952755429	19.18569529	7.47753325
Mgat4a	0.600901591	1.016252568	2.396908842	12.41699842	18.80200976
Gm14137	0.786707699	0	0.137820305	10.81443274	6.47467438
Cyr61	8.90711933	0.833211134	20.21765424	31.3814545	301.845969
Fgg	0	0	0.475784494	36.36215254	156.8409374
Them5	43.89148089	69.37795087	40.49800154	183.5336973	872.8788314
Crim1	0.337208069	0.281860247	1.601536638	4.943377385	20.42354904
Atp6v0a4	0.397027029	0.264985813	3.143327832	11.0809236	9.95834277
Ddah1	0.400285257	0.560285314	3.255341973	6.646873178	36.21899271
Relt	0.763928205	0.363540018	0.471590204	8.267429674	34.37452327
Arid5a	2.139776442	1.644225989	19.87756208	39.33190106	26.65596971
Fhl2	1.96094012	0.753490765	1.931701281	12.6787597	24.90837561
Ehhadh	2.989771813	0.986666933	0.501536396	10.87999224	29.91347679
Pcsk6	1.684154848	0.035740771	0.379337951	29.07125582	24.8399128
Dio3os	0.713810519	0	0	27.85185825	11.93054872
Wdr72	0.012497449	0.058878434	2.81210246	8.616752468	11.7474079
Slc20a1	2.78896702	0.55362987	2.234331387	17.47760649	52.13187922
Tmem213	5.167718793	0.869512271	19.48270175	309.4025706	58.96216555
Adi1	74.90584517	79.26779564	153.9726915	1806.638016	942.3809929
Hal	0	0	0.19705034	27.72071261	12.2401395
Ttc39b	0.602709591	0.964030226	6.283786355	27.1955323	15.30634899
Ndrg1	25.99441742	13.53644402	63.47736174	205.9189413	336.7801997
Nfil3	11.88345547	27.06749796	116.3744709	294.8707515	355.4660044
Klf4	7.069598406	3.330654758	6.824058417	24.7228415	68.44625627
Tekt1	0.324667018	0	0.773065204	3.960092979	19.77308453
Rnf39	2.480389298	0.920691494	5.26176192	38.58827584	38.66905832
Ly6g6c	0.135857687	0	1.509624551	8.606282712	46.59621528
Ldlrad3	7.770193475	0.9633478	4.433807147	17.44311966	29.75410419
Thbd	2.262589551	0.688604243	6.48026031	16.87561668	61.42146389
A630023A22Rik	0	0.076768533	8.46476135	8.572061785	7.521310373
Fam126a	3.210350574	4.361504896	8.330069242	24.67097712	40.44005715
Slc5a3	2.80255497	2.988851603	6.103237899	37.99671812	29.85181587
Olfm1	3.690509851	1.777522979	2.289978899	397.0128732	357.4634354
Prss12	0	0.15712961	0.277951586	15.45545475	28.10315598
Esm1	0.713204403	0.024348365	0.516847207	35.13044427	21.19619323
Vtcn1	21.71235887	15.54495453	30.56097298	193.8347334	182.2433296
Efhd1	12.75331103	11.6593326	66.17257311	324.49096	247.0141044
Cebpd	18.40931767	30.17900113	160.90193	194.5503355	374.2418713
Pnp2	0.213284348	0	0.049374474	40.97384837	100.5838171
P2ry6	1.020527223	2.030021114	0.913492164	7.932669466	50.15841699
Steap4	0.373824972	0.554444838	1.365392906	8.294063649	32.15330243
Atg14	3.590327734	8.487116139	9.361334509	26.54325788	70.68609654
Chi3l1	0.115828933	0.060633051	0.50052679	53.09943729	91.08697252
Ctsc	21.15686691	11.24761491	13.41074237	181.5469121	81.62053679
Pnp	19.37946843	12.37894553	22.31842077	130.2437723	175.8380155
Ifitm6	21.09435429	6.436069471	41.41003389	662.1090022	790.2367797
Hnf1b	9.516481508	9.695437207	13.17410407	85.01030044	66.28935092
Nmb	4.279226417	9.788042717	19.81247627	547.2833554	216.1117145
Tead4	0.084800637	0.016646493	0.058893025	3.483970386	8.137617899
Maff	3.369270626	1.706820384	5.770120506	19.69050963	70.48947707
Gm2115	0.105010772	0	0.194476593	8.22687437	20.78516443
Hand2	2.042424281	1.809916918	2.755824684	10.42029056	53.35636368
Hey1	1.774467742	0.301886599	4.135207387	23.45918411	64.91298043
Tmem62	1.911298647	0.964776351	1.474859994	53.43577457	16.19141824
Ttc9	7.568643692	1.780375817	7.245014985	25.74225174	146.1007404
Nbl1	41.04413376	15.25676564	21.93325114	143.3545152	275.8789361
Lrat	0.073148278	0.019145489	9.527935098	21.93770683	80.71619934
Sdcbp2	12.40106605	3.406235908	7.771892121	52.29407786	277.5349818
Grina	18.10365864	29.91220366	34.74009008	133.9909302	231.9020256
Prap1	732.9500346	103.7263835	41.55108089	3077.230346	1885.888592
Cyp26a1	22.15220223	12.42020897	357.4275579	3333.480141	3024.358862
Xbp1	125.7508904	78.23116661	216.4668582	1419.369707	1556.676276
Fam69a	2.753063251	1.240640367	3.679848391	31.56131514	15.21297654
Ppap2a	7.59953934	2.033516473	4.953455558	20.15148624	71.07147614
Tbc1d1	9.051843503	3.893134124	3.557289074	93.95986441	27.01378591
Clcf1	12.11757556	5.758953864	14.09967184	79.338454	58.39033992
Arnt2	4.452684644	0.889028314	3.770281015	42.60850194	57.34359566
Nupr1	103.1303077	11.69690008	299.3204461	1428.458826	2222.861104
Afap1l1	1.341410011	2.27208238	8.961039062	17.50527294	28.65482772
Sdc1	24.19831673	4.741788306	7.390046885	76.91387646	124.7099127
Fosl2	12.22371598	8.130904543	19.93950626	60.72755546	78.3014822

**TABLE 3:** Gene list of cluster 3

	D3AM	D4AM	D4AM	D5AM_mesometrial	D5AM_antimesometrial
Kif27	0.42383214	1.72135628	0.78492357	0.02683541	0.02342333
Aph1a	8.19456379	9.82682591	6.96822032	5.13967739	6.06283803
0610009D07Rik	181.844783	130.993876	157.23903	93.077142	75.8107452
Pisd‐ps1	3.10531855	5.46827959	3.44634176	4.02486002	2.43355715
Nadsyn1	2.80080613	1.79050713	1.9967668	1.8204422	0.81435004
Ppp2r3c	9.14869632	8.16708209	10.4885814	4.77458473	2.53104917
Dpysl3	2.82920131	6.59853241	1.86757676	2.08309846	3.63647118
Ramp1	0.56105358	2.05021774	0.93914433	0.9509431	0.88190911
Gbp9	1.29238691	1.25439318	1.66212447	0.64269802	0.30206611
Ly86	9.02629203	10.3744549	9.44840241	9.24769116	3.09246622
Chic1	4.29986549	3.92976886	6.69856374	2.47536233	3.48100484
Cstad	0.79818469	3.13369716	0.59128501	0.65952036	0
Unc119b	20.9973406	32.5809194	23.8523165	20.97004	14.5741303
Mpst	4.84160376	6.35852272	2.53918453	1.41610548	1.44205807
Hint2	30.012825	27.6383605	25.2370399	26.4399593	11.2296017
Dlx6	16.6225875	10.2791704	10.9380827	6.93170337	3.07396692
Klrb1f	0	0.98931627	2.1212523	0.38557829	0.03059569
Stra13	89.2046697	58.1285055	71.3036502	46.9733123	26.2108846
Mt1	1191.85793	2489.84785	3063.03378	532.090022	1824.28594
Mpp7	20.2321874	23.0830196	17.4963071	18.0341558	5.79108764
Mrpl50	96.4686477	66.982352	91.9700891	55.0585731	30.2660766
Fundc2	45.5941896	27.4001984	25.8501794	28.3284545	9.91945843
Fbxl19	5.3737492	6.32174236	3.9312217	1.6854839	3.2488495
Pigx	88.5956792	80.1688362	68.6802927	42.2717022	30.2987568
Sipa1l3	5.68160967	7.0437045	4.25114032	4.41668768	4.28872787
Zfp560	8.25481869	6.10300104	6.98025891	5.18736582	1.78903316
Mrpl30	142.219994	101.816406	112.468121	90.2812215	50.518044
Ncaph	12.5146412	5.89214164	6.27609316	3.82242181	2.85146375
Lyrm5	39.0948699	42.6547349	52.4601508	29.6213454	15.8092433
Baiap2	8.25533023	8.89343253	6.92503672	4.89240689	2.47535231
Dclre1b	5.58131764	4.60937035	3.36567414	2.10828795	0.91401768
2310033P09Rik	16.5525239	17.7008689	15.4246892	12.8809304	6.99011657
Osbpl10	1.77660052	2.6805833	3.16117962	1.63106807	1.11661192
Gpr19	3.02768559	3.82002657	1.79717208	2.09069118	1.72117751
Ccdc32	11.4033275	12.4041118	8.52493864	7.23560063	7.23779485
Tjp2	22.6433253	20.159548	19.8220814	12.527985	20.8681223
Ocel1	2.28951452	4.55634255	3.5456073	3.00798596	1.92960272
Coa3	400.89749	317.925011	281.908718	183.611816	126.786151
Fech	20.7931102	18.4273059	13.9670193	19.6676706	7.11536984
Shroom2	10.5205408	17.983792	11.5467947	11.4243133	6.17828794
Clec2d	0.59088022	1.56060183	0.09948103	0.34521297	0.129137
Hlcs	1.78107656	3.86709771	2.97364488	1.52368808	1.1028882
Nnat	8.51524541	4.10483249	24.7528521	5.29427093	2.15939825
Zfp647	1.02159305	1.06954802	1.52407701	0.91185498	0.38658679
Elp3	13.8269361	15.5701071	14.9870682	13.0682256	6.94017253
Syk	2.83260184	1.45688386	0.90485842	0.72679566	0.29736782
Zc3h6	2.85201653	2.08318192	0.67558462	0.94992755	0.3507924
Tatdn3	1.82010298	1.79665304	1.87800183	1.43229169	0.70843416
Shfm1	1018.55081	503.807491	663.865851	422.868516	356.010446
Pigv	14.3239549	9.73253242	9.60903488	10.0563338	4.14008262
Plch2	2.94293441	2.19160139	0.57313826	0.45031005	0.16845163
Gm17495	1.88180236	0.45464694	0.87125945	0	0
Nsmf	28.0905066	28.4508781	26.0935445	19.2300389	12.9265591
Pspc1	8.70404446	6.23079273	8.84798895	4.18458341	4.07057936
Ech1	79.251113	71.1375139	64.9483462	45.612575	26.358321
Klre1	1.99992038	5.83272683	5.22498749	2.55741722	0.62960768
Fam3c	99.0674845	135.554815	117.925984	111.061854	49.758086
Zfp260	28.8328326	30.5629248	30.127491	16.9491448	13.0924851
Dqx1	1.67262408	2.25378119	0.80309349	1.09957188	0.91012004
Ccr2	3.92469349	5.56416787	6.64588128	3.77001203	0.62609914
Hcfc2	4.48227874	3.49805184	3.56843356	2.08264577	2.58439969
Neu2	3.174737	6.07943475	4.88959368	0.77328098	5.71119538
4930427A07Rik	7.055691	8.01264413	3.7436889	5.85314219	2.28967015
Glod4	45.9557606	46.0496487	53.6595837	47.3880207	22.0173033
Trmt2b	12.1353254	6.99524242	7.55227236	4.8547523	2.91595928
Cd59a	57.0241599	41.8303159	38.7759757	47.7170196	9.85607647
Hoxb3	13.1089341	8.97215983	8.63338093	10.8122819	4.12891338
Atp1b1	384.05431	629.656047	370.587272	166.953171	289.357761
Fbxo3	14.3986315	12.6505511	13.3963322	11.7098271	3.79854484
Gpr65	1.88315079	1.47866135	1.20938716	1.75677548	0.02433975
Mir5133	4.23452529	3.32497478	2.35266164	0.77752978	2.03600407
C3ar1	3.15515967	4.43082472	2.90758113	4.10830167	0.48622878
Anapc13	157.754749	137.675144	122.545993	96.1144958	41.8827993
Galnt1	83.7381026	91.1522762	76.8117189	33.8702077	49.8516916
Pgrmc2	40.0552014	51.4089532	71.9719906	42.7868922	27.9969179
Znrf1	20.6438694	24.4226005	25.5197172	15.5518262	12.3245206
Htatip2	8.20053353	6.91861641	7.43199779	3.56415489	2.79638463
Yeats2	4.58186708	4.51805396	2.89426929	4.10055906	2.41221016
Eci1	60.3701251	40.6914741	51.0864697	51.8578865	12.9326381
Cd37	0.42552489	1.50356119	0.93581946	0.87900185	0.17049735
Slc35d1	8.34024374	10.1241057	11.2504339	7.55356874	4.28102756
Emc9	3.40234902	5.40669811	3.0755084	4.16485515	2.20714644
Zmat1	2.9227049	1.59329222	1.89618842	0.9058233	0.41770147
Cd82	50.1724987	59.7099514	37.2136625	32.197669	21.4866884
Cntn4	0.23843397	2.58703705	2.46878936	0.84575908	0
Arhgef6	4.08197488	2.60001529	2.92078814	2.04414263	0.16012347
Taco1	8.73066001	9.72772725	18.2013072	5.91085464	2.65782032
Zfp870	4.27274476	2.53315865	3.06713749	2.14789664	1.12487687
Vps45	6.0841066	6.25920804	4.66799366	3.46542593	3.16410228
Tysnd1	9.67049989	9.36287297	6.57736681	6.94028657	3.17750824
Senp8	10.2060303	8.74832887	8.10495148	6.36423258	2.58342353
Zfp383	3.53437401	1.32897465	1.54419611	1.0054469	0.51858526
Ankra2	20.3724419	14.9977543	13.6560977	11.1126802	5.35156936
Golph3	212.813148	265.545388	272.589108	104.772137	122.808986
Tctex1d2	29.1437345	13.7952202	13.4933161	14.8014068	5.71436324
Nupr1l	1.66144432	3.34297623	5.57695694	2.19268349	1.24818721
Zfyve9	0.69118381	2.4540459	1.80876674	0.38625673	0.60204421
Tmem106c	20.8597637	19.1038612	13.8866992	20.9086555	4.15502563
Tmem132c	2.65285183	3.31483012	5.51531129	3.73527555	0.2350716
Il17re	38.5777709	28.1351542	20.7270644	29.8148367	6.30512534
Mllt4	22.5675019	23.2962603	29.4522986	23.4354194	13.1723001
Tssk6	1.85033168	2.19897105	3.24152511	1.5610222	1.44269
Lamtor4	67.3680624	70.2384808	68.1654871	62.0543234	20.2594399
Rasip1	3.93090768	3.31400405	4.29131852	1.57652056	3.18319417
Efcab4a	9.4319219	14.7209464	9.52128348	6.99131546	4.02695133
Atp5l	233.897678	174.474973	226.826239	112.153744	84.5490707
Fkbp7	9.73092027	6.78546634	10.8262607	5.60029242	3.49158827
Uqcr11	278.196657	186.49753	228.659645	193.06635	100.559611
Lsp1	5.33004216	3.57607034	5.32944968	3.99744587	1.74458829
Sox9	7.1566133	3.32224807	8.31640732	0.92418397	6.40298561
Tle6	2.36250786	2.23623117	0.53941919	0	0.31121055
Mrpl19	18.4418658	19.3041386	16.0744822	8.50151058	7.61913442
Rab3gap2	2.26766465	3.7003905	4.78724672	2.46744424	3.14083054
Fancg	4.37614198	2.98552741	3.36579291	2.83212441	0.65154121
Gpr34	3.50452705	1.96940814	2.95880049	1.86107364	0.60572373
Mmab	2.75081114	2.21138095	1.23317171	1.04225953	0.68230334
Plk2	27.891326	49.5958983	63.4893867	6.34820723	30.3358064
Impa1	27.654169	29.7266295	26.0019915	27.4604624	12.597237
Rps6ka6	1.16395459	2.37388092	1.56771395	0.58287698	0.31494979
Syne4	12.8111591	10.2106685	12.6656831	9.46243401	3.24196807
2310061J03Rik	2.22202794	3.2619234	4.02566547	3.10435963	1.43224089
Etfb	107.700279	76.8635596	82.2277797	66.6912404	26.648981
Kdelc2	8.69727802	5.69096259	4.80561054	5.35607916	1.76390363
Gkap1	18.2575547	19.227028	24.7029472	12.6602131	4.81952653
Zdhhc1	6.34972634	5.75864911	4.58620117	4.92599561	1.55194922
Gm15408	2.36445575	1.55953132	2.27702615	0.91172273	0
Tha1	1.84293905	2.17063027	2.55979815	1.56182068	0.3124086
Ccdc25	33.0597205	20.6703843	35.7646122	19.5174987	19.0539019
Dlx5	85.1091396	66.6825346	69.2322838	50.3315043	31.5179994
Rasgrp1	1.865016	3.47047906	0.92263048	0.64502225	0.3992243
Cml1	1.48354981	0.91165481	1.97102315	0.76983908	0.82702176
Nav1	8.35641295	10.2192645	9.84265275	4.1079297	4.36740707
Zfp111	3.69295242	2.23939428	1.97220365	2.57639843	0.9302908
Ilf3	14.3061619	15.4178161	8.91522921	12.7234203	6.29175638
Smchd1	9.32652995	4.51890868	8.82470651	6.42190172	3.91485912
Klk8	2.41707473	2.67255537	2.10114158	1.76620417	0.03952908
Rxfp1	1.26012927	1.23682637	1.64421119	1.49871682	0.22950126
Tmem219	30.5334394	31.5689448	32.9430781	20.3582664	8.91255029
Lrrc61	2.08408492	3.81835076	1.7638225	2.34541456	1.47254635
Ube2e3	101.482779	64.3800671	62.0329153	41.0413459	30.0166179
Ndufb2	63.6351432	45.915344	59.3232681	45.264195	27.2198082
Gm5141	26.8881379	47.9252677	46.3522415	23.1518442	14.6461071
Lenep	11.4218079	21.5396644	28.6557475	16.8495824	8.2141182
BC029722	6.84957315	7.9569756	10.4696264	6.03005107	3.79712317
Hgsnat	19.7168602	22.5728429	19.880386	15.2859045	6.63214961
Tmem175	8.3061528	6.75261844	6.11813102	7.10580686	2.01701271
Ror2	6.32405553	7.14542942	12.7462506	5.17262945	5.51533159
Cd2	0.6840387	0.85042624	1.74187448	0.47100362	0.22839789
Psmg4	36.8849852	24.3245567	38.3963657	23.3503258	7.82412653
Rab11fip2	3.93629771	2.5770742	1.78771328	2.47159835	1.4181706
Mycbp2	12.984117	8.0472553	14.6909173	8.83917573	4.11664972
Slc9a3r2	35.971889	59.1904437	68.3845875	32.0437349	18.3607214
Zfp943	12.2122842	11.5556859	11.5513334	7.16072503	5.14164255
C1s	9.87460089	5.6779653	7.32306985	7.5815014	1.75066793
Smarcd2	37.5722979	24.0941881	17.6166713	20.1791877	10.3276289
Kazn	4.04523608	2.77233511	3.40081384	2.12569935	0.87439326
Tdrd3	9.16724028	7.9071464	8.28215587	5.43206949	2.92825518
Dusp19	26.1649089	15.2598842	16.8813791	9.41220256	5.82225724
Yod1	3.33982	3.30481815	2.79282514	2.26838776	1.44742315
Slc22a23	4.14158387	7.71632444	9.66005152	2.74878108	5.97729451
2010320M18Rik	88.3202614	95.6048519	100.506547	54.164081	29.2501363
Dhrs7b	20.7341845	16.2806066	14.6191202	12.1480427	6.14404767
Ccdc90a	24.6564859	28.0355828	20.9739389	17.6750582	10.7610013
Ticam1	5.66857796	6.80315687	4.75825124	3.68104026	3.82236382
H2‐DMb1	4.87564034	4.54620383	4.09150579	3.03079169	2.23844165
Abcd1	2.91448892	4.426391	2.41468908	2.20044813	1.29068649
Csad	19.9233948	26.9926297	20.6615281	11.2602789	6.76001291
Akap17b	2.22565493	2.43831484	1.49171417	0.91463685	1.05313218
Cytip	4.15035164	2.0267562	1.46355929	1.07525522	0.17312839
Mras	4.9329279	3.33998334	4.29823657	1.94517432	1.56824156
Cyp27a1	1.51815574	1.49008129	1.15019013	1.55217981	0.38709213
Bmi1	38.3939719	33.2245838	39.0973437	22.1554882	20.1963219
Pqlc3	8.91179271	8.01946411	3.58904232	1.6623283	2.99261682
Mettl10	57.9457631	44.1205938	58.9272202	31.5747839	22.8535313
Fbf1	2.852177	1.8297572	2.12409485	2.30654143	1.20601569
L3mbtl3	2.38975702	3.75290322	2.93870039	1.40415814	0.62813103
Fahd2a	5.69139542	4.21832444	4.82685827	2.93000617	2.0459682
Kctd21	4.12371262	6.78594583	3.04666912	4.5914271	3.84943789
Sec1	0.60723436	0.29022857	1.51573639	0.29086539	0.19041172
Pus10	18.7183349	15.0470305	15.7346952	10.7814634	5.58933577
Abtb2	3.00448767	5.52713227	2.95433326	0.73556569	2.30446358
Jak3	1.64426047	1.27854844	2.11384392	1.17248064	1.18970421
Prorsd1	29.7865424	22.7148277	27.2697217	13.1949872	11.1489082
Hist1h2bf	4.62130083	2.82230142	1.9018892	1.25710851	1.23442766
Gstm3	1.30206369	2.08737601	1.30616434	0	0
Pogz	5.56518607	3.80013282	3.54089947	2.90728968	1.49795535
Micall1	4.59813526	7.31275898	5.65601789	4.42718067	4.91816339
Cstf3	10.3283254	11.8786806	21.0242124	8.88241627	8.99150132
Myrip	1.1503112	4.25809033	1.78796356	0.99322	2.23866386
9530059O14Rik	0.67211646	4.72400514	1.3358166	0.49665741	1.3399343
Gsdmc	1.34146633	1.52624997	3.39697212	0.10053702	0
Nme3	26.4640236	25.8859878	26.5125198	23.2182022	8.9749616
Rabl5	20.0431099	10.9041654	13.4557095	12.8151587	6.71143821
Ino80e	8.90608776	4.88109954	8.08085003	4.4449617	5.02935927
BC026585	3.84490955	2.82042469	3.74771029	1.7649745	0.60811603
Snx19	6.20223071	6.05288225	3.01870421	4.33275346	2.31060679
Ankrd46	48.5143416	38.0524593	57.1709427	38.9395064	24.864426
Tpmt	4.15529366	3.03644289	3.49187557	0.8159051	1.21260353
Zfp93	6.50259013	4.74116773	3.6643878	3.75252262	1.57814484
Rbl2	6.47047247	6.25391839	5.12679369	6.46711346	1.35775258
Btbd19	21.846801	22.7134841	19.9254051	18.1304025	7.82139238
Cpne1	20.7491739	28.1625363	21.1004341	16.2770999	19.4480805
B9d2	7.40896767	3.91181165	2.89800599	3.69421836	1.17719987
Rab12	27.8366317	27.9969493	24.7247961	27.3137313	12.5053868
Stat2	6.2777753	12.9934324	13.7013717	9.27886411	3.84140928
Zfp868	27.5513659	31.7104701	28.6716666	18.2110772	12.6307512
Esrra	10.7328784	21.6055141	16.3486548	5.76800858	5.22108209
Ubr7	18.8987373	10.092686	12.7762497	10.5283806	7.09748384
Pradc1	14.1895806	9.83792305	6.4764757	5.08154105	4.55639696
Pawr	78.6250349	88.0114275	65.6042529	41.1686704	35.3338036
Atxn7l2	1.73262543	1.70058491	0.62169859	1.23278883	0.79835342
2210039B01Rik	1.63186134	1.23206476	0.40682856	0.80671521	0.1005918
C030006K11Rik	3.79595777	4.86457869	4.50980646	4.40557509	1.86531216
C8g	2.90181466	2.89993755	1.70993072	1.01720522	0.67647169
Wibg	14.2144863	11.1613003	18.184344	10.2393006	11.5309839
Amacr	13.4222118	20.1835653	27.202555	11.7647049	6.53471648
Zfp358	27.5983438	36.6296121	23.7733771	21.174559	13.5153053
Zdhhc4	25.2901203	25.9446843	24.9435736	17.0802381	10.1331287
Mcts2	47.1656874	42.1230389	48.9915892	25.3787675	15.5786324
Zfp867	4.45320154	5.47763283	4.91999561	3.79011584	2.22078794
Krt36	3.09412821	2.27576051	0.68907787	0	0.75326036
Fbxl21	2.62174223	3.1530575	9.74149583	3.01634071	2.31368808
Zkscan7	1.17076642	1.1950807	2.22242753	1.47793474	0.28145837
Bbs10	3.00831259	4.46389639	2.9391891	2.2616994	0.89215386
Cndp2	63.0178297	61.8399916	40.2762825	52.7764921	21.1220945
Tmem44	1.93743761	0.71234899	0.64072723	0.59291	0.13554157
Arhgap39	1.9761118	4.07448947	2.40908038	1.61845527	0.53544791
Galk2	18.1143582	16.6008602	13.1552997	14.8961746	5.64048534
Slc46a1	12.8837685	12.0099975	9.32999976	8.18887743	5.10926316
Zbtb24	6.56493519	6.25814044	6.50562329	4.31417793	3.11957152
Iah1	65.0708436	53.3058807	59.341613	39.7623354	16.3657202
Leprel1	3.91618242	3.43945567	1.36994399	0.74570916	0.55790859
Herc6	2.50766679	1.79444083	3.44305353	1.56507509	0.27717527
Mybpc1	0.4750412	2.49527622	0.54605862	0.07519441	0
Gulo	19.5491113	21.3182996	11.6504358	13.4277504	7.98281388
C130036L24Rik	1.49379068	0.80891961	1.52631866	0.80393877	0.66044155
Ctsf	3.36789485	3.4222829	5.22826933	4.48644523	0.60856763
Eed	33.1756557	18.2944632	28.5509575	17.0890477	17.1485572
Trem2	5.40585559	3.52981179	3.84651282	2.82108971	0.5471983
Fes	3.39988533	1.81681822	1.72715915	1.49566101	1.53253166
Ctso	24.154576	18.9811476	14.8820214	20.0606285	7.65776527
Plekha1	34.0294664	53.9645108	58.4576083	47.3586106	24.0280359
Athl1	4.69085767	4.59550631	5.03377329	4.97070212	2.12542856
Capn1	18.9442921	27.8408026	15.0820529	12.6831651	10.3335767
2610507I01Rik	6.73442577	11.1398673	9.97755297	6.51251446	2.24859887
Prkd2	2.89868303	2.75308265	2.04122777	2.78871806	1.25184173
Hyal3	1.28285291	0.67153589	1.61686564	1.14505068	0.51400758
Shmt1	11.7159985	5.8519556	14.3578819	10.1764151	2.65715785
Aff1	6.58564379	7.09969779	5.36613247	3.24858457	2.55259377
Nudt13	3.20501368	2.15128372	2.91992382	2.46787889	0.14498695
Tsga10	0.86309589	1.56625871	1.52738484	0.65152422	0.33813636
Zfp442	2.47549798	2.49914048	3.25284249	1.73159198	0.90685358
Igfbp6	9.76404661	6.95175098	5.15310165	1.11472073	3.60815512
Ptbp3	97.1312449	67.7277428	73.5243474	42.0626792	67.5424917
1810011H11Rik	1.59376838	1.46000929	2.58265707	0.73160643	0.31929188
2610008E11Rik	5.07321964	4.23779003	3.18416907	3.70912407	1.03798174
Znrd1as	12.4816642	13.1025106	9.7654321	6.31175912	3.36972219
Acaa1a	37.9845109	51.9036506	38.5048719	24.6562742	12.6036141
Ston2	2.51067641	2.9596845	3.79541823	1.05268229	1.66340874
Ndrg3	6.7083214	6.94687984	6.34683747	6.11417191	3.23322201
Thtpa	11.9673442	12.2465415	6.0831648	7.52853682	4.85944472
C1qtnf9	7.43612014	21.2836825	58.7666772	14.2202556	4.06416226
Snx5	180.850434	171.379099	154.92666	96.3857962	62.768762
Myl6b	2.03389285	3.39367796	2.35419033	1.22540512	1.83359431
Rpa1	19.3787474	12.3313102	12.4036119	10.8713983	5.19882429
Slco5a1	2.43653123	1.60634841	1.60345505	1.17474508	0.9209982
Zfp958	17.2351098	10.4968369	9.74446343	6.77053565	3.43070853
Mks1	1.43382996	1.60217389	1.8128261	1.18980138	0.81755822
Ptprg	7.2733386	5.1316047	5.92679584	3.47883236	3.41748858
Slc12a7	4.19490401	5.16038678	4.37879402	2.43913971	1.17146303
Zfp825	21.9258392	23.1772139	26.3720384	17.7921076	11.5623832
Rundc3a	1.4129913	1.3448355	1.5066509	1.41517567	0.90927255
Tma7	483.098843	341.230104	449.427315	268.991126	335.120303
Sdk1	0.22214719	0.88418629	1.69529082	0.60481642	0.17801782
Cenpj	2.07756701	1.60784593	1.24562191	0.80960756	0.64678011
Synj2bp	13.0323034	12.0698695	13.6182711	10.2143693	6.3726131
Ppfibp2	5.53996179	7.99666271	1.67168906	3.9854152	3.42345851
Xrcc4	24.1540389	26.8766287	28.2993613	23.4912503	13.1065842
Zfp606	6.80134371	6.92665122	5.52714344	4.41754075	2.58684078
Sumo1	653.566044	539.270954	447.296163	253.966183	217.502767
Lekr1	2.95862202	2.28720318	2.37246889	1.90418424	1.45431129
Zc2hc1c	2.42979745	3.06176615	2.3038447	1.64301306	0.27982564
Shroom3	7.07238647	6.23313662	9.69239688	2.02268131	7.57035577
Diap3	4.45130987	3.07285922	1.06480913	0.68111255	0.44588257
Lair1	3.00805629	1.39967048	1.27234871	1.84109841	0.25295457
Pex11c	12.9149261	17.5532683	12.9779446	11.0594289	8.30735466
Trim61	2.92560294	2.45800513	2.16724924	1.39669324	1.38321616
Mcu	10.7399475	27.8844054	16.1895669	8.04656686	8.71563811
Mir10a	0	0	2.19581753	0	0
Fam174a	45.2179774	37.2065782	37.97347	32.3359393	14.7074801
Cd3g	1.14741976	1.50160151	0.47221882	0.70228496	0.10216508
Ido1	1.42585045	1.18544438	3.14545566	1.27054866	0.30245462
Gpt	2.86931434	2.19448335	1.7252852	1.53950896	0.89584179
Marveld3	14.3038433	7.95628597	10.4001592	9.82574446	5.20128631
Dnase2a	2.65927158	2.36856389	1.80089113	1.63976913	1.27224438
Usp53	10.5274476	14.899598	7.16075024	7.18914801	8.05863327
Naca	37.556519	38.5235718	42.7691186	28.8198917	20.3858984
Mtrf1	4.7280226	3.95996996	3.43759833	2.41151152	1.45939139
Fli1	1.90121544	2.81981988	2.71898688	2.50184751	0.98183718
Dym	5.75319641	5.53283338	3.47012038	3.58588803	2.2628676
Gm609	3.1345864	1.35129983	0.76832918	0.11285541	0
Phkb	11.7231324	10.82428	10.9339062	7.84518619	2.96544264
Tshz2	2.93344193	1.63237858	1.40540052	1.22947941	0.60300975
Gpx1	632.497333	1084.24665	1640.89124	1063.70381	283.847228
Sdccag8	2.41524776	2.63941297	1.91368977	1.73734412	0.93780052
Aldoc	6.13035859	1.11314373	28.3830703	1.33669338	2.82472636
Bcar1	13.503732	21.1127279	46.4443198	13.0000892	16.8932248
Rbm12	9.94165416	9.11638606	13.132768	8.04221097	5.73829007
6720468P15Rik	2.37465536	0.11474423	3.01079182	0.1006215	0.55624163
Gatc	22.8993215	15.2720563	11.4857197	17.6206537	7.82244143
Nenf	84.6364481	44.5900341	60.4767525	61.8715184	23.5873237
Lsm14b	4.91532467	7.92115538	6.12428587	3.56761591	5.23811728
Ikzf3	2.22782736	2.38654973	2.13660869	0.83274236	0.15302324
Hps3	5.96172725	3.81594159	4.27770562	2.5680717	1.8566334
Dynlt3	137.026328	144.100669	169.487435	96.0753061	67.111851
8430419L09Rik	27.9190935	45.6571411	37.2295149	26.1583628	10.2553204
Slc2a4	1.34028278	1.56045237	0.92011135	0.19093839	0.35183959
Sytl4	0.96311572	1.97998498	1.63772373	0.88422089	0.51920655
2610002M06Rik	11.1644735	10.3355996	7.30905364	5.66820671	4.4107031
Tmem241	1.98078899	1.57552651	0.52405113	1.01554284	0.53597372
4933432I03Rik	39.6432985	30.8385926	16.1064561	3.04709596	19.2579247
Aes	364.362191	289.399618	215.781019	146.114119	126.876198
Slc5a5	4.7216114	13.6230848	2.06232862	0.69520934	2.89129266
Osbpl8	3.89337129	6.04976185	4.69435822	2.61176948	2.75462165
Chst2	2.74762486	3.15004339	3.99535367	2.98293905	1.30601735
Rabgap1l	26.5822337	28.0710706	39.9066522	14.3160038	19.6054883
Engase	1.39668076	2.14114133	4.0492734	0.27477212	0.2131869
Tnfaip3	2.75322476	2.73016971	1.81352068	1.0162881	2.08119937
Serhl	2.28980329	4.42000304	2.73874827	1.44528395	0.30582261
Zfp764	3.19818095	3.89955375	2.38361771	3.07942714	2.25032028
Znhit3	53.3369261	31.2540864	44.5362279	26.4328485	32.3751572
Wdr13	11.8622728	22.9350209	24.0370174	12.1069083	6.34545783
H2‐T10	2.61116879	1.66145443	1.41738998	1.09530515	1.62346199
Gt(ROSA)26Sor	78.7906822	70.1669178	94.1162438	55.0548282	63.7579835
Rfx3	2.06644472	0.72906684	1.02785699	0.78194141	0.40839235
Sesn2	9.99666954	16.373904	5.04913974	2.72852916	6.14098703
Tap1	15.4518546	10.2409223	12.2202828	8.30059161	2.74572978
Sertad4	5.0428328	3.76393793	3.00060628	4.03122098	0.50705541
Tgfbrap1	14.9428736	26.1663147	16.5846077	21.3836132	3.83107976
Zfp689	1.11657684	0.82017881	2.05118829	0.89283867	0.40408955
1810034E14Rik	2.93126906	3.89291442	2.78132825	0.79737793	1.01498908
Lamtor2	86.3398201	77.4794822	82.487366	80.9447986	38.4380452
Rpl31	91.0777787	90.2721364	116.191693	79.5078493	47.4195584
Chchd2	588.051785	376.888448	452.688294	257.900646	218.160446
Podxl	7.58559991	11.3553191	2.97959668	2.31391694	5.89244279
Kank1	5.96668878	2.80158318	3.89544524	2.44569764	1.46823115
0610009B22Rik	328.683321	225.171474	295.012919	168.162575	88.936243
Vwa5a	19.1226966	22.0363363	33.0904472	21.1567779	8.46024912
Zfp523	12.4556251	14.5570632	9.66337839	9.36017409	4.23917268
Gm9992	1.02341007	3.85722121	10.3863685	3.15697589	0.42645743
2310014F07Rik	1.92313294	2.01340742	0.64756013	0.10700589	0
Ntm	3.31829394	1.24612993	1.26915338	0.72850412	0.2890345
Slc39a11	14.1981687	15.2941564	11.9360541	10.7643355	6.46071166
Ptpn21	2.20478699	1.56166034	0.82084848	1.3668426	1.06554901
2210408F21Rik	2.00458604	2.45939537	1.74020121	1.09272436	0.55219195
Mmaa	6.07847264	5.72742401	4.09929706	4.08538157	2.2976362
9430083A17Rik	2.9443405	5.41159105	3.69580325	3.38394481	0.61170914
Trib1	10.5446622	8.34332468	6.67444536	3.40541047	8.20335336
Cep57	28.0527247	16.0295829	25.8489353	13.6460168	7.38919801
Dnajc15	74.5707746	36.7440499	43.0522557	26.4240135	19.6771833
Ift81	7.42750091	6.33548583	6.81633865	6.75818339	2.79887293
Tmtc2	3.69057693	3.52979603	3.77938445	2.78660531	1.38198442
Map2k6	13.0170621	15.257188	10.0641637	12.4728735	7.46896518
Aldh18a1	14.5695524	11.4981719	21.3324887	8.5552525	5.71912984
Tmem251	59.4752256	47.0402531	73.6145549	41.8840127	34.6070002
Commd6	217.248086	163.152522	177.497342	114.676312	71.1298381
Scoc	30.5185647	22.225086	29.5189437	22.7923314	8.84473606
Sepsecs	1.4228005	1.4895887	1.66254756	0.93303573	1.08586884
1700040L02Rik	3.84527733	3.76382847	5.02395246	4.40076829	0.42211178
Slc25a13	38.6424169	13.7278219	23.519583	9.31989728	9.90916179
Dync2li1	4.81593487	3.16411315	5.3695764	4.46654822	0.70884241
Zfp799	2.68898343	3.24553264	2.80297867	2.49692585	1.24363836
A930005H10Rik	5.75533772	6.48713228	5.97403025	4.30380717	1.45056233
Dennd6a	20.4964747	23.3159647	18.7347993	14.859967	8.5059059
Naa20	153.458908	109.093784	163.32798	85.1050126	78.0294505
Dse	23.6614249	23.9601224	32.7579595	16.8910753	12.838632
Mrpl41	61.2284745	46.0693575	65.1948465	39.1142724	21.0064979
Hist1h2bj	4.10782296	1.88153428	1.74339843	0.78569282	0.54863452
Traf5	1.66144432	0.83865606	0.71429005	0.49028948	0.28529993
Fam58b	37.0278793	33.3833987	26.0484236	30.277289	14.1317091
Otud7b	9.40220385	11.2320201	9.41442196	5.8606031	7.06355935
Dennd5b	1.33964825	1.60388695	0.83518332	0.57233403	0.70151345
D630032N06Rik	0.71325285	0.88007926	2.7361945	0.74837241	0.65321797
Ddx26b	2.38036367	1.89596907	1.71259928	1.69798782	0.411692
Ms4a6b	4.02016343	3.03524668	3.90157857	1.66797882	0.68148407
Camkk2	4.66845804	5.32050371	2.24791711	2.4246715	1.47505142
2310008H09Rik	43.7959476	49.6991303	25.9530607	34.4368658	16.7052465
Camkmt	3.67070664	3.62951381	2.09815782	1.9970466	1.12576829
Mamdc2	2.67645341	3.69691773	2.56880391	1.45280204	0.21917439
Apc	5.54212929	5.77075026	6.61620335	4.23690959	3.33718877
Terf2ip	8.62483029	8.2376966	10.7213125	8.13457312	5.35787566
Galnt7	16.3786158	11.7511357	16.2477389	11.2777058	6.19278943
Zfp952	5.70168695	5.20897976	6.54438303	3.16465992	2.04999257
Actr8	9.19297274	12.8434009	12.9823441	11.5449058	6.0609758
Smyd4	2.54023357	2.32946768	1.87147087	0.88519455	0.43089727
Ehbp1	1.12389402	1.72440253	0.8612755	1.19787026	0.66253487
2010003O02Rik	51.1660949	53.7254539	56.4831832	38.8693116	14.4712904
Nbeal2	2.02235502	2.21617142	1.22786684	1.2581974	0.76578547
Svip	8.80912159	7.36016827	3.00524949	2.720513	1.63153051
Oma1	21.7372298	15.2845491	14.5946554	10.5237364	6.3571481
Eml2	1.66413914	8.10592034	6.9474071	3.78070491	4.54314229
Bcl7c	22.9761393	21.2247094	18.2093573	9.05802046	7.310626
Igbp1	73.4168494	93.4703416	96.345472	71.8595273	43.4445892
Fam167b	1.45068377	0.92211695	2.87852139	0.95132086	1.1071491
Hdac6	8.37581334	9.3458924	6.83741105	5.27582674	2.30881485
Adamts6	1.66342551	2.15564831	0.42577549	0.43455884	0.04334918
Nsmce4a	39.8541127	30.6514016	33.2433002	27.0352793	13.0006308
Atxn7l1	3.52124831	3.92592627	5.6743251	3.06486317	3.92114014
Med24	8.6438955	10.1658422	16.7873792	5.40759419	3.17222569
Acvr2a	9.60784794	8.03987212	6.67519252	4.8477565	3.45868625
Smndc1	46.6148501	37.3010535	56.7476749	31.4985545	42.002684
Mastl	10.8218883	8.56345395	8.85265644	10.630363	2.10063217
Rfng	19.5819804	37.9803799	38.3749697	8.87588052	21.7862689
Polr3k	123.279339	70.3296479	85.2335779	61.5094995	32.3098023
Palm	1.89411546	1.79631417	2.3005972	1.44536656	0.47309638
Ms4a6d	6.70667267	4.59384917	4.97702624	2.18343519	1.33407026
Coq10a	7.12724489	5.71890356	7.10553239	6.7583372	2.20952868
Tmem69	10.3678195	9.07611836	9.47510304	8.25104658	2.95019158
Fbxo7	16.043597	22.018526	26.1262701	11.7136551	5.90182091
Zfp688	18.8703117	20.0584994	14.5613392	15.7924257	8.69242812
Pycr2	38.5397415	34.9369801	152.000971	45.7915926	39.7404905
1700009P17Rik	2.01815674	1.23802252	0.99278984	1.27382538	0.05053911
Polb	20.0365175	15.3154788	23.4748463	11.2225365	9.62524932
Emc2	35.093841	34.7122025	32.9328537	24.3807108	14.6488721
Cyp39a1	22.4705855	20.7132968	16.2186981	13.7271061	7.00075074
Marf1	9.01122252	13.9732868	9.34742414	5.05789879	3.94370361
Orc6	70.2188749	33.496608	56.8071536	24.4329566	20.4416942
Mccc1	5.68579453	10.3370812	9.49716854	8.8740605	2.38330366
5830454E08Rik	2.89634691	2.6188092	4.63249526	2.33677522	0.8439963
Hsd17b10	83.0357408	59.7480813	51.6354567	68.2817944	27.3620173
Ptprc	1.5652647	3.17204706	2.66435678	2.64162517	0.60871707
Taok2	9.74765086	9.9448817	5.81569481	8.27079793	4.23585855
Mir199a‐1	0.93159557	2.9259778	0	0.85528275	0
Tmem194b	5.05323346	2.76088507	3.14912841	3.19282042	1.35493651
Usp54	4.36752655	4.91942688	2.43585301	3.26311111	1.24709821
Tead3	3.32247369	4.62086238	4.66510684	2.47214596	1.70537093
Med9	24.3705152	21.5151139	20.7028498	11.1870871	7.38853915
Idnk	28.6137327	16.8422845	16.3451077	21.6339556	10.5829093
Pmch	0.61026982	2.25902698	0.32291434	0.32015932	0.27945154
Terf1	30.8287963	19.5348885	19.7831999	14.188371	11.3283117
Pcyt1a	8.4622023	7.66984747	4.32483891	4.41473443	4.54762454
Amt	1.62414019	1.66173456	0.91147143	1.35554248	0.23663745
Ammecr1l	67.1121236	75.4629517	62.2011565	38.5971707	23.1951564
Bbip1	55.1410894	40.1708964	55.1561149	31.7983616	26.3581011
Thbs1	15.7690452	34.5247226	14.6643295	1.75419938	17.6713362
Zmym3	3.51330537	4.73881713	3.20336791	3.41349835	1.68405228
Gjd3	0	0.97882173	1.94790265	0.21458707	0.12486843
Zfp472	18.1097075	10.385531	15.6065593	8.37715971	6.28341463
Col15a1	1.60607846	2.16601572	2.70194129	1.98102717	0.72702583
Cyhr1	25.1861014	25.8655181	19.3376488	20.2107633	11.2260876
Klhl22	9.36577359	15.2166493	7.65768298	6.6090031	3.57298753
Fam120c	2.85993915	2.78379814	2.27217397	2.09742365	0.56956335
D10Wsu52e	65.2775932	65.4870499	84.3375899	64.4984327	36.017759
Usmg5	4.78091566	7.50800756	5.84370794	2.98471108	2.75845712
Arl3	39.7458108	30.1496739	24.3890682	22.505798	13.6683079
Tmem126a	52.3566437	40.6428248	36.6566797	25.9487875	12.416258
Zfp235	3.5326147	4.12366223	3.06123171	4.00635056	1.08995814
Ranbp6	6.8333597	6.1651741	6.7866417	4.58833629	2.14741885
Poc5	6.19220791	5.04786642	3.24521994	2.90170113	1.48174733
Uhrf2	24.2009464	15.8910863	22.9729186	15.0774408	5.89336282
Cntln	1.95785673	0.47302182	1.08950964	0.68269539	0.20365918
Nsg2	1.56996488	1.84911883	0.86148882	0.42706941	0
Nfib	13.4066906	8.47766202	9.24021048	9.60510815	3.35472491
Ikzf1	3.56182383	6.02990583	3.68415056	1.73938968	1.95345448
4732490B19Rik	9.22454807	10.8228881	13.2570463	10.6473262	3.41831154
Atox1	322.336319	310.267212	283.450966	231.824586	150.687543
Zfp324	2.43270467	1.71915693	2.80172801	0.83753499	0.56046681
Zfp455	1.37059967	1.52934127	2.02619224	0.68435235	0.4046483
Ap3m2	2.85900687	2.90952076	3.41374574	2.24490137	1.20582493
Spin2	4.09445524	7.76302686	3.65269034	3.25478965	3.04101475
0610009B14Rik	1.66675245	1.85405409	2.25077143	1.78525474	0.44521779
Casp7	31.3982209	20.5777579	28.7657705	16.3119061	9.87752335
Slc25a4	508.250239	338.385629	294.335839	352.614675	168.915316
Coa6	70.596141	47.7149132	59.5935668	41.822542	25.1356221
Nrip1	18.4879244	23.5163595	20.5994253	6.62282318	9.14995542
Snord1c	0.8360473	0	3.87083218	0.76756145	0
Gm15446	1.68179728	2.80618438	2.76423838	1.54403076	1.21293859
Sltm	18.7895167	18.6518516	48.3494873	18.4689527	17.695421
Chn2	3.94641834	1.95552277	1.57880829	1.96986392	0.84434755
Rgl2	8.67271109	8.7256838	8.48265224	8.28809716	4.24813864
Plac8	383.493456	227.404388	554.049562	179.523359	264.365572
Mat2a	106.352731	72.0224636	113.081862	98.7082925	52.1828907
Magohb	29.7113311	21.5915007	17.5794399	13.6778247	6.41279262
Klf8	2.2532197	2.44706804	1.19225453	0.09402929	0.49244164
Uba3	71.6447707	52.9442688	56.5290438	32.6428833	28.7490775
Sumo3	118.626158	89.7775199	73.4647615	69.0195686	40.7704438
Zfp493	3.42799835	1.50233579	2.03005747	1.09785684	0.159711
Cops3	35.6873678	30.9320323	33.1616121	20.6065251	11.4459139
Topors	18.9083348	25.0692021	34.6247047	22.2213234	17.8181269
Adcy9	2.83847157	4.1014239	2.24902558	1.41043936	0.53934084
Abi2	8.6126587	7.13984623	3.35584433	5.23134112	3.09660746
Zrsr1	2.77280336	3.3057296	4.83940194	1.35946546	0.83760808
Prickle1	2.23491714	6.96934248	5.85372164	3.8398741	3.6842453
Gm561	39.2122578	34.2479646	44.861174	23.7392207	10.9296602
Srek1	23.930748	26.579012	21.5798656	23.9891761	11.8328434
Zc4h2	19.2532647	19.4405568	20.0813863	10.2482341	6.82466537
Snora16a	0.95899543	1.50601799	0	0.88043813	0
Gpr114	2.1968477	1.41629777	0.40684917	0.14861296	0
Zfp101	7.09186078	4.78109701	4.46641024	4.66944924	1.53079276
Zswim1	10.550725	11.469378	8.89789813	5.74093903	6.19118107
1810037I17Rik	436.570881	301.614049	300.440034	172.861758	166.249328
Gm9897	5.19596064	4.19219042	2.95745161	1.83810767	1.18419632
Gng10	105.386748	63.502862	70.8445236	71.8972065	29.5814424
AW209491	37.0381766	24.450645	33.7893226	32.4948257	13.8715986
Rpl22	201.069376	181.098663	210.341021	152.69732	83.7655987
Sp4	2.45250202	2.89301618	2.90936309	1.5149029	0.95095857
Zfp937	4.75894555	2.90800756	2.53931066	1.6611272	0.81557851
Etfa	53.8085526	29.9742842	35.6370386	32.3476818	20.0605738
Serinc3	245.046958	223.891956	254.699081	132.673017	92.9429793
BC037704	0.78528879	1.59291817	1.42403119	0.60080073	0.70795325
Abcb6	3.35730144	3.70111751	3.95292075	2.28619734	0.62359711
Sec11c	78.846861	53.4655944	61.0123585	28.6130958	21.9209771
Wbp1	63.533266	48.103651	32.0438095	25.5160434	15.7444816
Acy3	1.77889506	2.86173478	1.8481099	2.2705111	1.2169064
Ccdc173	2.147352	1.8044375	1.22095573	1.34888615	0.66416154
Atl1	4.51251933	2.43485378	2.12140285	1.44469337	1.3722677
Zfp72	5.88466443	5.12360447	7.56202353	2.24190139	1.47565509
Bsdc1	71.8824603	120.530148	85.2293485	29.2256454	53.9475993
Homer1	8.9687468	10.0706883	8.06189699	4.1903617	6.12641877
Fabp5	8.96404127	5.80283111	3.35824139	3.20394484	2.35789068
Pcyt1b	7.14597401	13.6898158	16.9872459	4.45388208	8.44473797
Dhx58	4.1365624	4.88269513	5.5327865	2.78002355	1.51659231
Npr1	1.81277267	2.09101243	0.98042037	1.29607424	0.51421833
Hexim2	3.60987313	4.46170372	3.9926513	1.56502277	0.7499786
Dapp1	19.5128242	19.0027477	24.6585047	22.2572934	6.9383256
Asnsd1	74.1569001	67.3334138	53.840293	45.219183	31.0402985
1700123M08Rik	1.22348386	0.9606869	2.43579196	1.46023885	0.14706596
Lonrf3	0.79634356	0.79898648	2.13027074	0.67424597	0.50342986
Gprc5b	2.99757601	4.84322072	1.46792222	1.8126324	0.63517328
Tmlhe	2.22674062	2.70215023	2.5707132	1.72573682	0.67204687
Btf3l4	25.4474417	21.0842518	19.2742807	18.0513872	8.86915047
Rrad	1.86319113	0.74774988	0.69011408	0.53217594	0.19907595
C330018D20Rik	20.2688633	17.4855366	12.3873047	9.85010681	6.93012021
Azin1	66.1714479	83.9152743	120.685896	54.2495577	57.0279556
Pafah1b3	81.327222	164.973019	280.963687	64.2051916	74.3617332
Stxbp6	1.70379674	1.4325452	0.85167258	0.31838271	0
Glul	42.9900211	124.80894	146.230634	29.6981718	105.585571
Slc44a3	6.7504288	8.32074937	6.08567397	7.06276462	1.02065308
Gtf2a1	25.4512037	40.7802487	27.1867625	17.0658813	14.2479373
Cyp4f18	1.64753387	1.41399339	1.52559003	0.77387511	0.33773902
Cnksr3	3.74475479	5.55409649	5.72676599	4.70554346	1.10637847
Wdr25	2.20890179	1.67662823	0.74998284	1.19424126	0.35402103
Dab2	3.62130601	8.56360616	15.8138717	3.37640793	4.08755239
Acaca	6.27424249	9.08212884	7.00658339	6.10496891	3.43677126
Dnalc1	1.23446929	1.69852046	0.82848447	1.10215318	0.38117617
Dnajb4	12.5655596	8.71195645	11.0991187	5.42039463	3.60642378
Stx12	126.797384	189.037715	108.224464	52.6699341	61.8529845
Klhl12	17.3843428	25.5221984	37.5044397	13.2065719	9.49986586
Desi2	13.4664538	11.011483	15.5083196	7.06273131	7.05779303
Mir3473c	0.80508259	1.2643114	2.23648082	0	0
Dhodh	6.83625563	8.54882859	7.03362604	5.34643965	3.74233592
Idh1	28.2459164	15.7310312	14.3655823	5.52530901	9.02394638
Tk2	16.6289808	30.9574547	19.5999588	17.0628909	7.51200667
Rpl3l	1.39009244	1.63726344	0.71931954	0.07507184	0.36039612
Hist3h2a	17.8922525	11.3216227	10.438409	8.99551158	2.96738214
Cep72	4.02186632	1.88915692	2.56037644	1.18684611	0.87767172
Phyh	35.0391168	64.4052577	53.2158017	38.3426624	33.5029027
Rev3l	11.4831273	6.9761182	5.9167657	6.75885893	3.63082619
Rsbn1	9.87820895	10.0932533	10.5563756	4.23636411	4.17935209
Ms4a7	9.84255687	9.48896647	9.46594415	9.55962892	1.73582398
Fam151b	6.69563262	4.77613513	6.2873734	4.32897996	2.26713396
Mrps33	87.2175353	61.7181059	74.7924255	44.5039714	30.3510403
Rnf43	6.47427583	4.26408155	2.19912832	2.04148911	1.12733512
AI462493	33.1159242	34.3212619	31.9727943	36.4550122	10.8791056
Parpbp	1.9232775	1.28434941	0.43274794	0.4125537	0.38890596
Ciz1	4.57313306	7.368241	4.01466638	4.2712208	2.7604111
Jhdm1d	5.50882796	13.0517355	8.67247936	4.57499725	5.1912811
C2cd5	7.42899507	10.7153786	12.3662172	7.4368252	4.38597564
Gm11602	2.71413802	2.64263533	1.57496901	1.06317057	1.07298845
Zfp160	12.629932	11.4012903	8.97421077	5.06408524	3.07072691
Setd7	3.58150117	4.42715238	3.55447216	2.0265876	0.65357317
Med6	61.9640954	44.1155269	61.8885922	35.182448	27.5860976
Atp5j2	279.986457	186.462076	263.313968	177.350141	99.4753741
Pion	7.26122151	6.11173868	4.78887575	1.43879342	2.90241577
Cyb5d2	7.68487924	4.74116773	3.35472122	2.74655194	1.60555259
Tomt	1.16017832	1.67012817	0.71620438	1.10952173	0.46485489
Oplah	0.77202756	1.66382743	1.55145294	1.19136364	0.71080646
Atg10	9.68650951	12.0612478	13.8108598	7.06977043	2.53330027
Cd44	5.57177535	6.15035047	8.70577753	3.86564203	4.09517865
Dnajc4	10.1699183	16.1538358	9.63284237	6.69971491	3.48382918
Commd3	170.246182	118.741227	116.040607	73.6649891	44.0125071
Pbx1	15.4017195	13.5967048	13.4055062	14.7966664	5.47640812
Gm13157	25.7357969	19.8701662	23.3220121	16.980293	8.52127511
Dlg3	12.2185139	11.7836021	8.84989268	7.3084488	4.93274607
Marcks	250.642827	299.460128	289.677693	51.6888273	154.325477
Vps54	37.8510125	36.751697	28.7187489	18.9443678	12.354581
Ugp2	130.190631	126.034453	86.2474662	42.98892	59.5822959
Narg2	4.83948751	4.75317069	6.74441348	3.98240369	2.20495518
Hadh	57.3635385	48.1978291	33.0010948	41.978471	16.1025826
2700081O15Rik	3.73833297	4.61857591	3.17114959	3.85211535	2.03205911
Commd9	42.2349751	43.3169224	32.5308031	31.7946814	19.2129473
Wwc1	13.7701909	17.0990965	12.1498591	10.4207754	10.2465564
4732415M23Rik	1.7624781	0.88965541	0.9325866	0.34673625	0.10088308
Rreb1	4.62925443	7.37132897	3.6349791	2.57112236	4.2237145
Lamp2	388.414846	340.085311	568.391453	302.916976	241.164706
Bik	10.7879359	21.9370764	13.6394498	7.9360807	6.982436
Gimap9	4.10225379	3.66540603	5.98173719	3.72292276	2.04042056
Pgap2	57.7001041	70.1223562	45.4433056	45.1577618	15.8734196
Col4a6	2.06974526	1.07061387	1.40789293	1.10769924	0.29084314
Zbtb3	2.99136191	3.38726788	1.86607381	1.73451838	1.3878122
St3gal2	1.32757578	2.5888685	1.43194812	1.08488886	0.40917457
Hsp90b1	376.663097	316.154606	250.688505	152.419509	214.446796
Ndufb10	94.5826237	67.3320482	62.1917768	47.707268	23.6187159
Arhgef26	3.04446505	5.65660934	15.7198176	6.79789578	1.52605774
Lrrn1	0.86810023	1.51323533	1.51123225	0.19127729	0.13913056
Zfml	15.8241658	13.2403887	14.6249253	10.798771	5.54074224
Gm6297	0.81855677	1.71396189	1.44375331	0.85886134	1.06201607
Egfr	6.20919007	6.14805292	3.22653523	3.14321062	4.20462142
Cby1	27.0986914	17.3427594	15.217663	12.2521739	7.42531957
Sdsl	7.44351791	8.34351404	13.6353185	9.30977754	5.3597372
Ifne	18.3653403	11.3359607	31.1928446	5.22959033	15.4253882
E030030I06Rik	5.86108553	7.46175404	5.11111977	3.57860454	3.92158782
Gm14325	5.73731729	6.29526531	9.12122329	3.33826084	1.9465179
Mki67ip	108.938641	89.0808844	117.45421	109.403037	37.7997305
Bckdha	14.3811453	19.4941414	12.9761298	15.5668215	7.51587513
Hmox2	109.274932	89.6642978	77.6252039	44.2668307	36.3047646
Jrkl	9.49899272	6.71279968	8.52999238	4.72549516	7.08095362
Klhdc1	1.60603443	4.70273241	1.70404003	2.67248434	0.26812436
Slc2a6	4.15205514	3.3561075	1.47004638	2.52260363	0.41590657
Lrrc3b	2.78749546	4.16172339	2.8356584	1.76617691	0.5033829
Mir325	0	1.89646709	8.94592327	0	0
Lysmd4	7.30571396	4.44994259	4.3017383	3.34478003	2.61055482
Ptgr2	17.5813886	15.5325843	14.1877177	12.2287236	6.38525531
Pigl	7.87105486	9.51781554	5.13837204	3.50474949	2.86990153
Fam216a	18.44237	10.9910204	14.3130222	12.2419414	6.4325025
Eif3h	231.161632	243.13851	218.084198	148.601896	106.217072
Mt2	374.849928	707.378815	789.783434	152.797187	442.026492
Nae1	18.0342062	18.6636278	24.013718	12.947505	10.5497593
Ddt	33.9443441	23.7105098	18.9384755	15.6310296	12.1848213
Senp7	5.82181151	7.30249236	7.83508562	4.99386772	2.04601657
Tmcc2	1.14741976	2.80799483	2.86872935	0.54427084	0.22987143
Pcbd2	17.795864	11.0661981	7.18868834	8.00456936	4.59406046
Mrap2	0.18847309	3.30511462	0.52356921	0	0
Zfp672	15.7524806	12.1862197	11.3211482	10.2878096	4.81979112
Dpp8	9.65034448	16.1546035	15.0835702	9.75209051	6.87771063
Zfand2b	29.4682539	30.3128147	27.5195607	17.7443189	13.3560195
Rab3a	5.79759335	6.387343	3.66221807	4.91006571	1.72870917
Eif2ak2	8.68283245	6.08395061	8.66830186	4.62240194	2.91124422
Car7	1.10240828	2.29721599	1.13859849	0	0.13591011
9130008F23Rik	16.3708827	13.5660203	8.72631514	5.90656131	6.82565884
C1d	109.053705	63.7070564	70.8813908	44.6037043	33.2418804
Psg25	0.55282163	1.53597139	0.96474213	0	0
Foxred2	1.98661659	0.51298834	2.85195887	0.53859556	0.42737631
Fam124b	2.3924624	1.46707832	0.44307639	0	0
Pex11b	21.5525893	17.6151772	15.3643642	19.2785356	7.66711441
Gm15545	2.512744	1.59720807	1.93896731	1.26330755	0.76708165
Socs1	7.64618018	12.5047856	13.7997046	9.56843589	6.75170778
Fhl1	7.34907388	5.64586499	5.12484717	0.99956524	3.39900551
Abhd8	8.14735466	4.7722182	3.92426332	4.79561564	2.39568102
Ifngr2	138.992258	113.416978	73.8038669	56.5436932	72.9584517
Lyrm2	71.095451	63.8325157	67.6039385	40.1982894	20.3528968
Crocc	1.77672567	1.06548518	0.54238008	0.57360281	0.1799283
H3f3a	578.117385	557.20706	716.033444	351.540289	219.374392
Trio	3.60175897	3.87920472	4.53793376	2.43186859	2.26356475
Dennd3	3.59347298	2.70565979	2.46143728	2.21447054	1.43981617
Robo2	0.96435828	1.53352944	1.29069429	0.33480063	0.1428686
Cetn3	226.66373	135.366189	187.890882	129.845938	71.7572516
Itm2b	603.060924	450.930002	379.621163	337.730502	173.581986
6720456H20Rik	5.37390312	4.42569563	2.90468197	3.49948003	1.10163245
Phf17	16.6754354	17.9543052	28.8938893	12.7846953	5.26788687
Sema4d	13.0512771	21.8412177	13.0923802	11.0110083	7.80817095
Gulp1	10.6494896	5.81557752	3.42912028	4.72762141	1.28185247
Ppp4r4	1.94767468	1.45981108	1.19687855	0.58520569	0.11351059
Ankmy2	36.1250365	30.6264704	33.6980787	25.3088167	13.7800684
Coq9	32.8947329	31.6361628	29.3186387	23.5872131	11.1120364
Lima1	82.9244153	88.662941	67.4882956	42.8780724	28.2348269
Nudt19	147.150415	605.077595	1532.93655	70.0557565	231.433564
Catsperd	0.78813348	1.7367646	1.60084943	0.95698304	0.16298616
Mcm9	2.15520172	2.74995074	2.04245614	1.76224022	0.94449281
Gatsl3	4.50752634	2.81162273	2.10645286	0.42543134	2.26178832
Zfp456	5.03596033	3.32660506	3.46410537	1.61453229	0.87117082
Ncoa2	9.29780695	12.5439764	10.947673	8.96625412	4.3139587
L3mbtl2	4.84809937	2.68712247	2.74637236	2.12947951	2.0110734
5530601H04Rik	4.87515042	3.88287917	5.43659002	4.01057026	1.58228576
Myo3b	2.81444312	1.94800153	1.26445535	0.44978904	0.35918635
Osgepl1	14.6819135	10.4068943	11.1089323	5.7955428	5.44777493
Rbm4b	13.8263036	14.410041	11.5490403	11.3850754	6.48220675
Atraid	38.2720009	38.896323	37.5128348	33.3302176	12.0175793
Nt5dc1	14.5501782	19.6447736	19.8985858	12.4104173	8.11952824
Limch1	1.55458207	0.9828749	1.31800038	0.34290748	0.606704
Tmem211	0.14277327	0.53250554	2.41275517	0.06553891	0
Npc2	66.611082	112.910721	75.9532745	59.2641204	48.3165028
Egln2	62.4880879	78.8118439	52.1311259	44.9236556	28.1996709
Snx21	9.86755829	16.3383136	8.26320807	11.4225263	4.77880516
Trim12c	7.85472706	7.51074066	11.7020147	3.17297082	3.46890914
Spef1	7.4601712	15.8770483	18.5635811	4.09885949	6.53217971
Zfp595	3.38653696	1.50640326	4.66519943	2.11359207	0.81547805
Pak1	16.4680288	23.2520124	20.2203877	10.9334251	15.1940933
Synpo2	2.52928981	4.51170481	6.31343826	3.26440129	2.71715176
Pid1	2.86080528	6.31657938	5.04906299	4.53456998	1.31280402
2210013O21Rik	35.65312	34.515258	35.6948609	28.2563041	11.4771008
Trmt10c	114.067468	74.9207236	110.00796	64.7346189	47.8130077
Top2a	27.3996595	8.2445608	25.1864522	7.96427759	5.15863148
Trmt2a	8.17801318	7.95033599	6.70397873	6.93930552	3.20580372
Wdr7	2.78901992	2.32682745	1.98791303	1.17077957	0.83811873
Kptn	2.61004943	3.04308789	1.94081507	2.46885743	1.01409218
Kbtbd7	3.48525373	4.79477811	3.93409225	2.49898208	2.45822311
Ttc32	28.2366616	16.7951126	21.2555137	18.7991149	6.42766484
Atpaf1	5.56958207	4.13153089	5.02776245	3.10911799	2.19795232
Fbxo27	2.26385807	2.07386083	10.2485601	2.33821274	1.51178701
Oard1	71.4743099	53.8212349	57.5120402	45.5748907	25.5992382
Ankdd1b	1.77045311	2.03891666	1.34796038	0.57792862	0.58326552
Pdk3	41.7354813	39.5811647	34.6609797	18.76918	26.1287189
1110019D14Rik	6.70400547	5.84511921	6.4496643	3.03745277	1.32562259
Prss36	2.96126261	2.40884158	1.51899497	2.2688324	0.78531269
Rpl7	2103.89825	2445.96691	2907.99317	1769.52777	1176.15641
Etv3	5.45439146	6.28479087	6.62461192	4.32050051	4.8384634
Ephx4	36.0789634	38.0574815	44.6887268	24.8902239	10.5096437
Ramp3	16.129345	5.5775703	11.8198286	8.33567958	1.71195537
Zfp953	4.58069614	1.47843524	2.35502731	2.01243001	0.5404777
Gimap1	1.34323081	1.93931087	2.12651432	1.35254017	0.65972845
Pld4	4.35184177	2.48515809	2.6559623	3.20839132	0.39628997
Ap4s1	32.2250038	33.3083008	33.854653	23.2378711	14.2264184
Rbmx	20.697699	12.4488903	14.6646937	13.6278556	6.45015662
Trim37	10.7471456	6.12238924	6.09527316	6.43624169	4.06972062
Tmco2	0.27209328	2.84865711	1.09180079	0	0
Mbd1	16.327328	25.3340181	18.2827285	9.45548374	10.5610091
Fam26f	2.5915921	1.89296161	1.22779076	1.10665051	0.09659416
Mir378	0	2.56023058	0	0	0
Cdc25a	9.79883397	4.23528631	7.27549963	3.84606058	5.15802666
Mt3	5.8181433	6.37678248	5.94870641	6.12051785	1.35985897
Tep1	2.02113898	2.16410523	1.75318397	1.27616611	0.87703895
Dusp16	13.5990698	17.6363641	9.44734691	5.0057571	12.9850575
Wdr83	5.53087835	6.08003061	5.17028118	5.85157102	1.94171416
Invs	1.79471092	1.38194264	1.13650712	1.52013146	0.45465455
Nkapl	1.0308377	1.6892243	0.49802047	0.53491911	0.43098918
1700066M21Rik	5.66020983	3.83140592	3.9560497	4.59924248	1.52931643
Itpr1	2.54851799	1.78337414	1.76074464	2.07304537	1.07932746
Fmnl2	4.61419236	4.82203742	6.31611844	3.49948199	2.7597929
Brms1l	12.1477738	18.7377748	18.5202576	9.14612579	7.67772955
Kcnk5	1.14372464	3.15081884	8.92132464	0.92545459	1.58450019
Ppp3cc	6.56134043	8.70057824	14.3522827	5.5013824	2.0387912
Zfp420	3.8727998	1.52047287	2.1553128	1.09539813	0.53291928
Slc22a13b‐ps	0.94119964	1.79480082	1.49406141	0.69950961	1.00564141
4921531C22Rik	2.05391148	1.96217147	1.58490767	0.94283138	1.75563045
Zfp951	8.51368596	5.06225405	4.26418234	4.66089327	2.21414566
Cdcp1	10.5212936	14.5804766	10.8878092	7.52844368	5.7022925
Spata2l	1.80839139	3.14112323	0.71041155	1.40870101	1.07588842
Inpp5f	16.536804	19.7904743	21.8099344	14.7906176	9.91127352
Usp2	2.9884974	2.40495896	3.68973443	1.26946893	2.25185949
Dis3l	7.27677039	6.52633084	4.00031493	3.61933256	1.82966847
Slc2a8	1.15834494	2.2123932	1.01463004	1.37962077	0.57701443
Gpihbp1	1.88189317	2.63411427	2.12142973	1.12678406	1.70475957
Ephb3	4.32646915	5.32429133	7.12382538	3.1198734	2.29453647
AI467606	15.0279285	16.4986705	12.939639	8.86497453	6.15755001
H2afz	484.011108	221.88665	277.442807	210.764192	140.77291
Tomm7	105.256018	82.1397691	95.995242	65.3763021	35.2466757
Mrpl39	15.3335594	12.5382103	16.4834681	9.41735659	9.71705869
Setbp1	1.82107983	1.77454455	0.80068485	0.5953902	0.66666947
Tmem205	49.8259915	38.0085048	36.8775798	38.4101121	9.72121119
Usp35	2.70214183	3.75483339	2.24434388	2.02973933	2.69030003
Extl3	6.83970046	8.83103956	5.05060071	4.86729998	3.44420048
Pdgfd	5.63109297	5.59114421	3.83503507	3.13524263	2.27079674
Tacc2	7.20015823	5.23482188	4.8627101	4.66428185	2.40547501
Mast1	1.13941595	1.57088508	1.42313245	0.86362359	0.53085573
Ctgf	47.0762155	280.388949	737.642025	117.546188	76.8850062
Clic5	2.63290808	2.08482149	2.93180918	1.58088891	0.23146395
Vps18	4.52009984	6.78265328	5.64526596	4.34181526	4.91121948
Wdr53	19.7472018	16.7316477	17.3075265	13.7869171	7.94901869
Dnmbp	4.97440618	5.9762462	3.46949234	3.88090325	2.98540608
Adck1	10.1795248	8.5751492	7.89466971	5.4974449	2.67342379
Cetn2	94.2435378	66.9200235	93.2250439	68.9057902	36.3951463
B3gntl1	2.31020016	3.52020479	2.58399432	2.77194411	1.06311168
Ascc1	14.5465873	16.4841208	13.9673754	11.1923884	5.16613453
Trmt61b	6.20009272	6.88523809	6.25379271	2.92741941	2.27129101
Rad51c	3.28928292	2.80810666	1.75799203	1.21604183	0.83144874
Snrpg	402.221719	203.906405	272.577502	210.534488	153.581019
9030624J02Rik	5.17755656	7.01433035	3.7476841	3.78265954	2.38131461
C78339	6.04424059	10.2705846	8.13295196	5.24565165	3.67050793
Ndufa7	252.72912	191.220541	200.448073	185.944149	85.2164855
Mtif2	8.8989751	8.57136874	8.02056712	7.60355083	4.33576994
Cdc123	33.590248	22.9048355	25.5975771	21.2845782	12.9114187
Adra2c	1.77405159	1.31293876	0.11329265	0	0
4930431P03Rik	0.54295447	1.41362425	1.6141472	0.28859234	0.02289984
Dzip3	2.84757334	2.39564116	3.05534825	2.60393658	1.35827684
Nphp1	4.41477367	1.38207137	2.64518284	2.1192847	0.73992832
Pml	7.713341	5.28520725	5.34559261	4.09746769	1.54527294
Hspa2	5.69173269	9.68322941	8.1759599	8.32951612	3.29410928
Fam60a	66.694897	78.8644187	73.6385471	41.7363718	34.765657
Mcm3ap	4.0559362	6.35344702	5.96943814	4.65226652	2.34966076
Rhobtb3	4.0550919	1.92911844	2.97215934	2.3525906	1.32310709
H2afv	85.4209356	56.1498002	44.587444	45.3048921	25.3621061
Mfsd8	7.82283788	6.32715128	6.35318887	4.8272596	2.15812427
Gm14420	3.81706502	2.30552496	3.28854748	1.77144756	1.07562479
Bad	49.9417347	48.8771292	54.9786131	37.3980042	21.3420527
Fasn	6.0565898	11.2064832	19.0244897	4.48812594	3.50731646
Zfp882	2.29643005	3.06267186	2.1798861	1.13361667	0.43463096
Rnf157	1.72310157	1.06699191	0.89507277	0.33439627	0.10103479
Zfp71‐rs1	9.53473946	7.68069173	7.07541204	4.14250586	2.58647924
Zfp583	2.1462075	2.42596894	2.14022721	1.36412186	1.17177618
Fbxo22	73.2468121	64.4564235	56.7940578	63.7718678	28.142445
Maoa	2.42917853	1.53823034	3.29425401	2.37407253	0.67817872
Gtf2b	67.6076462	72.9215834	83.0052349	43.2286683	35.5200315
Stap2	10.6646463	8.16802784	5.54473361	5.45730015	3.25733358
1700037H04Rik	66.1575481	149.900366	268.253349	88.7674607	118.663545
Bscl2	5.22283667	5.51434278	2.89629326	2.38395555	1.72615066
Zfp637	44.6216474	32.3348502	25.4397149	27.2927809	11.6972973
Sat2	1.70053159	3.0769211	2.05391095	1.42547126	0.77023434
Oas1c	2.73854864	3.47187873	4.25283557	1.88566277	0.59435406
Tex15	3.13338756	3.75752204	3.69147012	1.70893795	0.44127951
Zfp81	4.57321214	1.77272956	3.21624405	2.60418983	1.48445451
Bfsp2	1.5783721	1.4676481	0.73077367	0.11440088	0
Nr4a2	4.01362654	2.0500203	10.9692468	1.05536832	1.85797284
Gm14047	2.42908872	1.61389893	0.86511436	0.34309337	0.44920434
Znrd1	33.2712702	31.2004776	38.3787933	19.6365938	16.0733518
Fcgr1	2.51879836	1.24599866	1.02624149	1.13310925	0.08073764
Araf	18.8765379	21.560669	19.1582875	17.5151973	10.4127425
Pnrc2	195.669265	176.651882	181.123281	125.767378	59.1629827
Zfp458	1.75742512	1.68822544	0.79636158	0.7209092	0.62924667
Atp5e	319.757801	194.552787	221.703316	172.378727	105.650907
Capza2	399.708911	280.903445	286.053329	182.503386	168.402619
B3galnt1	11.2859789	8.269235	14.9930097	5.47992843	6.157636
Ncf1	2.03711636	2.14528692	0.88768809	0.94612315	1.36357151
Bicd1	3.99179811	3.29884235	3.02324206	2.25091418	1.09589563
1500012F01Rik	50.6318403	48.1517621	58.3394203	45.3485287	17.6315745
Ssx2ip	31.8290948	31.3964389	18.9132172	8.08744629	19.3383642
Thnsl2	1.83297722	1.74372461	2.08899397	1.55337841	0.28247264
Grhl2	19.5134049	17.951637	14.845964	11.5720829	6.85707179
Slc25a39	82.6979674	66.1328906	73.3530826	66.8731579	32.9156026
Tmx3	56.3866854	52.9036585	46.9879236	29.0195157	22.9724195
Ccdc166	4.83549811	5.9816489	6.9039996	4.56339763	2.27300371
Mx1	3.24414456	3.01203597	1.92825993	1.00621501	1.79168358
Bcl7a	7.56722677	5.64608476	5.53277992	6.6644243	2.22255314
Rasef	13.5254807	16.2317532	26.5400326	11.7591065	10.0219744
2410015M20Rik	121.613323	100.781527	132.2871	90.7959109	43.4671137
Nes	1.17095753	1.65499724	1.08428796	0.86980274	0.65684341
Nhlrc2	16.1288698	16.7514832	20.4148516	10.1659943	4.94147606
Sclt1	7.34171339	4.20488201	3.85904024	3.01331407	1.52273328
Ndrg2	2.7209328	1.99405998	3.91928488	1.16575396	2.64073283
Mpv17	48.9087672	53.0633269	38.0057703	40.2403525	19.6730284
Zfp532	5.20886676	6.38194303	4.12015434	2.60350809	1.38820704
2410018M08Rik	10.3144945	8.88012532	10.108606	5.76826586	4.19569953
Armc10	109.797835	58.9336095	65.0622696	47.8601621	28.5417982
Mterf	9.76279654	7.07040421	7.15316284	5.78683317	3.19013428
Pttg1	13.3950363	14.0238177	22.9657498	14.0201414	8.3888522
Mbd5	1.54363602	1.93786411	0.75320743	1.28140875	1.01477944
Cyth3	8.83788001	9.59116003	8.18373561	5.86548005	3.64473382
Srxn1	18.598782	13.5659444	17.2651229	5.42915973	7.35915552
Gm9776	0.86948919	2.35423501	0.66631704	1.21115903	0.21623767
Tgm4	4.19504183	4.01568511	4.05912781	2.6613605	1.43533259
2700081L22Rik	2.08344056	4.41701122	4.91954327	1.81713429	0.83478335
Gpr146	2.08516771	5.72443786	8.42414837	1.75837383	2.69827604
Qrsl1	6.22324753	4.39267987	4.35262307	3.46454638	2.41392225
Hcst	0.7376888	0.69508522	2.86896974	1.08361616	0.70937698
Col28a1	0.3699954	2.29997073	2.76942944	0.24061146	0.03706201
Fam114a1	25.076316	24.4302775	18.142114	9.58241512	8.3463049
Taf5	5.64447405	4.60495739	5.85686136	2.71968365	2.06912507
Ctxn2	0.37987392	0.49713215	1.81741208	0.11625203	0.05073538
Pigyl	204.883615	193.999527	142.442143	110.64215	59.3662642
Zfp322a	12.0280519	10.486938	13.3260017	10.4333352	6.19174038
Entpd6	14.0074709	9.97465833	7.99497162	8.86072933	3.34447601
Bckdhb	4.10488461	2.41738509	2.72121164	2.569519	0.85974325
Phf11b	1.9625186	1.89320576	2.2845565	1.33844765	0.04493331
Zfp942	13.1817217	8.25527783	15.7461907	10.6577372	7.26019363
Wrn	2.72779588	2.50287307	1.31497924	1.87591392	1.38359815
Ccdc141	1.56615213	0.72814268	0.44842695	0.92704056	0.06605459
Mecr	9.02330783	6.20429848	6.54885744	7.16467229	3.43953217
Cuta	80.3608666	66.802324	75.2489776	60.0540075	31.3544626
Kdm4c	8.83121267	7.20192105	7.35431729	5.90399683	2.51728625
Macrod2	3.99730452	3.63643145	3.91705795	2.11402958	0.73602025
Asah2	1.69187655	1.1903106	0.95252359	1.2426275	0.41215912
1500011B03Rik	28.0871509	32.0948974	24.5498461	29.3668391	10.3072462
Zfp280c	5.8740078	3.8111613	3.64415403	4.17509507	2.10244497
Stoml1	9.89101464	10.9498398	13.5786335	9.74072025	6.17504114
Slc24a5	1.06959204	0.91858606	1.57849005	1.1660954	0.34820435
Kdm6a	23.7932869	24.2456024	21.4587467	10.8140879	9.13140412
Entpd8	1.95126044	0.88819795	0.44516249	0.02596262	0
Slx1b	5.66981374	7.04548826	5.00482246	5.15671563	2.60437934
Zfp708	6.8578732	6.62899489	8.34477352	4.43227395	1.78556165
Smarcd1	5.19128895	5.84638762	3.2940745	3.03811191	2.45293396
Tmem223	163.726675	151.902451	134.978195	117.978709	43.3848514
Vldlr	8.79902551	14.4220981	6.87580561	1.71509517	5.69246282
Rpl22l1	883.29712	593.200594	822.729556	512.972417	378.181708
Thnsl1	10.1856678	4.41260566	7.06220872	3.48562271	3.01562541
Etnk1	85.482653	174.433117	171.093911	76.3028133	54.4422762
Tbl1xr1	7.38215262	8.83337829	9.60720829	6.32806103	3.94165243
Sp100	7.70573718	4.03372751	3.6096676	5.13248919	1.42872513
Psmb1	304.216308	245.69317	317.254302	228.325822	141.084994
Shpk	0.46898015	1.34409804	1.67193226	0.27986599	0.16911792
Tnfaip8l2	4.53343296	1.96238707	3.0138672	1.44071694	0.69862885
Msh6	14.1957419	11.3027303	12.8475671	5.89850175	3.55492773
Scnn1a	3.84036822	5.10875055	2.17510227	2.63577704	0.83659706
Hspe1	307.208194	196.884398	324.018895	194.031138	123.090761
Lpcat3	47.4100972	59.7387135	73.2487692	24.7423524	27.2774635
Ly96	8.43534293	6.02906703	6.40901965	2.28359077	4.67977054
Ttbk2	3.90066372	4.04892695	2.58838714	2.38565291	0.7884065
Oas1g	5.56246223	3.00599074	2.99955505	2.91388354	1.54700894
Tmem17	4.61717802	5.8305981	2.95313416	4.85076423	1.5257646
Mir681	1.18566708	1.86198587	1.09790876	0	0
Zfp930	16.7382474	9.38295452	22.7955323	7.75349786	4.53502463
Rab15	25.4713605	29.1568166	19.7806211	15.0536331	9.829614
Rnf217	34.5667557	40.4086742	26.8565375	15.7419875	31.7381711
Zfp91	29.3563052	31.6228872	18.290976	14.9754536	7.93783864
Tceanc	2.64262839	3.87166077	2.50671534	2.91434376	1.09757406
Gmfg	4.34353288	2.488793	3.26111514	1.40109326	1.12887354
Clcn5	3.62536845	2.11735127	1.1652512	0.70143796	0.51621177
Cldn3	947.092653	1785.39088	1357.58635	1275.94237	687.001184
Cog5	3.26384833	2.30651403	1.69247197	2.01763399	0.9938852
Nab1	34.4642754	41.0603017	38.167866	16.7324775	18.713257
Oat	108.333108	71.4726086	216.293121	81.3451291	25.7528533
Spon1	2.89782727	3.06430452	4.6321921	3.04883473	0.53393101
Znf41‐ps	2.72786524	1.96250701	1.38861654	0.69494065	0.78969847
Ncald	10.2220249	5.71327682	6.91647289	8.36492291	1.47058699
Tmem126b	31.0171609	26.1369247	27.110195	19.7945371	8.54791034
Trps1	3.35582602	2.73037725	1.97692453	1.4495039	0.91176474
Dzip1	1.13639674	1.65877316	3.50761737	1.75222137	0.64212669
Arid1b	8.02118177	10.0193748	8.98443221	8.75447036	3.78376149
Dnalc4	34.1963738	38.1606184	25.9377226	21.4979608	10.5506209
Gimap6	4.16675188	4.06587486	5.50657094	4.15968784	0.8428619
Pop5	34.8806712	20.1919095	20.8203022	20.3399903	12.1528925
Acyp1	78.0930109	90.2402259	126.8271	77.6089906	23.3061721
Atp7a	12.3042434	9.00135081	8.68784667	4.63759382	5.8441598
Irf2bpl	22.1865646	28.2048748	32.4393058	18.2288377	7.10406315
6330403K07Rik	2.32607403	1.34580331	1.246999	0.7867745	0.03270178
Map1s	13.0064854	18.814645	7.28529869	10.0026807	9.20969504
Hist1h2bc	535.309144	320.169494	320.173119	217.521431	140.477755
Vps25	104.448714	117.909776	105.546384	91.1793939	50.9840606
Cep63	10.1107145	11.8894981	7.03296898	7.01737927	4.67138074
Gm8801	2.68950193	8.47467861	9.41190668	2.7898618	5.6260016
Rtca	31.5607856	29.1800639	27.5990335	16.5025711	16.9501689
2410006H16Rik	111.609595	131.621398	140.156032	85.9735419	43.6423287
Cd200	14.392695	7.65778383	8.92781797	9.3108114	5.21015783
Zmat3	1.49998618	1.14489879	1.57519293	1.28479252	0.88640218
Kctd4	2.55223196	1.85117833	2.32326962	0.75457782	0.60663692
Zfp414	12.5885035	10.9875678	10.0558255	9.07719311	4.02646372
Sdhaf1	31.7628808	21.5514756	24.8533845	19.3787437	9.45713712
Zfp563	2.92236653	2.24652009	2.14782058	1.73549227	0.62230731
Aldh1l1	1.9453552	2.25805031	1.80136782	0.40767369	0.44056206
Sec61g	41.9397387	25.2407841	37.1301751	20.2127799	26.0280948
Anapc10	26.8573482	16.3737801	20.9688035	10.619303	8.54172251
Hdac2	114.966517	75.5038059	70.7203358	61.4830507	41.4095864
Ccdc132	5.4398697	4.21900842	6.56179351	4.39167706	4.10963473
Klk11	6.57067403	5.61872549	3.87564067	3.22280458	3.39006152
Rassf9	4.46444522	1.93407409	1.93870575	3.02458349	0.19738409
Fermt1	39.9832662	28.8421789	21.2927444	12.3727107	13.4347856
Ncoa4	6.45371487	9.39061369	4.77745315	3.91655899	2.951077
Zfp512	10.9971791	12.8749236	7.6923544	6.53719302	8.43219802
Cenpq	33.2841911	19.1395878	24.7905058	12.8707599	8.37133711
Kiss1	0.39442554	1.54852656	0.36523175	0	0.52678869
Zmat5	32.7298214	26.9327171	24.6438076	23.674747	10.729664
Rcbtb1	16.6913856	10.8758962	9.08172802	9.37239134	4.86022288
Tvp23b	96.9221793	110.142643	104.050095	81.0023226	45.3515635
Ddx60	2.2539355	1.05162251	1.15950458	0.45984479	0.06980456
Zbtb12	4.44221318	3.71128792	4.90319473	3.26265901	0.65499786
Tmem246	33.6879697	55.6504695	31.1945214	27.41607	22.5987578
Sarnp	87.931393	72.756835	85.5738211	58.6859268	37.839896
2500004C02Rik	4.1573311	4.42442376	2.73609508	2.996644	1.34911193
Mrps22	14.3186851	8.04282961	13.3085322	7.43448907	7.43465191
Ccrl2	2.34885706	1.16484424	0.77269882	0.22699448	0.42103149
9330020H09Rik	2.51106856	1.64308847	2.62466812	1.64170377	0.94514619
Hspa1b	34.2535423	20.7551788	26.7542002	15.8942582	13.1666427
Acot1	7.32480467	4.76551263	4.92226747	5.6488187	1.30811301
Lgr4	19.5377315	19.9455117	23.4379654	11.5835034	8.34466594
Ptprz1	1.72971016	1.25663276	0.59127585	0.23746076	0.22669935
Use1	63.709116	100.946234	117.820283	73.8302145	34.0516207
1110020A21Rik	2.2984448	1.88869469	1.8808437	1.27591362	0.77101138
Ttc8	10.962797	9.53985397	5.88675085	5.65494577	2.9434432
Trim24	11.4614485	13.0975588	11.4858148	5.35897446	3.49194999
Zfp740	35.1287602	34.6162367	27.0964471	23.5952704	14.7188285
Polr3gl	4.27922642	4.01748022	2.06738883	1.75082846	1.41639275
Pnpt1	13.4330292	10.5666321	17.3740814	9.38786692	5.56588094
A430035B10Rik	2.2568095	1.26953582	1.15405183	0.6184896	0.43187965
Rlf	19.2812695	25.4040191	21.3204379	11.0374986	10.2134127
Grcc10	196.670175	152.259215	133.123858	142.969488	43.225288
B930003M22Rik	3.19926523	2.88889468	2.96246846	2.39870797	0.98276457
Zfyve16	8.05126895	14.6070759	14.0909369	11.2907993	6.48065087
Zfp157	5.39742302	6.92189036	6.63733871	5.64383416	1.85226212
Cbx3	37.3258256	23.5717291	23.2095355	26.0664378	11.709376
Nipsnap3b	133.885181	199.144146	234.688016	156.487799	139.850197
Zfp551	1.5081076	1.51311272	1.49992675	0.58972387	0.13428035
Ttc23	1.34402755	2.15206288	1.39096544	0.82261991	0.31677574
1500032L24Rik	203.772596	143.022795	125.002074	90.9816167	88.8823086
Gimap7	1.38748276	1.53331382	1.18961746	1.27382538	0.37062013
Zfp189	1.54695288	1.47129856	1.19034879	0.46007525	0.33173783
Prdm2	5.29079168	6.72589797	5.7148485	3.88280152	3.71489794
Caps2	1.77205678	1.57695362	0.45945095	0.2819954	0
Zfp629	8.6579287	9.18434579	7.74065172	5.18819329	2.30698303
St8sia5	0.43412442	1.4609019	3.84764032	0	0.74546987
Fbxl8	5.17988697	3.36695236	2.50942324	1.60618592	1.81430347
BC051226	4.11454708	4.44992457	2.22849339	1.35419769	0.68432359
Dcp2	8.54851837	8.0334899	5.65214052	4.64799618	2.56964145
Nmd3	19.4734562	12.2855714	19.6303308	10.1964664	10.9145281
Galnt10	11.1391165	13.8658733	4.86830636	3.33671255	3.83510232
Tlcd2	3.83261684	4.35688361	4.60832758	4.46397578	1.28351601
Tram1l1	1.72998637	0.80582797	0.97745475	1.04987496	0.32895869
Ngrn	39.4762265	38.3289467	39.7528607	40.9121867	16.5775923
Ptger1	3.25745231	4.50069352	2.75531859	2.53051958	1.85737291
Nudt16l1	51.9021327	68.0307132	55.816851	48.641258	26.2316689
Ssbp1	19.1403042	10.9256644	12.8845045	12.5391011	6.83406019
Kctd2	20.9002357	20.267284	22.225503	20.3069116	8.93643612
Ly9	1.65092885	0.87815177	0.64108188	0.53782582	0
Zfp704	14.1596604	12.8751266	11.7943507	9.15085824	5.6847516
Lsm3	305.285786	171.709918	267.856679	182.835326	108.241634
Prmt10	6.07329291	6.48113026	8.16424065	5.53273526	3.28840403
Ccl11	1.8232532	0.42948882	1.68830332	0.33477983	0.09740436
Cox6c	585.608751	372.78586	512.972223	420.278804	201.653452
Plp1	1.69247798	0.68460747	0.45116581	0.98880507	0.10274762
Rsph3b	13.7372828	11.2942018	11.5981068	6.34308214	3.20538187
Pdhb	90.681575	102.960923	71.3234064	57.5717603	40.5742934
Zkscan5	3.82992014	3.97823556	2.05153086	1.34025083	1.04597452
Trpv4	2.95130399	1.28567066	0.84231996	1.03927725	0.097193
Clec11a	2.20039443	1.93718878	1.26573838	0.55094901	0.66791204
Zfp386	80.7488088	62.4621871	62.7535939	43.0250714	23.281764
Cep70	7.25698135	3.95103439	4.16771105	3.66786633	2.37073292
Znf512b	10.4639755	13.2491456	10.487563	8.89353958	3.16151661
Piwil2	3.34486989	2.08445396	2.50733489	0.15841793	0.94665417
Gm6548	18.2209133	13.5039613	18.9739086	12.3261338	6.37848137
Dock9	10.7071635	7.64093123	8.14103179	2.97125575	3.0521731
Slc46a3	2.02018865	2.05156849	2.89329117	0.5687754	0.32377595
Fam110a	11.8482777	10.1490971	10.885825	5.17044459	9.44530375
H2‐T23	33.9675163	20.1397029	20.8619338	19.1383316	12.2211775
Plcd3	8.9717171	4.6119627	4.92084809	3.04188324	1.91374978
Gm14322	1.73573403	3.1079416	5.13722982	1.31058253	1.55992351
Qpct	2.98265524	2.68797597	2.16557368	2.33380169	0.81482491
Pkd2l1	1.099625	0.81717688	2.0001048	0.48674628	0.18882543
Itga6	17.446647	10.0217349	14.0156344	5.51001422	11.7066602
Zfp105	8.36297615	5.97898159	10.0340315	7.2596605	4.06794425
Hsh2d	3.72638226	2.25718288	4.43644766	2.59028491	0.81052352
Ap5s1	17.2592391	18.4150663	13.4516825	10.075852	6.2037461
Btbd2	17.1326151	22.7425638	15.4255093	17.1537047	7.54676149
Kdelr1	53.5377281	98.9172364	72.7391554	53.3135597	53.0113155
Fam105a	2.78446262	2.03616773	1.88949096	2.36122716	1.02198379
Aasdh	2.33024987	1.38615624	1.78179833	1.49107226	0.84879433
Dhcr7	15.2839897	17.7652363	15.8467691	8.22784439	16.9428411
Tubb2a	47.7000147	57.3619262	41.6863303	8.76598212	30.0846557
Oas1a	15.5085003	6.23784992	8.82746559	6.15187489	2.49108548
4933403G14Rik	2.42613952	3.70901498	3.06349965	1.06307693	2.577523
Triqk	10.948265	9.17657595	6.87930175	6.66106289	3.09854228
1700037C18Rik	6.83622999	5.18114119	7.59628762	4.36607422	2.62001739
Batf2	3.42468391	3.80953346	5.37342656	4.10485815	0.91479103
Uba7	5.50648991	3.2549421	2.99824231	2.95372792	1.04118235
Caap1	34.9094346	33.1652439	27.3436026	14.0235551	11.2989055
Pik3r1	6.47786535	8.72955703	12.5614511	7.68351996	7.23771532
Slc37a1	2.51991072	3.36370344	1.18336564	0.4461729	1.35583747
Caskin2	5.34454126	8.54494576	5.86969648	2.07934054	2.50109719
Paqr3	2.13420075	2.53230079	0.61482891	0.56604168	1.59622719
Tcerg1	14.1693805	17.2462799	18.8180915	10.8088807	10.9635132
Socs7	13.9905335	26.5548648	14.5216628	14.9439782	7.65796806
Bola3	97.5714525	48.3062373	70.9808374	54.6735466	32.439051
Oxsr1	18.3685283	26.1974588	18.551784	9.66693408	11.1956772
Cyp2a5	2.24343422	2.56769481	0.10562971	0.3141855	0
Bnipl	10.57684	14.8917215	10.4356597	5.15657052	4.35478648
Rnf114	68.1017777	84.4082683	78.3334672	66.632825	40.1203387
Gap43	1.65410127	1.53495761	3.20258552	0.46018288	2.04852369
Rps27a	338.687752	321.260602	439.243743	322.903097	171.79394
Map2	2.07921329	2.64371636	4.78047775	1.75704827	0.51121406
Cntrob	2.22875395	1.46484079	1.46758437	0.86394385	0.47627032
Zmym6	3.23376787	3.92643006	4.08993589	2.92542287	1.3399343
1110018G07Rik	11.0786655	12.3464092	6.15742316	6.07183063	6.15585498
Thoc7	104.520478	56.6908199	102.991111	62.2687566	48.024039
Acot8	11.3313101	9.83164131	12.9059128	8.47851974	6.49245316
Atp2c2	2.67078592	4.73440094	4.70264676	0.92878984	1.07011818
Hexim1	12.4285808	18.7649841	12.4677228	10.8646065	7.43899995
Spats2	9.82294643	14.6796505	5.57490116	7.76736379	8.02397974
Cnp	100.350027	114.97137	190.535138	64.2963122	114.38876
Fsd1l	3.76671458	4.86257216	4.45349917	3.06089444	1.26763936
Sub1	133.565165	115.777003	172.51813	118.171181	71.5805734
Tank	18.3643721	14.9685148	15.2450726	9.3923464	15.6346727
Gm7008	1.46360401	1.53230758	1.05410193	0.29860246	0.1303178
Lage3	81.7720265	45.343031	56.7301016	55.2220062	23.1032882
Gm11545	76.6655065	148.499078	181.760366	12.8721166	97.3544615
Slmap	29.9617361	27.1551672	25.7182872	18.6619239	11.935269
Exoc4	4.3240096	6.68006253	4.94798775	4.80894832	3.39462062
Abhd14b	41.4702134	102.964663	71.2338093	64.5376071	22.9578438
5730405O15Rik	3.01429456	1.32774077	2.51887749	1.28244201	0.5302333
Elmod3	4.28909099	3.02416498	1.86423435	2.38407676	0.67806071
Il1r1	14.4411189	12.1324929	9.2826169	5.81456379	6.90192573
Dtx3l	11.4916565	8.64775817	11.6201172	7.49961311	2.18874039
Ccdc102a	11.0950444	7.37844229	5.60867455	4.31353542	1.8825379
Etv6	19.5752992	17.0743768	19.076413	11.6058622	7.55984632
Pycrl	35.8110017	30.1248121	26.822893	24.7355611	13.093327
Nabp2	40.5403469	35.191002	24.2802732	27.3739262	13.3624057
Phf14	6.0957291	4.53552967	4.23535717	3.1571358	2.2369094
Anxa5	98.2527835	96.6735933	70.2680725	40.3417466	42.8856744
Ybey	1.96125382	2.04954327	1.49560327	0.96649599	0.87827626
2900002K06Rik	1.78555817	0.73149445	2.30038027	1.14037701	0.68432359
Ttll10	0.5370707	1.06537553	1.57048068	0.70068678	0.81546067
Ipp	5.97311982	3.84397225	4.96080589	4.97501583	2.195898
Dnajc22	0.31223107	2.34658616	0.49563597	0	0
Tmem29	38.4448137	28.6619348	38.0778528	15.3969536	12.5471
Msra	14.8805198	10.8119185	10.6616747	9.85981616	5.20685879
Aga	16.2776465	15.9170924	18.4431651	13.8303862	4.90166664
Wdr6	13.5459277	19.3581462	9.62145853	11.2658212	4.20153886
Cln8	9.1667497	9.62624403	7.02347482	5.99535788	4.94865177
Blvrb	12.5900025	7.75468683	6.07340282	5.74473156	3.4435537
Dleu7	1.46873175	2.19118833	2.49337388	0	0.15692924
Elmo3	5.79659463	12.4832158	11.4455195	8.58916308	6.48948573
C920006O11Rik	2.97441017	5.8741987	4.5069648	4.01338625	0.65005797
Zfp758	12.520469	10.1582787	13.218098	6.90495593	5.72828672
Aplp2	123.785698	112.375686	59.3714821	61.6039747	33.3859104
Ufl1	9.7629496	10.53622	7.80059745	7.98263904	5.43670228
Gm20748	7.05845428	3.72246772	4.2923089	1.45080273	1.18191297
Zkscan8	3.5394141	4.13710706	5.2588337	2.66416878	0.94543741
Sh2d1a	2.14983592	0.43764625	1.76952328	0.43860654	0
Gm14403	4.16356962	2.30365575	7.9385429	2.11286176	1.30925154
Gm4285	1.38748276	2.70486576	3.18981563	2.19625065	1.07352036
Cdkn2d	32.5031492	21.4494908	26.5789015	9.42831382	9.17591229
Parp12	17.3682419	11.3858504	16.5502351	11.2545569	4.85513512
Timp3	23.1117695	28.4409293	13.1322178	9.09366004	15.6515547
Mapk11	13.4589236	21.9747882	20.0294167	3.92267275	23.4537886
Jtb	163.886495	138.433501	193.199095	145.359297	74.5722638
Tia1	28.1328145	18.8919089	20.3932328	15.9857555	8.70763015
Matn2	2.55804808	3.27488277	4.11949849	2.73991945	0.40106618
Prosc	16.4754401	27.0585216	22.0453109	15.9705576	13.4445392
Rel	2.04417448	3.58670073	3.17815695	0.85726871	0.56625707
Hes6	53.9767168	47.8676818	26.5512992	33.0965596	16.9885603
Ndufa13	123.322667	77.9753172	91.1334597	86.0897901	46.1907216
Kbtbd11	0.96339702	2.90835143	2.11330115	0.24044094	1.03435548
Mettl15	9.62139682	5.03651917	5.30084983	5.8255113	2.84638608
Zfp68	25.1005045	22.8045057	25.2338004	15.4008746	9.34470351
Zfp788	6.17902462	5.57356382	5.46948284	4.50078317	1.41863052
Man2b2	2.31678105	1.69986189	0.61545555	1.20297487	0.35000614
Tmem143	1.56339689	2.26631752	1.97664072	1.35252183	0.21683584
Zfp748	11.5310954	11.2689288	15.6823601	9.20147288	7.6110209
Vmac	15.7298847	15.6808498	10.4002254	11.4689058	4.52316135
Mnat1	21.9194581	16.6389436	13.7402953	11.9978587	6.32095953
Lcmt1	23.4838298	22.1799377	16.7172448	22.1286451	7.55805162
Chchd7	10.4151044	15.3245456	8.42783203	9.64808171	3.3835751
Prpf40b	6.39937441	5.83528337	4.14800288	4.73803362	2.43173262
Hexdc	1.09645548	2.04474069	1.6752454	1.35896125	0.68095022
Lipt2	40.9098557	28.5395923	26.7777178	25.4288298	7.78121326
Rrm2b	6.2436724	6.16225711	6.04920476	5.3606818	1.93417378
Osbpl6	1.15971541	2.53444042	0.70590577	0.73711099	0.30544703
Prkra	24.7586209	25.0751026	23.9752471	25.8896401	11.6351824
Scp2	89.0296227	84.1493203	86.4067386	67.5782533	24.4417295
Bcl2	1.60985301	0.91330443	1.77166743	1.42381309	0.22964365
Ndufb6	126.393443	74.8891416	135.92275	81.7323576	61.847754
Nme7	28.8361325	36.285527	24.9564511	10.7407602	15.468896
Fopnl	151.05999	110.655254	127.267082	92.3308408	57.5125809
Fn3k	0.91278409	1.91517937	0.69280613	0.57699204	0.05995576
Kif14	1.87381366	1.06846567	0.44755118	0.23210638	0.04766927
Ofd1	2.94353236	1.25338034	1.74930669	1.66715794	1.09138597
4921530L18Rik	1.46726302	3.13628246	1.73606823	1.19739586	0.71853977
Amdhd2	8.55819849	11.4449132	11.5569344	8.22544658	5.25074733
4933424G05Rik	0.71004132	1.05637021	2.07627445	0.9263521	0.11978782
Zfp516	4.89692488	9.45225207	6.00991634	5.59101301	3.42945702
Pias3	9.16545556	8.4872264	9.18944315	5.84189901	3.91719727
Fam120aos	47.0452572	49.0667051	49.3495336	25.5296926	14.6292211
Phf1	4.40293005	6.70360908	6.36416443	5.15141486	2.58167663
Dpysl2	20.0255211	9.13872918	9.27846859	13.4139676	3.87921864
Tmem51	50.3876875	63.8828078	27.2280202	17.3155964	20.5516177
Txlng	1.86710643	2.64967388	2.50612744	2.39038836	1.33148712
Akip1	41.1510559	40.626886	35.1883708	12.9830691	16.5419227
Arl6ip5	70.6175876	63.82412	61.2869235	41.4930304	23.2211915
Usp46	12.8199104	12.2255972	14.2302593	10.6187664	3.11114048
Tex35	0.43571285	3.70634716	2.62251036	0.1333403	0.11638628
Cyth4	3.17763876	2.29329145	2.01211155	1.90914065	1.23575453
Uqcrh	601.356881	592.416724	757.355965	343.209618	204.5314
Zfp827	3.69624384	4.71373331	3.01766086	2.03135278	0.89340701
Tex14	2.90837732	1.74942106	0.95123642	0.01224832	0.2138193
Nicn1	8.34139063	5.98969778	2.58423604	3.53012568	1.36669476
Sirt4	5.36781427	5.04072094	2.35091699	3.1966074	1.07537553
E130307A14Rik	2.35138304	1.56937211	2.61281172	2.15876657	0.91073659
Krt8	653.923313	567.420853	477.459537	266.113766	407.984383
Pex10	9.81936794	9.23361847	6.85609693	6.61584838	2.82386883
1110001A16Rik	40.170056	25.2807963	29.2545881	27.3430448	10.8452316
Cmtm8	70.1761133	99.1367479	76.9000476	92.8431937	26.2269471
Efcab12	0.89471154	3.13128945	1.58596386	1.73671684	0.92182858
Pex7	43.8302433	56.8008917	44.7626794	43.0847558	25.3837607
Sh3rf2	1.55395749	2.49387009	1.1360051	0.97613793	0.41508834
A230072C01Rik	5.06642386	7.32823836	5.98088418	4.05297552	2.68291252
Aco1	17.5447982	18.5304024	26.1309404	19.087915	9.19556672
Msi2	13.3215843	15.0107081	9.67895371	5.12743203	4.42184214
Rnf182	1.66048284	4.00036028	1.15318542	0.13858748	0.42338202
Rab20	164.022754	208.293551	168.860497	118.74452	62.0285652
Ccr5	2.0726887	2.46748128	1.87800183	1.75967265	0.42863244
Phkg2	3.68971429	4.5662169	3.97367348	2.20921745	1.67120957
Mmachc	7.50103372	6.20822751	5.56918487	6.2041236	2.89717401
Zpbp2	1.90979933	2.06193067	1.17896243	0.3287542	0.31883733
Ccdc130	7.26990727	8.85529966	8.77437805	5.47641744	3.60997068
Cdc42ep5	72.6083858	68.2135508	54.9335601	51.762425	31.7536514
Nrep	41.4021477	23.6185166	24.4724532	25.4537699	15.4970333
Gm14295	1.8901939	2.03099908	2.71755449	1.37001814	1.31540461
2610301B20Rik	17.8577809	10.8791668	9.06630042	9.52602463	3.79964372
Tbcd	1.88367099	4.20160539	1.77512089	1.48450151	0.4274637
Fuca2	59.2248804	36.2213704	29.7496067	22.2884004	14.0886264
Tcf3	29.4895828	26.3168894	19.4757315	16.4907774	10.8875801
Rab27a	6.07998586	6.44581781	5.10152491	4.29224701	1.89963086
Coro1a	3.3483773	4.48882005	4.65081577	4.64862511	1.07983434
Aifm1	18.9424432	13.0206012	11.3293728	10.5484873	6.66904443
Suco	62.627206	47.6491867	40.3109003	22.7596327	20.3794066
Eltd1	1.33409204	2.36942917	1.91184795	1.00609248	0.52181075
Nop10	357.209931	235.24651	424.780111	298.401384	138.049864
D10Wsu102e	38.6363929	21.6404785	22.9562742	14.4854529	15.1157878
L3hypdh	7.07575517	4.11438814	4.00205453	3.89768152	0.88883618
Setdb2	3.43898165	1.83958558	1.67183207	1.95356278	1.20567589
Jazf1	14.5873792	16.7266194	12.4161503	5.48838643	10.3232898
Ep400	7.8328914	9.74964635	11.8443593	5.25066621	6.05911391
Cdpf1	25.6651493	22.4777103	24.5653191	24.0158866	7.41590885
Arl6	16.3901038	9.17301864	10.6965376	7.00348512	2.93424115
Nudcd1	24.3858044	13.7704308	15.1181432	13.5301911	8.11074243
Mirlet7b	2.30158904	1.20481439	2.13123466	0	0.61479339
Hist1h3e	3.49065005	1.49502515	2.05690936	0.29133719	0.7628823
Khk	2.16010073	3.34640702	3.189538	2.35834457	0.42105366
Zfp119b	25.8165413	27.505271	17.825825	9.90991826	8.15403351
Ddx51	6.24395872	6.80473996	10.4421409	4.39819361	4.1948294
Ppp1cb	234.933863	169.596189	171.64101	108.298266	116.826021
Gapdhs	1.08933726	1.2052718	1.65061454	1.18193972	0.87294125
Prdx3	130.37825	164.24455	226.45413	102.213688	109.469387
Mx2	1.02229546	1.94340935	2.11746018	0.46927643	0.34493358
Naip6	4.12375188	3.49764535	7.6460626	1.2143612	1.13010119
Crebl2	1.56857771	4.21993841	2.73827797	1.25122201	2.51396191
Mars2	2.28535067	0.62184643	2.87048666	1.08023221	1.03354405
Hdac7	5.94267816	3.94358097	3.29926011	3.24697062	2.0860832
Gm3219	7.98951163	11.0208601	6.59835896	4.95610867	3.89335215
Ltb	2.02483785	1.21581726	0.93748374	0.5467561	0.09544738
Pkia	2.55037569	1.16816604	3.0504092	1.21950963	0.14192677
Mycbp	66.7486654	45.2738417	45.0955859	35.5590284	24.1404729
Gm10767	1.53950115	0.86787477	1.53521141	0.90601987	0.25306265
Enox2	4.52562894	4.58356593	3.68960288	3.08607179	1.83958795
Ddrgk1	25.8832955	24.0908163	22.3221505	18.488401	11.0590218
Prelid2	39.0909852	37.2011404	28.5243976	6.45006055	45.6891686
Gm4944	7.46994558	6.03333025	6.92942417	4.58838166	2.66641434
Lyrm7	2.18369419	1.73813819	2.32676078	0.7140434	0.83899556
Usp25	15.9445758	15.0028468	16.9537713	10.3920828	7.54316515
Cbr3	9.16428199	8.46828623	11.4871174	7.38753227	1.61205463
A330069E16Rik	4.38643652	3.38685211	3.24941607	2.01355805	0.99593771
Ssbp3	33.7182194	21.8601221	14.9875714	15.9465395	8.37427484
Dcps	26.7073	23.9726783	32.3165859	28.8819101	6.95890502
2700046G09Rik	1.62929876	4.18124189	6.88116492	2.73627938	1.27379495
Guf1	5.27857316	3.26947494	4.88787436	4.46991666	1.55012007
2810410L24Rik	1.83372062	4.13956438	2.26399464	0.66638902	1.07147646
Psmb8	118.906024	52.5025207	75.3454477	57.6668977	34.0549287
D730005E14Rik	4.93562275	9.42607764	11.7187379	3.85078789	5.28804124
Rabep1	16.3059286	14.8618954	14.9320046	11.4705356	4.5001334
Rars2	4.99478929	5.89662104	7.18021747	5.22698244	2.85498588
Camk1	10.7321888	7.14150788	5.26368393	6.47128165	4.22724043
Zfp110	27.8743683	31.539386	30.5412913	20.5807684	8.93780352
Klrk1	1.22779373	1.69489908	1.9070872	1.19994119	0.20630024
1500011K16Rik	58.1356986	34.7256922	64.8524283	44.4409559	14.6269277
Gamt	3.86849006	2.76635084	3.83802852	2.66369841	1.05179165
Oxld1	14.4914866	22.2518806	14.8353228	17.8131112	6.25798945
2310039H08Rik	67.9956297	69.8846897	92.6475921	57.3139025	25.2836762
Rps27	5.44375843	6.32265638	12.6020832	5.62255445	2.72647501
Hemgn	1.46796877	2.03931786	1.12404945	0.07775298	0.09048907
Gm16845	0.94509695	2.19255583	1.39227561	1.2226374	0.92948012
Rnpep	55.3208249	50.2032176	32.9348772	21.127454	21.5525026
Zfp251	7.03482267	10.2292318	10.6156247	5.87762806	4.07142708
Arxes2	3.00651296	4.58846519	5.76402101	4.04315484	2.00207067
Casp1	35.4289549	39.1564676	41.2677273	35.5340872	8.84197227
Ly75	0.15130322	0.95043363	2.21831914	0.72927242	0.24249391
Ccser2	11.1828534	10.5465201	7.03957549	5.2723531	3.97821873
Dnajc1	7.71174005	10.5654628	5.80421883	3.56442551	3.42781515
Wbp2	115.891526	223.303454	166.622182	137.250249	90.7736439
4931406C07Rik	5.381441	6.87079658	5.36800411	4.29793212	1.49006448
Islr	10.2178826	8.39277285	6.92311896	8.00806146	2.01928953
Pacsin3	46.7035783	78.4959161	77.0646496	35.2626544	36.1610556
Zfp54	36.6430038	33.7191251	41.9313696	13.7162262	12.4873846
Fkbp5	10.7166714	25.5219562	26.3247511	9.22387678	7.2906953
Dguok	26.3784222	21.4044502	22.8483716	20.9274591	10.4985663
Fbxw5	25.4875701	21.445594	14.8722656	17.3594482	7.88624325
Gm10012	63.6339874	54.9210753	60.2226568	33.958404	37.6478315
Iqsec2	1.38860187	2.05677501	0.95462627	1.0044295	0.89357774
Hspb11	33.5974358	20.7932859	26.1416201	18.1001699	10.4701843
Specc1l	9.62272523	11.866617	7.99130238	6.1841274	3.44162063
Pfdn5	121.286048	124.159855	137.959199	105.611021	63.664666
Apobec3	35.881015	41.7924585	47.6891562	22.8133371	20.3801882
Acn9	41.9726147	26.1609015	30.4120728	19.4848962	14.9171231
Ehf	74.2643266	76.614331	47.102213	51.7088317	19.3925481
Ly6g6f	0.12211927	1.63010936	0.56540245	0	0
Zfp94	2.59866131	2.29073262	1.24856166	2.02566968	0.19645653
2310043L19Rik	1.68167681	0.14671809	2.07627445	0.25732003	0.74867389
Ppp1r35	19.9315814	22.5758351	22.1861755	14.345797	5.56522233
Noc4l	18.2938138	19.4873853	27.4531037	11.6862077	15.0441961
Vsnl1	0.60630703	1.50757379	0.37428708	0.12369792	0.10796991
6330418K02Rik	5.80832862	2.62587751	4.72648982	2.18149043	2.04516288
2700054A10Rik	1.34982798	2.07267977	2.20819047	0.37177654	0.02403746
Fbxo4	9.87826522	6.77576984	10.8643903	5.71594946	5.7777376
Dffa	7.85091823	8.49557325	9.85969985	5.38332599	5.54424283
Zfp58	11.4721709	11.1786244	11.6725527	8.1975172	5.13934953
Pard6a	9.94831933	9.31644437	8.9584438	6.99667977	2.0478772
Nme6	47.3341434	28.3748481	37.6448815	19.8008388	23.605067
Mlh3	3.18369523	3.94317016	4.23851845	3.93764112	1.01087527
5730508B09Rik	65.3744137	57.8168103	43.3204799	17.8899755	23.4718373
Cutc	13.5608406	11.7833242	18.8818484	5.67572959	5.19378203
Lace1	4.48930453	4.60495114	3.36406585	3.54978079	1.85074093
Dact2	3.85373459	2.35151117	1.91323012	1.79033912	0.57671078
Ifi35	31.0235023	24.8869472	46.9315633	19.1055074	11.6042251
Hist1h2ai	1.82526358	1.82408285	0.61460542	0.7617022	0.39891174
Rwdd3	2.96067223	4.22679576	4.20131361	1.9415322	0.52380686
Klrg1	0.04780916	1.53914155	1.77082059	0.52671372	0.15324762
C2cd4b	4.02057033	1.34748978	3.40516816	1.80059527	0.82511744
Ccl2	0.56635462	2.09646673	0.14983867	0.29712056	0.71319084
Tmem9b	175.897406	171.239161	93.6056814	53.6518618	67.5780159
Acad8	12.6142724	14.3029641	10.0360515	13.1905863	5.32792312
Serpine3	2.04658655	2.00874011	0.86141201	0.68325013	0.82001685
Rmi1	10.1425455	10.0111157	11.4789094	7.98366852	4.56932458
2610306M01Rik	15.1992429	17.0351899	9.22710557	7.29554535	4.24528507
H2‐Ke6	10.620576	8.92362525	10.4608838	10.8063734	2.71044801
Rn45s	13341.6543	23947.0353	21736.3678	13047.304	13675.5107
Tmem134	71.6167929	54.4449825	45.0200492	47.2407	26.6282131
Glrx5	172.986278	189.346945	183.75215	193.707749	90.1300323
St6galnac1	0.08178724	2.05503458	1.81760816	0.10011671	0
3000002C10Rik	3.21466075	4.72781233	5.05570663	3.3260996	2.28984078
Mtmr4	14.3209086	11.6676706	8.29603935	6.63700619	7.55584526
2810021J22Rik	2.61273327	2.08293493	2.99949931	2.57004425	0.99344679
Hist1h2bh	5.47709728	3.89746387	3.16981533	2.04280133	1.09726903
Ubl3	102.05984	93.4239369	100.800128	47.7925717	35.0734209
Itgb2	1.54940422	1.7175551	1.49802017	1.06686214	0.4929947
Morn2	3.62847113	2.21596464	1.30663021	2.40589585	0.56538186
Cmc1	43.7684544	31.250828	35.6797147	19.8891197	10.469157
Mir429	2.35704902	6.16923031	1.45505981	2.16396841	0.62960768
Rdh9	5.53501739	9.84996048	8.16920624	2.99570868	2.42111924
Apip	17.2204029	11.9144929	15.3576524	9.07118072	3.50645145
Xpnpep1	59.9864768	64.7725134	41.7018063	28.2368836	32.2126371
Trim21	7.35540859	7.89750405	12.8821295	8.00421374	3.7286388
Endod1	19.3009095	28.6608363	33.9614865	21.2557855	14.8238101
Tmem64	31.7072859	31.7882079	20.0724273	17.1129636	15.8859931
Pebp1	230.778763	227.778213	221.411601	173.987088	99.3421849
Usp11	1.95785673	1.43384738	1.15052218	1.45180791	0.42240423
Rcbtb2	11.7367512	9.37511819	10.2573037	9.19162794	2.41229873
Sox7	12.2197042	12.0250879	35.6282487	16.516437	5.15214175
Oxa1l	19.7899721	25.0163019	21.8758538	19.0902891	8.10464602
Mll3	3.31873997	4.64251086	4.18602194	3.96877639	2.14684242
Rac3	59.7672999	82.6968726	81.502921	39.154178	50.9001017
Ncapg2	8.33414082	2.88433003	8.90596192	2.62904905	1.05242871
Eny2	54.123823	47.0517468	45.891592	26.9401744	17.9426526
Zbtb46	0.54766866	2.0887304	0.61580366	0.48484835	0.40752651
Cox7a2	211.029871	144.649632	203.979508	133.952706	93.6336848
Fam69b	11.8201213	5.52454193	10.6846423	9.26468433	1.67533093
Tubd1	4.50496213	4.45257492	3.85063654	1.53289808	1.08554098
Rpl18a	458.300278	594.14801	672.513766	443.713538	254.72971
Nuak2	1.04105507	0.93188469	1.56168057	0.49700339	0.73414025
Srpk2	5.96794362	5.24298306	4.55045312	5.60000237	2.28159416
2810403D21Rik	5.25586752	3.92313939	6.60930152	3.27645632	0.93595411
1700010I14Rik	3.43767692	1.88131959	2.73307959	2.29532752	1.1965228
Fez1	2.23292945	1.95254939	1.59773493	0.27954767	0.04066727
Eif3e	95.8497578	86.1938756	95.0642222	64.376539	40.3884544
3830406C13Rik	53.1803039	52.3829171	56.362567	38.0681969	17.1941105
Lyrm9	2.29718598	2.33070664	1.12333036	1.03080783	0.53777692
Ablim1	8.40846329	5.8784313	6.12984831	3.29271164	2.5267982
Pde4dip	2.90744562	2.17856143	2.19191886	1.39369413	0.97593952
Tonsl	3.13115315	3.71224305	3.08599266	2.54734797	0.84466503
Ptpn6	4.84352383	5.9490835	4.05907348	3.27917537	1.84310465
Nr2f6	112.606299	116.953364	103.618233	79.7904001	47.0976636
Gtl3	59.2146331	52.1938815	44.7520848	40.5300916	23.6248849
Lrrk1	5.09165593	4.00486833	2.26828481	2.85132499	1.85782412
Enthd2	3.98920015	4.51426046	3.36800008	3.89939996	2.48218128
Nrarp	1.01299712	1.53116702	2.79060694	0.39525688	1.8061794
Rspo3	0.46268728	0.83346404	2.97388476	0.07496218	0.26172339
Amn1	26.7323479	26.8736958	24.518537	14.3407683	12.1102923
Mkrn2	28.3263276	26.5623922	24.1809504	19.3774999	10.5448044
Zufsp	6.45496419	9.96367229	8.37828854	7.31910749	3.49267139
Tarsl2	5.30840303	3.67121354	2.45774817	3.09283526	1.07983434
Phactr2	11.7648824	12.0643649	5.40639691	5.31325099	2.81426793
Mir3101	3.70520963	16.2923764	10.2928947	2.72135422	4.75067615
Fam185a	4.29525405	2.525341	4.05570775	3.7102954	0.98343004
Gpr85	1.61316616	3.19269689	0.16370039	0.24345561	0.08854191
Fam69c	0.66635261	0.1902633	1.62672037	0	0
Zfp120	13.8351949	14.7388274	15.0372758	10.5386037	6.74906849
AU041133	8.36809076	5.69588124	3.64510712	5.0005142	1.58716591
Atf3	3.20118112	1.65844896	2.32249931	0.66656652	1.63965645
Nlk	14.8524845	23.723133	11.7788321	6.48500647	5.7883016
Chd1l	2.98896001	2.91227299	2.8283364	2.38357489	0.92661231
Alad	5.03154744	3.43886654	5.64345627	2.537407	3.62315206
Zfp429	18.0680026	14.086308	14.0264328	9.33992338	6.37787625
Zfp960	6.83749431	2.92324835	7.02989343	3.79771074	2.47637858
Echdc3	1.54751802	3.9844648	5.76523283	2.01548419	0.92286866
Prkab2	8.18780894	6.14070793	3.07522673	1.3488618	1.92546766
Napb	3.19613209	1.62224196	2.20741202	0.82679649	0.26885765
Ift88	4.46887512	2.62758318	2.3926495	1.84723297	1.42566573
Dolpp1	18.3105175	14.5002038	8.39082165	6.7988217	6.48523065
Sult2b1	3.38687472	3.56088408	1.16942747	2.31890043	1.65604556
Fam178a	8.52112048	5.52980034	5.42411612	4.83831465	2.8255475
Emd	14.4136351	12.6537874	19.9738505	9.37332136	9.30447087
Arntl	4.23831132	4.03227512	5.48199287	2.20291191	3.57607638
Zswim3	3.44368163	5.9983089	3.32353192	1.84797055	2.15714972
Mtmr10	22.1381471	18.5044907	8.06293228	18.0126985	4.57817322
Dnahc5	1.20685056	1.8460679	1.59701573	0.04984038	0
Tagap	2.4380183	2.33050898	1.27601555	1.07049369	0.62858426
Dgkg	0.05922951	0.33485305	1.55761443	0.11963083	0
Rcl1	44.7403549	48.4214435	36.3716531	27.0626074	12.5788841
Fam53c	13.3483592	13.6049795	9.93147414	11.8803075	6.2358596
Tnfrsf10b	14.2977501	11.1461438	7.74994422	4.68939239	4.22464973
Rgcc	4.13815973	1.96078928	3.17120256	1.89958861	1.31501211
Qdpr	30.7814144	25.1982522	24.1280022	23.4926662	9.14692597
Ubxn2b	8.37353902	3.36317512	5.16492455	4.83004453	2.47215756
Trim34a	14.7610956	14.765981	18.7708355	7.12404123	5.65109501
AB124611	3.29301569	1.3712023	1.66324358	0.6871055	0.15993095
Hexa	18.7068386	29.3499891	22.8588726	22.9206606	9.83504592
Gm5547	0.71036699	1.00401199	1.71025004	0.52174111	0.22770125
Ccdc17	2.14876261	2.81203558	2.17060345	2.33142478	0.70441878
Mid2	5.67681205	7.38295024	5.14435952	4.43255058	2.92380187
Slc25a38	18.8499318	16.1613543	17.0727938	9.1641302	12.560295
Sh3bp4	14.3632608	16.1295223	18.002057	10.8557806	3.61377326
Aarsd1	6.84893964	3.87971925	3.71460505	4.58118444	1.92094107
Prdm9	1.16853317	1.96826975	1.97211069	0.44988862	0.66454545
Camta2	2.7925815	4.61689956	3.10566187	3.23376759	1.37194048
Pik3c2a	44.6151102	34.2361597	34.6977123	15.8273041	13.7953917
Sirt3	6.66608382	6.72974894	5.75095067	5.58784733	2.9861393
Atrip	4.15813261	5.15629642	5.91641348	3.42179609	3.29148713
Lonrf1	6.48798234	3.60907822	3.33E+00	2.71165983	1.75521165
Marcksl1	319.459888	287.686122	205.331569	166.633227	206.712275
Cirbp	62.2003217	76.6030773	57.019577	55.0077555	27.8345747
Kxd1	71.5397976	88.2605804	67.4861765	54.0600596	25.0543472
Arl2bp	136.042309	157.622467	130.759799	113.431296	72.3267638
Zfp445	17.8175743	16.67935	18.4696673	13.2141116	7.51480361
4632415K11Rik	5.32030101	8.11634868	8.02317943	5.74508113	2.72047267
Zbtb38	1.19552107	3.11658476	1.09227296	1.75614156	1.41788697
0610040B10Rik	2.66775094	6.51695056	3.43096489	2.04101566	1.90027046
Maml2	3.46294361	5.83861021	3.13777639	3.43283386	1.76206053
Rbm48	7.27684089	7.55982248	10.3924368	7.09187245	4.30619229
9230105E05Rik	0.60864244	0.59169773	3.78412554	0.63861112	0.1625787
Dnaja1	129.860238	99.1017058	174.887006	78.1719574	65.3082589
Arl15	3.62826122	3.59544431	3.32377914	2.95697281	0.80850543
Parp11	3.95753003	3.90497037	2.07553107	2.94204406	1.16470659
Letmd1	10.0889741	8.16017656	4.21453927	5.59465687	2.08705548
Magoh	134.820878	62.4898384	95.9406129	53.5677093	50.7962922
Naip1	8.11344841	4.2216822	6.55550449	0.36853258	4.19151244
Decr1	15.6032025	10.3482115	9.01999394	9.42863246	3.53210123
Mcts1	75.4847639	61.7261071	62.8665566	37.6578263	24.9952813
1810064F22Rik	1.13313101	1.02320249	1.91490169	0.83224734	0.49941947
Zfp790	5.4907522	7.20825139	5.4046227	4.92189236	3.49922732
Zfp146	2.5699188	3.00164964	3.24234633	2.65432579	1.23564385
Abr	9.74634284	9.86842884	12.2518745	4.71651358	4.50417494
Fbxo33	52.5894618	69.0801622	55.1630654	34.6779183	21.8522832
Tnfaip6	2.56610646	5.30916022	2.86649509	1.64539093	2.57859936
Suv420h2	12.9839829	14.6855284	10.7531198	6.69684483	6.99103976
Scrn2	6.61567865	4.35138003	4.37572334	4.25951095	0.75735417
Map3k13	1.22472394	1.98437904	0.32402137	0.20524746	0.07010239
Nxn	27.77712	25.3770655	28.9086461	20.3004246	11.4552594
Slc25a16	11.8547834	8.99870919	10.6889685	9.66379654	3.95965303
Pltp	1.58876763	0.65210746	0.70215095	0.53040791	0.49190279
Cd274	1.82836513	4.6165785	2.0416033	1.02032082	0.43092994
Matr3	106.989751	85.5776535	108.46882	88.5596331	46.0758222
Gng7	0.03731713	1.11346223	1.50314548	0.71946532	0.13456851
Klf15	7.27732891	8.45969824	3.55360618	4.3584374	1.29846292
Leng9	4.66900262	8.39270852	7.11042096	2.86949894	3.71058729
Gm11627	0.86692054	1.51269163	0.53516971	0.17686793	0.84908688
Gimap3	1.09446192	1.31293876	1.57648438	0.39075855	0
Ppwd1	9.42620364	4.97762496	5.69139941	5.54162495	3.3351112
Hdac10	2.26613635	2.06332815	1.83052441	1.2837146	0.69547791
Ddb2	4.49500941	5.96273022	6.77951496	5.59618429	1.4462894
Ppih	8.834289	5.1307226	5.92958742	4.0313127	3.05795026
Ptrh2	20.3773521	9.57737069	15.226344	13.9498719	7.00546563
Arsk	5.68841372	4.38256588	3.15382324	3.51982648	0.68590566
Mical3	2.46743358	2.71657657	1.95402813	0.91705607	1.04430059
Zfp955a	10.281044	8.80910228	8.65169649	8.27494606	3.4652735
Tjp3	8.84262765	10.9301827	6.57441243	4.32579499	3.13786
Ythdc2	2.79523038	1.05677084	1.80225062	1.29263245	1.14486846
Agap1	5.80582102	8.31052823	8.04208716	4.77408639	2.14353126
B4galt1	42.7506842	65.2054314	57.6819718	29.2286045	42.9121716
Alyref2	6.60825448	4.78660548	5.83452694	3.38619409	1.60097413
Stard5	128.890568	146.519093	47.9593713	68.7823681	72.794072
Psme2	5.67348374	5.34582434	5.06813565	2.88012309	2.1929938
Ints4	15.5591392	12.3907583	13.4954106	13.0111603	6.28249097
Nat9	8.8872568	8.4736835	7.21413502	6.51680045	2.54350361
Gsdma	0.4305981	0.22540548	1.61705931	0.26355008	0.24921008
Xrcc2	1.66325305	0.97153384	0.77007287	0.37244039	0.26006812
Vaultrc5	2.26823268	0.44525749	2.97549187	0.69414253	0.60588334
Kctd8	0.09028964	1.75467516	0.29262366	0.20723362	0.03617684
Esrp1	45.212763	42.9090404	31.7865045	22.4821608	34.0809376
Cyp4f16	9.4191319	13.4686795	7.44014315	9.31062306	5.57058981
Neu3	1.32726576	1.22609067	1.55435751	0.5555114	0.40667267
Ankrd6	4.60285955	4.52837129	3.86102854	4.5489608	0.93297481
AK010878	29.9681947	26.0429651	32.894745	16.5904245	19.967558
Pex2	96.401133	88.4790523	79.2238246	55.5877497	33.6382892
Mpnd	21.0186437	18.801221	14.5793729	13.3947175	5.55273836
Maged1	36.0891727	31.4260861	29.2928601	21.4069317	13.4061595
Top2b	18.5922197	14.762345	13.7214232	11.3830194	5.86307485
Cab39l	28.5797691	25.6781769	21.4538675	22.138221	9.75632768
Ptov1	36.2248684	39.9738603	27.0016755	35.83285	18.2887156
Sult5a1	0.67460369	0.52970288	3.2795292	0.87740214	0
AI427809	0.28639302	0.38229179	3.42102006	0.42069244	0.29835164
Plek	1.31468794	2.57453354	1.78940195	1.48329338	0.55849581
Raly	13.8070352	10.8691656	7.53992718	6.40301258	5.8725785
1110006O24Rik	4.2455527	6.00054041	3.22367743	2.72844108	1.2247837
Atp6v1d	191.380122	208.413664	246.893207	188.483695	80.3133812
Cnnm4	5.86364384	8.47077598	11.6236478	3.09671342	2.37222764
Slc26a2	3.00483581	2.717304	1.56316288	1.2398611	1.74507105
Bcs1l	6.9882401	4.78730922	8.91413076	5.49924833	3.31430441
Dbt	15.688839	21.2782225	40.6663138	15.2766178	10.5880045
Nlrx1	1.63848466	1.06783486	0.83446583	0.45127985	0.65650047
Slc25a10	30.7473116	42.8582599	35.9592568	31.3268691	10.5951955
Fuca1	49.3158556	40.1640611	42.5303659	24.1092784	23.6328721
Ift52	26.0048498	23.4567265	17.4843521	22.0424567	10.9238712
Ptms	76.334376	83.0357034	53.6929667	50.2802589	37.1910076
1700029I15Rik	1.61237694	1.96940814	3.81553458	2.13820689	1.43564389
Brf1	7.85854117	11.8207379	9.79749073	7.99712659	5.04554571
Trpt1	1.8241032	2.19320452	1.68909041	0.94200723	0.31975705
Rnf150	1.07058943	2.03020866	0.93523462	0.27199493	0.52338373
Klra14	0.24423854	1.59814643	1.88467485	0.52320668	0
D11Wsu47e	7.0378736	7.18170326	11.4115517	6.21598941	5.23526243
Izumo4	9.03786742	12.3371452	10.1070812	9.63788775	5.18117453
15‐Sep	437.726444	474.92362	494.164442	310.418329	210.588124
Rdh14	42.8837741	38.2295299	45.0426021	35.0189481	17.5729835
Gm9833	5.42121274	4.75030734	8.29384091	4.97712735	3.02211688
Wars	27.7087477	27.7069698	37.0310955	12.7921583	15.1533881
Helz	3.03090677	2.89868499	2.21418075	2.08937682	0.91605994
Gm14326	2.69127608	2.79593434	2.50485851	2.05267861	1.17233617
Oas1b	2.62692707	3.54000308	2.36674943	2.4769212	0.14223581
Klhl20	4.58759471	3.602207	2.16889334	2.78809042	2.39475501
Leprot	278.693417	314.318401	220.585315	163.454037	95.4477666
Gng11	6.70036476	5.87293035	10.3166696	6.29455408	2.62223702
Col6a3	2.26174664	0.89741502	1.13072146	1.20391245	0.39526888
Zfp74	2.89946882	2.44225714	3.38455302	2.33929398	0.74633366
Mrps16	156.577184	99.7856652	114.582104	74.2554674	46.8101076
Tmem70	96.1232352	71.248911	85.5570127	58.4871186	35.4216119
Ift74	11.4135881	8.21114631	7.99801087	7.02351401	2.81804536
Fnbp4	8.49559537	14.1058333	19.9695324	9.90596145	5.74943238
Ada	1.60733038	1.20198619	1.7718598	0.31621369	0.92002531
Six5	3.38287068	4.36127606	2.79383723	3.12674347	0.89751646
Mir411	0	2.49778593	0	0	0
Hmgn3	155.620431	139.955524	109.170215	83.1999535	96.4840946
Adc	1.49677326	2.83134524	1.51198703	1.748935	1.52656052
Mina	12.8966813	10.412478	10.2842734	7.38273387	4.1095891
Tapbp	170.356646	212.024331	162.402664	90.0006267	60.276579
Dusp18	2.29479823	2.91839755	1.16024685	0.90476803	0.72203714
Ccnh	28.9096356	16.1698773	19.3745337	16.1187904	7.08754722
Trappc2	32.8189549	32.5299885	39.4673085	27.7044531	17.5557667
Mnd1	3.58142002	1.81618187	2.14180142	2.05502721	0.41853783
1810013D10Rik	103.422732	104.770861	99.6288907	61.6897341	47.3822679
Ms4a4b	2.85106751	3.58188062	5.9520968	2.42715376	0.45694103
3300002I08Rik	0.30190597	0.31607785	1.67736061	0.18478331	0.16128839
Ndufs4	117.372539	75.3382061	111.558696	78.4473817	48.1695025
Scd2	196.017893	149.490036	101.959893	130.77777	74.8821952
Rps20	1035.85261	1040.51443	1398.83856	977.833528	652.801577
C1galt1c1	72.6978357	62.0435439	81.3414158	54.9832735	36.5143211
Deb1	97.5021973	88.939538	89.5846832	58.2485605	28.3019005
Emcn	2.0085736	3.15428818	4.95975212	3.23731255	0.9657432
Cenpp	13.3021947	6.46130297	9.2811832	4.88501156	3.02438681
Zfp617	28.9780574	26.6097235	29.311706	14.2160295	11.1853607
Ccdc122	3.59971558	1.72566384	1.54383452	1.18278498	0.91093732
Tbc1d19	3.17809945	2.64785752	2.58343487	2.02684194	0.99149156
Lrba	2.65788497	1.9801905	1.34340448	1.3423487	0.85377581
Cish	19.7958669	11.9422139	21.264679	3.40767446	11.4381157
Mlh1	6.12457746	3.40969892	4.55560296	3.89453233	1.16664026
Hint1	785.710287	632.412511	656.057669	606.181652	299.935918
9830147E19Rik	1.35342561	1.81888263	1.9762796	1.57709293	1.04285447
Copg2	2.00469932	3.84266072	2.26580177	2.70659099	1.48834475
Gm5918	34.2637691	29.652388	30.9600967	24.2692522	14.0976986
Trdmt1	3.09118741	3.00975153	4.62562244	2.06604139	1.8033473
Ankrd32	7.78985174	3.43462253	5.58343233	3.99985254	2.6533707
Nudt12	1.97351166	1.34748978	1.3507167	0.99782988	0.27503915
Sema6a	8.89164319	14.7137127	12.2188932	8.97960048	5.05594879
Cxcl10	1.60065056	0.93099294	0.7136407	0.38098959	0.33254733
Zfp945	3.08420351	3.62873418	3.1296538	2.65062697	0.88438093
Sys1	108.523931	79.7365344	65.6048281	47.8437088	62.4944358
3110052M02Rik	4.0766639	2.2160915	2.5027895	1.61773198	1.19664387
Abracl	707.184544	858.909947	1067.84504	257.957502	434.510917
2210417K05Rik	7.02459851	4.68839352	3.03551077	1.18234779	1.59493077
Zfp354c	1.71321935	1.00176713	1.7101765	1.0485859	0.20447293
Ncapd3	3.82761689	1.44225237	3.01760314	2.30645031	1.67132637
Orc4	16.6893717	11.6953884	16.1697833	8.55110942	8.42963489
Ndufv2	60.4714377	38.7165303	51.0318734	38.0287067	16.72096
A630072M18Rik	2.57953818	1.76991911	1.6965836	1.2947811	1.13015161
Emc8	16.9870304	7.88881102	9.5480004	9.38158318	5.02129039
Nagk	13.2074308	15.5558314	11.7627567	8.92573563	5.36539093
Dcaf10	5.96696452	5.60105899	3.14775451	3.3782068	3.31091765
Uvssa	2.10073147	2.54415487	2.4892526	1.50369122	0.71331474
Zfp97	7.76498221	5.48554651	4.78621465	3.293597	3.12069389
Reps1	12.8779387	14.7863584	13.4240105	8.69003868	7.32562247
Slc22a18	4.24857965	5.7908104	4.08257408	3.42218238	1.89501464
Rdh1	1.20700299	3.93577862	3.74107472	1.49289715	1.50458432
Peli1	36.8441368	47.0465326	33.545287	21.2507701	12.8506812
10‐Sep	16.6837744	8.39261719	9.69254045	8.57216325	4.2333644
Trim30a	16.1904884	11.8300309	37.8487301	7.47975395	6.90297188
Rnf146	13.5635576	19.8935264	16.7416129	11.664006	9.48085666
Nfix	11.6395769	12.3520471	7.29877376	11.9279638	2.81025913
Med7	87.2782714	95.5063481	116.852213	41.3320209	42.1064203
Xk	2.68967508	2.51809313	1.58057062	1.05659559	0.32123351
Sh2d1b1	0.63709955	2.10821499	1.179888	1.29515951	0.58347453
Parva	40.8855312	29.8252479	18.7532686	23.6250239	8.93660901
Arhgef19	12.3819664	7.59703392	7.11038941	5.97464226	3.66125115
Zfp518a	11.3548424	8.9308628	7.90125131	3.71997982	3.07127046
Cbx6	44.280281	74.2180257	47.241912	33.8518685	17.8666923
Evi2a	2.61301195	2.20786435	1.89360571	2.32073674	0.63728583
Rps14	1787.14984	1503.11695	1869.10232	1173.52817	704.468775
Dus1l	37.6786242	60.1097614	55.134114	19.1307353	23.4597298
Cd53	11.6133594	7.74830667	7.54273509	4.89299877	2.31260609
Clec12a	1.73154351	2.65010366	1.57620565	1.77831068	0.23518199
Gm13212	4.00939863	2.6235033	3.09386587	1.84048188	0.56698847
Igdcc3	2.28157947	1.87294222	0.4993669	0.15234044	0.38229042
Iqcc	12.5430514	14.7255092	10.9944645	12.941043	4.80612289
Gpnmb	3.02192137	1.69873119	1.19243645	0.37832413	0.0275184
Pmvk	59.9722377	45.2545405	218.284092	42.0201667	22.0341786
Spns2	16.0921913	42.1189519	29.1813441	15.2312693	15.2596631
Lrrc36	0.07831668	0.96341743	2.41733315	0.09586836	0.1882774
Gtpbp8	9.65173012	6.1649724	9.81719033	5.87678948	4.44829416
Efnb2	75.7355487	53.267497	82.265625	16.0105605	29.0023335
Tbce	9.27471179	11.9864697	8.22064504	6.62642975	4.68495014
Greb1l	3.08983068	2.03699618	1.07561422	2.22529927	0.83775728
Dcakd	37.3997029	38.5378932	25.9648377	30.2492652	15.3662477
Ppp1r14d	3.60334134	1.44918712	2.5228227	1.61374105	1.5494119
Pbld1	1.98017997	4.50642747	2.23763186	2.95805461	0.61859036
Gemin4	6.24125799	10.7517804	8.16107936	8.97699619	3.65256453
Vps37a	12.0323183	12.7389432	14.6915648	8.10902978	8.24125761
Zmynd8	9.06152829	7.45325228	4.84783394	4.40219065	5.12850604
Nub1	20.535548	27.6401159	17.0755796	13.0136655	8.77259446
Sprtn	1.04782488	1.54872171	0.94173176	0.56587706	0.4692303
Gars	52.1966369	50.6689984	35.2203619	29.8346959	22.5428528
Cdk14	11.4860744	13.2208201	7.91853562	3.73043303	4.45853521
Gltscr2	42.8043331	47.7515067	40.954747	28.5313977	21.0103764
Pkig	110.119038	66.968163	71.9877065	44.2935704	35.5834306
Ppil3	26.8048049	16.0210748	19.6343807	14.6552438	8.36760199
Ndufaf3	13.8735272	12.6691822	15.6197329	11.614852	6.95459808
Tsacc	7.99893905	5.32272469	11.1731048	6.22347118	2.17286643
Ptgr1	20.3011676	23.0956161	21.8407658	4.43901505	13.41208
Myef2	12.031677	14.5343774	20.5682596	12.5245635	6.80844845
Khdc1a	1.40455947	1.28667998	3.90179884	0.06140492	0.61636978
Zfp566	7.93948466	4.66046701	4.42537693	2.65380287	2.37814581
Psmd3	62.9336845	102.027633	94.6134511	49.4964519	27.5039146
Nfkbia	96.1449102	117.553336	361.047888	117.009614	67.4812638
Ccdc66	4.11583789	7.88832495	9.52800022	2.98637921	2.25201717
Mpi	21.4526787	20.6916757	15.2251264	13.0507289	7.91148459
D6Wsu163e	4.89524747	2.97435188	4.10121415	3.74520598	0.77055203
Syn2	1.339808	3.02003819	1.82887565	1.32549134	0.57385072
Zcchc17	33.7381526	22.753573	18.4878187	15.6993787	14.6661554
Tmem129	18.0756109	23.2291865	13.4816738	18.4091609	11.2712121
1110059E24Rik	94.1642184	65.5834975	72.901757	39.9131952	25.3602271
Lefty1	0.69114634	0.82999994	3.53877077	0.41057838	0.81448625
Twsg1	38.5920426	21.5977939	21.4562896	24.3059228	11.5438965
1810044D09Rik	5.09885638	6.2161977	4.7214595	5.05074384	2.04298625
A430033K04Rik	2.42831562	2.3304454	3.82451032	2.3246705	1.03117796
Cep19	33.5451078	37.4476994	24.8852865	18.0911661	14.3469861
Rara	5.44614691	5.5902396	5.9347138	3.46378128	2.21376219
Rpain	8.97803145	5.74099519	15.1335817	6.61381058	7.45303934
Tmco6	7.43736624	7.81469829	5.35646397	3.62847229	2.18819023
Mgmt	1.91096526	0.81277161	1.47460274	1.02341526	0.25522558
B130024G19Rik	2.66317054	2.00636171	1.3996519	1.51987333	0.11535858
3010026O09Rik	5.31990099	4.44671626	3.23112623	2.31076393	1.87943416
Tmem55a	40.0708671	17.6521161	23.7832078	17.5933163	10.8869662
Rpl31‐ps12	0.67367448	0.84635722	1.87143539	0.24739584	0.21593983
Hoxd9	4.78219057	4.29143411	5.75095067	4.1908855	1.86633706
Zfp133‐ps	5.68109251	3.17952639	3.35783033	3.44015491	1.35607809
Golga5	40.1112563	61.0326503	30.2549152	20.4035924	22.5632562
Rccd1	1.42128041	2.73528908	2.19347157	1.02341526	0.96028625
2310009A05Rik	137.315672	103.76219	127.520318	98.931182	58.2054387
Cyp2j11	1.01155672	2.14455715	0.06244569	0	0
Gxylt2	1.71421747	0.33650347	0.99208623	0.327874	0.05723706
1700030K09Rik	3.11683395	3.7343289	4.40384912	3.82357556	0.62441932
Mndal	3.75958378	1.23449273	2.05714037	1.7258064	0.30127454
Trex1	11.3561254	20.8961651	33.4055881	9.8993149	11.0765545
Hist1h2be	2.15640248	1.18321413	0.69767509	0.6678702	0.47885302
Ppp5c	15.349489	14.1808808	10.6235541	11.3720776	6.41536451
Brdt	1.45347447	1.7017254	2.10295957	1.70375729	0.59277094
Ppp2r5c	25.380189	26.6417841	18.3822643	17.1867318	10.0850966
Fkbp3	31.3292138	25.4668438	32.7165194	21.0969746	12.0551549
Hipk1	25.4721769	32.3106252	25.1472804	18.6028515	11.8356701
Zfp791	1.98543309	1.51505926	0.62145779	0.25673153	0.0896354
Ostm1	37.9480453	38.3566615	25.6671293	19.2602059	11.9832609
Nudt3	71.1591398	66.2994763	54.0433073	60.3937569	28.5959631
Aip	17.0022851	19.3298629	14.1608252	13.2980569	8.34425244
Pigp	46.4092344	38.8721507	39.2433293	20.4817712	17.0404688
Atp5f1	449.226131	379.497914	367.659935	203.41749	134.41216
Gm20300	17.9521715	20.1990284	15.4999823	10.6686578	5.46880162
Tmem35	2.00599045	1.28145013	0.72411605	0.1560735	0.13622898
Dapk1	7.54851402	10.784459	10.0421192	7.50516021	4.55216433
Zfp961	41.4830799	44.8746816	50.4096852	28.3269145	13.4777277
Tbccd1	10.1848013	8.14877238	9.65444569	7.26768765	3.36520879
Nfu1	37.9661774	21.7150447	22.5732341	15.5473389	10.2873281
Glyr1	77.2929721	93.8242017	51.8100264	51.9216849	41.5471574
Med31	52.0682482	51.1164038	55.807998	28.3620896	27.7265992
Trappc12	7.91604684	9.30555748	7.18835076	4.31557323	2.8343433
Elf2	7.87121664	13.5282737	7.06296688	6.30914857	7.24495866
Cln5	455.149344	885.607507	1176.95191	580.763903	206.531132
Ehmt2	17.2420863	19.6775969	16.84298	12.7787076	6.5482609
Tmem53	13.1381368	10.6922536	5.82940016	6.97329171	5.42863204
Axl	12.2624656	12.2371367	7.82724295	4.40426878	5.28330507
Aspscr1	19.3439272	20.5803677	14.7815723	11.93556	13.3227139
Zfp108	2.77602023	1.55696272	1.90496143	1.54737587	0.15889757
Srr	1.53377048	1.67498189	0.96315597	1.19771956	0.72049995
Wdr33	13.5567839	17.682168	20.3205726	15.2904867	8.69876971
Bloc1s1	88.0950642	94.1233859	118.903519	77.2864598	38.0054092
Igsf3	16.0582673	27.7748343	14.48251	14.5713111	10.4158413
Kbtbd8	1.34585827	1.08945982	1.60598357	0.73881872	1.03403015
Acat1	15.0607489	11.476634	12.9613777	12.7442962	4.15208814
3110009E18Rik	2.75458009	1.36357551	1.44169838	1.15451391	0.23993314
Shprh	2.90640968	2.7727333	2.63278262	2.82301316	1.16452682
Dusp28	7.04163784	12.6036837	11.7853989	6.34435095	3.50485833
Tstd1	2.24867895	4.92530746	0.5479581	1.41253595	1.2329341
Fam118b	44.1300269	48.1973566	39.5537607	22.2373516	27.3176447
Zfp873	17.3236271	14.6044457	13.8885459	7.47070893	4.24875689
Vill	11.2750228	17.3375477	16.6964993	7.53763772	4.96580111
Erap1	17.5619921	10.1541781	12.7419232	8.71050299	5.3377054
Gramd1b	2.89675238	1.87422707	0.85835085	0.76592485	0.76139124
Bcl9	3.23814191	2.86987498	2.73127746	2.74722091	1.49869741
Cnpy2	135.681352	105.732236	112.991761	123.860417	42.1348072
Wdr5b	3.76291542	4.3889667	3.07845007	2.38137551	0.93682802
Ppm1l	2.75426205	4.83037939	3.74160865	2.58920955	1.17625492
Ank3	3.91819417	2.16128028	2.09101014	1.91628162	0.577106
Pisd‐ps3	78.5364672	105.485434	68.4208459	61.187221	36.6513919
Mterfd3	3.41248152	4.1172821	3.66086362	1.68109182	1.23390249
Cav1	4.38358303	6.40288962	6.3069244	4.71688284	1.75639382
Immp1l	26.8358052	24.2886796	24.7862963	20.3745368	11.3817247
Fam89b	117.133538	151.585929	85.5453912	75.2818186	53.7665221
Timm8b	324.821034	322.676499	463.486247	285.489164	167.580777
Dpm3	112.990551	93.5371369	113.744979	67.131228	34.2777747
Zfp433	1.6138799	2.00823455	1.44376845	1.43145058	0.87680265
Krt10	20.0747266	16.2278477	13.5086862	9.90198294	6.89438471
Cnnm2	1.13581922	2.5525422	0.8341469	1.04277717	1.16127639
Coq3	12.8027542	14.3639419	14.3278564	5.93428103	4.07979996
Scarna10	0	0	3.40197082	0	0
Ppapdc3	1.64459305	3.8178877	2.64846412	2.19916454	0.71624778
Dclre1a	7.1390313	6.91274824	5.24700139	4.21763145	1.60338235
4930577N17Rik	3.72185721	2.95351433	1.61319928	0.69066549	1.04705249
Srgap3	4.09499468	6.29829399	3.4996923	1.51594681	1.8759985
Nipal3	5.15204791	4.89118677	5.03508606	3.22879878	1.31033575
Fundc1	99.9912573	102.69807	103.088003	49.2774448	34.7846944
Mirlet7d	31.65616	20.8795503	38.6932895	30.8067866	7.1029527
Mgme1	7.37154673	3.57505415	4.99676246	5.44069783	2.23932869
Pced1b	15.2951054	22.7395025	11.9946533	13.0897138	6.88848042
Zfp869	34.8754581	31.4620142	27.2605934	19.6879181	9.89463372
4932416H05Rik	1.01266939	1.67313677	0.8595727	0.65854835	0.57481491
Asb13	13.6646901	7.60632819	8.64432316	10.4834243	3.53442421
Cdadc1	8.91189554	9.76448492	9.45207133	9.71959321	4.22921837
Zfp784	5.65208493	4.17558575	4.38958863	3.18040838	0.92534139
Arrb2	3.11625277	1.90600667	2.49072864	2.58994073	1.28803544
Prr5l	7.01106334	17.402197	17.7379187	2.67973207	9.96723058
1810014B01Rik	3.7033799	2.0861138	1.93828337	1.84783311	1.03224568
Col1a2	16.1285765	8.88090871	8.53585105	9.81338427	2.67231307
Skida1	4.24481316	4.82353778	2.94797185	2.33276754	0.53898343
Stxbp5	3.25555382	2.11048322	1.15792229	1.36577552	1.09997472
Zfp113	3.17491224	3.60094254	2.90258569	2.45216307	1.28422451
Pdcl	46.7525374	44.2889432	42.2779388	25.4100975	18.7600179
Rad51d	3.19547438	3.09649754	2.21494269	2.55216083	1.27294707
Ccbl2	6.53278279	5.24357108	4.03283495	2.37240052	1.18661145
Mrps18c	41.5692045	25.1943009	30.4330686	19.2012682	16.1352646
Peli3	2.01998006	2.37915077	0.06234898	0.24726812	0.16187126
Sfxn2	2.65962993	1.91299919	2.36877576	2.06897478	1.09005241
Ccdc126	5.80042329	8.90613014	5.10520324	4.87710773	2.02494695
Fkbp15	5.34669771	4.70535237	4.32513344	3.68153724	2.34689092
Pex3	16.2690286	13.5835934	10.5705104	9.03648016	4.36746153
Hint3	12.4614292	18.8338801	17.0917262	8.08586282	7.13283992
Adrb2	1.3382996	3.96453615	3.01361617	1.06112506	0.58497132
Itgb6	8.10360526	6.04234457	4.66772366	1.00759338	3.11908386
Mir219‐1	0	0.46549647	2.19581753	0	0.47506762
Aftph	26.9847158	42.234744	29.3224344	20.7940079	12.8001661
Paip2b	9.29913984	9.50458644	9.80983463	6.34000212	4.48055648
Cpe	13.9516891	18.8777269	24.6845963	13.2662769	15.5973728
Metap1	65.7233544	106.185789	80.6159646	44.5559031	52.031925
1110054M08Rik	16.4174628	20.4458695	12.586097	7.64146067	9.48466375
2810013P06Rik	10.8686149	9.39256188	12.2080092	7.08518258	4.81860989
Wdr34	9.89043958	5.63250727	7.17910342	7.05133115	3.2515739
Ifih1	6.085164	4.08226655	6.0833575	3.61236474	1.79904207
Gtpbp3	7.17328585	3.98664476	5.62874297	4.38332411	2.37024807
Apoo	6.20571193	4.66973063	5.31541079	3.56085207	1.98918082
A930015D03Rik	2.20364908	2.81977836	2.41842231	0.97410176	2.55073851
Sod1	97.5156283	85.5780776	94.4913145	77.516599	42.9833554
Sccpdh	81.8369382	128.757486	79.8011729	34.5762071	51.8767904
Kctd13	48.1399474	41.9171971	25.4829005	23.6004262	14.5019129
Gm10451	13.2175989	6.6514389	7.43823967	6.23504001	3.51113803
2610528J11Rik	112.534708	184.28064	120.70397	69.1610612	115.480371
Ogg1	5.11381363	3.05302933	2.9351093	3.95769207	1.42243188
Hps4	4.88056566	9.31974307	5.77115781	3.2606528	1.68927768
Churc1	108.788202	117.874779	104.8941	63.3364318	36.3921123
Tnik	2.54233141	4.06928688	4.21443713	1.22416425	0.88330456
Mknk2	61.9160222	58.4336943	34.3960103	51.1635888	21.8605453
Cox20	182.388944	126.411385	174.550339	151.411775	74.0702521
Orai3	23.915309	30.1943025	23.7486027	23.2160419	8.2732216
1190005I06Rik	1.51035248	1.46498207	1.06948878	0.89723123	0.42717252
Zzef1	1.90663774	1.74968224	1.77634745	1.4927177	1.08732965
4933411K20Rik	9.60225188	6.63407083	8.54441488	7.66407473	3.24659297
Mettl17	6.83055282	6.59381627	5.24601508	4.3897137	2.02847725
Gltscr1l	2.72582682	1.72005155	2.45982295	2.32963474	1.13585858
Trim14	5.92005561	6.29330421	4.25899664	4.13904294	1.27724184
Ddit4l	1.53382879	1.12909838	0.71015047	0.85811169	0.34569419
Numb	9.70568187	15.4753546	21.1161214	8.6055803	9.45931093
Rnasel	3.3578949	4.65985641	1.96714422	0.55365088	1.84515543
C230081A13Rik	1.68092797	2.52315282	3.57502854	1.77771677	1.5945206
Ranbp9	34.3510662	41.862778	38.1938953	23.0764443	18.0103963
Ppp6r2	0.7961805	1.15596681	1.55796314	0.56545992	0.40929576
Nek4	1.20139443	2.22628746	1.66870805	1.01474225	0.77019068
Tasp1	1.50989141	1.79880472	2.98911688	1.79250877	1.04306263
Smoc2	2.85531409	1.44645795	2.23884997	0.84561854	1.40238887
9930012K11Rik	2.79103404	5.15655705	2.46283159	1.05510712	0.94726473
Psma6	195.962108	138.360304	210.10582	134.091522	95.5519648
Gyltl1b	11.0569325	12.0406884	11.5837527	11.6825132	2.56544352
Prepl	3.29712478	4.81753056	3.98160033	4.11926643	1.86585066
Ogfr	26.4494965	23.4091013	26.2336854	17.2962994	10.0951868
Adck2	4.80933907	5.02062735	4.00974026	3.77252438	1.21082506
Snord8	7.24574328	4.65496468	5.48954382	2.41898153	1.58355872
Becn1	45.6578151	71.4998908	56.2820143	33.162736	32.9085489
Mir467c	0	1.16374117	8.23431573	0	1.18766904
Zfp865	3.65765414	4.28214412	2.92923612	4.00846263	1.46553198
Exosc7	40.5607905	30.2684926	27.6012358	31.9267072	10.5321162
2900076A07Rik	19.3191539	17.7477342	16.8286016	11.3570333	5.20126722
Mitd1	51.4689494	38.9555019	52.8675677	35.2203907	15.4773859
Zfp963	5.89210813	8.35902432	7.67002609	5.20038794	2.00726954
Skap2	17.5462813	19.5571	40.8215742	20.0540063	20.3099377
Anxa6	55.4336121	31.384713	39.4397215	28.1912252	10.5064747
Lancl2	12.0187801	8.8491426	6.98464517	5.00961351	3.2866313
Loh12cr1	24.0377627	27.756759	19.9828464	10.5398035	13.51625
Bola1	39.4490468	20.0182638	26.4236067	29.0725743	13.9066432
Arl2	24.7639327	39.1841964	37.3844883	29.7626588	12.8087851
Atp6v0a1	4.16175918	6.03977911	2.95402073	2.40846944	3.41288952
Notch1	4.24710972	5.68220634	6.62459335	2.90862836	2.35155716
Ufsp1	3.92841503	7.0432046	11.5192235	4.14760613	3.30544034
Klra18	1.11337031	1.87333945	2.79832844	1.46023885	0.08497144
Efcab2	2.20969476	2.76399991	3.83056821	2.4061146	0.55593019
Rps15a	28.8714784	21.9918521	29.710258	21.3754052	13.4562393
Slc1a4	2.32542958	5.68650296	8.29024588	3.53789912	3.82014382
2300009A05Rik	26.1719155	23.9754034	34.6355332	23.2270768	11.8846347
Ndufv3	41.9188505	39.0439217	33.6916841	22.4085165	20.8831179
Fggy	1.29798424	1.67976794	1.40223143	0.55169363	0.21188051
Amer1	4.62827414	4.69234513	3.48704018	3.71181403	0.9935578
Mrpl24	25.4937164	19.1545175	24.3067051	19.2416089	10.9580928
5830428M24Rik	1.94943334	1.38257389	3.26090549	0.75053742	0.2015716
Eif2ak4	2.62188767	1.6879569	0.94351534	1.25489276	0.29872773
AF251705	3.49246099	4.13592999	3.44602619	3.62687946	1.00936227
Gm12657	13.9358314	15.2880844	19.140434	10.9577637	6.67912036
Smox	84.4407775	162.170573	100.321292	24.1384841	127.293759
Gm14207	4.26307266	4.70726766	5.05778195	4.03616581	1.86824343
4930455F23Rik	10.7654319	8.23702875	6.6245128	5.46280282	3.22474695
1700011J10Rik	11.4276806	13.3033777	8.94982297	6.05483519	3.87260872
Ccnd2	4.21486054	4.26779036	5.39917705	4.05633148	0.41653823
Zfp213	12.9740534	11.049013	7.81346439	8.06025632	4.69027115
1700001L19Rik	0.93777872	1.52934127	0.5009816	0	0.37574485
Coro7	1.47698494	3.74345641	4.83010608	1.97391805	1.25849334
Stx8	3.38452482	4.54153962	3.48714443	3.58868641	2.21559312
Wdr8	3.50512831	3.26429399	3.50987708	2.20769861	2.3189238
Zbtb8a	25.4527456	25.7286518	11.3509679	9.40081986	7.57252238
4932415G12Rik	3.53262841	2.8976733	3.12512362	2.01255168	0.65716729
Manea	12.5688909	6.81547984	5.76710503	7.49019231	2.68739632
Aif1	7.7508751	2.69189958	4.07167308	4.31062508	0.59722786
Kdm4a	1.4737105	3.4508354	1.86418528	1.66707655	1.41293314
1810021B22Rik	2.29337746	4.61937627	2.12363087	1.83088051	0.55933037
Mdh1	211.521675	169.513939	196.975935	141.479842	57.8888572
Clock	4.7003344	5.77233051	10.1906723	4.97980892	2.86514435
Pnn	37.3148469	50.5108621	69.3844158	44.8894007	45.5087112
1110001J03Rik	93.7510457	52.0173831	69.9698998	55.7970178	27.7284363
Mttp	2.01106344	2.7750345	2.50577136	1.37116759	0.79393386
Mmp15	7.23896998	5.87554668	3.64087996	3.00818075	1.83188027
Rpia	8.20666296	7.10854088	5.86129623	4.25710716	3.68633167
Lig3	10.859474	12.9109693	11.7925928	6.0020852	4.27201257
Rps15a‐ps6	18.8354152	16.7782101	23.8901749	17.2924887	9.32262541
Acad9	17.3320105	13.3082264	13.2559927	11.5210718	5.38199524
Fbxo44	1.68710284	1.59507502	0.51011601	0.94830717	0.05518209
Ap4b1	8.4932063	9.57227102	7.61556303	7.03624369	3.39403977
Syce2	24.8402867	21.7423806	22.6498658	9.4187001	16.5944959
Cdc14b	6.74641207	7.14284252	9.33030091	5.32464535	3.19142703
Mettl5	19.9710799	13.1211817	16.417167	12.223416	7.07652802
Depdc5	1.57508698	3.6011768	2.45229089	2.24706658	0.68059651
2310003H01Rik	8.07187212	12.1278324	9.4904271	10.3711452	5.16813715
Zc3h12c	4.08304073	4.17451586	5.75001296	2.82119193	1.16733556
Adh1	4.39958925	8.1374645	1.90117635	0.80783828	4.19156359
Ift172	4.95599194	2.76474604	3.91921758	1.45024831	1.39147024
Rsbn1l	5.83547168	5.02457927	6.82783016	5.10952139	3.23668681
Rpl39	666.350919	538.428187	698.718612	543.994564	326.012137

**TABLE 4:** Gene list of cluster 4

	D3AM	D4AM	D4AM	D5AM_mesometrial	D5AM_antimesometrial
Stxbp3b	3.10840228	4.83062373	3.71784398	7.1344341	4.0996401
Gsdmd	3.15777325	1.15325702	1.59802599	2.69683751	1.17696932
Daam2	0.19417873	0.17788203	0.56938693	1.93128364	0.17289475
Prss23	59.5112257	93.5841257	500.244465	271.975144	155.865178
Iqcb1	3.85459479	1.58108637	3.78238529	3.22192974	1.06036265
Snord33	0	0	3.6820111	2.19035827	0.63728583
Gm5113	1.12548094	0.81442191	1.26696071	1.62084041	0.17684412
Bend3	1.75723349	1.33746547	1.88671873	3.6620637	1.56366279
Hrsp12	40.609694	34.5141748	38.3329212	49.0884116	9.62083612
Kidins220	8.75714934	10.1551432	7.37476679	12.8927925	3.92530875
Klhdc8b	1.3912416	0.93635253	0.8383963	1.90577735	0.42471334
Mapk1ip1	7.18869019	10.3148397	11.6490713	16.6959724	4.14904853
Zgpat	6.70036476	7.5042999	4.78558798	7.22443139	3.392259
Zfp874b	4.7024336	4.07247706	9.09296654	10.9835357	3.53278012
Emb	119.107087	210.071703	156.973317	241.739701	85.3817174
Mylip	7.82627719	6.33406023	10.7490531	11.3598065	2.10221003
Mettl22	2.13564558	2.02790541	1.42569265	2.64466716	1.67160121
Vim	21.4764779	36.9376778	38.7201412	42.4376939	28.8356199
Fktn	6.96079832	2.99778625	3.48168879	5.75330652	2.28684234
Hmga2	21.5029925	13.5368513	15.7469458	45.7195329	15.479514
Ell3	4.90840674	2.93646518	1.76754129	8.01125065	1.84180933
Tbc1d2	3.84756542	3.6872078	4.90934813	8.01045311	4.35782117
Alkbh3	3.28442303	4.15959476	5.64116404	7.24662805	4.37247448
Cers3	12.4766751	12.4743317	2.87901329	34.584888	3.37559579
Tlr12	0.12327352	0.12906014	0.01902488	2.07488255	0.09878533
Enc1	9.12936155	5.67860545	4.48144923	15.6522334	4.01048014
Zscan2	1.19811503	0.91836792	1.58490767	2.95944295	0.25145749
Mical2	1.84043846	6.29462389	3.99016096	4.67910457	1.03522197
Klhl8	2.37095931	2.13689811	1.35546435	4.16419678	1.27215387
Fyb	7.64903643	7.67017154	22.5118461	19.8820492	8.78440305
Clec16a	1.36815044	1.91894284	0.88972107	1.68755342	1.07962996
Hsd11b1	3.11391459	1.34655726	1.71334551	4.2675354	0.10849295
Cpq	25.9810109	15.6182968	14.7887711	21.8005753	4.93239601
1700125H20Rik	2.9428865	3.99133382	9.66159713	13.8775109	4.50217925
Nr2f1	0.24132615	0.52636319	0.84419794	1.55090335	0.0644623
Sostdc1	0.03724254	0.35091681	0.86214994	13.9160512	0
Inadl	4.60509433	7.26677616	9.46086231	8.19743076	4.29545696
Fam195a	7.5121309	2.88653447	6.21610109	5.35599862	4.09862256
Lpar4	0.5848582	0.56800018	0.5130657	1.89345108	0.2096711
Rab3ip	28.9452629	31.7506662	24.7376395	48.2964499	8.1785804
Sgsh	1.31207609	0.94735637	0.51670776	1.5784358	0.33839229
Klhl5	11.4809313	5.58923577	4.39421231	18.0593154	4.78413162
Ppa2	13.8931593	11.8392165	13.6127994	15.6719441	6.04132228
Mtcp1	7.05337842	7.30055917	8.66883715	9.47791213	4.08502094
Plekhb1	2.84838963	4.88175003	4.4635686	14.7348587	4.60901383
Mon1b	4.94430301	5.81848019	3.4659598	5.24745905	4.03305414
Chsy1	4.65351616	2.28066517	3.45594126	7.0106151	2.62788839
1700102P08Rik	0.17817402	1.30576423	1.6498629	1.58126047	0.28555977
Smim6	8.49424059	9.34812395	6.37913144	39.0535264	3.10864758
Calcoco1	5.93463322	10.0450973	8.57449639	15.7518612	7.43891287
Fcrls	4.51994634	3.18903658	3.15421303	5.29235737	0.28871513
Dbn1	2.31140875	0.80663648	1.30797795	3.32064161	1.45778871
Adap1	6.52388724	2.56129778	2.36606432	7.38701903	3.41993235
Rpusd1	5.70554418	4.14928737	3.0139304	7.73420694	4.11185946
Ctsw	1.00569976	2.1612336	1.66646866	1.70084639	0.76350153
Cd74	59.1744236	30.5418319	25.34889	61.7314683	12.224178
Cyp2r1	1.3644292	1.19740015	2.22959934	2.35794876	0.77180216
Proca1	1.42233031	2.50438946	3.47223624	4.59014935	2.55588261
Fam53a	19.0784074	22.7007963	17.2743858	21.5507342	9.99231085
Fyco1	2.29835193	2.89130035	3.01250469	6.32280884	3.6446612
Pdlim5	10.9420084	11.2977879	10.8678785	21.4975797	7.38753854
Mipol1	1.39034551	2.95817563	3.36395017	3.66465719	1.76108284
Gatm	14.4423004	7.73391672	9.42794798	13.4370473	2.28363007
Atp6v1e1	109.570804	129.585093	138.524353	240.125606	142.9701
Nrp1	3.83338986	1.60014411	2.35623832	3.92389858	0.89155426
Fam13a	1.39750185	1.34780366	0.15472509	2.24529155	0.31649042
Tifa	2.41072694	2.82739549	2.37357908	3.55800828	0.68470204
H2‐DMa	1.29794075	2.40890211	2.14880214	2.81655237	1.29225268
AI463170	2.29448537	6.0686947	2.12465678	6.20871925	3.09673705
Mavs	12.7396935	13.4715174	12.2512021	20.9516114	6.30890985
Lrrc28	7.02468094	11.8367854	13.1370285	13.9101373	7.4811164
Kat6b	2.44421625	2.77064615	1.56373812	3.50879254	2.07975628
Tln2	1.29520498	1.2322191	0.40807389	2.02296152	0.99915325
Pfkfb2	2.18253446	2.56528664	3.5900852	3.27320559	0.7549926
Rnd2	1.048726	3.14414281	1.32423206	4.63903365	2.52119217
Slc1a5	70.8627388	63.5137694	59.3121524	154.927742	39.8846651
Per3	1.50075617	1.37623683	2.50547465	3.98260338	0.51792186
Crebrf	4.61110431	8.58791955	8.81508929	7.8562535	4.01063389
Arsj	0.30043326	0.15264247	0.75276671	2.27148265	0.12745717
Tm7sf3	8.34835639	5.16375008	6.23540575	9.0202375	3.40809376
BC053749	3.12042959	2.34635892	2.00793533	3.1561529	1.20789698
Park7	164.43745	98.5532833	137.019743	155.475819	57.6394329
2810029C07Rik	1.03457645	0.28825523	0.61806532	1.71581801	0.53487654
Rasl11b	3.46713734	1.10055161	0.54647043	3.4201635	0
Nit2	13.7421212	14.6128246	17.2327282	23.2309694	7.43632278
Ino80d	1.28697769	1.77885782	1.65591162	2.31442839	1.23990225
Pik3ip1	12.4434254	6.34635845	5.43814818	14.4135926	2.25990703
Gpr64	2.08754826	0.80329473	0.96011737	2.65270017	0.49853396
Fcgr2b	1.34978918	2.55029437	1.4842363	2.05245732	0.37181877
Acvr2b	2.2618987	1.9566724	2.30748021	4.11802808	2.18123344
Gphn	17.9193051	21.6026516	26.8259261	27.5482071	8.54814221
Vps11	5.24974608	8.88834766	7.42469433	11.8045158	6.83619311
Ppm1h	4.06922331	1.64868494	1.21316289	4.25365478	1.63391403
B230216N24Rik	1.16251739	0.82588083	1.79412086	1.88046038	0.62105614
AV051173	0.94055321	0.24617602	3.29020736	3.16619096	1.00495073
2210404J11Rik	2.98194382	2.51416946	3.2817925	6.79930555	1.19479149
Mfge8	13.6496716	15.5107102	22.3816396	37.0877315	15.3871853
Gzmc	2.79279184	3.23455255	2.32747254	3.14092061	1.39875369
Tcf21	2.07274319	1.96138284	1.13191083	8.9292356	1.27768796
Kdelc1	11.5607642	6.08109844	7.43119831	9.09381964	4.49719774
Inpp4a	1.48295362	3.44819787	4.27018212	4.61144971	3.00541602
Akap7	3.38273894	1.88553645	1.91422133	2.79890802	0.83665446
Mblac2	1.22660483	1.46449675	2.17827815	1.94372427	0.9425459
Fryl	3.94567677	5.19635291	8.88664775	9.29625989	5.94103738
Beta‐s	1.12257995	1.44238342	12.0958963	4.59095438	1.63560055
Tef	3.59849162	9.53338438	9.38689828	14.9070144	2.80159884
Ptpn18	6.88184391	3.71095087	2.57202403	9.24879825	2.42517034
Gm10664	0.13376757	1.47049141	2.04379939	17.2547813	0.10719474
Mettl8	5.20262107	4.25804494	5.07244045	8.0618252	3.35294662
Sipa1l2	2.45888539	4.23152014	3.38687173	4.49611327	2.84891294
Rps6ka2	3.01515117	3.49445194	1.1502965	3.00068686	0.73452289
Xrcc6	14.6286516	11.2684072	12.8886072	16.2353958	5.4903697
Sytl1	1.55830531	3.80377114	3.98384037	6.68675608	1.14016228
Sfpi1	3.22878583	1.6133486	1.31370179	2.91938224	1.72492668
Ifrd2	5.17082589	2.17231685	3.90367561	8.22453719	4.11725267
Batf	1.94769427	1.86919489	5.00981599	5.2319841	0.8092966
B230217C12Rik	1.97444981	1.89487208	2.38695892	3.27294914	0.96691643
Hmgn5	80.1211267	48.38527	32.3615373	74.3113789	21.4291118
Itpkb	0.70312545	1.86709621	0.79313738	4.01401081	0.2356246
Stx2	3.76778651	2.22324296	1.36253293	3.64335833	1.98310276
Tmtc4	6.77124328	3.27769932	6.84006876	9.80169174	5.96567209
Srd5a1	9.71446182	15.9666162	65.7097366	71.7763557	12.3927582
Dcaf6	3.63054489	4.60144315	5.40815573	10.7421444	4.37982156
Hoxa9	0.31572561	1.10432481	1.7807191	1.23850364	0.46001266
Gns	31.0072703	35.8365868	48.0730246	52.3220057	14.0573185
Frrs1	10.1007237	8.82563957	13.3615838	14.2237241	6.45103462
Ezh1	7.21464567	12.7462123	8.72515477	10.677779	5.46996069
Thumpd2	2.14327212	1.9587971	2.34252086	3.77411948	0.59127489
9230114K14Rik	1.66262098	1.03956482	0.88382174	1.49815818	0.46878721
Rfx5	6.74066879	6.01400546	6.01110946	9.0183431	2.05836171
Apcdd1	4.2402742	3.65877896	2.437836	14.2241558	2.09104431
Fam71e1	1.30576999	1.68872688	1.92037045	1.90398634	0.55396577
Fam118a	2.04059045	0.65734725	1.74420381	4.8356987	1.13208143
Leprel2	1.54874829	1.27136315	1.21682584	3.08074141	1.39152587
Dock7	5.99988284	3.9986563	5.22999277	11.8668509	6.00259365
2310069G16Rik	0.37825806	1.12863993	0.14010437	2.08363548	0.48498782
Igfbp7	52.6316934	29.54924	34.3990293	74.8742586	29.3382647
Prps1	9.47633159	7.64828375	23.0838792	28.6768729	13.2561905
Olfml3	11.5698159	7.55091046	8.66120998	25.1411745	10.3551605
Oas3	2.32207797	0.41241527	1.40787368	3.00744826	0.45412356
Napsa	4.27689918	1.35022302	2.98046226	10.9700567	1.30731927
Dancr	14.9181182	7.90804811	12.3567724	28.2012151	7.86888058
Lysmd2	64.7769449	72.0278202	54.0948797	79.5270414	30.6141489
C1qc	45.5986861	38.2334244	26.812307	45.7259803	8.0815582
Rbks	2.35398294	2.09814018	1.64954097	2.97888725	1.12162305
Itfg3	14.8280859	31.5490516	20.0791138	35.3614459	14.7116643
Ropn1l	1.21824035	0.84403206	0.39814274	1.51319257	0.05742576
Emr1	2.15027759	2.52472661	2.47494688	3.50547323	0.40259967
Fbxo21	5.05478177	2.46264184	1.86916931	7.48946755	1.69779856
Tfeb	2.46821747	1.34034003	1.70880742	2.96489954	1.1275924
Trim11	7.67366684	11.9598693	8.82178468	22.7834869	7.1471096
Pald1	1.19010571	0.88655621	1.8084412	1.28872741	0.58688746
Hoxd10	6.09817421	6.55972591	8.35597589	15.3396118	4.0393587
Glb1	13.0534236	10.7719009	8.1608263	20.4315527	10.3715357
Nudt6	7.66480679	5.30197004	2.92912226	6.86938854	2.4861281
Tmem41a	23.4646918	21.4765479	11.8637161	38.9880549	11.1128767
Rbbp4	101.524257	126.994873	132.074164	129.466559	57.3088714
Slc25a23	2.38579352	4.31020377	1.92183018	4.39852434	1.91185748
Znrf3	7.54908553	4.26299097	3.15043753	7.46236237	2.40321233
Sfmbt1	2.4087056	2.52177353	2.80289808	2.99275251	1.22247146
Cdc42ep4	26.3551836	33.1350731	40.3818976	62.8322388	34.9002265
Actr3b	0.15245281	0.83794806	0.07058443	1.57460004	0.64138293
C87436	5.86946981	5.42880411	6.266731	8.20764616	3.36442824
Tango6	0.5916234	1.53300266	0.76696734	1.07274067	0.49780276
Arhgap44	7.73128278	8.0313566	23.6559723	12.8145457	8.10670118
Elac2	2.57939081	1.17838732	1.62850545	2.21739973	1.20261633
A230056P14Rik	1.6709045	0.61955758	0.68765816	1.10791076	0.37193906
Acaa2	27.3454351	15.2589742	14.4923957	22.7904345	5.43477352
Casp12	12.0910733	5.85757168	8.99400116	21.5347393	4.05220822
Wdr70	1.24299362	1.809182	1.99318165	1.92050939	1.19042978
Phka1	2.67671857	1.77520485	1.68860702	6.2758033	1.86297979
Ttc38	6.0326463	6.25212266	5.40905198	10.231015	1.85290302
Mast3	2.17616812	2.38071685	1.49434151	3.12032291	1.48915083
Ankrd33b	1.59237847	1.17889687	0.22468831	1.49674482	0.30989876
Mr1	5.69205261	4.67351775	3.2009576	5.65529729	1.93700347
Thra	4.99394951	2.39380419	3.33381023	8.95647835	1.76828373
Zer1	2.70389932	4.32116966	1.7231812	6.74630348	3.84920639
Hsd17b11	5.87250647	4.43029894	5.15351524	6.37812389	2.12228558
Vps37c	25.8686537	28.0300192	13.3021989	28.8564964	11.3386739
Ppp1r26	1.16918141	0.59954174	0.28723189	1.77440659	0.61186901
Mtg1	6.58249656	4.26972623	5.4516849	11.6361353	3.47290809
Acadm	16.8879933	10.8518018	9.8396479	25.3183605	14.0147251
Fam49a	0.49769648	0.54922438	0.06227824	2.16114145	0.31259606
Vars2	3.28711726	1.5625277	1.91091715	4.73653424	1.7954635
Cpsf1	2.88802877	2.21100515	2.79461055	6.31045646	2.77718889
Dennd1a	2.53234732	5.1534053	2.27553701	4.48473172	2.01729079
Dip2b	6.20241381	4.74587594	3.46465747	12.0690843	4.08810806
Zfp239	1.83139832	0.70991107	0.42932799	1.46854451	0.11146272
Ccdc64b	2.51075183	1.83825088	2.79618283	6.91524141	2.39264023
Pwwp2b	13.5610673	11.7925772	8.94338181	28.2794059	12.6684697
Sgcb	0.48991878	1.26397217	1.62020344	3.18063294	1.64049375
Abcg1	1.41228479	0.88010676	0.43591771	1.88315092	0.23353272
Alg2	6.85203023	10.6934672	9.94227364	14.8351663	9.95542676
Msantd2	9.93403738	4.49241681	6.43122463	10.4530411	6.18147779
Lrrn3	0.92630241	2.44385646	4.01422892	11.9229332	0
Fgfr1op	10.544693	6.73744889	6.18400419	16.1728166	7.17421386
Fam63a	15.0488514	18.0137607	14.9364405	29.0053724	9.58852068
Ppip5k1	3.59601615	1.39792015	2.30360355	4.54625076	1.71955409
Anks6	1.28514028	0.82169562	1.68302737	1.65181298	0.52963619
Plk1s1	5.06528007	5.9302085	4.83079856	11.0828733	3.13544626
Tmem26	0.86484228	1.22639707	1.00402583	2.34644081	0.16550257
Nmnat3	1.60010164	1.32752697	1.76122864	4.60110445	1.54836852
Nnt	7.49638701	5.83220633	6.71227284	9.54683893	2.98708408
Fbxl17	2.0997642	2.04360293	1.76306517	2.611462	1.21507463
Nprl3	3.71672842	5.9915586	4.09345517	8.06536069	3.49736738
Glud1	35.2903032	20.9812436	22.2571625	56.8933655	23.2163347
Il15ra	2.51682919	0.8107608	1.94211966	1.92554999	0.46542993
Ift57	3.62001899	1.04828339	1.47396413	5.798413	1.93393667
Zfp592	4.14950959	5.21034308	5.73872635	8.12823572	5.10964798
Ccdc117	16.2022575	25.0527708	30.0770831	29.8556803	21.8187479
Zfp184	5.8866638	1.72386964	2.01059874	9.39134004	0.98598939
Tgfbr3	0.57099282	0.8628565	2.95290842	4.65862752	0.41439898
Scmh1	27.9176738	43.6461229	34.9606629	55.1952215	17.8605267
Snord53	0.60381194	1.42235032	1.67736061	4.9891494	0
Fbxl3	59.5377832	83.5077084	175.051305	206.076208	84.1094735
Tmprss2	60.2604698	67.8084479	63.0785228	179.912421	31.3280115
Nek8	4.19218004	4.90101282	2.88985271	4.74681929	2.18361436
Mir27b	0	0	2.4815746	4.10067074	0
AW046200	0.95771507	1.23055141	1.93489929	1.39882693	0.66281129
Ncam1	2.71110216	3.89913152	2.54245815	4.13354917	0.48771392
Cd55	57.3119362	71.3540113	75.3975587	344.926799	27.8871837
Snhg7	11.8646605	13.4567375	12.0444439	24.4481768	13.1700011
Fuz	2.61153998	4.61384309	3.80517849	5.21833294	1.26181092
Nxpe3	4.76663983	5.57437451	4.37626775	5.50280677	2.38124872
Abhd14a	6.18465117	6.4273571	4.96891763	11.648307	3.97214833
Vwa2	14.6650708	6.33132755	8.18199396	22.4858727	2.07306682
1500009L16Rik	1.42002004	1.15789323	0.20229475	2.50711025	0.43766698
Rcn2	67.8623116	30.4920703	42.4942308	81.5366767	46.6273514
Calcrl	7.93115143	4.04360741	8.42788706	14.398584	0.98203377
Zranb3	2.13499995	4.27732311	6.49785435	4.5831955	1.24542281
Ophn1	1.55897732	2.17476351	1.42823076	4.15677859	2.29922094
Slc25a17	91.0303549	89.7846908	155.110074	125.05496	58.7778618
Bbs12	1.0180954	0.86252632	1.93509803	2.38593669	1.30965641
Prnd	1.74472572	1.53114199	1.59658121	2.73248976	0.60860094
Dkc1	19.6588932	9.44444672	14.7138576	26.5921841	15.1570806
Gm11944	2.10118217	2.23909694	1.73719741	3.78922739	0.60135141
Rcor3	3.15012553	2.82685434	3.29579262	6.23487177	3.01611309
Insr	1.84725791	2.36455288	3.00149831	4.64622871	2.04449195
Pms2	4.35685894	3.64276544	2.33471165	3.79625967	2.44072784
Snora44	0.55736487	0	0	3.07024578	0
Npff	1.36671206	2.96393959	2.04899141	2.50951227	1.56459394
Urod	12.1830012	14.2638367	12.8928736	20.3727068	6.62479611
Tcp11l2	14.1563709	30.9361195	27.7267709	61.0921344	19.5094434
Lpin3	3.4799312	3.60452559	2.39963028	7.17145971	2.92214454
Igf2r	5.92302388	2.60926176	2.77950808	9.17256989	3.6285823
Lrrc16a	6.89033943	7.76308767	3.92186293	7.0687677	4.36660637
Pcdhb9	0.78979788	0.43578393	1.73940374	1.64617434	0.58158851
Taf1a	5.64096103	3.10266138	4.36548345	9.13183216	3.18829332
Slc27a1	5.57624107	8.77531625	6.13571911	7.60421339	3.14105529
Gm13580	0	2.00802398	7.64230611	2.34783501	0.65205359
Abtb1	11.0304879	16.3418973	9.86073909	22.4830179	8.8670865
Fgfbp3	3.63937675	1.57462928	3.19806568	3.10259177	1.24989316
Fam214a	2.62519962	2.04999661	1.01509813	2.38366793	1.0171764
G0s2	2.3207007	3.34075164	3.58155291	3.55099601	1.17780702
Pigg	2.76396278	2.26829288	1.28794876	2.60303447	1.30036282
Rgl3	14.0908396	11.2047738	6.81995091	16.1061482	3.6682672
Malt1	2.65638671	2.12670911	1.91717495	2.98870771	1.33565336
Slc17a5	3.62175382	3.39678849	6.22295094	9.97522003	3.12802929
Rad52	9.19821101	11.5456303	14.5314719	29.8743607	11.8094412
Nkg7	5.6949938	8.81752228	6.31331301	10.6778843	1.41410041
Zc3h8	4.03671594	2.02596421	2.31212439	4.9668622	2.03427167
Prkacb	10.9220917	7.42723848	5.90658061	10.0627442	3.56885079
Patz1	15.9189937	15.2937381	8.41088923	16.0420783	3.73696209
Smyd3	0.88428508	1.02109701	1.71794551	2.91310079	1.22272122
Gm5577	0.91653815	0.07196713	0.63652476	2.10364697	0.6610217
Xrcc1	4.29701156	5.13234157	4.51136061	7.55665134	2.37644032
Mbnl1	26.9535663	15.7430601	16.7672536	26.0133168	8.95996005
L1cam	2.51573084	0.76497056	0.53670464	4.1324649	1.06728504
Zfp346	3.51711642	1.25271221	1.49409603	3.64511666	1.5545026
Eno2	0.24785895	0.17515831	0.27541611	2.04799747	0.15889757
Slc16a2	0.65886239	1.52739279	2.3241754	2.34755262	1.40794636
Vegfc	0.38135491	0.95277055	0.89887267	1.55960651	0.30559905
Dirc2	6.80183411	6.98244703	5.91385639	13.7645308	6.38013513
Npc1	9.07630542	10.84252	17.5393507	29.5498632	11.3463548
Ppm1f	1.0046804	1.5777622	1.95856419	3.10696675	1.34536683
Rpf2	13.7669122	6.77309676	13.7703319	18.2460321	10.9491774
Gtdc1	3.44697654	5.45327108	4.35036676	5.76662844	4.11266444
Osbpl7	0.90392441	2.31760618	1.86194146	2.62512529	1.07914935
Tpk1	2.95259797	2.75809383	1.72057131	3.06125795	0.81588505
Hebp1	4.60968588	2.8956425	4.89454143	9.93127571	1.92086717
H2afy2	5.70175505	3.7800263	2.75052012	5.86159751	0.9028772
Mir5115	0	0	0	2.4945747	0
Lancl1	4.41468959	3.61565851	2.30115377	4.26778617	1.51114312
Smap2	5.2528521	8.19626312	7.2337259	13.0456382	7.33942671
Dkk3	2.622454	1.06771606	2.23048489	4.49424718	0.65380172
Anapc2	19.3019684	22.6406867	16.7324644	21.6896531	11.3452508
Mtss1l	1.50946574	0.28271818	0.3846994	1.65280804	0.4771862
Efha2	4.05078732	3.38439293	4.39768229	9.01344494	3.46997062
Dnajc18	6.17157271	6.77839255	6.12381084	13.1511931	6.51321654
Mettl7a1	4.05192123	10.1702202	11.6729333	25.4995081	2.02591235
Akap1	4.33521843	2.02933132	3.18059481	8.25104022	2.40509813
Mir3470a	0	4.04246933	0	3.15104173	0
Nsun3	0.53372663	1.56204544	1.16816186	1.24728735	0.77764044
Smc2	28.8250129	11.0665858	16.0466763	21.0855087	7.25770465
Card10	1.71028966	1.26711919	0.3448381	1.31693284	0.76264027
Hook2	7.15160676	8.62867164	10.8217641	14.5728154	8.22700204
Tmem109	32.453718	31.3757651	29.6133712	68.9014723	27.4696951
Cadm4	1.4827716	0.42769513	1.48510629	1.94472645	0.92147686
Fam73a	2.93537863	2.25705796	3.71061993	7.69339138	3.21118734
Zbtb20	6.04350371	7.12624689	8.9231311	13.8806399	3.74456162
Slc23a1	39.7685112	48.022404	24.9246087	54.7164939	11.7227552
Hddc3	5.00461803	4.82275992	5.47677744	6.26544343	0.72917355
1810043G02Rik	5.80599359	3.35124521	4.27252231	5.08328429	1.38654758
Kcnb2	0.95809226	1.39850606	1.56962281	1.92837344	0.88588612
Slc9a8	4.35226575	3.97375034	4.44519158	7.92511448	3.49927853
Fitm2	14.4870765	11.1260172	11.9814389	24.5965369	9.28738394
Gpatch1	2.36784349	1.85924497	1.91604294	2.56556848	1.41883132
Trap1	8.28986214	5.88507507	7.28195038	14.8045461	5.41457126
Zfp27	3.64032384	1.36390861	2.78910515	3.78321445	0.75520808
C2cd3	4.22390104	2.1433574	2.58174398	3.34142725	1.32449727
Fxyd6	5.34939641	7.40066651	10.3786688	13.9985732	5.4532036
Mri1	8.37525654	8.29185892	9.66609298	13.4560772	5.34976096
Mfsd1	45.4379637	35.62658	19.1212351	53.4365041	24.1984576
Foxk1	2.37340423	2.42063165	2.47346488	3.11168544	1.17203762
Mospd3	15.3843057	18.5160948	12.6752377	19.9442406	7.05394515
Epdr1	0.68298795	2.23095082	3.06098987	2.33258933	0.70056054
Ss18l1	1.80604588	3.70351505	2.90245965	3.34360939	2.51180177
Akap13	5.05347702	7.05017652	5.5074878	9.59883228	5.41614199
Apoa1bp	60.4559199	56.806	41.2352695	62.9790527	23.09721
Rarres2	2.21056575	2.74827011	3.24100186	3.63615973	0.14761988
Ccl12	0.7299816	0.28659298	1.46456113	2.90413174	0
Wnt5a	48.2861767	59.2605852	53.0940444	87.2787398	18.0684303
Pdzrn3	0.77822036	1.43412819	1.85302186	4.92839502	0.62362748
Slc38a6	0.85949844	1.38437611	1.53054196	1.98284545	0.17660506
Pus7	5.26186797	3.07939489	5.730095	9.4445548	3.8849591
Nr2c1	10.2580186	6.66315562	5.41208488	9.05790698	2.74464201
Zfp362	6.76554309	3.55340816	3.41525055	8.60031027	5.05892613
Aldh5a1	1.28183063	0.46244647	0.40634484	1.52669562	0.62001918
Ndufb8	170.811898	120.622408	219.119295	180.076381	63.3448192
Foxl2	1.57853693	1.889694	2.06075732	2.51833255	1.11980224
Tmem198b	1.89909845	4.27618812	2.63780221	3.12810052	1.40994372
Sdpr	4.01302705	4.64277492	1.97659516	7.251006	0.8894883
Zcchc11	6.87232539	4.33894379	4.17927114	5.81490554	2.29608167
Spata13	3.86915173	4.16203507	11.0937422	5.75043108	3.97648721
Mzt2	10.0723006	6.13441386	8.37019553	12.0332356	3.8805028
Tshz1	5.58572779	3.84871263	11.2793413	11.5950098	7.01978996
Rbm18	26.8985423	30.4253461	46.7580728	62.501432	36.0551709
Tmed3	70.6295792	94.7130617	230.36597	245.900252	123.341762
C1ra	1.72355298	0.94548703	1.09313871	2.53612084	1.17304893
Zfp691	1.14406473	1.81804176	0.52969283	2.12570275	0.98228266
Nr0b2	1.45822204	1.00760416	1.51232513	2.94529392	0.32719326
Nog	3.15540433	4.0494802	3.61304523	5.63810223	0.21751275
Znhit6	2.59641281	1.92950189	3.19849354	4.57589956	1.46759246
Ednrb	3.05469223	4.47966866	4.46474494	6.94242144	2.43588056
D030028A08Rik	1.66497931	0.74446421	2.14131142	3.29071556	1.00067434
Arid3b	2.1846174	4.21414898	2.64413269	3.86918998	2.90855166
Ugt1a6a	1.18494893	0.72883608	2.57852286	2.13950198	0.28486792
Tnfsf10	3.57450806	2.91029447	2.79695811	6.29698468	0.47564415
Psmg1	22.1123902	15.5433288	19.6787606	21.0304044	9.44347605
Peg13	0.92469486	2.00483664	1.13741024	1.55851524	0.38709213
Angptl4	1.88473091	2.77145296	2.1260083	3.86967132	1.09842223
Srgap2	1.69517949	1.40112121	0.95384238	2.6732905	1.44942893
Hoxb6	95.2031474	115.578337	144.796061	221.545406	104.000997
Gin1	8.24331989	9.90976068	15.8580845	15.1654497	2.89323393
Ocrl	4.73168569	8.49787069	3.56576698	6.34282444	3.36819456
Ramp2	7.10448453	4.52150813	6.09389727	11.1912571	3.71555176
Atp5s	18.0924014	21.6197249	26.8377698	23.5850699	9.99401502
Rilpl1	3.2765322	3.84774538	2.01372773	3.9664736	2.25387793
Myo19	1.52891831	0.70465062	1.29264488	2.53271804	1.34505637
Polh	1.71875771	0.63509596	1.21706165	2.13489184	0.4861157
Sptbn2	2.15392587	1.85294162	2.05316272	2.8935249	0.75514694
Gal3st1	25.8548549	12.9076725	15.3105339	44.4554457	13.6846447
Copz2	6.66857966	9.14958613	12.0899964	22.5558961	6.86265597
Pnpo	13.836665	17.5041305	27.2562861	20.4074834	12.3456913
Cd83	4.92507865	9.83510487	17.0598131	13.9277513	5.31101232
Arhgef10	3.55007006	3.27395214	1.55540418	3.57643812	0.63006775
Txnip	36.0165954	47.7129725	25.8484804	83.2106759	7.89178727
Dolk	5.52121548	6.33434282	9.25940929	14.2211878	8.55389855
Kctd1	26.1610319	19.6281965	19.9655948	31.2117403	3.86066936
Csrnp2	2.07046324	1.7002016	2.19528534	2.53931501	1.15255134
Slc48a1	42.7257903	23.0481565	19.3633553	50.0574039	9.26814414
Mapre2	2.75415167	1.67806481	1.32392645	2.8877883	0.96482691
Skap1	1.72706758	2.0575369	1.15806815	2.65176708	1.62260537
Prune	14.1015087	22.4224016	18.7693447	20.9162939	7.88854314
D630045J12Rik	1.40885783	2.52522287	1.66788054	2.22124026	0.69073186
Agap3	7.34935425	4.35599095	5.47357811	10.2588327	4.66086696
Fam212a	0.18438744	0.82043204	0.51221945	2.14425271	0.68954206
Gdf10	7.17354576	5.26536061	5.65582733	13.6252099	0.3749039
Ifit3	7.73532053	9.91801034	9.48993211	45.7563431	0.31385849
Mir31	1.23040924	0	2.27867857	1.12961873	0.4929947
Idh2	24.3298607	8.00260086	8.30204806	46.8194443	6.50915205
Slc12a9	2.14421543	2.42507512	1.712274	3.35921009	1.10347531
Ccdc171	0.21617135	1.04106614	1.18768252	1.56124543	0.73911072
Car8	0.43307309	0.78748791	0.84425001	2.05076536	0.40183979
Cnrip1	1.65253794	3.59330607	3.17815695	5.7963607	2.64852511
BC005561	11.2399226	9.73490491	18.7530276	14.7912246	10.247743
Spata6	26.4003025	23.3760183	20.1118152	29.8121118	8.7452972
Gbp7	9.17333025	7.98688645	12.3880574	11.7431924	3.4825742
Zcchc24	2.36997912	2.32907833	2.26357418	3.13375601	0.69278432
Mapk15	4.15597814	4.53678164	2.19012888	10.6090514	3.41162547
2610019E17Rik	23.2109403	13.8859964	29.0666693	24.5567625	11.3372068
Ephx1	10.3398715	5.32035904	4.89607963	11.1109706	1.75760751
Zfp692	5.62112049	4.88663815	3.00530696	5.86717826	1.74280834
Gm15706	2.06086604	1.96496252	0.74970068	4.05438777	1.53351397
Ndufa12	201.795888	113.727176	148.002407	245.668918	86.3505254
Mapre3	5.26894029	5.29239689	5.57594797	10.9311059	5.04478937
Xpc	8.72601352	6.69663835	8.32953634	13.1047592	4.56101535
0610011F06Rik	31.6007324	26.896627	22.7095185	50.8634861	16.8177451
Cdh11	19.1335057	18.8495154	24.9503413	27.7622023	7.90802781
Clip3	0.31617789	0.99305913	0.64044678	1.68723962	0.57008114
Col18a1	8.34430357	6.78189208	7.4660071	8.94273368	2.43645453
Nt5c3l	21.6084166	9.70991152	12.5541123	23.8592211	6.6837908
Prmt3	4.155775	3.48889137	6.65565934	11.6380158	4.98487782
Cx3cr1	1.68546067	1.14606645	0.62750181	1.56334657	0.11139342
Dcaf8	25.4714107	22.6268842	23.376393	27.5116333	9.8148487
Naalad2	1.52726262	1.61798958	1.25708513	1.75825786	0.17483901
Hyi	1.75867555	1.21521192	1.17252392	4.00423641	1.29657073
Usp20	2.87075756	4.80389668	3.06500631	3.88858409	2.23762904
Nrp	2.73108785	3.50117002	4.79983191	4.86122249	2.41188174
Cpsf4l	1.24212742	3.51117336	4.52408119	12.8862602	2.12347683
Glmn	5.10000815	3.58945059	7.08378902	6.22394603	3.16485197
Dpp4	16.1347443	6.54989952	5.57753683	23.0699662	0.97863476
Tcte2	0.92630241	1.60014411	1.75836951	1.82840987	0.5196052
Msantd4	61.8522273	76.8162714	116.578095	96.9028798	22.281487
Ccs	17.3980099	12.4014953	17.7098812	18.9181748	6.427121
Sfi1	4.81189361	9.26135582	7.02116064	13.6993986	2.90824001
Palld	3.36482077	2.36925432	1.45628392	4.13010909	1.97833822
Sdc3	1.63947329	1.05045663	0.25504518	2.03499497	0.88286902
Traf3ip1	4.58345018	2.1359638	2.34638787	3.52376495	1.91112915
Sgsm1	1.35732009	1.58682191	0.37240199	1.43997652	0.64455674
AA543186	0.96125722	1.05669894	1.24615234	1.55322576	0.15406084
Tmem104	1.07115127	1.61486291	1.8382638	1.56033845	0.98426186
Zc3hav1	7.70830709	5.65988262	6.47030599	10.9259333	2.53447983
Mir484	0	0	0.90126839	1.78715799	0
D10Jhu81e	15.6450642	16.3043297	15.0187153	24.3783822	9.00990305
Bcl2a1d	1.06501503	1.47953023	1.5172106	1.72990607	0.32825024
Dhx57	2.8386416	1.18001567	1.96248218	2.19337827	1.56453231
Cyp1b1	0.50867153	2.39647168	2.92033455	3.18729591	0.24457459
Tbc1d2b	2.78034335	1.69991717	3.09353464	3.74312321	1.32977302
Cep68	3.26736324	1.73520825	2.52336411	3.2610989	1.01038289
Hoxa11as	9.32608719	14.2281438	15.4656479	19.3054851	5.20016917
Ccl4	1.58088944	2.71539607	1.73835554	4.80772578	0.31671174
Fam107b	123.71163	110.704761	61.2865367	111.314453	49.4520035
Atm	2.69807642	0.98865474	1.3526559	1.99165643	0.59403306
Mir1839	2.67993245	2.80573214	7.44472381	7.38120733	2.86342124
Tmem161a	7.97176966	12.1085053	9.58011572	12.2579101	4.92204323
Acadvl	10.1036393	11.6544317	10.4873369	19.8106627	11.4880382
Snora81	3.97632253	2.18556269	1.84100555	9.85661223	1.59321456
Asf1a	87.2677055	54.4665002	81.915466	156.341925	75.9912236
Mcam	1.10490069	0.90298589	4.09248149	2.50492563	1.13838817
Tspyl3	0.4670108	0.60005404	1.06145476	2.18275286	0.7825007
Aldh4a1	4.43167369	5.6012198	4.139656	8.35143458	2.39870206
Psd	1.43800136	0.95844529	1.54833287	1.47371798	0.80396058
Cask	6.90969101	4.0613975	3.23203706	7.77915811	4.77771439
Mgll	10.9061901	6.25121122	5.91063026	9.77994022	0.93720124
Hoxd8	1.00748397	2.76878869	6.61520871	3.86799223	2.67892764
Fam149a	1.17830941	0.64484727	1.25641321	1.60628939	0.46735069
Rufy2	1.94846924	1.1345695	1.50692155	3.24272378	1.14620163
Pgf	8.64578286	11.2942395	23.550861	41.8590228	2.42335323
2510002D24Rik	98.8232341	92.5589853	88.2248114	121.003071	42.1622509
Wnt5b	1.20398372	0.81032166	0.61431594	1.94001589	0.33473114
Mir106b	0	0	0	2.19035827	0
Tbc1d5	2.27712296	2.55430095	2.26995033	2.98655656	1.12651879
Mfap2	5.03830437	5.21486825	6.73300502	11.0908923	2.20224496
Exd1	0.58095046	0.76570689	1.53700241	1.33340296	0.6151846
Amigo1	0.99807197	0.91600378	0.46810064	2.4276362	0.7582574
Milr1	0.73804661	0.28975932	1.48887789	1.74237069	0.59143422
Angpt2	0.29308625	1.29449861	0.52582428	1.71537047	0.32293922
Me3	1.11919376	1.35920708	5.67922829	5.89107801	1.80569178
Cited4	105.800523	467.069092	528.536596	305.535826	164.831091
Letm2	1.11791468	2.12133391	1.09268063	2.36628229	0.64699685
Fcer1g	35.5790221	28.6372296	22.3002289	39.5795743	12.8658129
Bcas3	2.7112081	3.03922339	1.86638484	4.66709271	2.75866671
1700029J07Rik	0.52359751	1.04651761	1.03580079	1.66062199	0.4958735
Mir680‐2	0.70882271	0	1.9690755	1.30151723	0
Cxcr3	0.89219973	0.92346625	0.18776425	2.15948258	0.94245379
Entpd5	10.5331907	6.39288036	5.32452747	16.593171	5.48755458
Klrb1c	0.39642365	1.73146293	1.67481143	1.34206602	0.0397093
Cdc14a	9.9453279	15.8466	10.4344172	14.9374132	4.34604777
Trmt5	9.377355	7.63927398	8.10439543	10.2951651	3.31892654
Ctns	5.06961307	3.58942626	4.05241408	7.71743508	2.89933292
Itgb5	25.7111293	28.9098553	17.7548774	49.9827033	10.3336615
Dopey2	2.41427583	1.75833408	3.30477165	3.95919622	1.91365265
Mir677	0.8360473	0	0	3.83780723	0
Echdc2	21.8536496	11.0479728	10.1642663	22.1247972	8.0365302
4930430F08Rik	3.34280674	1.92625864	2.12183691	3.04422675	1.44739493
C1qtnf1	1.96255583	2.82140922	4.30271392	6.26652179	2.06994498
Asb7	6.30831181	4.96345394	3.23725621	9.52231719	4.11443328
Cul9	1.23872281	1.80821996	1.23171815	1.99973046	0.32644243
Reck	1.77997168	2.18309758	2.24757998	2.64707408	0.10609451
Csf1r	1.63239584	1.95568581	1.74532077	2.62654575	0.49897424
Gtf3c3	4.38361441	5.91281205	4.94332964	8.46744158	4.83446512
C2cd2	2.44693039	1.78856865	1.86976073	3.99071072	1.99273329
Ube2cbp	1.67666817	1.31652995	1.05706752	2.81663139	1.54370987
Dimt1	6.09838197	2.47472189	6.00290784	4.84604217	3.59334051
Esyt2	7.23789732	10.3314845	12.783177	12.3067486	9.87430739
Snora78	2.90752756	2.60915218	1.15385316	9.15207023	1.33139969
Ndn	4.22631786	4.93127392	6.54688782	6.20092948	2.75372325
Tdp1	3.01465231	1.29568847	0.88153258	2.62203472	1.04260581
Zfp11	0.98250503	1.62139219	1.11024482	1.66644725	0.74729737
Gdpgp1	2.44543836	3.28756881	2.71046226	3.58878587	2.12295841
Irak1bp1	2.99705071	1.32542312	4.88056115	3.51059007	0.33126743
Echs1	128.805115	89.9085667	80.1083129	178.16383	64.1389074
Hoxa10	15.3170678	22.5710971	26.3487621	27.7626633	12.4240312
Gm14446	5.27185027	4.2041681	1.42381431	20.2927087	0
Hap1	1.98837586	1.16060102	1.41756434	2.22936627	0.11280613
Mthfsd	6.69727253	5.07017839	4.5708763	12.9097303	5.49515609
Adcy6	6.11528707	4.97173407	3.84860301	9.42017652	3.06172208
Marveld1	4.51416635	1.48105636	3.86605182	9.41728297	2.98905071
Csf1	1.10603273	0.74108891	1.05147979	5.2396223	0.40179848
Ndor1	5.43430746	15.4123333	15.146316	12.9693063	9.07788822
B930092H01Rik	0.02435089	0.63097542	3.40482908	1.2966572	0.72200343
Il2rb	0.84659093	4.10244662	3.20291262	4.06386205	1.14361603
9‐Mar	2.12150141	3.20349171	2.32714896	3.74560766	2.61548737
Htra3	1.23005218	0.62408406	0.47312667	1.77211807	0.84922127
Snx2	58.3438911	58.8611381	80.5433069	127.859036	28.5300169
Mir130a	0	0	1.88703069	0.93546551	0
2700046A07Rik	1.86493814	1.94447892	3.00084768	9.48710265	1.76396414
BC030500	0.40630336	0.31903185	0.22573825	1.52938412	0
Snord118	1.48208385	3.49122351	12.3514736	12.246094	3.56300712
Vkorc1	60.5038938	62.0205589	65.9552544	80.0398299	18.9328417
Tcta	20.1359606	35.0165361	28.7282757	32.5626988	24.5728651
Bcl2a1a	1.80233313	0.38596441	1.06204742	2.25639923	0.1313001
Ebf4	0.68386791	1.32326524	2.71393178	4.70886011	1.52662178
Frem1	0.12251439	0.15498734	0.43487775	1.82465082	0.34361952
Slc22a17	3.07012313	4.30797298	2.86920795	4.8282091	1.07066241
Rnf215	5.490447	7.6436636	4.27311082	8.56608089	6.15428502
Rwdd2a	0.70728514	1.25882631	0.87324631	3.46318396	1.39806594
Gm14378	0.97059259	1.57186249	0.61789284	2.72894869	1.06945454
Zc3hav1l	1.00739992	0.47774263	0.80353219	2.13362828	0.76731631
2900005J15Rik	0.86606769	1.61195039	1.39601287	2.53261298	0.30845512
Aasdhppt	47.5568175	51.882582	83.8025726	68.5269231	15.1491945
Wbp5	970.039897	458.678637	467.064193	1186.98628	670.928007
Gria4	1.06000796	1.04040579	1.29564656	1.80037576	0.06795506
Scai	2.44300145	1.97088004	2.2283322	2.91965918	1.45973224
C4bp	1.03895416	0.32631723	2.18065788	3.27487449	0.55504448
Rorc	4.87198799	2.35258902	1.49575265	9.99823148	1.5449662
Arhgap24	0.85753567	0.55091632	0.34289141	1.89665213	0.21865037
6‐Sep	2.27856284	0.94044605	0.39673573	2.18130946	0.49159602
Foxn3	23.9517466	35.8295003	24.4453949	55.5360544	23.9171345
Gas6	4.14960616	3.35703199	3.84246897	5.42591643	1.28980949
R3hdm1	8.52413814	10.1621787	13.6531298	21.0265897	6.80910504
BC048403	2.00844472	1.13349958	2.09226117	3.01557827	1.27416274
Mpdz	1.53592403	0.48376945	0.94012547	1.02770503	0.41722505
Dffb	5.63597122	2.07518689	3.42024312	5.81742615	2.67921434
Slamf9	4.95494934	2.32544961	2.05678105	4.75821061	1.36919051
Pigh	19.1864325	24.9274151	27.702124	28.0512379	11.9090079
Tmem220	0.68224959	0.22321104	0.68439823	2.19226791	0.77452174
Eif4e3	1.97218003	1.71157476	2.42693322	5.19363901	2.47458619
Smim4	29.4504404	16.7717338	12.8861252	21.6898009	7.00223731
Ica1	5.49978105	3.01058439	2.35719689	8.62702074	1.18366244
Trp53inp1	17.461841	16.9550445	19.783588	20.3755759	5.81279786
Slc25a26	3.941926	4.42937234	1.63627883	3.61901822	2.75039146
Ssh2	1.39239474	2.26594143	1.16240589	2.05328419	1.08110862
Mrrf	15.4479247	16.1578996	21.9532957	30.8662043	18.7197755
1110051M20Rik	4.81096851	3.49298765	3.32590351	5.68747905	1.2938931
L2hgdh	9.80715043	6.68819971	10.4922977	19.7832179	6.74744028
Tmem120b	2.17372298	2.47764249	8.01886589	5.02133746	2.61287189
Sil1	8.55927414	7.50438391	7.0727554	13.2848216	3.9677513
Mex3a	3.10478093	1.64890196	2.7599784	3.09921538	1.13996488
Tbc1d8	3.12910337	4.54485626	2.9927227	8.7666964	1.96598155
Fam21	6.76853934	6.5897487	7.47610204	16.900642	8.70754605
Nfia	5.08547034	3.9825219	5.21002852	7.3758492	0.70991645
Angptl2	1.43905006	1.32323183	1.61307153	2.67710456	0.53107152
Gtdc2	2.75270956	7.21922341	10.6801636	10.9175815	6.04412745
Gmip	1.89404652	2.2699624	2.4461687	2.53207277	1.47785365
Tmem38b	9.29967471	7.32005806	7.3449751	17.7035459	5.6622879
Gpd1l	11.1630299	8.78859028	8.12372511	12.8516309	1.84382036
Ppip5k2	10.8861922	9.39441301	9.66810764	22.2373516	4.56372232
Tmem206	1.76834743	1.33979716	2.11146345	3.1615352	1.55380107
Atp6v1a	116.045738	138.042235	157.695702	292.961437	153.156935
Mocs1	4.39019695	5.42257611	4.49918361	10.7104433	4.9806635
Msrb2	10.4032082	6.92186604	9.53180237	23.6764672	3.42240146
Sfxn3	10.3116259	12.6889378	16.7351501	41.2185155	12.8617429
Klhl17	3.80798187	5.91214493	3.12543371	10.4086833	2.70476453
Clec4n	0.57616754	1.27497426	0.48501994	1.58691017	0.20986923
A630007B06Rik	10.371358	11.3416169	13.5723689	18.0435468	7.30541522
Mettl1	8.38120925	2.71320941	4.0846662	8.70710628	4.1240368
Ccpg1	10.3183053	12.5122991	12.3628032	20.8407507	12.007419
Mmp23	9.86815665	4.32061227	3.32664824	10.1035494	2.66024613
Zfp397	9.53331663	7.50428503	8.61856379	9.6036124	4.31509648
Arrdc2	2.41833631	1.13497008	3.03726679	8.472622	1.33650736
Il17rc	13.3506414	7.69474112	4.97048185	11.5999303	3.26471118
Slc35d2	8.4236541	8.0316548	12.6288451	10.8638947	4.31651946
Mir3091	0	0	2.38361771	4.72656259	0
Dtx3	8.82236335	6.69031447	5.71545098	13.6123706	4.19349809
Scel	1.21751271	0.53670081	0.1582312	3.7651491	0.616203
Selm	5.16009929	4.58022901	6.1631461	12.1524694	2.99641271
Snord95	1.91799087	2.25902698	1.77602888	4.40219065	0.76849173
Tmed6	3.92932544	2.61066386	3.14869547	16.5803945	1.45327753
Fam13b	2.08661806	2.84810339	2.25721374	3.63444017	2.34408363
Def6	1.23148997	0.33731628	0.4508321	2.28751514	0.09180051
Eif4enif1	30.5862865	28.8222648	25.7671141	40.3944181	15.1707171
Vps13c	1.11487485	1.05759764	0.7808177	3.174559	1.2471401
Pvt1	0.95462786	0.69960758	0.86432635	2.80457064	0.61199342
Tfpi	2.13921945	2.26221432	4.47296163	5.65436932	3.02761345
Gzmb	7.27644558	11.2100491	8.40101798	14.7560436	2.91549264
Adal	4.13729752	5.30388725	4.1698736	8.8887392	4.96186288
Pcgf2	2.43739037	1.87774316	1.72467685	4.11657602	1.7505136
Osbpl1a	2.44574561	1.64055484	1.9688281	3.83636064	1.73337901
Polr1a	2.43382963	1.07287449	1.91761115	3.90050377	1.86480953
Pcca	1.80378089	1.71141037	2.76058888	2.5300335	0.58219965
Fbxl4	5.91876972	7.29133839	7.91654666	12.9448201	7.12505605
Nkd2	3.66335065	2.63810227	4.34801857	14.5865262	0.79472826
Mbtps1	27.7604403	28.9503897	26.3407741	34.7118872	11.1946089
Slc30a5	22.1748463	22.0184662	25.5672179	60.1146205	14.53148
Smarcd3	1.05850858	1.78102997	1.08517939	2.1171347	1.27235501
Il2rg	2.4272341	2.60470108	2.35995898	3.75114707	1.03736849
Snord42b	3.55700125	2.79297881	10.9790876	5.44270844	3.80054092
Gpr176	0.06669567	0.62843809	0.18527737	2.00535486	0.25387147
Tnrc18	2.7575905	1.69597851	1.72058842	2.30352016	1.19499767
Dusp12	12.1026061	5.36642217	10.8552333	13.2027272	6.08529114
Tmie	5.83346259	3.42484681	3.89021017	8.91782305	1.39381758
Dynlt1b	19.3941259	16.0263784	12.861217	29.3906256	11.0622888
Aldh6a1	33.4153826	31.9665044	21.4047872	43.5546922	6.98745877
Haus1	15.0038847	7.80309319	6.7361733	12.1051473	4.73648091
Sema4b	2.79289801	3.17920168	2.87693275	5.58339679	2.44998124
Pomt2	2.23001034	2.00405477	1.86203938	3.23076763	1.28087019
Inpp5e	2.85448992	2.92243974	4.27879075	6.11004899	3.18473381
Lhfpl2	3.49455466	1.53095665	1.54156693	4.11708097	0.79323618
Rassf6	6.06155643	1.4121803	2.83729231	6.81867405	0.40033788
Ddx19b	2.56982362	3.16330711	4.19563801	6.99811356	3.79350289
Qtrt1	7.15798258	7.59735341	8.45664018	13.1885766	5.30090587
Plce1	2.00936219	0.68926178	2.08924799	2.88991849	0.34622133
Pamr1	2.46771635	1.47119066	1.35411312	2.11872778	0.03662049
Zfp275	3.92681336	2.16798575	1.89383666	4.62847857	2.02408454
Ccbl1	10.1342491	15.9947877	20.8397235	20.9108632	4.83462781
Wdr59	15.8845785	17.806889	17.2181613	19.6967262	6.90494677
Gzma	2.45100883	1.9245469	1.85694136	2.59117554	0.59518722
Gpr137c	0.53364721	0.73329026	0.29648927	1.56778508	0.2138193
Crtc1	2.43653123	2.51781576	1.42608171	2.46907499	1.52885701
Cuedc1	2.51522039	5.10601018	3.88175645	5.20035516	2.0155738
Cldn23	91.951653	138.288413	121.096193	243.845688	11.3210332
Klhl6	2.49458438	1.17099943	0.92899972	1.86704136	0.43457329
Slc2a4rg‐ps	2.88276725	1.76626758	1.4560344	3.24812674	1.64507674
Lrfn3	4.12559098	8.42970012	9.96215013	11.1327262	4.36544132
Cluap1	8.05435915	5.97119608	8.17752735	8.22036653	3.4401448
Tgoln1	29.8151375	36.2121785	44.7358486	57.8558607	32.556883
Ppp1r14c	5.21900209	11.441918	18.1824827	16.9125049	3.37478698
Clasp1	4.22691291	2.91708763	2.25516771	6.2454373	2.59997126
Ch25h	0.72350247	0.83321113	0.71461517	1.94842521	0
Batf3	0.1831789	0.43149954	0.50886221	2.27034327	0.29358111
Rhbdl1	0.80299553	1.12829345	1.21317851	2.01764694	0.27093941
Smpd2	3.58216522	3.48395921	4.63630012	4.37251296	2.28340864
Pcyox1	22.157689	18.2933278	15.8174598	40.9224195	16.6113132
Prdm16	1.84089691	1.38004994	1.78180593	3.92965067	1.14738101
Tubgcp4	1.57749698	1.51835758	2.21466575	5.53614549	2.16125181
Eif2c1	3.69262115	2.23983932	2.37641917	3.1952027	1.46474698
Mbd4	2.05606052	1.74301412	1.61745488	2.94003447	1.38517204
Jam3	2.38337139	4.88653146	6.59023916	7.17221883	4.77479126
Ubr1	2.62157546	1.95291393	1.75840819	3.54081109	1.4274677
Snord22	13.4563804	6.50217289	11.5019013	20.4317547	10.3685392
Sox18	1.08753281	1.96089309	1.71569436	2.77346168	1.5170473
Bcorl1	1.06468065	2.12342389	1.6168387	2.19929851	1.23711485
Poli	3.36283277	2.65835788	1.87256565	3.04564103	1.00144915
Smim5	0.76311552	0.38131094	0.25695737	3.12087218	0.61152321
Tmem100	23.5116976	13.2946214	14.2062174	25.8621214	5.24351841
Tmem201	1.24168285	0.98963816	1.33996739	3.93417822	1.39153664
Apoe	393.146733	382.008505	305.593382	446.463067	102.300931
Timm21	29.7543831	18.9632288	19.7456595	25.0405977	10.0143531
Fut9	7.01066454	2.84849674	2.41237751	5.20302953	0.87604212
Gsn	57.5096963	120.569606	61.2276588	100.299224	19.6365593
E130215H24Rik	0.55736487	0	0.90319418	2.04683052	0.33498358
Cbr1	60.7476146	53.0993243	56.3071803	73.0511163	26.7813326
Gm6484	0.37694618	2.13105898	2.09427683	3.25304076	1.63115702
Ephb4	6.05611884	3.18194215	3.30933518	5.65612351	1.97717891
Txndc11	7.66680932	12.0400497	6.00401568	11.9256927	8.51355636
Slfn2	22.455708	17.6323572	24.7128442	54.364938	15.4197127
Trim13	34.0652348	35.2521737	66.2172182	61.6327431	32.7147563
Heca	13.7643435	27.675904	22.8234484	26.4818118	20.6778135
H2‐Aa	27.7561448	17.1001369	21.6911442	41.2199883	4.35071277
Deaf1	7.13439267	5.86426762	4.35266793	7.57956396	3.44176319
Hck	0.84164227	0.51400423	0.11545886	1.80296221	0.19983724
Nthl1	3.20020328	5.26269619	3.41063325	5.04458439	2.17739324
Rnaseh2a	5.42367281	2.83078332	3.27922358	8.72857057	4.19286682
Cd34	6.96652921	9.20159177	11.7037661	10.3704258	4.66548662
Rogdi	19.9570257	27.2208423	23.7722487	36.3003629	10.9245075
Rpp38	58.6079741	26.0759609	48.8606817	64.5917531	22.5414264
Flt3l	1.03125463	0.80974735	1.29195776	3.17449134	1.31251239
Hgf	0.37872488	0.72485657	1.49044602	3.58566817	0.30349147
Il23a	2.35126769	3.84317173	3.64353828	8.01788248	0.96132152
Mpc2	97.2830123	71.650481	68.2336155	98.1472013	16.8888206
Nr1d2	5.10169037	6.63232145	11.5694502	17.1738565	9.20136349
Spsb2	14.2702425	18.5014118	21.4087553	43.8740466	16.5326754
A230050P20Rik	4.3784438	3.71180191	4.98723262	10.9565634	4.00547205
Cybb	1.82515882	2.78004835	2.71935736	3.5906757	0.75878855
Kifc2	1.24489385	4.67569949	2.05574072	2.76204898	1.3800087
Mir324	0	0	0	2.0180829	0
Dtd1	14.1300465	8.02192277	6.2126404	18.9922102	4.23598871
Uhrf1bp1	1.94105064	2.05607405	1.76918764	3.17973103	1.29316876
Tyw1	2.57854855	1.82376823	2.19631462	2.49382173	1.16723525
Stau1	16.3355936	19.0862881	25.2108308	33.1943407	19.9150756
Elmo2	5.10941529	6.61098544	6.31517271	9.32055708	3.45356597
Agpat4	29.7378961	44.9313764	54.4729445	74.1946594	38.8237194
Mpzl1	138.339569	213.533662	284.855132	221.487739	123.701074
Lipa	38.2797666	115.990251	128.696636	110.248279	17.0966777
Sugp2	1.81979115	2.65706587	1.54583326	2.36110115	0.98826335
Snord49b	3.10531855	0.81277161	4.79245889	10.4534559	1.65896628
Scarf2	1.79717255	1.50419362	1.68244044	3.84385102	0.50643186
Ercc8	1.91890116	1.40952419	1.57625724	2.75622682	1.51291111
Nkd1	0.32174415	0.57417147	0.74482485	3.62521733	0.76177023
Cx3cl1	7.51085957	3.06282754	2.5552665	7.08608429	2.37760534
Sardh	1.61670051	0.80884862	0.41069207	1.83891421	0.04585997
Lins	4.56469389	4.26786482	2.75262459	8.48431357	3.49844757
Cfp	1.02695574	2.18392372	2.69434303	2.94634807	1.37158629
Epb4.1l2	2.45475327	1.90454078	1.79950764	8.0756476	2.11933592
Metap1d	7.19970202	3.05031443	5.08404138	7.84631915	3.23755373
Asrgl1	4.53097931	1.93649806	1.75259842	6.31871164	0.85027493
Hoxd11	1.25436876	2.68729973	3.34117806	5.30311867	1.62567664
Nars2	3.16072636	1.45909279	1.55987836	2.90181153	1.54476048
Tnks	1.40664569	2.01036107	0.96518516	1.7095037	1.26507764
4930481A15Rik	1.17665917	1.77489869	2.12178949	3.09867399	0.94291673
Dnajb13	1.93980529	1.5231468	1.05660511	3.09039175	1.64590355
R3hcc1	10.547282	5.87739889	6.5810879	10.724502	4.05941835
Slc18b1	4.68917331	2.67123106	2.14976496	4.72711794	1.49201708
Tfap4	4.93794147	1.76901876	1.31458077	5.29846249	0.86559883
Tcea2	1.06536724	0.35222433	1.03843477	1.8532352	0.44933308
Gna14	1.38789289	1.10491772	2.85592584	1.92900012	0.86503592
Zfp354a	1.32721731	1.25819224	0.68942893	3.1205402	1.03762597
Sult1d1	754.728606	813.926603	402.137748	1663.12482	114.425016
Fcgrt	3.3018577	3.53541622	1.80668531	5.13268074	0.93209469
Fam35a	1.56795198	1.48333174	1.59184049	1.83841195	0.36331938
2010111I01Rik	2.45310431	2.04293121	1.58319132	2.90050293	1.07225293
Dusp23	4.00382286	2.34883539	1.91765998	5.99965461	2.13897769
Icosl	2.53917423	1.06210439	1.12068198	3.4640819	1.54033932
Klhdc5	4.84299262	5.30670225	4.19046596	6.45074011	2.36991576
Elp6	4.676032	6.3013427	7.60663533	6.52650357	3.49395659
Poglut1	12.8645552	12.0401605	9.07718208	23.5761395	13.3364305
Ifit2	4.012773	2.8007587	2.32426368	3.83566918	0.03969924
Cd72	0.5640399	1.7715501	0.88387865	4.06301987	0.45199381
Gne	2.26683893	1.72599814	2.25807605	3.68930781	0.33027875
Rab2b	3.40728712	3.06558814	4.66690899	9.14556704	4.47487493
Fam26e	3.90685934	2.46561959	5.88297137	4.96121463	0.43889225
Metrn	3.63616171	1.878379	1.86072579	4.48035133	1.11186038
Cbs	1.27661717	7.18051085	21.3024128	12.5575874	7.30727255
Acadsb	8.95979008	13.7340923	13.2204874	18.4366058	12.3307574
C1qa	25.9761906	26.1228702	23.8861112	32.2035299	4.87800481
Cd7	1.69299579	5.02199075	6.0384982	5.29617398	1.05519826
Tmem181b‐ps	1.77220727	1.94599267	2.32052691	6.88947509	1.63096462
Rps21	4.66989081	5.76215577	9.88398683	14.5463691	5.2123787
Notch2	2.49525026	1.74971022	1.25873892	2.96898554	1.66133487
Ano6	8.48402825	8.99286033	3.90160364	9.9276298	6.93593727
Wbp7	2.29786963	1.99805153	2.16349173	3.66708675	2.29248071
Txndc16	13.2430889	16.8436222	8.67996639	16.9294176	3.68448667
Maob	5.08283484	13.3318806	22.1061108	32.987958	13.1726875
Abhd6	9.11283273	6.34348508	8.10918242	17.6227239	8.23481922
Galnt6	0.27129148	0.22189553	2.03062689	0.77833844	0.27174955
Znrf2	34.9794503	31.8606472	66.1458481	59.9615473	27.0096872
C1qtnf7	1.81143582	3.08680282	3.03352451	7.99679265	2.3472608
Lime1	5.4259854	4.80941732	6.13405382	7.76259692	3.4144727
Erlec1	8.41921842	6.36218395	7.1751364	12.740073	5.10483967
Zfp651	1.49864736	1.81347987	2.22415958	2.16748817	0.1809639
Apmap	16.6155812	8.53410192	9.12667993	22.006933	8.08916501
Mansc4	1.04524525	0.27357762	0.75279586	3.41199175	0.09306757
Hpgd	1.85885561	3.84257992	1.05383913	3.41317027	0.97279698
Gli1	0.62326847	0.75506527	1.20374213	2.05996556	0.48518648
Smo	10.1006791	3.68230297	5.42052768	12.9012855	5.43992437
Ttll2	0.52233639	0.75718464	3.16249136	1.29109227	0.19318831
Braf	13.400305	19.7017744	16.1949443	36.9139615	14.2783994
Zfp61	4.22333908	1.94171727	2.70150111	4.59163302	1.13554722
Bgn	8.32547732	8.35906174	4.52445031	15.3120316	4.44046038
Kcnj13	0.19336305	0.6452768	3.53625914	1.46456869	0.38737908
Atp13a1	4.48184581	5.03673363	5.32264149	8.22941965	5.36726877
Trim68	2.42940158	4.92970308	3.51339996	5.28778831	2.88739296
Ccl3	0.66037154	0.38889578	1.37586035	2.12196734	0.06614866
Ftsj1	5.15981544	3.18440358	4.03741924	7.62138092	3.92121503
4933412E12Rik	1.36172902	2.85130087	2.45182642	4.41036177	0.21218217
1190007I07Rik	15.8670154	6.2978934	7.91140139	16.1680456	5.30957923
Rgs2	18.399709	22.4966755	34.4770353	65.4608236	17.9575243
Eif3b	42.4117833	47.0958126	71.4806716	91.6617428	44.4522978
Wbscr27	5.79297563	8.91468085	7.56158712	13.0077431	5.76080922
Tmem176b	379.042945	358.932326	420.424541	949.877528	388.307464
Gm10336	7.00178533	6.6924373	13.4633536	20.5861933	9.79480606
Avpi1	73.8308575	44.4367889	91.4436511	122.806686	74.10276
Fas	0.36208601	0.39803696	2.2799438	0.96403331	0.55130001
Daglb	3.31891696	2.32173902	1.71357753	3.28711385	1.24115444
4933401H06Rik	0	0.0546474	0.77334022	1.78906531	0.11154202
Cpped1	19.5659557	36.610432	30.9521219	53.8221119	28.0223887
Cgn	9.59147716	15.1133897	9.62468054	17.6234424	7.42682124
Napepld	18.9438804	12.8534309	11.0971584	21.0432623	3.9694596
Nup85	6.19193903	2.1582305	4.05549903	4.90826926	2.46885532
Lrrc17	6.58673829	8.65891511	11.6464636	53.5489874	1.33748469
Telo2	2.49322517	1.40664042	1.29970508	3.17067438	1.27276682
Dctd	4.47393416	3.20028822	3.92589143	4.60206022	1.44533861
Hdac5	2.8673913	3.28960643	2.87774664	5.54875383	3.46731814
Rab3d	18.7355022	22.6560875	20.4406365	53.9091333	17.9732072
Mgp	98.8517765	79.0722298	143.149707	192.802722	90.9925
Habp4	10.1869449	7.02143485	10.7973147	15.8828706	5.92400248
Cst7	1.6534404	1.40215468	0.489939	3.3395929	0.42399544
4933431E20Rik	1.76084828	0.52085327	1.17263235	2.65743486	0.21262505
Prodh	10.6258206	8.43317299	6.55956003	24.939191	5.76823228
Rnasek	119.939185	193.293292	227.463043	214.068198	140.47337
Cables1	5.87158796	3.50758482	4.01001077	8.3097768	1.43813469
Slc39a4	153.742564	200.447882	166.831824	415.887963	66.6247117
Fam102b	3.80839927	3.91532982	4.60671119	4.61990684	2.39200127
Lilrb4	2.57535586	1.37219692	1.44787686	3.50436728	1.17929338
Sox12	1.11749457	0.9467369	0.80331769	1.59292797	0.23565925
2900056M20Rik	3.29584233	3.94807536	2.59766143	4.23620302	1.26528352
Zfp2	4.35026167	5.30617736	4.59535971	7.07657849	1.25228316
Syde1	8.27046063	4.7498195	1.96508305	9.46845871	1.71648518
Anxa11	1.96043493	4.29733901	2.370016	3.24971735	2.66196551
Ccl5	5.03517754	4.62866545	4.88993263	5.75020609	1.86985182
Rhebl1	2.20418736	1.3508224	1.64279011	3.94854363	1.03394765
Mzb1	1.58350681	1.8085514	0.59985082	5.55084172	1.32662369
Snord15b	0.44973579	7.06270504	0.41644815	3.30316098	0.72079224
Cxcl12	0.32783422	0.77940269	1.03719492	4.47288635	0.04378503
Rbm47	17.4448558	19.5069729	23.6117879	44.6012111	16.5202816
Prr24	4.30862949	4.57184032	3.77406138	8.58846432	3.88820221
Ikbke	4.58090232	3.13334495	3.63855808	4.48602881	1.09311531
Tfdp2	1.90218532	1.53379412	2.67763361	2.73310107	0.60835997
Auh	22.3513672	24.0604569	33.9861944	47.0946028	23.5449868
Clec4a3	0.44128211	1.9634851	2.13390538	1.84561015	0.90370005
Kctd18	2.86330141	6.83852295	9.27979966	8.23675159	4.9331939
Trnp1	1.8924795	0.68107706	0.69366062	3.69209121	1.16899951
Mthfd1	11.4179312	13.1554568	8.99234841	13.54455	2.56256388
Ank2	1.07145217	1.42480037	1.57623446	1.60245106	0.51931997
Slc25a27	1.06785052	0.85753908	0.85397444	1.87162731	0.27227545
Fmo1	6.86881511	5.63558923	11.0593303	15.7782386	0.7863329
D2hgdh	2.08984486	2.62888293	2.98173064	3.77857096	2.03356281
Thy1	7.59236962	3.60055482	5.35981973	15.2866387	5.21068399
I830012O16Rik	5.76635904	6.30836744	4.67961113	22.0979911	0.31151975
D330050I16Rik	0.86497627	1.56507752	1.84567708	1.62276832	0.99451871
Gpam	2.50421065	2.71539607	1.73520063	8.50814192	0.90099031
Zfp276	2.48705644	1.33339874	1.31244784	2.5165691	1.2965245
Cldn8	29.8956049	15.005253	6.73505101	32.1438328	1.13024993
Pigw	3.38576231	0.93609893	1.87668134	2.23280397	0.72606312
Dixdc1	0.61595789	1.71070156	1.18298373	2.49239298	0.17367349
B3gnt1	11.5041752	7.19632378	6.88418468	13.2096005	2.59361239
Zfp874a	7.16523502	5.07304948	9.15093401	8.22285734	2.51609885
Dnajc28	1.93698088	0.78219209	2.01567974	3.74292057	1.2861095
Zhx3	2.88212378	2.77739299	1.81963674	3.04694481	0.58323033
Klhl13	1.03221483	2.30436644	2.82996036	3.21461023	0.90826087
Hsd3b7	29.8335431	26.9084026	28.5859789	66.0768092	28.8376206
Ptpn14	3.48394544	2.07792355	1.48377097	6.73702341	0.97277434
Pnpla7	0.68210092	0.55790599	0.63161454	1.95695553	0.11387544
Otub2	4.51127973	8.18722456	5.41270499	11.5227192	3.33378539
Rgs5	3.96533214	4.07598898	4.70662381	5.26218743	1.79102329
Ahcyl1	56.4230978	36.8976362	33.2579971	84.697645	48.7564201
Nhlrc1	1.5066345	0.35713766	1.36879645	1.69639779	0.54672123
Zfp763	6.3574173	2.0421337	3.56779805	4.36863776	1.34310106
Ociad2	7.82184736	16.0523317	27.2157666	14.9878491	11.0165612
Dbp	3.73082372	9.00200262	10.0401728	8.91981418	1.12113692
Spry2	12.56114	18.5484841	21.9029379	47.9933701	11.6433678
Snord88c	0	0.60957871	1.43773767	2.13820689	0
Prkaa2	0.94624937	0.21228592	0.27979872	1.70827113	0.4396736
Selplg	1.62778795	1.98604676	1.66963084	3.86256728	1.00340702
Ly6g6e	9.75020864	22.1121058	26.6076306	23.431889	8.27294528
1700019G17Rik	4.01853609	2.63989608	1.34475402	3.12316491	1.13187251
Flcn	6.14214927	10.3897266	7.99074749	10.1750686	4.31184878
Dhx33	4.0041161	2.35309858	2.40187417	5.6644523	2.56292512
Med23	4.74650389	4.85543122	5.01783818	8.23118866	3.37042512
Abcc3	7.14434344	1.06463007	5.93950643	4.90736006	1.31636089
Tmed1	27.6722099	29.820574	35.6543472	37.2022115	15.3925741
Rftn1	11.8926386	12.4630782	12.4347376	19.8497636	2.87210757
Gbp3	18.2612499	7.66419915	15.2163748	30.5587764	7.34653633
Zeb2	1.53851786	1.34290598	2.41195363	3.4359598	1.35331453
Hoxa11	10.2874056	14.1246232	19.5363177	17.1818835	10.5958708
Fance	7.50451983	8.50870281	7.18868834	8.93889267	3.83635951
Spata7	3.31857906	2.07449513	1.64089625	2.72135422	1.05857458
Txnrd3	1.79355141	1.06525814	1.36270763	3.27215017	0.82919066
Snora19	0.5874927	0	1.08801769	2.15747001	0
Pan2	4.32302212	5.94894026	3.09388223	5.62372436	2.91232462
Igflr1	0.76978386	2.24505885	2.59665606	3.33170854	1.27779569
Slc25a42	0.63731475	1.48512745	0.62821703	2.45368003	0.39539045
Fam161b	1.45691889	3.98105559	1.92469261	3.03183342	1.58780418
Mthfs	24.5392946	18.668348	27.2046924	24.7898361	11.0230533
Ddhd2	6.58688498	5.18406191	6.86353792	12.2209022	3.33115141
Msl3l2	2.65112943	0.52640255	0.28217281	4.22445734	0.80583899
Noa1	6.11625052	5.75857333	5.87331132	9.87864579	3.08597982
Pdgfrl	3.27547299	2.30470854	8.42947564	6.36580315	3.78380664
Fbxo32	2.88638822	2.34761627	1.94144334	3.28006362	0.99451871
Afap1	11.974037	18.3321331	15.6688853	22.5925454	3.78287935
Dst	1.90792311	1.16984979	1.96156361	3.51099422	1.25236179
Timm10	32.0183521	17.2219714	36.629929	48.2733915	25.1086788
Ddx19a	8.35722623	6.16443867	8.09041507	14.8570088	7.79295382
4933439C10Rik	3.50899342	7.33832739	7.2384768	10.6534421	4.78864107
Lrig3	1.61016517	0.5436539	1.46116747	3.42957825	0.55483206
Ncf4	1.07639653	0.95084217	1.03825623	1.72938879	0.2156428
Wdr85	7.00274579	3.78137036	4.3979577	6.61163701	2.42522661
S100g	1029.39369	4023.50903	6447.35485	10720.5595	2118.49475
Tgif2	2.21967413	1.22920422	1.86066229	5.23404853	0.69277148
Ermp1	17.4695757	7.21459117	7.17589423	15.8942424	5.22186854
Pth1r	2.25281103	3.12383885	3.52856014	3.16841241	1.45959767
Mettl4	6.00554164	2.16726495	3.56692685	5.90344003	3.4028099
Ctc1	2.59782589	1.9169473	1.4346324	2.57180055	1.73062789
Slc6a6	27.1354202	38.2810291	50.1462576	78.9788254	27.1664132
Zfp608	7.5966497	8.96415811	9.34623577	17.9354697	7.64367626
Rhod	6.28722567	5.93177488	7.62710162	17.2271078	7.49882514
2610015P09Rik	2.39867318	1.84128578	2.58579665	2.8266825	1.49184011
Atp8b2	2.46172037	1.42626002	0.97377285	2.67697076	1.33109471
Sepp1	37.1082764	32.7885125	30.9282286	45.604788	5.89546905
Tnfaip8	9.86948216	7.87873647	5.53416175	11.3933827	3.04639771
Snora75	1.68650921	1.3242572	1.56168057	2.58059452	1.35148546
Sbk1	6.66140915	2.22737558	2.93661081	6.48150494	3.34590632
Zbtb16	0.30603843	3.04384081	2.04274578	2.34140762	0.23502563
E230016K23Rik	1.99657582	5.57925078	2.01192646	3.90865374	0.25881667
Mir1249	0	2.08998414	0	1.22183251	0
Qsox2	2.50302116	1.88175379	1.0849078	3.39808134	1.92044483
Pus7l	1.90048112	1.99568535	4.00728989	3.4265366	0.84404569
Vstm4	0.76076342	1.04537304	0.30819896	2.24811836	0.09525599
Lrrc56	1.6377365	0.85730704	0.52848348	1.16185671	0.17896383
Cry2	2.71777593	3.34172528	4.12061476	4.04432995	1.24450962
E130308A19Rik	4.33536733	6.52378659	7.32193372	8.85155698	6.01494619
Hsd17b4	22.6115765	32.7633231	29.5740229	31.3508114	11.7165652
4732471J01Rik	0.43695448	0.50873343	1.61845146	1.21731557	0.61578774
Siae	4.82486721	6.23464248	3.83452551	8.59767196	2.69431007
Ankrd13d	1.21834077	1.74588353	1.07175587	1.95744302	1.02513423
Mir17	0	0	0	2.13820689	0
Mir3098	0	0	1.11824041	2.21739973	0
Rbbp9	83.3260477	71.2834569	45.4564726	92.6873088	13.95951
Cbx7	6.1762886	13.9117956	10.6242502	20.7360983	3.99201304
H2‐Eb1	6.21249769	5.09284932	6.54864095	11.2995714	1.75339515
Fam20a	0.56460337	0.68514632	1.71102665	4.64161715	0.45244535
Gnb4	6.51879848	8.37589065	10.2946407	13.7759283	5.31882318
Ptpdc1	1.05887689	1.35405375	1.21862525	1.6804094	0.44850967
Ankrd23	1.10132331	1.51989123	1.01980778	2.9107627	0.45464506
Gm16973	1.02127934	2.71235851	2.41984958	2.75770579	1.58866462
Zfp580	0.88763643	1.48688527	1.09591619	1.68417747	0.47420542
Mfsd3	2.73594456	2.36074742	2.33856085	3.14668344	1.06008633
Hpn	3.9759251	4.60611198	2.5952566	6.13358697	2.08925288
Hoxb4	5.23523307	7.50309195	9.15423149	9.2394536	5.9670418
Sdr16c6	7.68171597	16.7499831	19.2413163	15.9821905	5.97860516
Fancm	1.23294127	1.26447401	1.64651012	1.38605308	0.99473965
Mgst1	1586.10319	935.855228	719.304882	3379.05618	702.122731
Atp6v1g2	4.18750649	8.01463485	6.05869385	7.64891667	3.42557117
Itpr2	17.1095882	26.38837	30.0162852	44.6326951	16.6279693
Cd93	2.25951922	3.02383364	2.83822151	3.22889211	1.18874256
Adk	13.5634969	10.7812988	12.2811669	34.9649405	13.1714443
Parp14	5.16225483	3.95456936	5.62960252	5.7136804	1.41255796
Sparcl1	18.793356	22.2007157	31.8590855	62.0601511	12.1587427
Slc52a2	20.3605386	28.2763244	32.0711349	55.4793413	33.7350792
Gab1	4.26781586	3.47757189	3.7158077	9.17945989	4.89342131
3110040N11Rik	14.8381324	12.188729	15.0786303	16.4868974	5.91478583
Gfra4	0.65255193	1.33220804	1.24878882	1.51771323	0.41833839
Elp4	3.02493998	2.16523857	1.91894869	2.63905801	1.43299483
Rbak	2.80154719	6.98582509	4.36454587	5.17886742	1.36521608
Mcc	1.02921024	1.68919924	0.80255346	1.35696338	0.30385837
Prrg1	3.07555762	3.40933605	5.60445735	4.9375592	2.45142079
Hoxb5	14.3768387	16.6090908	16.1472499	29.3349354	18.6869951
Adat1	1.21794545	1.16705667	1.96886773	1.97102199	0.82711994
Parp8	1.16147763	0.41454511	0.33243028	2.03572806	0.71075531
Dpy19l3	1.69171245	2.48313329	4.0776197	2.98919706	1.67583492
Dkk2	2.16492249	0.95360815	1.20606982	5.21941783	0.23977772
Ptprcap	3.16037739	2.84470064	4.78226217	4.59992498	0.98832034
Twist2	2.33608949	3.59056727	3.82008652	5.65842554	2.50931294
Snord99	12.809439	10.0580487	20.4877618	27.7966895	13.0643594
6230400D17Rik	0.47949772	1.55308105	1.99803249	1.43071196	0.5763688
Gpx7	13.4828123	7.80300917	4.32742458	11.7865572	3.65888155
Rsad2	2.86001069	3.70675391	3.01526066	6.4694175	2.15380721
Ntpcr	10.4451624	8.06860545	6.58745259	9.53769859	2.75991662
Eppk1	1.10582094	0.32820806	0.95538106	3.21086678	0.74623197
Ccdc24	0.15272058	0.39972374	0.37711152	2.6172587	0.20397126
Ift122	1.50722863	2.54323372	1.46990736	2.13452667	0.60390941
Top3a	2.61544209	2.02627875	1.38853167	4.05001539	2.23561231
Six4	3.39400394	1.8838739	1.33297789	3.49204642	0.92847924
Ttc21b	2.02874325	1.31793427	1.37852913	2.65313764	1.04093822
Usp40	5.01722743	7.1952304	8.417094	10.7216032	4.93476932
Cped1	2.31662597	1.13950646	2.1821441	3.17806168	0.0853531
Tox3	13.5202438	5.85333016	6.69473337	23.025401	1.60389254
Zfp12	4.61108177	6.12633025	4.59497054	6.27995017	1.60283876
Fahd1	20.1150485	12.519109	15.5790679	18.552402	7.24250203
5730408K05Rik	15.623634	8.13406589	26.8901873	32.1176493	13.3365336
Uap1l1	8.56626928	2.39358835	5.6097291	19.0945671	4.23625383
Bace1	5.48872828	6.38531517	3.42671095	7.28030384	4.49807702
Pld6	0.77413538	1.01309294	1.46780906	2.84288445	0.23632533
Ypel3	50.286697	77.9010329	105.688055	170.626067	56.4261222
Bbs4	4.48939641	3.8071092	6.74639453	8.14907486	2.79583459
Sp110	3.50707209	1.19503406	2.11393392	2.61227914	1.14006587
Ppargc1a	2.52011302	1.16678715	1.88732123	4.52595354	0.32987759
Magi1	2.9340916	2.40357816	1.49152205	4.26457989	1.40324369
Per1	2.10987161	3.62944565	3.99738813	5.30136948	2.5472442
Elac1	0.73463179	0.97856329	3.04901036	2.43285016	0.98817122
Rangrf	4.18866016	1.87019362	1.52103229	5.05198503	1.71120073
2410004N09Rik	10.0904924	4.96672906	10.3198353	12.85887	6.99984154
Gimap4	2.12568491	4.97901195	6.40547876	7.83930436	1.32808958
Hdac8	4.69039994	4.90067338	2.55690469	8.05672385	3.81927909
Sestd1	16.5820883	19.7941498	19.6259639	33.911698	20.5228147
Etv5	3.39537578	8.46711525	20.7445006	20.3325461	3.37718135
Nadkd1	1.36331058	1.89392559	2.92942422	4.49304261	1.59671613
Sertad2	5.71292054	7.63778758	5.19990139	12.8242745	7.60407713
Plcl1	1.07442601	3.06993323	2.76361474	2.94097228	0.82113136
Uaca	2.28270368	1.1679462	2.08647296	2.50133506	1.67582569
Zfp39	1.84403235	0.65815696	1.31946619	1.5852413	0.2418082
Spon2	2.94429175	1.03004359	1.26870548	2.1410744	0.51393099
Aplnr	1.07954256	1.5516549	3.87995536	3.3260996	0.49852775
Spry1	5.26407614	3.41364077	3.17688058	12.1903554	3.81961994
Zfp13	1.46337592	0.6702801	0.64930088	1.62339784	0.43975403
Cd302	10.3238872	4.8882684	12.1058198	15.1936865	4.78091117
Lipt1	9.01640724	5.66378983	7.72772439	8.97084355	3.29339479
Adck5	4.29614867	6.04184207	4.30473078	9.09526351	4.5731681
Fah	1.60457785	2.3906254	2.09537793	5.32595633	2.86838933
Svil	2.4331409	1.01388442	0.9516491	3.58863925	1.7527077
Zfp661	1.67992643	0.51393053	1.77781145	1.24186255	0.6818468
Trub1	2.18222073	1.66545101	1.21808642	2.50900774	0.59652744
Rxra	6.7477446	7.67048386	3.47382752	9.78695492	6.49869408
Uhrf1bp1l	7.18279547	8.69032656	7.19448747	14.0990297	5.14791699

**TABLE 5:** Gene list of cluster 5

	D3AM	D4AM	D4AM	D5AM_mesometrial	D5AM_antimesometrial
Fam110b	3.20338124	0.97532593	1.42260359	1.53050598	0.94299136
Birc5	26.8597328	2.68314563	1.76771025	5.95893722	5.90496808
Hn1	212.106062	69.9067377	82.1733776	79.5324788	95.5487613
Cdt1	12.8280412	2.99141501	2.64580478	5.92959609	5.31876249
Fbln1	8.32861411	2.17989266	5.42907549	5.81052469	3.23594732
Ybx2	1.58442724	0.09821856	0.61774918	0.42107911	0.36753952
Rhbdl2	9.85945018	3.0714352	1.09159376	0.59033485	3.73574123
Hist1h4k	2.50814191	0.16411735	0	0	0
Sapcd2	2.59091621	0.37734895	0.13543572	0.70976685	0.4185953
Mcm2	27.0441833	7.29107491	10.4433238	12.4321729	7.40998375
Hist1h2ak	7.6329204	0.65145816	2.61207301	1.37106396	1.32970579
Rundc3b	5.19919049	0.30653101	0.27933189	0.5050241	0.7820841
3110021N24Rik	3.41893516	1.66725832	0.96642631	1.22250681	0.60563256
Pif1	2.46315893	0.18088586	0.16408963	0.40672414	0.11360313
Zfp933	5.22062031	2.49572748	1.91946611	2.90397394	1.61189648
Krt25	3.35735531	0.0620286	1.4264169	0.14505098	0.15825996
Tspan3	63.1372865	15.547959	15.85586	36.2889201	13.3288201
Acp5	18.4416721	9.37906251	4.78296887	10.7069122	4.3008906
Slc35g1	17.0779101	1.91777571	4.8885566	9.4867145	5.31457088
Ly6e	511.972695	154.392697	120.927422	214.24071	65.0208797
Hist2h2ac	1.6720946	0.13129388	0.30966658	0.61404916	0.53597372
2610204G22Rik	2.63765009	0.88131862	1.40309339	1.23655338	1.12429943
Pstk	11.6676362	5.71459792	5.2415662	8.64373448	4.58236168
Kif15	7.58527013	1.44387874	1.28958183	1.27857944	0.66093794
Hist1h1a	3.31732825	0.068547	0	0.48088187	0.27982564
Fzd2	12.5821893	8.24241817	3.84479902	7.34345584	6.16487964
S100a10	542.661583	201.352288	214.320236	197.846638	253.971147
Snord37	3.69122771	1.93224949	0	1.12961873	3.94395756
Pola2	5.33591471	1.60460134	2.39689692	3.02267408	2.80210495
Tubg1	14.3473823	4.70993319	3.97812809	4.24186226	4.44951458
Cdkn2c	24.5870475	8.00940126	9.17240234	13.532955	3.26018373
Gjb1	12.2053964	6.77708094	1.81639318	10.5652576	3.03903548
Nudt11	4.47506829	0.60516316	0.6445976	0.68474792	0.83675653
Cdca5	16.4910988	3.86855407	4.65718382	6.28224478	3.78359203
Hmgb2	125.045595	50.4057283	60.5420009	79.618818	51.7915467
Mxd3	6.82739783	1.22197302	1.53402957	2.94970496	0.20114487
Ipo13	4.94358405	2.62587751	2.01956462	3.05355967	1.82056291
Hells	18.4467648	2.44235312	3.44028384	2.30039615	1.95597564
Nuf2	7.07702743	1.29874483	0.95410782	2.58896401	0.71704592
Dlgap1	7.03247303	1.66107144	0.95296941	0.62989261	1.12578648
Serpinb9	14.2824542	3.87388436	5.99050308	5.95701795	1.98445966
Laptm5	16.4524905	8.26362997	6.21980424	9.48728993	8.21561885
Mir676	2.19814684	0	0	0	0
Tuba1b	309.358911	100.639025	104.99719	112.380431	88.8242619
Tsc22d1	69.7739802	35.3966762	32.2755389	28.4149462	60.5922965
Rfc3	16.2089961	5.47868272	5.36755396	6.7507503	4.47306463
Bdh1	3.09134322	0.50697635	0.2898769	0.08981367	1.11319474
Iqgap3	3.76839925	0.26161623	0.08510921	0.63287307	0.31302905
Clca2	2.83761867	0.15414634	0.46272017	0	0
Ifi203	2.32284382	0.8106271	0.52577953	0.66346472	0.0965177
Ccna2	26.7249614	4.13498642	2.19113062	7.75261635	3.92256114
Cbx5	16.4444345	6.77523738	6.53845844	7.24886875	5.32693222
Scarna2	8.55235273	4.19709931	0.98991774	5.88883208	5.99675515
Ift27	10.8043036	4.44379272	4.10903724	4.9596278	1.85529365
Cftr	10.7479292	1.5432862	2.16481693	1.73797146	5.5208524
Htra4	2.30229257	0.46955169	0.11074733	0.54901231	0.69484901
Mcm10	3.77010913	0.3663753	0.25923719	0.78821135	1.3311139
Mal	58.5215054	12.8656655	9.89026884	19.8919434	55.7186935
Ifitm3	3971.06986	1425.5111	1325.0217	2595.00443	1178.13103
Pcna	89.7954614	28.1625363	37.3811794	45.9476902	48.110022
Csgalnact1	3.68514375	0.04822662	0.22749228	0.09867873	0.63983677
Mir1191	5.43430746	2.13352548	0	1.24728735	1.08869662
Gins3	11.787851	1.67709963	2.02785387	2.13466094	2.16656044
Dck	13.4817755	1.84249502	2.31110334	2.15429415	1.57268049
Rnf187	93.8658203	59.0477109	45.4989473	56.3807332	36.1409097
Aldh3b1	4.27150805	0.50310208	0.29665111	0.45752025	0.31377283
Parm1	9.1014876	3.06982084	1.41911948	1.03372304	5.76460943
Cep128	1.9806338	0.50064838	0.15074262	0.58537139	0.51094229
Rasl10a	5.12041245	0	0.76298714	1.1887504	2.12236886
Nup107	4.09154844	1.34138859	1.6404717	3.09804879	1.67318445
2810408A11Rik	2.72689263	0.5811754	0.54108407	0.85834828	0.4370395
Aqp5	12.2350752	0.13205574	0	0	0.26954191
Rps15a‐ps4	4.0231028	1.32235726	2.5124311	2.23330647	1.19959685
Kif11	13.4916071	1.65758859	1.84199883	2.49716297	1.34466119
Ptn	45.7546639	4.48452065	4.27266325	4.88793459	0.82743098
Prr16	1.72998637	0.04604731	0.27151521	0.32303845	0.1409823
Rnf141	15.8919243	6.34679008	4.75722022	4.40219065	2.68972106
Zfpm1	7.3185003	3.100102	2.28271639	1.67974906	2.62367525
Kif23	13.023298	1.62957742	1.44571344	2.08014755	2.19710103
Ccl28	9.7146058	0.62995778	0.46835102	0	0
Chaf1b	6.37458753	1.20449949	0.78913986	2.40981393	1.66633753
Sdf2l1	76.3353442	17.7460026	21.3091408	40.7957523	68.8119148
Gtse1	4.19888217	0.2947028	0.32581825	0.68914869	0.56392918
Rai2	1.74209856	0.30624768	0.3611542	0.09548611	0.37505338
Slc36a1	3.77124864	0.97088759	0.69842319	1.77089262	0.96112466
Pggt1b	12.1795224	7.95393271	5.0100113	6.25831898	5.88278173
Eri2	6.57781122	1.97989252	2.48713711	4.7305356	2.53307277
Dio2	14.5226377	4.68458355	3.58259997	3.90208968	2.89370506
Kif18b	4.61329698	0.44512402	0.09050507	0.84348929	0.28196459
Spag5	2.09864935	0.40192003	0.36338425	1.00252924	0.27345598
Gramd2	2.24046009	0.7860357	0	0	0
Hist1h4j	2.29913008	0	0.19354161	0	0
Kif4	4.27640324	1.26055495	0.21785753	1.19434646	0.68759786
Cenpw	26.0501723	4.19931999	5.67107636	4.51399231	2.76494379
Trp53i11	8.62303333	1.23106504	0.93004668	2.13660042	5.7714826
Zfp454	1.93478262	0.39847947	0.46992204	0.69886918	0.05083408
Rnf183	9.5680969	1.42235032	1.11824041	0.25585382	1.89824026
Ttc7b	3.29658163	1.04134887	1.15787589	1.32193616	1.59414031
Zfp808	7.18291892	0.9043846	0.75626663	2.03795698	1.07401285
Htra2	6.58169024	2.46518721	2.34674814	6.2856337	2.87961519
Topbp1	10.5390052	3.81705283	4.71406923	5.78667142	4.3147405
Tpgs2	3.86191196	1.053438	0.90510921	1.81236992	1.22868333
Casc5	5.56672967	0.5964062	0.35166733	0.86249203	0.35238732
Fbxo5	33.7787169	5.1289622	6.31586143	7.42160132	5.61037239
Prim2	6.02627384	0.81948527	1.62107334	2.68904077	3.93886727
Agrn	17.3214408	8.09808669	6.75945185	9.38736282	3.59761192
C330027C09Rik	11.1256984	1.66215271	1.70184147	2.96787851	1.88042617
Kcnn4	5.53835164	0.1508236	0.20750853	0.26452044	0.38481177
Setd6	10.2369459	3.58953838	2.53262357	7.64066259	5.79246613
Arhgef2	8.45735108	4.44477143	3.44879176	5.46851922	3.82502895
Rab42	1.86319113	0.13933228	0	0.0814555	0.35549277
Slco2a1	10.7042248	0.05078563	0.29945441	0.3265895	8.22798734
Bard1	2.40593789	0.15038065	0.2327615	0.29671153	0.18224877
1700024P16Rik	3.53507246	0.016328	0.69319495	0.07636453	1.16646066
Krt15	5.38375576	0.02977012	0.14043019	0.10442406	2.09637395
Tbc1d4	3.5448607	1.10258387	0.96383197	2.20871845	0.11016475
Gpr126	1.84457354	0.25976206	0.22278856	0.20248047	0.26510307
Esco2	17.1633388	1.6603082	0.72903566	2.87061097	1.92878435
Bcl2l12	15.5901435	8.14510565	6.79682123	10.1360952	5.29866113
Slc14a1	5.97433751	0.26916066	0.13603626	0.53950251	0.19621066
Sectm1b	2.6118377	0.11025972	0	0.23205346	0
Sgce	7.75584361	2.78553087	2.67304187	3.51236118	2.53622764
Smc4	27.2700524	8.49228859	8.92017251	8.06672857	7.10506564
Pla2g4c	2.97522486	0.58771434	1.10893511	0.27486849	0.28490425
Rnf26	16.1665263	8.20716803	4.60000083	12.3904899	9.87161637
Gemin6	30.2039409	7.77970617	16.6809343	16.4834881	12.7515847
Gm10941	1.65092885	0.28807095	0.33971861	0.92625558	0.66148655
Rad51b	2.02278064	0.7400032	0.61725925	0.52758013	0.2026196
H2‐D1	807.821073	380.465601	281.019741	254.274183	263.424445
4930426D05Rik	2.01146482	0.41064772	0.07450337	0	0
C77370	3.61655133	1.90412888	1.26968698	1.98448216	2.03924442
Leprel4	13.7269384	4.44303539	4.46337843	8.14511375	2.63106542
Lrr1	6.34304242	0.17056833	0.68390719	0.75784548	1.46223343
Rab4a	13.6242553	6.49123616	5.43122714	4.19766961	2.84973279
Tmem107	10.9803082	1.86198587	2.1003472	4.87477364	1.81765001
Dna2	2.26231723	0.43477957	0.7764202	0.49383138	0.36765301
Xlr3b	7.13819978	0.09499928	0.26140685	2.73986683	2.13295664
Sema3a	6.41558471	0.69556209	0.3977057	0.50932041	1.16876542
Rassf10	7.3845665	0.76140686	0.77704438	1.18130275	0.08966103
Flrt2	4.62187964	0.76478636	0.68068178	0.72548995	0.34607575
Ccdc92	4.59603663	1.1121569	0.50856146	1.67189581	2.50168585
Myo5c	2.66387621	0.76061515	0.17939686	0.51581224	0.20958865
Muc20	5.22241642	0.03586594	0.18328386	1.02042841	0.29282711
Tmem221	42.6693771	15.8704352	3.45281249	4.66820996	3.08994596
Tcof1	4.78979789	1.28315726	1.5523467	3.45328034	2.69810491
Mphosph9	1.64892189	0.34236003	0.37437808	0.53130256	0.2032869
Fam72a	1.57995067	0.41352893	0.02868645	0.34130048	0.29790463
Tmem186	6.59367674	3.11355352	3.23116304	4.86780108	2.28785169
Fam171a1	26.80191	13.8941602	10.4428442	11.4223363	5.60891456
Lor	2.45597707	0.4893077	0.16971608	0.06730724	0.35249536
Mir5106	2.67993245	0	0	0	0
Cdc25c	4.53558892	0.68589382	0.2177717	1.6656202	0.80768837
Psrc1	8.12847343	1.55953132	0.37463892	1.01302526	0.56000638
Homer2	1.90680875	0.18371015	0.17873391	0.2309087	0.76869852
Clec14a	5.5456515	0.71070512	0.90464393	0.34294219	0.05756492
2210404O09Rik	13.2126341	3.57348707	2.28380842	3.18083546	2.09406211
Hmgcll1	14.6658655	3.31448938	2.37196887	12.7689102	1.85033251
Cks2	163.311119	32.4640188	27.1471389	30.6250381	29.6677672
Mettl21d	3.3018577	1.19660241	1.52873372	2.50671966	1.42474027
Dbf4	17.7704522	4.52544848	3.90377544	8.86778436	9.36528548
AI450353	5.30388408	3.03814028	1.48949622	2.87375005	1.95094434
Styk1	21.2159996	15.4917445	7.57940568	4.11183894	9.77699841
Fam64a	12.4106831	4.01673283	2.30665689	6.82009236	0.89115685
Serpinb7	4.83817516	0.05427092	0	0	0
Rcc2	38.3964766	22.7817704	17.376512	21.8055099	13.9972802
Usp1	49.8914216	22.4800734	27.0014147	23.430959	14.5476971
Hist1h2ah	7.91976591	0.26462332	0.62413418	0.46410692	0.67516069
Ezh2	9.73162821	1.84313562	3.28302302	4.73660528	4.36948204
Mex3c	38.2639178	17.3568573	21.51955	18.0343077	21.2359882
Brca1	3.84521394	0.34660161	0.56315717	0.72045479	0.76248066
Pvrl3	32.6451573	10.8903948	15.9153088	11.3618343	15.7389775
Gen1	1.5493487	0.21835655	0.64376314	0.56532549	0.14325828
1700030J22Rik	1.51450911	0.56874863	0.28454721	0.74560159	0.77392368
Pomt1	2.60846758	1.05128052	0.81225817	1.67423491	0.35146595
Tspan17	4.44244158	2.59971999	1.12542704	1.76992961	1.94789935
Scd1	15.1805587	4.42495919	8.21445487	3.88652366	6.80632329
Atrn	6.62790206	3.21750392	2.14253685	3.24805789	3.09824342
Cklf	10.1019062	4.22230591	6.87893742	4.19488062	6.79994304
Sgol1	7.64326536	1.84444431	1.08756548	1.29394398	1.33213751
Cyp4f41‐ps	1.69761131	0.45964642	0	0.16122924	0.28145837
Mthfd2	71.1658003	55.7808691	28.5297162	17.2089501	32.1370619
Nubpl	4.37295487	1.88851878	1.61971452	2.50921177	1.18269138
Cdc20	17.8016215	1.63602178	1.25237911	3.38948939	2.57772114
Vnn1	1.94339472	0.06358189	0.04998757	0.14868326	0.47585415
Arhgef39	6.2316966	1.57844055	0.40951591	1.29188825	1.99750995
Rgn	1.6572221	0	0.46036835	0.15214687	0.73040891
2210417A02Rik	2.25276746	0.55859576	0.10979088	0.21770834	0.19002705
Mir25	8.53962601	0	1.43773767	5.70188503	3.11056177
Dhfr	8.81405634	1.74231192	2.02044269	2.53512922	2.18315572
Tk1	36.5356946	3.44106958	3.30816191	10.4083351	2.97741427
Adat2	2.51332045	1.37438393	0.6649413	0.8652895	0.86316484
Pglyrp1	35.3349683	4.88737114	2.83747346	5.62652972	5.21807014
Dyrk3	6.29456463	2.24885118	0.67029662	1.18468219	2.2698694
Adtrp	2.7955128	1.39685222	0.84717824	0.37331126	1.20155449
Tesc	1.79989801	0	0	0.12711209	0.61022486
Tuba1a	229.900922	31.7655258	29.9078352	52.7200375	73.3115028
Map7d2	3.09645542	1.83348116	0.5327165	0.1398101	0.28474473
Tbc1d22b	6.42590123	3.15353523	3.32108753	3.30990261	2.15556317
Wnt4	7.3859957	0.81702362	0.39487956	0.75170025	3.11658274
Tcf19	26.3347827	1.93497404	2.86087901	6.68596671	0.79579854
Nxnl2	2.74752604	0.17611216	0.20768696	0.10295751	0.26959985
Arg2	13.0239295	7.51627325	2.85518264	10.7317766	8.29775828
Zfp934	14.1973789	5.47946027	4.3079077	2.7983421	3.23529032
Wdr76	2.64288657	0.43434638	1.05289554	0.74766706	0.35708428
Pbx3	6.54024467	1.7546085	1.96825265	1.75131028	1.31025753
Rpa2	16.5773824	5.02851123	4.03244268	8.93679286	4.39877422
Fam111a	15.6556979	2.673076	3.05794532	4.02375913	1.99293761
Ldoc1l	2.16586497	0.98336918	0.66851878	1.01451298	0.38962843
Blm	1.6349069	0.55904752	0.29301245	0.58102508	0.86637887
Hist1h2ae	42.9804317	3.85489263	6.26151092	2.04101566	2.9691726
Macc1	21.6176067	4.83062373	5.47515823	7.06011707	2.21847613
Kctd17	6.33887452	2.86277371	2.45529787	5.43925055	3.78484619
Adra2a	1.90436662	0.68703899	0.09531962	0.33077234	0.74241032
Tead2	16.5881272	1.96102768	2.91218353	2.46272907	6.64645425
Cdh4	3.87589578	0.22603328	0	0	0.23068079
Upk1a	2.09395333	0.31317805	0.27699533	0.27463208	0.43947386
Tnfsf13	2.01965924	0.15248544	0.39561334	0.07131601	0.21786901
Bub1	6.52567979	0.87367795	0.41769644	1.14576731	1.0964784
Xlr4a	3.50423856	0.25795774	0.45630969	1.15617568	1.27243131
Chst7	5.73387521	1.25970343	0.60522533	4.82776917	2.9521286
Ccnb1	54.4838598	5.23985014	3.51984999	13.1837627	7.91984483
Cdk3‐ps	2.05643029	0.71765398	0.38084432	0.83910011	0.9887532
Fam132b	1.5752735	0.12732941	0.17160919	0.34029012	0.7982486
Ckap2l	17.5242124	2.28519146	0.7507218	5.40106833	1.54923229
D7Ertd715e	13.5002768	4.92666349	5.45199564	3.74015577	4.43223138
Ipo9	8.13577525	3.27624118	4.37684604	5.60679265	3.69556287
5033406O09Rik	10.7491796	3.52420403	1.64146568	2.52775875	3.17352861
AA986860	3.65400667	1.00332171	0.29061181	1.13194864	0.43113733
Pemt	9.07021101	2.210271	2.02731355	1.07679483	1.94242274
4632434I11Rik	7.37389357	1.23223623	1.99590837	3.2286986	1.54545046
Galnt12	16.3617777	6.12329515	5.47715767	11.4552311	5.40024965
Plch1	2.19242615	0.39162227	0.11545886	0.09539483	0.18318414
Oip5	13.4025309	0.82216782	1.55131617	2.30712111	1.44320477
Vcam1	5.75735929	0.34658801	0.10662445	0.10571476	0
Stmn1	236.054012	46.422294	37.9399982	55.8596463	24.9455316
Tcf15	10.3666273	1.15499124	0.12972069	0.06430697	1.01034788
Cep55	19.8123348	1.47457485	0.56515822	2.49996255	1.95636196
Bak1	7.71986534	3.01805403	2.8352589	4.81470362	4.95949708
Dhcr24	5.60159995	0.54662321	0.44973919	0.71344341	2.40010575
Cenpm	12.7182437	0.96394224	1.31864795	3.87711008	2.91193553
Ptp4a3	4.02366995	0.87576625	1.21580501	2.44974904	1.93462261
Lig1	4.28073324	1.23682637	0.97810286	1.75236961	1.30680719
Xlr3a	1.82133438	0.248717	0.14665448	0.50891141	1.26915453
Spin4	5.07514346	0.55177383	0.37596014	0.73116845	0.53809143
Kdm8	3.46870689	1.84370123	1.03771246	2.71912725	1.64640863
Ung	5.19330098	0.83021306	0.63350959	0.59955443	0.99680377
Lix1	4.22415391	0.54455436	0.3113631	0.07717666	0.18525034
Poc1a	3.74384627	2.0097885	0.70749833	0.80668145	0.94902201
Odf2l	11.8360226	5.17198282	6.26283869	5.44481475	4.30759839
Mrpl42	142.66281	61.5839247	88.3107695	92.3217375	48.255742
Vrk1	5.85805746	2.07732091	3.11787579	3.91140901	2.10626659
Fndc1	9.14703822	0.13138371	0	0	0.03575603
Bet3l	2.1462075	0.12963193	0.10191558	0	0
Cdca3	44.9645117	5.50587221	2.15080917	6.79972781	4.77621742
Ube2t	21.153004	3.22698201	4.70096246	3.1072367	2.56686209
Tmem218	17.709449	6.62647914	4.85447895	9.33264417	3.02273414
Nudt10	8.67751209	0.29243068	0.79317795	1.23090379	0.98486319
Prkcdbp	38.2626093	14.7724805	17.538718	28.3593755	10.6959668
Acpl2	13.3349545	9.32186518	3.98695715	6.98480916	2.79711285
Avil	5.8911978	1.70513214	1.33392476	0.2171341	5.18611564
Scara3	10.0163623	1.81960587	1.68474987	2.91876229	1.88771361
Phf19	4.84340093	0.64296467	0.66144383	0.28791244	0.37695721
Dnmt1	5.9377395	2.46113536	2.40428783	2.64969419	2.22989384
Tubb4b	197.306118	65.6699143	65.4799649	100.656089	117.546574
Pold1	3.75195761	0.55332086	1.21687026	0.92672284	0.53417358
Neil3	5.73098736	1.20454701	0.75045695	1.88670896	0.13916761
Troap	2.2159312	0.58751982	0.05329654	0.55483921	0.27673842
Naglu	6.00252936	3.70029288	1.95026183	4.83405674	2.08860974
Bmp3	1.55066357	0.39489418	0.03880783	0.57715096	0.03358447
Spred2	4.38226187	2.26427077	1.70305834	2.64744557	1.20090212
2510049J12Rik	3.95533682	0.68568602	0.47565957	0	0.94676728
Padi2	2.83707855	0.68875476	0.82493145	0.52848493	0.36244125
Clca3	19.9967702	1.46548122	0.26746329	0.02039857	0
Sgk1	216.044116	62.8817758	6.98705229	80.4216798	89.379452
Rad54l	3.96442976	1.26513345	0.80487135	0.73961383	0.67955056
Hirip3	4.33705781	1.50901683	1.29860176	2.19355673	0.93651322
4931417G12Rik	1.72873404	0.1885295	0.48912725	0.30860718	0.46177412
Ncapd2	3.75610086	0.83941986	0.58859974	1.36610294	0.86825605
Hist1h4h	15.6890048	5.67749877	3.28453877	3.63226776	2.07717849
Tspyl5	2.42368215	0.6003035	0.94892838	0.44801541	0.45622607
Chac2	67.9124749	28.0699979	28.5555487	24.7503887	14.5203272
Mir1898	2.35704902	3.08461515	0	1.44264561	1.25921537
Melk	14.0538576	1.19489142	0.60094869	1.49982666	1.14772684
Eno3	1.74819367	1.24499991	0.30117198	1.19440983	0.87964515
Ap1s2	41.5151051	17.2558254	13.1238717	12.5606224	7.50051791
Mki67	14.712275	2.75971256	1.5942834	4.6172535	1.24484219
Ctnnd2	3.15858839	0.03444643	0	0.0503446	0.11425272
Dbil5	2.26823268	1.51387547	0.31505208	0.20824276	0.2726475
Fastkd1	2.14632312	0.43029085	0.80344164	0.48214469	0.3293536
Gpsm2	7.59058084	3.08284645	1.67928419	3.99932962	1.82781175
Tcf7	12.5295025	3.50058336	2.15853376	11.3158724	8.26592178
Hspb2	3.80401522	0.24383148	0.07188688	0.14254713	0.68432359
2010001M06Rik	4.95608841	1.3313199	1.57000953	1.55661461	2.29932726
Arsa	3.21295062	2.24414367	0.9513532	1.01183551	1.4520113
Cep152	1.57150223	0.31070759	0.43969647	0.68505658	0.49829388
Calml4	7.5827546	0.71448295	0.42129057	0.55692831	0.12152893
Prkcq	2.77538431	0.57185953	0.14581342	0.23492524	0.0630938
Mis18a	38.123757	13.1556464	15.5142954	13.4937302	14.4310924
BC030867	2.56436058	0.34230373	0.14247341	0.30605871	0.80143141
Mir2861	3.18105803	0	0	0.73011942	1.27457165
Slbp	60.6136857	29.259778	22.8063452	30.9810577	31.9119607
A330040F15Rik	4.54005434	3.84266072	0.05459763	1.29916368	0.09449808
Rrm1	26.9200119	5.89295004	8.53578055	10.7397151	7.63531239
Mcm8	3.57296358	1.41077202	1.4746489	1.2371341	0.76897294
Dlgap5	4.224239	0.88105101	0.79453924	1.31293405	1.23415001
Gusb	24.2950716	10.382694	10.5231158	18.0145671	7.68116246
Ccne1	7.70138051	1.1780202	1.57445505	2.93839474	3.44642611
Kcnq1	9.7096309	4.64881749	1.41542876	3.85427851	1.4664517
Dmd	1.5959257	0.34747571	0.23976134	0.70882515	0.19240033
Car13	11.8283056	3.00679537	5.06929166	3.07291672	5.13709479
Dctpp1	74.571727	26.4099495	27.1446686	53.0706996	32.6402923
Ahcy	11.9849898	4.01012109	5.27566549	7.53968268	6.51932615
Ccdc69	1.51655092	0.07217	0.46810064	0.29534077	0.51557726
Gins2	30.4698164	5.57429779	8.20258427	8.88241627	6.24269969
Stra6	40.806546	18.3476162	5.83622012	3.38637488	27.4918316
BC052040	7.62764888	4.02982141	3.46614157	4.14981957	3.47125218
Msx2	37.9747998	1.23156328	0.36309194	0.94152311	0.70095546
Prc1	32.1618595	4.01338038	1.70465741	7.21777973	2.86365899
Mir135a‐2	1.95635069	0	1.20769964	0	0
Lpxn	6.78232754	3.55034963	1.94906696	1.53879324	0.71842263
Cysltr1	1.53912848	0.66934133	0.08770513	0.08695685	0.15180084
Lbp	87.887279	23.4425865	59.308814	55.1513418	23.614153
Tex9	3.94261083	1.897785	1.59459994	1.6642055	1.13787333
Prim1	13.3996622	5.37765327	4.6874187	2.53851046	2.55662611
Zfp36l2	12.6436477	4.76827546	9.1611521	6.89697302	6.78917744
Wee1	19.4547889	9.76467	5.36912388	6.44401396	11.7384358
Dmpk	4.86724929	2.28194464	0.43757233	1.0845977	1.43897292
Hmmr	14.2785943	3.82006811	1.51193874	4.32885829	2.55012024
Hist1h3d	4.60131143	0.24917086	1.32229888	1.31101736	0.2542941
Asprv1	11.5430228	3.71241795	1.10436283	2.07256631	6.51937988
Slc6a9	1.89337118	0.29733729	0.12918548	1.20764252	1.13394807
A930001C03Rik	2.35046324	0.34336705	0.15184824	1.35497436	0.13141015
Sema3f	5.57364868	1.90149751	1.05001885	1.3233818	5.22112331
Rnf122	3.62185025	1.34255113	1.6683756	1.83981038	1.48801838
Glis2	2.43259964	0.43861369	0.83427718	1.8693801	0.77974624
Aaas	3.87822823	1.71997869	1.29681404	2.7033717	2.10065691
Pigt	8.95162418	3.54447252	1.86562909	3.30138474	2.28894526
Smim1	5.32378988	1.99343926	0.85963513	0.99145212	0.45546866
H2‐Q1	3.80186728	1.59381943	0.74586193	1.39025705	0.87784233
Calcb	1.83026572	0	0.75324302	0	0
6720489N17Rik	6.6497136	1.88280793	3.09020947	1.06667182	1.01028405
Hist1h4i	20.6442965	7.34218825	4.83529651	4.12510677	5.54687886
Ccdc80	11.011623	2.14756183	2.0856655	1.73963762	3.28041481
Pctp	21.3732884	6.57268733	4.90187576	11.299626	4.4011845
Papss1	21.544939	13.4707517	9.35967222	13.1253007	6.91406099
Zfp286	2.32124111	0.63026302	0.40176302	0.69708674	0
Ltc4s	1.55265928	0.69666138	0.10269555	0.20363875	0.53323916
2900026A02Rik	5.08244583	1.44720125	1.5308286	3.70212319	1.23527345
Xlr4b	2.02520775	0.03975513	0	0.69724138	1.58232925
5830416I19Rik	1.95883022	0.19469434	1.14800346	0.09105672	0.35765547
Thbs3	3.33946132	0.62738921	0.70193036	0.94046172	0.50894771
Gclc	68.7510459	28.8371463	15.8935669	51.6064686	7.42613719
4930415O20Rik	2.53472998	0.09046751	0.2133745	0	0.09232763
Greb1	2.41583257	0.19837138	0.15352114	0.60159719	0.34796115
Crybb1	2.61444345	0.70384346	0.55335608	0	0
Tmem237	8.5978901	3.84294245	2.78652808	7.77214349	5.82993727
Sod3	1.94518976	0.12519465	0.70867461	0	0.63884398
Eme1	3.74645254	0.25280553	0.1084111	0.48368773	1.10237862
Pmp2	4.82872481	0.90096091	0	0	0
Slc35f2	6.22152022	2.25316242	1.50492167	3.45043977	1.44481234
Zfp658	1.83233082	0.27260663	0.44650238	0.65518555	0.46368623
Scx	29.391184	0.67611723	0.79733691	0.26351141	0.87402405
Cmc2	16.031207	5.01378488	4.52887365	3.90816703	3.95559772
Mir3094	3.05679795	0	0	0	0
Lxn	44.2507893	21.2883641	21.1789818	20.8338107	27.7079271
Lsm2	61.5345546	24.3616654	31.0171317	44.5726651	41.8059502
Mdk	39.3268963	12.2812593	11.8456363	21.1035285	7.56831856
Fam129a	4.22162499	0.23398909	1.14975213	1.10954427	2.98500215
Ect2	22.5397703	2.82328074	2.4200928	2.41398727	2.11974547
Cdc42ep3	17.1400323	7.75748563	1.62704832	2.83065111	8.47489712
Tmsb10	179.868085	89.8659937	47.257812	42.283438	154.167754
Mbnl3	7.82901266	3.80299502	3.02238291	3.908004	3.83900217
Clhc1	4.41590969	0.72573465	0.66566122	1.2256808	0.61721383
Arhgap11a	4.88730223	1.63299539	0.67402013	2.57761117	0.90619834
Snora68	25.2861653	9.92742468	8.62642601	25.6584826	10.6647832
Cd79b	2.72168988	0.81209317	0.7560724	1.3493192	0.61068792
Ankrd9	7.82642247	2.32202304	1.88850608	2.99583013	4.06537533
Epha4	11.387313	8.81191877	3.54026997	4.2385694	3.22892828
Gdpd1	16.3029224	8.53410192	6.07739352	6.55579051	12.9170961
Cdk1	35.1139867	3.38380125	3.20927177	7.85006024	4.9516663
Slc9a3r1	56.4579872	30.9572105	21.6468536	22.3655105	45.650488
Mir186	3.673898	0	0	2.52970955	0
Aspm	3.53621061	0.5034307	0.24482052	1.02554176	0.20127535
Tpx2	13.9984772	1.31384112	0.84386802	2.9214813	2.89720502
Gm15441	2.22179695	0.30606462	0.50531366	0.64414601	0.31235767
Wisp1	9.4921834	6.66822301	0.79358997	0.87026979	7.33602023
Kif20a	4.45877621	1.6259595	0.95873723	2.37639382	0.95013523
Prnp	107.423282	53.1487939	46.8218411	25.9088856	31.6030799
Xpnpep3	4.61479509	2.76909982	1.86603776	2.3866512	1.90555552
Cenph	12.8949669	1.94238639	2.48558037	1.93284238	2.10885544
Serpinb11	2.07264934	0	1.06624453	0	0.03075776
Msantd3	28.9751016	19.6641284	7.41282235	7.21949701	7.26665076
Ttll5	2.81537763	1.38562365	1.24736588	1.36039604	1.71636699
Mcm3	21.4434835	2.92749858	6.23517833	6.49315493	8.27499966
Mcm5	14.1018849	2.03501671	2.73514676	3.32415565	4.04682086
Gemin8	7.31677998	2.84470064	2.99293756	3.22827314	0.99619298
Usp51	5.97846375	1.86861511	0.0806208	1.33221613	3.814072
Mad2l1	54.6644076	8.22242168	12.7421064	10.7939163	9.3003092
Kpna2	65.915643	15.5020831	20.5925233	34.5470443	33.4621964
2700099C18Rik	4.75661735	0.72288863	0.42624693	1.45565771	0.61479339
Slc35e4	3.96514359	3.53192973	0.59220316	2.30945915	3.31413629
Tmem45b	207.228258	86.9665624	34.6974024	221.727302	89.3519236
1600014C10Rik	11.7381041	8.7864145	5.28587379	6.26463629	9.1185442
Egfl6	6.98006603	0.1327527	0.17891847	0	0.01935461
Lmnb1	13.1203259	3.40403503	3.33466134	4.19067491	3.62107465
Oit1	27.210633	6.50802017	10.5709921	2.6800227	10.1089528
Klhl23	3.53106398	0.93159787	0.71933594	1.25782324	0.11318483
Fam83d	12.3786791	1.65395427	0.24047117	1.82788305	1.06364696
2810417H13Rik	108.26725	6.30672635	9.33475424	16.2528805	4.5536435
Slc6a14	192.404886	172.726045	36.4386066	76.7038109	187.499443
Fam184b	1.69237225	0.31048152	0.20504238	0.10164651	0.46896076
Bora	6.39887926	2.34473799	1.52740033	3.10706731	1.64087899
Hist1h3g	2.12070535	0.52037207	0	0	0
Fgd2	3.57732697	1.32644327	0.39106465	0.61580358	0.0199076
Snord17	5.31135933	1.62186552	0.54647043	1.62542424	2.36458994
Dut	30.0877182	5.56483238	10.168331	11.1302521	9.17417597
Bend5	11.0395051	6.38715804	2.75340327	1.09824043	4.43695226
Cyp21a1	2.98420628	0.55917673	0.31401447	1.43214273	0.70654882
Zfp367	14.7053294	4.19613722	5.83087076	5.76E+00	2.75511992
Arsb	9.61255288	2.75983742	3.54226267	3.79720671	0.64191889
Mxra7	6.46446314	3.23553777	2.1003472	3.47071263	1.21176667
Hist1h1e	11.9211426	4.05712354	3.59620556	5.27077409	8.30814779
Cox18	12.6121559	5.18187598	6.65411721	8.34766226	7.91304529
Gprasp2	5.39730065	3.03096967	0.99639965	1.14470793	1.98463291
Noxo1	1.95227131	0.38895371	0.4856701	0.16050883	0.46700123
Parp1	8.26435052	3.13088035	3.39241472	5.91345223	2.44423343
E2f7	3.37014393	0.22441758	0.22054413	0.14213063	0.65846662
Ube2c	171.749799	20.8448419	10.312795	25.3369478	23.0134795
Tbc1d7	10.5497103	4.83215604	2.58587552	3.03859377	2.11350462
S100a13	34.1107299	18.5482439	16.9753586	14.4859778	25.14204
Mtfr2	5.00323756	0.68805503	0.65436695	0.64878406	1.35910111
Ctsh	95.3866611	42.5600869	37.3918096	47.1064235	26.4989964
Tspan8	515.352301	160.039643	220.194908	216.821483	143.931129
Glrp1	2.29923688	0.1569891	0	0.12237055	0.05340566
Fam84b	7.86572779	2.53123641	2.33377155	7.89941181	3.42871917
Gpha2	1.88537198	0.26124802	0.41078219	0.4072775	0.35549277
Anxa9	2.90269534	0.62160378	0.85522483	0.72679566	2.27321176
Kifc1	2.10632072	0.02205194	0.10402236	0.07735115	0.04501071
Gbp4	1.83937776	0.63187709	0.50564771	0.30343879	0.12667074
Nek2	10.3416724	1.1051355	0.83107263	2.20977027	1.99418436
Ptprs	21.2917054	4.68965842	3.11575083	3.28653155	1.69273053
Pxmp2	5.82361506	1.75874524	0.2962953	0.82254867	2.35902074
Fam84a	109.104173	52.5226001	19.0444943	25.6483609	18.9703775
Nfic	7.98700757	3.49769388	2.70509372	3.61406215	1.5523655
Mir181a‐2	2.57414564	0	0	0.78776043	0.68759786
Hist2h2be	3.11362154	0.88014038	1.24552675	1.23490023	0.27945154
Pkmyt1	3.77389866	1.55698181	1.00690995	2.52516046	2.28097693
Fxn	13.3996622	3.08630535	3.58449665	9.40420489	6.77676361
Ncapg	8.02361242	1.05233923	0.71848005	1.27899233	1.03158273
Uevld	8.54956815	3.22321193	3.57827222	6.19880425	4.85482599
Ppil1	147.222769	90.7775582	62.6711624	84.3597409	54.3219291
Chek1	9.69440778	1.08529056	1.93758111	2.18541487	0.95377131
Ccne2	2.12845117	0.55709089	0.69451176	0.74441769	0.47108041
Cdc25b	11.4239456	3.73756289	3.35365657	3.43904554	2.3218157
Apol7a	2.07194331	0.12843966	0.50489115	0.35040849	0.28400781
Alg6	10.6801418	3.35444469	3.64559301	4.01823593	2.2151515
Loxl4	1.89506105	1.21489698	0.27765763	0.29731183	0.74008142
Dek	76.2640935	33.8477832	36.2263478	30.9703079	33.1981339
Nudt16	7.06527645	3.82599837	2.18077768	6.63563083	4.78321503
H2‐Q4	2.94598263	1.8277162	0.67356366	0.63442614	0.49546929
Ldhb	581.091761	103.361867	69.2320841	224.024411	20.1738015
H1fx	23.7021772	4.48401965	1.93322918	9.75280052	2.85398436
Chtf8	25.3638096	7.96632646	5.78921186	17.8118251	15.707541
Cacnb3	13.6693734	4.43116831	1.83864529	12.74152	7.70462222
Racgap1	14.8295425	1.77086095	1.79283307	1.9142707	1.415128
Emid1	24.1259364	4.17996829	2.78067945	2.66296828	0.65629435
Akr1c12	1.96005891	0.24957569	0.68675041	0.04863509	0.16980483
Gpd1	2.43519415	0.49641814	0.3252333	0.27946402	0.4315695
Mtap	26.3345095	10.5291734	10.3631771	17.0621888	4.39223968
Cdc45	2.86042875	0.88407402	0.53537782	1.28511921	1.31679964
Rad51ap1	8.56458702	1.86959617	1.25047919	1.40294337	1.62325556
Chchd6	5.67561774	1.40971844	1.39432463	2.71168688	1.25306467
Dnph1	26.5327851	4.58341459	8.68102364	19.8663779	3.02393853
Wdhd1	4.32765645	0.49070064	1.18628858	1.27657201	0.88890227
Fut2	31.6358769	10.6627452	14.430017	1.37001814	16.1948516
Spc25	110.554837	15.8274684	15.4206907	16.8785889	6.57347036
Cdc6	4.24479409	0.45747579	0.48811515	0.57309948	1.14497258
Clspn	3.04984417	0.39056107	0.55754901	1.20172206	0.39859146
Stc2	4.80086058	0.42837098	0.23574728	1.90327841	4.63409514
Suv39h1	14.168265	5.80077916	5.61991168	5.74488705	3.32059457
Zfp947	12.3114961	7.10586749	4.04231922	4.09789481	2.27975283
Pck2	9.64749407	4.36745216	1.86483033	5.8813267	2.09029751
Slc7a1	5.08685696	1.71894573	2.08601136	3.3358291	2.50404772
Snta1	4.34744597	0.38736349	0.39971147	0.82090969	0.81536428
Psat1	85.5527103	32.9921323	15.1750296	12.257565	26.978348
Rad18	4.17683165	1.17537427	0.44713056	1.6402683	1.56714271
Ndc80	10.6957837	1.32606218	1.72842066	2.20329542	1.0446739
Akr1c14	4.40078886	0.31413872	0.14818401	0.12243311	0
Gsg2	9.32589797	1.29332174	1.97631389	0.93712945	0.83656517
2010300C02Rik	2.43740092	1.26808027	0.26308522	0.49422438	0.58716222
Cldn9	3.38938095	0.39033314	0.16738734	0	0
Lce3c	13.2967098	0	0	0	8.80222798
2700094K13Rik	88.549599	39.5157827	47.4008769	53.0804348	29.6305059
Hs3st6	3.26058448	2.14052065	0.24747944	0.53980961	0.47117362
Cd81	174.885895	59.5025921	51.7528733	93.3542816	90.4349729
Trim59	55.4299361	22.8667169	14.9928466	14.3728509	7.14065673
Pir	3.99172116	0.76758943	0.79205785	0.41134773	0.48960747
1700013G23Rik	1.84910273	0.58077064	0	0.22635082	0.29635598
Dsel	5.02411767	3.41186376	1.42501433	1.28819266	2.05837903
Cenpf	6.06852173	1.05889774	0.46149285	1.59875278	0.64840194
Skp2	2.97803907	0.61971048	1.02314396	1.43950284	0.68304865
Mex3b	4.56252746	1.93366361	1.4495224	0.66599887	1.75925215
Abcb7	13.4635699	4.99687301	7.41312421	8.22313007	3.72943339
Efna4	52.3818208	29.5485258	17.3663576	10.9161088	14.5705262
Pole2	2.88547299	0.60418421	0.99751003	1.94267764	0.89408006
Foxm1	4.84045531	0.68038201	0.19367463	1.67333671	0.7781749
Zfp418	3.86439642	2.27576051	0.93932194	1.79609378	0.46451056
Kitl	3.42211802	1.50325465	0.72018786	0.7140434	0.41230639
Cep78	12.7660389	4.64035514	5.49600464	3.47615902	2.41913599
Col12a1	5.70511393	0.80810091	0.00517926	0.13864692	0.07619662
Nrm	33.2739973	3.56753441	6.4344653	10.2236668	10.8155762
Them4	60.3490838	46.2321406	36.0564662	14.8463243	56.4259501
Rrm2	42.3576846	4.01650852	5.09671408	11.9190321	23.3001053
Mir3061	7.16611973	1.12537608	0	2.63163924	1.72277267
Cdk18	3.27557075	1.09267884	0.69385239	0.15724174	2.02441814
Fignl1	19.1425034	2.04887316	1.74616962	1.77153388	3.7778578
Cdca2	10.0172585	0.70321694	0.95215384	1.73579765	0.87715943
Vpreb1	2.37750951	0.33336336	0	0	0.27217416
Pask	1.75800218	0.64018268	0.60160853	1.04090869	0.47980066
Nusap1	36.0206461	2.43913859	1.65242687	3.96435114	1.21816324
Mns1	2.74271633	0.70842019	0.26733805	1.02709661	0.69406669
Impa2	6.2106371	1.58859906	1.82984794	4.10362938	2.60156075
Csda	125.940513	57.99706	50.0073267	90.3512985	91.9416573
Iffo2	4.36563612	2.07653075	1.54509368	1.17542883	1.74920294
Plk4	12.2444278	2.51186986	2.07695738	3.57834679	3.65374811
Gm6880	2.49614123	0	1.15569344	0	0.16669039
Nasp	22.3021911	6.54055735	11.3688366	9.94350917	8.57982654
Sgpp2	6.91589704	1.76744282	1.13278481	5.06152825	2.44425734
Fam46c	2.0248085	0.55520032	0.53569723	0.50161975	0.0772658
1190002F15Rik	35.8825442	6.07369362	3.08391177	8.0878468	0.51654798
Mb21d1	3.92900429	0.94728531	1.34356585	1.39197268	0.37886642
Lsm6	16.9334221	8.16363972	8.84883846	9.23012328	6.33798442
Uchl5	19.6586293	7.63504742	8.90605084	13.4876843	11.0669452
Anxa2	203.940779	121.042633	120.769964	41.2203959	201.841501
Spire2	5.92579433	2.21674486	2.0188721	0.8981752	4.25585648
Pigz	2.7768714	0.5392427	0.13824401	0.05482582	0.23927398
Rbm28	11.9331472	6.22626547	3.70734362	7.7089867	5.20576959
Fkbp10	7.01470345	3.02939308	1.83265985	4.71506244	3.61365301
Ubd	33.4757979	1.31153754	0.83778524	1.53348455	0.16731303
Tmem191c	1.8062897	0.3273022	0.42887061	0.17008464	0.14845863
Anp32a	32.7617045	14.1473544	11.8633975	21.0631776	9.66712638
Pf4	2.43989315	0.9579094	0.2053911	0.91637438	0.35549277
Ccnb2	53.4050306	8.34027092	3.91855821	9.7905177	5.01888435
Zdhhc21	8.07787355	4.31239843	3.42947504	3.81070444	4.11442973
Txndc5	191.897295	107.398591	53.5319673	89.3128281	81.4513139
Chek2	5.57216039	2.64340674	1.28993286	1.83845826	0.6976961
Prdx4	60.2622895	19.5890463	22.0482601	29.5971694	30.3361126
Focad	3.80289011	1.192654	1.28146183	1.99359386	1.46061161
Isoc1	24.4766466	11.0480492	10.060237	15.4404733	9.97030317
Fancb	3.30911821	0.7621796	1.2665321	0.38481937	0.74249404
Tstd2	7.87580792	6.21161686	1.86373401	1.63894763	1.61288388
Mybl2	1.77914118	0.20747412	0.34254042	0.66306362	0.9034241
Gmnn	33.8406618	9.06693735	8.77652758	5.20873325	12.4091357
Dsn1	9.59751541	2.1307219	1.2960421	5.74309445	3.79970857
Spink2	20.2454592	0.58567366	0.19733654	0.58695875	2.98857895
Mal2	181.112249	32.4203613	76.9663717	104.33525	131.65108
Tspan12	7.25447309	3.41364077	1.35805582	1.65904245	2.1196792
Cytl1	4.08409965	0.15771441	0	0	0
Gdf15	8.84328262	0.51960399	0	0	1.25340718
Apitd1	21.9475901	4.90022627	8.50584156	6.24448376	7.41718471
Snora30	3.65673025	0	0	1.67859232	0.97677454
Dclk1	4.20024503	0.20883759	0.36709608	2.096249	1.28683186
Gsdmc2	10.1822751	0.90804026	2.19391803	0.15537144	0
Neurl1b	1.55968488	0.2730692	0	0.06772601	0
Ckap2	12.5108163	1.79147238	1.6715566	4.58058007	4.50044831
Cchcr1	3.3353854	1.07839723	0.95380566	1.6436611	1.08091729
Xrcc3	3.95793491	1.05707291	1.24659335	0.71685549	1.1003837
Slfn9	11.7536629	0.59756419	2.20806081	0.93158391	0.35235824
Tgtp1	3.08763073	0.67446445	1.09634535	0.06394068	0
Arhgap19	2.9687812	0.48813735	0.49341814	1.01336031	0.4270063
4930579G24Rik	21.1300447	6.03325032	5.88822578	17.0882801	14.2520285
Nup210	3.75419108	0.29114207	0.33475683	2.3573465	1.14394391
Mcm6	35.5843173	9.42546107	13.8213127	13.0223507	10.8261703
Prr11	3.56133868	0.64223187	0.34713081	0.79784673	0.28675364
Rfc4	15.1767337	2.56865239	4.07201359	3.8403321	2.57849199
Cxcr6	3.82382332	1.97460995	0.60608276	1.70785463	0.63492925
Cdca8	34.8116522	4.50323459	3.63943926	9.38917414	7.13477932
Tmem54	35.7324326	10.0830999	9.30590477	12.2507538	23.4442614
Dnajc19	16.1904884	7.92031922	8.6859186	7.66805228	5.76633795
Elovl6	9.47098668	1.4201985	2.13159291	4.53879249	2.03794344
Mboat1	46.5151066	23.016304	17.0612202	20.5523169	8.97862952
Mvk	5.26724919	2.77782703	1.52873372	2.02092128	4.15791548
Gins4	24.6217156	9.98565005	11.6431454	12.8605933	11.3403237
Trim2	10.5887818	4.01726731	2.21475159	4.86432978	0.92024878
Ccp110	7.30506346	4.17162171	2.11009821	2.42683066	1.82608858
Me2	36.0457282	14.4749493	13.1100592	7.92573632	5.76853997
Slc6a15	3.29279662	1.4894865	0.53027427	2.90805525	1.11857304
Icam2	20.3286878	2.91444812	0.89890078	2.51641861	1.37278733
Spc24	47.7123333	4.21327789	4.25246352	5.90265563	3.91252869
Ubqln2	27.4802491	17.0682038	12.1423363	10.4371746	11.1049019
Ptges3l	2.94590795	1.22666763	0.78529409	0.90153008	0.2146096
Dcaf4	7.74762734	3.19441613	3.15774677	4.22933399	1.07870858
Hist1h2ag	15.1655092	3.46415977	1.53196572	1.89861922	1.54673177
Rad51	20.4358428	1.79623287	2.65490567	2.85627636	3.03083362
Pmf1	23.9284829	9.91056997	13.3309587	17.2608475	8.79737106
Ppp1r1b	2.70084428	0.21514543	0.2899639	0	0
Mir3112	12.7242321	0.62444648	4.41841332	1.46023885	10.833859
Wdr46	7.92545025	3.14704868	3.48804194	6.4739055	4.66066797
Mcm4	20.3139228	5.57843497	11.0372104	9.15812656	9.58077292
Igsf9	3.23833055	0.79536464	0.49740512	1.70492938	1.38976005
Rbp1	60.7682588	7.15344211	10.1369536	8.50073571	22.737058
Kif20b	5.81430847	1.14135751	0.65128508	1.85108833	0.92998137
Cdca7l	5.07007774	1.76500001	0.78632301	2.77450974	0.70050185
Slc27a3	19.0596638	1.15295152	1.41298192	1.85028057	0.73829493
Fam101b	6.25928208	2.46470503	1.80587386	3.32515226	3.39353341
Dtl	10.0874817	1.18201983	0.97719628	1.19682594	1.13170558
Mogat2	17.3333554	6.4439212	6.5769196	7.36547112	7.43164464
Samd12	5.09684511	0.82234387	0.96978023	0.3205021	0.89520236
Gm7030	1.8859888	0.85435948	0.26867623	0.86574784	1.10443973
Atoh8	12.8761935	1.4350337	1.46154734	2.82188832	3.35068921
Lin9	5.7738511	2.33506372	2.91464379	3.45709493	2.46044791
Slc25a40	5.25446707	3.11279833	1.28155178	3.51035116	3.4775633
Cpn1	1.92977082	0.22871965	0.16857896	0	0.58355598
Slc26a9	1.79152993	0	0.03317856	0.27961167	0.54554468
Hacl1	3.88579968	2.09277824	0.64582869	2.4011949	2.21565148
Clic6	2.43912277	0.10944079	0.11292944	0.4478638	0.68410752
App	140.699514	64.0324573	59.809373	59.709284	61.2238613
Spdl1	6.02753465	1.22401462	1.20288809	2.43295572	1.97787115
D630039A03Rik	3.44523084	0.08840575	0	0.39279215	0.10826817
Dusp3	6.73373144	2.55840315	2.6866504	3.56113447	1.28063671
Ercc6l	4.84660484	0.21955288	0.53221637	0.85569023	0.93361311
Xrcc5	5.97008425	1.84304549	1.748235	2.43601661	1.96272067
Dusp8	3.77548483	0.12750681	0.12530604	0.45967677	2.96042343
Lpl	5.97518815	1.40373225	0.93955393	0.34008526	0.41300025
Dact1	5.1149823	1.53845283	0.94727837	4.13883173	1.04209195
Cotl1	20.4109803	6.21703337	8.47379248	7.41523364	7.90716568
Ska1	5.64402409	0.42206874	0.38461772	0.47106244	0.50906459
Snora69	7.48330864	1.25912979	4.94958869	0.98147201	3.85505688
Il17d	6.08357824	1.92750584	4.20026471	2.1067112	2.95070638
Aurkb	23.5255252	2.06702315	2.83874213	6.57741719	3.44466503
Cks1b	166.259658	37.8792371	64.3734221	94.5895273	69.3084114
Robo1	5.57504798	1.71854801	2.31390655	1.4714299	1.13933367
Neurl3	3.50926243	1.48677734	0.6779576	1.80791477	0.44508851
Atad5	2.90547132	0.61259643	1.2953874	1.54778892	1.00605628
Bub1b	6.98506241	0.97692091	0.49374474	2.333437	1.05397939
Lbr	51.64428	22.3587704	22.440365	26.2773105	18.8315091
Spink12	27.6655653	1.6091236	0.20331644	0	0
Hist1h4d	61.4494767	27.8999486	33.4826984	27.6322121	25.9612271
Ccdc18	2.50652618	0.80264905	0.80392289	0.69421705	0.33663799
Mir466g	1.84561386	0.48306237	0	0	0
Efcab11	5.04295328	0.27223331	0.23348471	1.04171703	0.07577202
Slc7a5	12.604252	5.43675494	4.86448902	7.02145022	2.66270463
Cdc7	6.47891516	0.96373999	1.90042214	2.30907871	1.06417491
Trip13	9.46389319	1.01641267	1.94446568	2.29760563	3.52685839
Cenpa	67.9430692	8.73197564	5.46490862	15.1353702	10.6703669
Chaf1a	6.30826501	1.62529752	1.51510082	2.24421231	2.62235026
Hyls1	9.92265513	5.23577282	5.1821561	3.64393139	6.74289519
A630001G21Rik	4.44625156	2.01009839	0.87333652	3.92740893	1.88947347
Gprc5c	10.8174357	4.04419984	1.87410582	10.9821088	2.11401022
Ncbp1	14.2539212	8.18022884	7.49231857	5.06131646	6.01355278
Zfp820	6.88296588	3.58917304	1.69960437	1.36104536	0.74956541
2310009B15Rik	11.0779928	6.00824689	5.60491077	6.3958973	4.39291946
Siva1	63.2103078	21.2152099	29.0342113	51.75445	27.3984008
Ccnf	9.04841126	0.90876206	0.42867687	1.02391062	1.82116917
Rbl1	7.81576852	3.31908008	2.43101128	3.10681658	1.322118
Suv39h2	8.01059054	2.21224968	2.63708352	1.57993615	2.86793504
Rnaseh2b	8.50392657	3.33866719	3.19124476	4.27347869	5.88210006
Gngt2	5.75943697	1.89646709	2.15046232	2.64382276	0.96773033
Aifm2	2.57686803	0.93045588	0.67926573	0.51805416	0.22609217
A730017L22Rik	2.88304312	1.29359019	0.66105665	1.03354169	1.45220669
Sass6	3.18293321	1.37640314	1.2529777	1.27052142	1.25684005
Mlkl	4.90428688	0.78220911	0.67409792	1.65327865	1.99573058
Hist1h2bn	2.05391148	0.67197653	0.4754723	0.31427713	0.41147589
Haus5	3.59481295	1.30635911	1.37551212	1.66380745	1.07133699
Morn4	3.59095207	1.24064037	0.36576806	3.49460244	2.07188079
Shcbp1	17.7099288	2.42573441	2.26585299	3.56635257	3.06387416
Snord1b	4.23452529	0	0.78422055	0	1.35733604
Pdgfc	9.24698787	1.99744641	3.73107662	3.30715826	3.36280778
Nucks1	58.5685696	34.3715995	29.7071131	34.8356554	22.2111255
Tubb5	250.146617	65.0463118	73.6491312	81.8031876	152.287459
Gtf3a	2.66272692	1.65685184	0.69782337	1.84498591	1.04675915
Haus7	6.11508884	1.80060172	1.99071369	3.72815559	3.29240999
Aurka	17.7663343	3.35988265	1.58490767	2.79706643	3.64841954
Axin2	2.97112912	0.64937741	0.99270755	0.87175368	0.60136554
Phf6	20.6728551	5.47359641	7.17678982	6.50997977	4.60105716
Phgdh	6.44797572	1.23153796	1.25869284	1.66393866	5.9212062
Knstrn	13.1552767	2.6932509	0.96832877	2.76499364	1.726272
Mcm7	22.8077612	7.41569291	6.75424674	10.7195915	8.37514861
Ssbp2	2.49834448	1.23857309	0.68041972	1.08837814	0.47892183
Gm3604	10.5446788	4.08724717	6.16468813	1.76389249	6.03314714
Ehd2	5.33123688	1.20560236	2.84350471	2.99544745	3.21268747
A630089N07Rik	7.9869784	1.53782034	1.72852365	2.72518531	1.15255728
Lgi1	1.83738031	0.38472589	0.17013848	0.1686869	0.01226988
Tmpo	33.0271089	8.60025775	13.3074324	16.1627077	13.6768266
Xpo1	40.6812426	18.6144292	25.5321415	18.7733234	16.2948931
Gm10416	2.43782017	1.35588535	0.0940576	0.74604103	0.7325809
Cenpn	5.31135933	1.3032848	1.12709525	1.04975315	1.62565558
Ly6a	1069.02915	108.08159	87.2629281	121.771096	468.595886
Lamb3	6.14324671	1.98252133	1.83165297	3.38105612	1.22428029
Sesn3	6.34590121	3.51171571	3.49773992	2.08073886	4.93999874
Myo1b	20.4910367	8.17689194	9.69082533	8.25421662	15.3631269
Rab32	131.502833	48.1133886	22.5488224	41.9146845	85.5861833
Zfp36l1	111.716541	47.8069865	48.4051197	35.6931506	64.7789082
P4ha2	19.5023014	12.4297884	9.48023609	3.55029915	19.5073626
Nme1	147.373138	32.9202118	75.6401354	100.445515	83.4426455
Plk1	15.955107	2.51025785	0.54820683	2.90788372	2.06372995
Cpm	25.2687452	7.87535796	6.06755739	7.66379446	4.75799192
Crabp2	28.9829731	0.4551521	0	1.13087386	0.05806382
Inpp5j	3.1920493	2.16731296	0.45206148	1.70662525	0.54168378
Eogt	5.45295875	2.9556484	1.90292767	2.57785691	2.59249067
Tmsb15b1	12.1807712	2.79934095	4.40163878	2.31842022	1.78556165
Klhl31	4.61703348	1.42394157	0.7431107	0.51669631	0.05846285
Wfdc15b	18.4046929	8.11780427	2.41960729	13.7679663	0.81936749
5430435G22Rik	3.07577925	2.19187398	0.64621265	1.42377629	1.26345767
Rmdn2	14.8734764	6.36505715	3.25046796	3.8872174	2.55197254
P2rx6	4.1417484	2.00802398	0.28313504	0.25519946	0.04455025
Lgi3	2.48054525	1.11069418	0	0	0
Hist1h2ab	3.73742885	0.40477954	1.312717	0.47327899	0.41310227
Bmf	4.18041696	2.79619398	0.69078438	1.57654106	1.20689528
Ltf	148.212709	17.4043392	4.09613664	6.13545287	16.65682
Bcam	42.9958252	6.36068918	6.02093714	36.1905062	12.1362309
Sirpa	7.66379988	4.26200597	2.65585402	4.79921016	2.9162344
Il33	1.67851833	0	0.04639645	0.04600061	0.18068265
Shisa2	7.04767147	0.45573081	0.47985523	0.70412662	0.88037006
Afap1l2	2.35242168	0.87958842	0.77055683	0.94028632	0.33342169
Tube1	1.64754695	0.26222915	0.26801119	0.67453164	0.66013151
H2‐Q5	4.3621706	1.51749484	0.56243421	1.31804794	1.81418708
Rfc2	30.9734046	11.6374117	11.8940116	17.5902026	13.4946743
Cenpk	9.28195581	1.35486655	2.34157093	1.58414598	0.7628823
Tubgcp2	4.46018363	2.58927695	1.35058793	2.79457055	2.32067065
E2f1	7.66104808	1.31133761	1.03096311	2.45806873	2.08180036
Unc93b1	12.2020108	4.92612768	3.91729583	9.1416365	2.81350724
Id4	57.2762912	17.2121526	6.76602811	74.2601827	21.6585043
Fam92a	27.6879195	13.2066193	13.9805351	15.9833383	8.27606479
Tst	18.1474136	5.46618572	4.95862079	3.44142057	3.81441151
Gm10509	9.93484781	3.964393	5.06727122	2.39239931	4.15031598
Kif2c	6.45203995	0.36187005	0.53343624	1.73474311	0.75708655
Stc1	34.8794556	36.4164242	6.11432225	0.47985407	32.7813155
Hist1h2bg	8.4828214	1.74845015	2.74923496	2.53108067	2.37920042
Hunk	2.16113804	0.51112609	0.19288475	0.107572	0.20865417
Zwilch	5.01268016	0.84605321	0.73746027	1.5053468	0.84467841
Ift80	4.44842577	1.9279976	1.16636029	2.35673267	1.3160186
Hist1h1b	4.0757306	1.29535476	1.07830325	1.3363793	1.63304493
4930512B01Rik	2.29457036	0.50447893	0.76490477	0.42132155	0.03677511
0610040J01Rik	65.6999054	26.0951844	29.1268737	29.6147371	36.3007549
Slco3a1	10.6054033	2.68042024	0.73118924	0.6245731	0.85667931
Cd276	13.8817977	0.59484164	1.09963233	8.1956765	4.41357951
Galc	6.46130941	1.57595187	1.51614561	4.34799521	2.79345914
Ly6i	2.07964386	0	0	0	0
Cryl1	15.3548092	3.41364077	3.12631467	4.54331052	7.78302264
Ly6c1	11.9719941	2.55244872	4.95560643	4.22182125	1.46130221
Nqo2	8.46381632	3.96218626	2.74680759	3.98145223	1.05935968
Ccdc34	29.861245	6.30513735	6.04140272	5.29872144	3.51901937
Mgst3	25.4685024	8.38626709	7.79774571	21.8736881	13.0849332
Ly6c2	4.72194506	2.04562743	0.30154798	0.74743811	0.52192197
Mre11a	4.94948909	1.36363812	2.33177694	2.0527925	2.52241294
Nsl1	3.55341194	0.6717053	0.24373353	0.44303244	0.19335076
4930520O04Rik	1.55884517	0.20400244	0.43303971	0.14311504	0.16655757
Ttyh3	3.6539826	1.30814701	1.38711745	2.5963285	1.33504403
Serpinb8	9.87394995	5.18429177	2.42347754	1.92952205	1.25519537
Gfer	27.1501062	12.0583254	16.0573023	15.5492814	9.95099377
Dynlt1a	10.7809648	5.57469561	3.00301658	8.69077637	4.84645592
Htra1	12.8888234	4.62072022	5.30108474	4.75669761	6.97107555
Cenpe	4.23170698	0.79300627	0.61057386	1.20306636	1.1169835
Dscc1	7.17378403	0.97793376	1.33778799	0.77752978	0.47905978
2310007B03Rik	12.8380337	5.5930524	2.88886663	1.95172483	1.48232359
Ttk	4.33070893	0.59139109	0.6780476	1.21007281	0.63708137
Magee2	1.74573608	0.35374516	0.78218889	0.20680412	0.27076393
Psmb10	13.9665981	5.28173552	8.13057632	9.38117225	5.71951484
Kif22	10.2051239	1.53812302	0.49735693	2.75563484	1.56974861
Mis18bp1	14.890219	3.098287	3.03729242	2.71322268	2.0429327
Mitf	4.19379927	0.63288821	0.24489854	0.23124679	0.28258174
Zfp618	2.38745725	1.03551864	0.40003998	0.56362776	0.60128851
2310040G24Rik	4.03891755	0.66070467	0.46749664	2.5492944	0.26971581
Nfkbie	2.69358486	0.25380229	0.17459516	0.71715159	1.10083822
Rfc5	30.60803	11.7504224	12.0071872	12.7007017	7.03814045
Uhrf1	25.7203048	2.95923666	5.09410421	5.08343899	5.19568608
Pold2	6.96671466	4.42077081	2.7004464	4.16485515	2.46681072
Cxcl16	37.1344343	8.36676659	8.11546532	15.9701694	12.5093376
Tipin	50.7261862	15.6869619	16.4051685	23.7798269	25.5461628
Pbk	83.4757168	5.00847051	5.1727111	8.91136041	6.79410186
A2m	3.40647661	0.46041398	0.53003303	1.47399404	0.40275482
Gm5741	1.78934514	1.56111621	0	1.09517914	0.63728583
Jun	66.9714025	22.4612635	9.47364011	12.9245113	18.397504
Hmgn2	342.959404	96.7742658	86.6288538	92.191842	57.6748754
Pdzk1	2.91241813	1.45526708	0.53937053	1.28830654	1.27301919
Gas2	3.11258415	1.31785383	1.13027575	1.54086973	0.4401656
1700026L06Rik	3.79024723	1.29728524	0.53995513	0.44612364	0
Traip	3.21585269	0.60754452	0.38062466	1.35411841	0.71691702
Lck	9.76188912	4.30047434	4.30945912	4.16847992	3.22913671
E330013P04Rik	3.43385225	0.76683235	0.55425829	0.86767816	0.07573542
Ankle1	3.57533631	0.4558986	0.36785603	0.81360075	0.09795209
Psmc3ip	5.70266832	1.58038925	2.48497869	2.09421086	1.2903071
Tacc3	12.3715891	2.7114061	2.09334604	2.75971235	2.88660132
Stil	3.21897041	0.24026188	0.09066814	0.48318339	0.71598967
Slc40a1	70.7296017	8.210917	3.82319117	10.2380888	18.2901032
Anln	17.8276561	5.76181757	1.16960397	2.6505719	2.61238989
Cenpl	8.28898295	1.9762353	1.52113775	3.27920982	2.81395493
2310069B03Rik	3.77522616	0.64320375	0.16489618	0.22888506	0.39956534
Entpd2	3.36663343	2.00906065	0.34291936	2.03996196	0.53957086
Exo1	2.68853133	0.13949676	0.51545481	0.50018352	0.55047791
E2f2	3.56475888	1.46978201	0.44606888	0.79607365	0.05514715
Mlf1	5.39218105	0.70566217	1.19625718	0.82507897	0.45010713
Asf1b	39.1107006	5.70007558	6.04227463	7.78794052	7.54938593
Tmem200b	4.95037643	0.22425377	0.70522607	0.26220347	0.72473819
Aif1l	17.8653088	6.05161173	5.33710813	14.8610085	6.17603988
2610318N02Rik	2.62701119	0.52579775	0.14309237	0.37832413	0.37149837
4732456N10Rik	2.52199904	0	0	0	0.08249004
Gipc2	20.0175123	3.56487802	3.29633209	3.97868876	0.95088692
1700007L15Rik	6.02356972	3.70153818	2.18258971	4.64852474	2.16864869
Susd4	2.65973967	0.37742957	0.14836605	0.94144146	0.48790728
Reep6	14.4914866	6.14658531	3.23490975	11.9680191	2.43660672
Ptchd4	1.73203762	0.03715864	0.14899052	0.00868938	0
Eif5a2	3.18743034	0.5436387	1.06447885	1.18732161	0.98401425
Sh2d4a	7.63591883	0.03701092	0.04364654	0.84384601	0.24551742
H2‐K1	998.182575	472.693847	298.659866	275.379223	237.634825
Ska3	8.94496626	2.12974933	0.90844663	1.61595458	1.06364696
Zfp711	3.79522997	1.69105299	1.56192101	1.45180791	1.36376223
Uox	11.0750184	1.86148072	0.58976108	0.06496993	3.57267344
Tmeff1	100.781702	49.1077806	40.1824717	35.0098543	37.4918226
Atad2	8.17010786	1.18933815	2.56075676	4.21395691	2.14252736
Pih1d2	6.31916293	1.5861635	1.72665556	2.80565351	2.24138335
Cars2	4.79394042	2.93388585	2.18691557	2.84786041	1.97730845
Sel1l3	13.499051	6.40057644	4.33615563	2.8661071	5.47822873
Mrpl13	39.1602003	14.7881003	18.0540341	19.8042572	19.4801899
Cox6b2	3.13102483	1.52596524	0.79980109	0.89209956	1.38430299
Mms22l	2.78957059	1.14679981	0.75734804	1.74312947	1.36934383
B3gnt3	26.2459987	6.47974627	6.98075673	8.88647733	18.496448
Cenpi	4.84737464	0.86208526	0.84400864	1.3312851	0.89641094
Ccnd1	20.3915373	3.46669788	5.21766968	10.1097306	2.62939162
Sox4	67.4420927	25.7670345	14.8661978	27.6337716	34.3132989
Gins1	9.95898969	1.36957977	6.2000111	5.4755807	5.50078292
Cdkn3	27.5863163	3.57703625	1.6404717	3.71765854	1.01405118
Coq7	22.0117528	9.64128162	12.4791008	7.97347413	7.43963522
Depdc1a	13.5407962	1.67959755	0.7995604	1.89176294	0.99073686
Ascl3	2.77103497	0.14505556	0	0	0.2220571
Lgi2	5.04231538	0.05314438	0	0.06213782	0.298304
Spata24	4.84545062	2.21939493	2.0564545	3.21282686	2.10324053
Nans	21.0293999	13.4734823	8.97528882	10.0621501	10.2099616
H2‐M3	8.28302864	2.37158201	2.33064843	4.78958342	1.50354733
Kif18a	5.20841582	1.04902559	0.39443872	0.9065794	0.52753945

**TABLE 6:** Gene list of cluster 6

	D3AM	D4AM	D4AM	D5AM_mesometrial	D5AM_antimesometrial
Wfdc1	14.4480989	26.2374357	30.0633529	19.3078801	2.59275568
Hemk1	7.34151655	5.39118745	5.94492075	2.12846118	0.66691352
Fam107a	4.23129283	3.53416053	0.17285777	0	0
Hey2	5.7433676	10.8395228	6.53455177	0.04729052	0.51596996
Parp9	20.2368487	18.3612496	29.3283962	12.5820636	3.85625873
Sox2ot	3.0519949	8.77547046	3.98500218	0.34268905	0
2310067B10Rik	7.23124019	12.893395	9.45177261	3.96655353	1.87231412
Sult1a1	22.7526917	44.9628815	29.2228052	22.1561934	0.80102642
Mfhas1	8.09040156	10.7794977	9.03701588	4.22302533	0.83181315
Prkag2	17.9991802	15.5389717	11.2916426	7.35394854	2.80921486
Elmo1	2.68662391	3.94015737	2.51861093	0.45865521	0.46261266
Tmem229b	43.5128744	46.0208071	24.8491595	7.39114584	8.35116336
Ms4a8a	5.06264503	12.4935466	3.34852026	0.16599758	0.09659416
Fam221a	14.8165939	24.4902247	20.8393041	4.11515521	0.94786761
Clic3	7.75358011	5.19095561	3.02302789	0.67437814	0.58863196
Psg17	5.50732119	8.86989672	2.17316394	0	0.02507555
Bmp5	9.10590443	3.03824831	2.24532766	0.89994151	0.04134291
Pgr	68.259761	22.2092291	19.801085	4.31924619	4.58930902
Aamdc	68.1327059	60.20699	74.7881189	21.3198142	5.03263118
Osgin2	27.395315	32.4598935	15.5125757	11.9196754	5.25140855
Cfb	26.2318591	12.8430933	13.7828117	10.5050019	2.73937788
Incenp	10.0746689	6.30308554	5.01174703	2.7729559	1.55943861
Jdp2	16.8142847	43.364671	27.1266932	11.8084493	1.33352313
2310010J17Rik	6.4241999	8.56582721	6.9588641	3.00461785	0.97132784
Adh5	198.229924	249.877168	190.096493	58.1514165	17.6918527
Ppp2r3a	12.8368073	21.056427	8.54790807	4.66855876	3.15915769
Nod1	12.6139939	15.0772356	4.95115821	0.97635902	0.92323446
Elovl5	354.103461	242.342717	263.231621	180.535089	34.9134553
Pllp	109.123425	227.696526	80.428065	32.7715012	9.8484107
Samd9l	25.8741576	9.85073702	8.37546954	3.39352054	0.96119161
Phf11d	10.561089	4.57524452	9.12743958	4.74767903	0.42802071
Pknox2	6.04562865	11.5492595	6.28085737	1.59066154	2.80636325
Gm4951	5.3440093	2.8038779	3.10134349	0.67828315	0
Slirp	49.9593135	33.6068453	44.3880059	18.003804	11.3494674
Gcnt1	42.9748577	52.2637959	81.7960381	36.4522725	3.45034482
Slain1	29.4404598	34.0865735	22.7435407	10.0729834	6.80872455
Ikzf2	7.96024233	11.0551088	8.10175324	0.88969016	0.36054914
2810474O19Rik	195.399105	96.0086466	71.0218761	52.5830088	34.3541083
Pigr	25.1087877	11.8931989	15.2178313	6.34629136	0.96395897
Nde1	86.3297608	127.338374	106.560177	18.2600244	17.1748809
Ccdc28b	54.7312394	52.1189796	38.1000482	24.8031999	5.91006736
Traf1	9.36804486	15.1081133	14.8040601	1.77678095	1.61829485
Tsnax	194.762307	234.393339	191.938881	85.2599935	43.4276573
Reps2	10.9056508	4.98624199	1.3770625	0.29817197	0.05479155
Pik3cg	5.41086351	4.80825587	4.84538682	0.4649078	0.46591331
Acss2	6.83652375	9.83244217	15.9008813	1.81743812	2.58243603
Col9a2	1.92357861	5.76191732	4.77186493	0.80669422	0.20933424
Bivm	16.0518273	16.1887445	12.4625404	8.11452806	2.79506318
C1qb	55.0185253	49.4206467	34.4001075	62.0120264	8.16830005
Gramd3	120.109669	180.090845	149.397777	55.7558992	18.4154809
Fam102a	38.5672169	58.0893672	39.9368544	19.1236924	10.9298222
Zfp677	17.8033287	15.8431851	20.4670252	8.36779041	3.76856522
Stmnd1	3.25340784	6.04838038	1.63921081	0.30747509	0
Gck	3.07903746	3.30720208	0.48415576	0.18667641	0.11638628
Akr1e1	57.9786358	52.0415812	59.3274517	21.2497572	6.31482172
Enkur	9.73552329	8.15686472	5.69099497	0.39946484	0.52301022
Dnajc9	69.3698843	25.4924246	55.6089779	19.1979777	6.40802424
Pxmp4	7.28522962	14.2312544	9.54313068	4.119107	2.49183967
Dnajb3	5.8350676	8.60962495	5.28442812	1.94267764	1.74705298
4930444P10Rik	4.36563612	1.92820713	1.7685978	0.25050123	0.07288346
Zfp667	6.05752823	7.5739864	3.07599881	1.3699435	0.66905464
Gstm6	8.09485382	5.11157146	3.72164386	0.36379214	0.18144944
Add3	36.0922534	51.7023477	33.6165078	18.7926648	6.34962791
Gldc	8.08039165	14.9893552	10.4656666	0.63270587	0.86980676
Gm5617	27.4033324	22.2452894	27.7599207	10.2147514	5.86141876
Ptpn22	13.4821822	11.6561817	8.92050871	2.655515	1.45585237
Tsc22d3	175.295731	263.143956	171.204045	134.442336	49.589956
Gstt3	26.2169793	24.9051795	9.34951671	7.160082	1.59032245
Zfp599	15.0713082	14.82842	7.55234666	2.52947362	1.24283218
Zfp931	4.58204676	5.929258	3.19066235	1.48075935	0.8518638
Pts	250.397781	232.941442	189.897945	69.8673547	16.0404104
Ska2	56.8003967	38.0713309	30.5729746	20.6814851	12.0087514
9530077C05Rik	5.80465665	4.55785137	1.09180079	0.77716931	0.09690763
Kcne3	88.2141831	61.6660892	55.2884614	28.9939858	7.96941134
5930412G12Rik	7.18548762	3.57687099	4.34370141	0.77171043	0.23901531
Aldh1a7	7.66079071	3.70363452	1.93466447	0.08718902	0.17757382
Zfp959	39.6596128	46.176254	31.0584886	16.5318631	10.5937538
Cndp1	5.18650765	16.7967054	17.9938282	7.97840646	0.24013669
Irgm2	14.7347984	18.6370797	13.4379784	5.31570158	1.68044692
Gm4961	2.26167709	2.25400469	0.8860402	0	0.04647171
Smarca2	10.2114262	20.4364117	12.1543876	4.43662119	0.84147698
Arvcf	19.1106479	43.259769	7.09283916	4.62090266	5.88933028
1110059G10Rik	27.9395516	24.8329416	31.728616	10.4028441	8.31827705
Sh3tc2	6.90899178	13.3876374	6.19810609	2.37369223	1.5078688
Aqp11	86.2747693	132.465788	60.2936281	60.9114004	10.0799142
Ercc5	6.74884069	9.20661469	8.73398743	4.49396699	1.60290894
2610001J05Rik	156.790169	170.142445	82.9581811	39.3819047	36.2584884
Bcl2l14	10.3986208	11.6478959	6.9361128	3.31711014	1.62421766
Cldn22	3.08345011	4.99682829	2.73372655	0.24092472	0.57830162
2610524H06Rik	90.123245	63.9380334	57.2539089	46.9455201	18.0274335
Mlec	55.5044387	99.535846	74.5377122	44.0272317	20.5623184
Cpxm2	5.15220707	4.26461682	4.83975469	4.91799781	0.25338746
Hscb	9.16532452	6.68716942	6.60930152	4.46789498	1.23493945
Dhrs3	74.3202783	102.950619	62.8716201	38.4139516	11.9229546
Gsta2	4.80507186	5.38995911	1.55376562	0	0.18335943
1110058L19Rik	285.777764	219.174162	222.166036	89.5802897	33.9242437
Hdc	476.802206	906.35177	902.727549	274.927728	35.6220801
Asph	5.11869859	9.49621883	3.94413052	2.59562385	1.26669241
Vav3	19.5609415	7.86601133	6.72263962	2.20213439	1.3979173
Gpr128	15.1379968	5.11856539	2.39214849	0.02216579	0.05804232
Cpd	15.7685172	14.6250934	13.5391153	10.5190807	2.87210349
Arl6ip1	389.498578	369.992291	391.950858	201.398057	112.102606
Tfb1m	8.50587255	10.517289	6.38252059	4.62263018	1.4102457
Gprasp1	13.9747151	11.0245875	3.73922779	3.204978	1.98190651
Caprin2	18.0859117	17.661714	14.3596368	2.93808574	4.9971358
Ankrd29	58.8827909	120.966177	173.439912	81.622239	5.92229561
Frk	34.1197118	28.9445181	21.4987132	7.56630527	6.223455
Itm2a	25.3734209	17.2250807	12.728207	19.4063238	1.05667613
Lrrcc1	15.4836524	7.22736254	4.85727427	4.18987589	1.62147867
Acot13	81.3720208	67.7005578	81.7749975	64.1462066	15.0068735
Tgtp2	11.1156289	6.49149372	9.39226638	0.97798667	0.07422932
Mccc2	18.0529536	20.3709495	15.6932722	13.4171475	2.58334791
Igf1	8.74272107	16.3825935	16.8092262	3.30099536	0.9693397
Agfg2	19.0368215	24.0992187	12.9466342	8.38915033	5.37321043
Clmn	5.33708778	6.87893419	3.04398463	1.02101971	0.20969378
Mrp63	67.7965562	54.9771185	56.326362	33.197942	16.6611391
Arl4c	23.4472691	7.86273536	12.2109823	4.30229222	2.08186464
C9	2.54646665	4.95528499	3.3490256	0.84705423	0
Marcksl1‐ps4	7.44025988	3.92748698	3.99478788	0.6314168	0
Nrtn	17.2750419	7.30779402	9.62145853	6.9058021	1.22598094
Rmrp	10.1967369	2.79297881	4.83079856	0.65312501	0.95013523
Cobll1	5.20917956	8.43429776	6.11629848	3.46520902	1.05654284
Ergic1	38.1773521	65.9495911	39.947328	22.6072544	3.21231231
Enpp3	181.689265	229.502161	128.53528	108.169722	11.7138342
Rdx	137.628719	271.452122	113.885927	32.8158674	27.0029139
Traf3ip2	12.6920554	17.7349771	11.6052058	5.72177301	4.21048766
Fam198a	1.55891495	5.81776811	10.7210511	2.65337518	0.16843654
Zfp605	4.97657289	4.19949157	1.93124863	1.19436255	0.64535784
Fndc5	97.3348423	126.230169	79.045774	52.0703231	10.4630917
Psmb9	60.4381291	32.2093524	30.5670877	14.6331876	2.78792782
Nbea	10.6459582	10.8302206	10.0834068	3.84849984	1.41313475
Rpa3	271.068026	167.177439	237.564741	73.3827241	54.2232347
Apol9a	20.4797042	5.17579989	9.75065826	1.02764425	0.59798721
Ankrd13a	64.088733	74.8859571	68.6108575	29.2353109	21.5168232
Bmp2k	7.50609581	7.75709325	5.09337394	2.20592111	1.73448865
Bspry	211.722864	147.092077	87.3382328	35.7226532	48.5097729
Ap1s3	26.1209549	26.136429	22.9278166	8.05619256	5.61458562
BC029214	15.7176893	17.750932	10.0951303	4.2983441	0.3751816
Fam229b	4.377223	3.08630535	2.4815746	1.6402683	0.14317106
Fzd6	76.5110567	89.3296497	51.5417488	26.1586722	25.585686
Zfand1	50.4150711	65.6873741	59.1704507	23.2178895	5.12392224
Gtf2a2	171.758935	135.686362	140.322538	67.2678907	44.3052189
Trim35	42.8195106	62.8660309	36.9948166	23.7919046	9.68833603
Rec8	7.39383147	11.1877716	4.23135641	0.99054645	0.12714705
Fbxo36	7.02837098	5.74629529	2.95215468	1.19739586	0.98708493
Prr15l	35.5264858	41.8810112	28.1973925	41.3198276	2.49393058
Sgpp1	45.142027	56.3497199	21.6906343	11.0409696	5.23517652
Atf7ip2	7.46264882	11.7194336	0.98391596	0	0.35643573
Cth	16.0042014	16.8312771	10.3938614	1.22997353	3.30780005
Arhgap18	19.1342928	33.7871137	25.783853	26.1102498	2.2544691
Tle2	2.2400163	2.10407393	2.63639087	0	0
Hsd11b2	7.19479971	11.7141972	11.7896044	4.01506452	0.84787622
Gas2l3	11.0651133	10.0542546	2.63088096	0.55895035	0.17811516
Ptger4	24.5368326	37.3935228	24.6141077	20.6496285	7.1356245
Mme	118.734491	262.592706	298.236299	129.143662	22.5668322
Rfesd	10.1430432	10.395981	8.97670758	5.68618817	1.49255586
Spsb4	7.68399388	12.984682	11.7956327	5.44514692	1.10040303
Zfp1	25.6810175	25.493011	26.71246	11.9164529	4.26648237
Mpc1	27.0337324	15.6380126	17.1767645	11.8155379	3.51455755
D730039F16Rik	6.00782434	29.2787614	24.3942791	11.9377052	1.71780505
B2m	3947.13132	2399.6343	1870.45649	1080.65674	555.341861
Parp2	31.3658015	39.1480447	28.132377	9.91133035	5.05622859
Hook1	45.4589626	51.0596586	14.1881203	7.23126537	5.3502543
Nt5dc3	8.95126854	13.3345343	11.2308762	3.28921745	1.44866889
Jakmip1	17.3569316	15.2410032	10.4190712	3.36799002	0.59233871
Galnt11	33.4912455	37.9450152	40.3517117	33.0803578	6.9512058
Zfp85‐rs1	15.7001994	13.4129303	16.852256	4.04890393	2.54454611
Rab17	5.9192149	5.48808401	1.42446624	0.64475162	0.88435664
Dach1	17.1110481	20.5798439	10.5977088	5.33958343	2.31032882
Lsm5	56.8812541	32.296721	35.9176141	13.1450643	12.308139
Pccb	14.6306453	14.7065187	10.9119959	8.7544219	2.62313633
Wnk4	3.8202605	6.4684707	5.692027	1.01033884	0.3527503
B230118H07Rik	6.17915965	3.74612848	1.99597781	1.76786078	0.43758983
Irf9	40.9513884	50.7855262	45.131484	24.0771004	13.3559746
Alkbh8	22.8772609	24.5755055	17.058957	17.9424677	3.66193592
Zfp759	3.98911254	2.51464347	2.04636068	0.65337511	0.06003152
Creb3l4	10.16177	8.34176865	6.84337208	0.84812327	0.23554544
Trim30d	5.84788169	5.32756868	11.1032524	1.92768625	0.43107519
Usp18	25.8490153	21.5111412	53.633866	14.1645642	4.39658849
2810001G20Rik	19.3970397	19.2656641	16.6879695	8.04022744	4.05995631
Fam53b	6.1963021	6.61848412	3.72832838	3.52378469	0.70360082
Enpp5	23.2549773	31.3893358	28.870453	11.6196994	1.64914991
Spint3	2.58963388	7.48659483	5.12291364	0	0
Nsg1	17.6741962	16.4141886	34.7636906	11.6941932	2.51519443
Alpl	5.70213502	3.6225838	0.93601522	0.14277375	0.58156131
Bbx	18.9846431	23.5639616	10.5476856	5.51990997	6.10925433
Ndufa2	313.85437	222.725541	290.167532	148.48614	75.4719894
Tpd52l1	225.538772	182.671542	165.427208	47.8202291	21.8186145
Pinx1	25.9205605	37.3327479	41.1207872	10.6356405	9.32201516
Smim8	83.7129564	87.8819169	63.3814321	30.4192798	11.8382545
Tmem56	3.73117349	4.00781836	4.51286028	0.6062033	0.46193492
Zfp946	17.0574286	7.5832678	6.74854444	3.34548379	2.6155591
Vegfb	11.3942422	11.6228876	9.79725138	3.6893242	1.58973484
Plagl1	6.90418382	4.525783	2.76616245	2.42392432	0.71517497
Hsdl2	51.9499851	57.1941418	63.9628384	28.9507981	15.4814657
Tc2n	12.6435977	9.05592394	6.2862075	1.85355396	2.79083839
Fxr1	36.5746422	53.8914772	41.5033124	19.750594	6.6305136
Gsto1	1584.06552	2368.55509	2376.44291	1277.17068	422.155476
Gm4832	9.14308224	9.81861623	6.47426612	0.74065723	1.61620941
Ube2l6	112.322081	62.4275401	66.3930048	69.322918	16.5400253
D8Ertd82e	10.6958085	16.1648031	10.4978912	8.0160392	2.47074915
Epb4.1	15.8286298	19.6473332	12.0612643	6.1611554	4.49282356
Tspyl1	105.968996	95.2679271	87.0385906	44.4804136	28.5904008
Rfk	129.241056	178.63849	155.46335	64.5011386	31.1186515
Pmp22	34.6516307	94.4769151	135.153727	25.7629591	4.75858955
Itpripl1	27.9354655	39.4395477	18.633172	10.105817	7.8747896
2310030G06Rik	63.374001	40.732815	39.0897951	22.7524494	14.8104815
Il13ra2	2.88697584	6.90062148	7.15499136	6.47030313	0.20413062
Tmem108	5.45785809	4.46584098	1.89725296	0.29188956	0.05661694
Pdzk1ip1	579.049768	706.614568	296.469403	183.745644	56.423221
Ppp6r3	27.7768113	40.3665087	31.812992	13.0104453	10.7340742
Tppp3	22.1617851	14.601315	19.047216	7.89299026	4.13364497
Ppap2b	97.9000285	127.460942	65.9040395	57.4083529	15.5351017
Lyz2	209.146289	249.289433	231.999444	197.961141	35.0030898
2310001H17Rik	5.42724074	4.39467407	9.34436003	0.46712452	0.33977528
Slc24a4	1.18453356	4.57708526	1.81847699	0	0.02497966
Efcab7	7.09835873	6.58706932	6.63936031	1.77733305	0.86185989
Lurap1l	216.131268	140.44349	173.300278	128.901532	36.2018859
Parn	29.5497491	42.1352903	29.6098654	10.5009388	4.39380248
2010107G23Rik	12.5450564	8.66721616	3.5930983	4.66922127	1.66040386
Tspan13	14.9888543	10.4912205	10.5822875	3.8916922	0.76089873
Tlr3	10.1577718	9.97584643	8.02435051	5.21629579	2.07725428
Coro2a	81.0168422	111.523038	57.840952	18.5064969	21.6079867
Snx10	81.9072351	102.314365	141.845419	43.3884086	27.3581917
Susd2	1.34229899	4.37323564	5.45412741	0.47822262	0.35319927
Iqgap2	5.20925124	3.91288056	3.81918531	0.22823349	0.08149661
Gdpd5	21.0395515	21.4079007	13.3140206	3.96012863	3.42743451
3110021A11Rik	9.73426919	12.1797377	8.06110197	2.61567056	0.82444987
Lrrc51	8.56795921	12.8478724	12.7271267	3.76917491	1.38272417
Stat1	11.1770596	9.71319741	11.4546568	5.98027243	1.87330621
Cntnap2	5.09708962	2.32389608	0.5075079	0.0167726	0.11711991
Bfar	88.2084192	122.887657	84.0724642	19.6176744	12.581461
Gata2	188.215052	248.396839	88.6689129	29.5379798	32.8885077
Aldh2	24.6132023	14.0830083	8.91957596	9.3046393	3.83584273
Snx8	16.0782267	25.641513	23.0027018	15.723843	1.48049402
Mlycd	17.0481988	21.6766189	16.1601714	13.1713544	3.48382918
Ndp	9.77498873	22.3887383	24.1164088	15.0916594	1.65337329
Lypd6b	17.0948805	14.3641821	3.99042989	1.650677	1.66949363
A330035P11Rik	3.05491869	3.58866698	1.78192496	0.33126038	0.34696934
Ces1f	2.99667294	1.73801554	0.50452204	0	0.02728848
Cyb5rl	8.85741328	4.33883313	2.70885901	2.16351899	0.32559151
Il18	11.596536	4.73021816	3.83507392	2.35054614	1.20686923
Tnfrsf19	24.9953116	17.7077702	7.95104746	5.97820623	0.5469692
Slc50a1	43.5808062	39.4806594	39.2325128	30.6964495	6.74949293
Far1	98.7040981	105.06485	149.001311	43.3196839	29.1810163
Noxa1	5.03731015	8.03329834	7.08709969	0.93210456	0.18775127
Stim1	9.3728233	16.0973432	9.04769431	4.20284616	2.49397316
Cmbl	5.13675172	3.83665677	0.75408719	0.92018894	0.1505978
Pabpc1l	3.33641202	3.85026924	1.52132707	0.32487485	0.3848416
2610034B18Rik	48.042861	54.1587237	26.1281172	14.6659062	7.72854962
Jak2	88.1070859	135.0967	96.0637428	32.6129686	12.2176435
Slc29a3	4.79131688	3.7230834	2.14343639	2.15942573	0.27923822
Ndufa6	462.537198	437.433681	542.602196	250.063295	116.459433
Tmod1	3.11180824	5.76074174	1.51435692	0.1876796	0
St6galnac2	49.2054755	38.9638633	28.0984717	16.6386783	8.58533408
Ippk	36.8570853	62.8573356	54.5523417	12.9264325	11.220347
Slc52a3	23.3404095	19.6043686	8.95077463	2.07791038	8.14278946
Cap2	4.88463722	7.2789497	9.0626996	3.61706512	0.75714713
Zdhhc17	6.20858874	9.61493805	6.69173943	3.31732361	1.06858877
Myzap	14.282017	23.0117965	11.0824825	3.14690107	5.31043607
Adamts16	4.78533709	3.1192194	0.874089	0	0.01050612
Gm166	3.6257288	4.38146715	3.57166692	1.03875035	0.53576237
Ppp3r1	155.503025	133.19715	81.2176941	52.1665038	38.1078452
Mir221	1.37287767	4.31196729	0.63563139	0	0
Bphl	34.6824784	38.4140469	35.5117636	31.3462191	5.96486882
Birc2	27.1646146	26.7764243	27.5963214	12.641297	3.44499669
Ccdc53	35.4195471	31.3020214	38.3153439	18.0173778	7.65482409
Tcaim	10.4930952	14.1027509	17.2471749	1.7927447	0.87697576
Prcp	15.7528913	10.3134795	9.63633485	5.77690983	3.0501136
Ccl6	10.8534776	14.5483855	13.5824159	3.83566918	0.65503752
Arhgef4	7.61684067	7.6599382	4.22739373	3.83837286	1.29006939
Sox5	12.1015966	19.2977292	12.0786431	3.15104173	0.59853078
Enox1	2.93498462	7.21617732	3.94073582	0	0.22049552
Tnpo1	13.1155962	18.0176401	12.3855408	5.70601758	4.50707381
1110032A03Rik	17.8216009	17.4992191	15.5537075	11.0869987	2.52196388
BC064078	32.2953129	37.0014276	33.2117401	20.7406068	1.67970336
Krt35	1.9692264	1.93281054	1.50787121	0	0
Fzd10	7.87865459	2.48482887	2.34426302	2.04374795	0.20986923
Tprn	23.9666893	24.6723075	15.3758056	10.4900064	4.59671906
Stk30	4.46813703	7.86367336	6.06346541	1.16698356	0.18520061
Plek2	29.6416771	18.8839702	12.7310697	6.17612305	4.95283259
Bag5	51.0763842	79.1322203	72.2867037	30.2712435	16.0491007
Tmem119	5.0211118	6.11104122	5.86995494	5.18602632	0.35207128
Ddx58	27.1506488	49.7543494	56.7201035	16.1332316	2.73815019
Car2	727.784314	1005.67001	1100.25485	143.587162	220.970137
Egln1	126.8149	134.37578	115.029621	30.4785495	54.8228567
Lrrc45	14.9857151	22.2863758	21.5503042	6.34430204	4.05244442
Glrb	7.07114706	3.17029891	1.63694228	1.60293957	0.13991282
2010315B03Rik	29.0722646	35.7634239	24.8850035	13.1283888	3.5987362
9030617O03Rik	3.02934451	3.4411275	0.6732299	0.68602736	0.21038919
Nudt22	28.0038186	15.5817837	10.1548318	6.17276142	4.1847798
H2afx	207.055512	140.162734	94.4209616	69.6057929	36.1841419
Hnrpll	107.423492	91.5142752	46.5289929	15.1403712	12.930194
Cacna1d	1.96650037	2.98306157	3.1262869	0.53062289	0.17509518
Klra7	4.26667759	5.29417631	4.34108514	3.38520638	0.3799006
Dpt	16.2630619	14.459982	11.2206813	6.36756965	0.95826597
Ifi27l1	77.0402106	46.293248	47.7689364	22.0034281	9.67730328
A130010J15Rik	14.7296096	20.7409394	14.2063781	5.04380362	2.97134101
Zfp709	5.6926934	8.1025662	10.451539	2.74120812	0.86767066
Flywch2	40.1885994	23.964949	17.9048493	11.1385661	3.64586775
Msx1as	17.3162667	13.4096172	6.98235853	4.70640053	1.93457608
Ctu1	15.6200428	20.6359374	14.2674561	6.6224415	2.52892846
Kcnc4	0.7791639	5.64219417	1.96406513	0.19870492	0.22547185
Ntan1	81.7274438	74.912926	80.5133094	27.6161794	28.698384
Secisbp2l	32.8888733	45.6826194	30.0636721	9.40722947	5.5970891
Nmi	52.4839694	36.4945148	55.3970413	18.8187214	4.9277847
Slc39a14	23.6154467	31.1475056	30.9461438	4.701756	6.00037399
Rtp4	129.88444	53.1902958	115.77947	56.2159466	16.4114267
Zbed3	13.2902387	15.5722578	9.08644133	8.37671335	3.06260591
Fra10ac1	23.5097937	18.8629291	13.3468952	9.16461778	4.18657584
Mbnl2	36.3655787	44.614253	79.1251489	20.1582237	13.8649209
Emx2os	10.7755728	6.6770895	4.47207114	3.95715134	1.18601392
Ms4a6c	5.05295255	2.02123467	1.91277965	1.57552086	0.05093318
Il20rb	10.7896662	9.17467616	5.26358197	6.23341668	1.4340217
Tubb2b	16.6931068	10.2036245	6.56631699	0.68529419	5.73689873
Kbtbd3	19.7301649	25.4967728	32.5820039	14.5602546	2.97259951
Fam198b	9.1121208	14.1861577	4.492198	0.7227378	0.4337044
Mob3b	19.0119652	29.0923713	23.3528654	10.4250883	2.07989802
Prr15	13.8828651	40.0070788	47.3984942	12.3554605	6.43284896
Sfrp2	21.3135657	11.9503016	22.6632291	16.6654046	2.4809878
Sort1	32.3988235	33.1980259	21.0432513	8.13814499	3.00122081
Chd3	18.9070013	19.0021783	11.4450316	3.62622972	2.79194386
Nmral1	18.8164651	7.23303729	8.00972199	6.18809228	1.26029996
Atp10b	14.3561991	12.8289452	3.88932074	0.71056926	3.66838288
4‐Sep	12.9945054	11.1110251	1.32876489	1.13445206	0.35136419
Avl9	25.6616916	31.0855077	18.1308368	9.07307874	5.38897504
Klrd1	4.74403746	10.169969	12.2025079	3.52581921	0.72412154
Osgin1	5.18767138	3.47282679	6.52810617	1.67916298	0.37307707
Zfp467	4.73270474	3.62487446	2.06050104	1.2653916	0.3326804
Zfp41	8.55063631	7.91979274	12.4098652	1.79433149	0.37756244
Slc13a5	6.08429652	11.7321059	12.5424909	1.44683806	2.493778
Slc27a2	7.68029894	9.99168647	5.69471582	6.29660576	1.04469454
Pdlim2	19.8281455	31.6035731	27.7236925	2.7922314	4.0198029
Car5b	19.1721233	22.2990219	8.29877689	2.13510714	2.72726552
Samd14	2.08331498	3.78093123	4.11302168	1.37133944	0.14174712
Tmem14a	28.5007924	22.6552778	13.9558816	5.59933314	2.20872264
Prss33	1.8860937	6.33115587	4.2789069	0.0865796	0.07557113
Tmem123	343.236161	372.384199	315.20119	144.375901	47.9785186
Plscr1	85.5275218	114.654324	123.0827	14.8765669	28.8746141
Ldhd	9.68926499	14.040346	19.7114417	5.47211523	0.89409394
Klk10	14.5450002	4.19771926	0.71355961	0.22108491	0
Cat	21.754272	24.1069143	11.7645379	9.4345969	2.02802287
Mir100	2.44543836	7.68069173	0	2.24511723	0
Enpp4	11.8059346	16.8771838	23.3748318	9.32796859	1.8233339
Sptssa	552.9358	510.505845	406.568584	288.938736	130.356044
Dcaf11	40.7965102	53.6672825	39.5864551	26.7786088	10.1692239
Rbm43	19.5483765	14.2089827	10.6759527	9.30690514	1.52771164
Hibch	28.0900118	26.4136464	20.3055133	16.3728424	5.88816199
Epsti1	34.3751542	13.3855294	11.5724879	3.71285537	1.64289102
Rtn1	4.72828692	11.1932453	6.36402371	0.98438204	0.12677006
Dtx4	14.7037102	15.3525092	8.43505988	2.86378905	0.19924846
1110008L16Rik	17.134544	23.6227884	27.2575136	9.31563988	3.47041694
Il16	3.54226656	8.04658558	1.28298418	0.80402408	0.24091423
Pnkd	23.2898891	29.7718241	13.3192018	9.7502234	3.97156527
Casq1	4.17577521	2.59575139	1.09556531	0.79868987	0.02788551
Gstz1	24.995752	22.5902698	20.3097497	17.2395466	4.58616033
Dmrta1	3.2501775	7.56817484	2.53519075	1.32292692	0.05132083
Gstt2	14.3751316	8.74589715	5.28919551	1.52952025	0.72473819
Mfap4	9.74080756	3.21509458	2.35473492	0.91011582	0.06907791
Zfp810	17.9845703	17.8061744	11.9166163	5.15863872	2.56606936
Nudt14	83.4676732	89.4950714	85.209073	39.4853741	15.6179227
E130311K13Rik	10.3255368	8.57286812	5.250868	2.44762943	1.42427799
Pycard	109.500659	67.7771579	114.26479	114.720057	9.35004272
Bambi	126.502914	175.629786	105.218435	43.0135952	31.3544626
Cdc42se2	55.5276082	89.8609929	88.4222411	28.6566057	21.6656872
Glce	17.6952801	36.8008927	30.0031759	7.43032672	5.98841989
Pls3	338.962594	551.446686	592.903746	87.5415699	51.2847191
Mrgpre	2.49680492	5.78621641	3.37166877	0.30245309	0.40294222
Dhrs4	59.2332958	76.0657166	51.3891494	21.918555	5.92545132
Iigp1	14.5123677	6.36163601	4.15714734	1.66784245	0
Apopt1	41.3742443	38.8363479	40.7428462	24.1503754	8.07844448
Ggct	16.9492938	18.5720104	12.2366043	4.65946992	2.14861712
Atrnl1	8.093263	10.1429156	8.80595211	2.65562502	2.54023698
Cyb5b	175.14821	124.970733	95.1337689	34.0898098	22.9888914
Slc41a3	2.96085825	5.52636299	4.33729123	0.82440727	0.66124037
Itga2b	3.87058035	4.90145976	5.53426516	0.78386403	1.01869314
Lum	34.0357878	24.3067674	35.5034593	28.7663535	2.89619534
Fbxo10	2.71598103	5.39228538	5.98116244	1.39532534	0.68789463
Rsph3a	15.5376832	10.3454215	6.10011553	4.97896746	1.62637944
Xcl1	9.09326348	7.24208649	9.02166066	3.22008846	0.10409848
Bbc3	9.14151721	20.8601783	2.50585452	2.54507216	1.85122502
Wfdc18	16.3029224	3.1061621	1.77602888	0	0.48030733
6430573F11Rik	6.00304868	3.19853464	1.62129541	0.98416098	0
Zscan21	25.8029839	26.6308217	27.7536159	15.7497641	5.41761773
Isoc2b	14.3282022	20.1933679	14.344977	4.66590873	5.74095794
Irf6	274.761959	287.858321	192.780959	54.0628294	36.0632075
Apol9b	18.9297883	7.41584029	7.11747751	1.16361353	0.62250239
Dcn	63.6158181	31.6466121	37.7840771	49.0966775	6.06828211
Ube2ql1	9.75014448	26.3087079	18.1245006	1.98673063	1.67566728
Gm12250	3.93393982	5.00296723	4.00033745	0.50962444	0.03868056
Prosapip1	14.3484331	11.826755	7.68457788	3.3609113	1.13116718
Ubn1	24.4278645	36.2517544	12.3202202	9.53464709	5.50504379
Camk1d	8.07924321	10.6931301	5.22243903	1.8690893	1.60939092
Fbxo16	8.80808825	7.12448216	3.29193962	2.87413977	0.34016233
Cfi	22.6180438	5.77997062	7.36970596	4.15882786	0.88230117
Gm11240	2.14794761	3.64301584	3.58013728	0	0
Cox7a2l	570.389452	799.175195	832.671589	391.159734	157.18164
Meis1	38.1491227	22.1127732	14.2144247	16.2680198	5.46721231
Zfp872	6.63132383	4.27281725	1.18321924	0.84950382	0.38839988
Mphosph8	70.6718923	73.8266625	67.6402232	23.4343981	10.6364696
Bcl2a1b	10.0719625	8.84687006	5.84880716	5.87727023	1.0943966
Osr2	12.6228475	15.2715508	14.0898291	15.6851855	2.20031317
Hdhd3	14.8126275	11.0636003	10.2593137	3.31688603	1.49582693
Zfp956	6.23763987	14.8048116	11.4425165	10.845977	1.15496511
Ap3b2	22.8737797	24.2703939	15.7546735	9.40676198	2.29295641
Adh6a	7.68340563	3.97112573	0.09005963	0.08929126	0
Pde7a	58.4180583	71.4584422	80.4856454	44.3839558	10.227404
Tacc1	17.4152075	41.9219042	9.66787497	7.77256959	5.22913932
Trim47	20.4696374	25.5439862	10.7014843	5.67331066	2.04744403
Ppp2r2b	18.0875788	4.39772095	0.97772234	0.06322048	0
Serpinf1	39.6769918	28.4812799	44.1772325	29.1735001	3.56777687
Ctss	57.2403048	46.7176095	36.862408	36.10704	6.11850707
Ptplad2	12.3305971	1.93641183	2.91952283	0.74514821	0.10006211
Cenpv	52.9135469	40.3100133	36.4996265	19.5705899	11.674534
Clstn1	22.3790643	44.0461482	18.7395395	12.6449909	7.62054831
Nqo1	4.66398037	7.09342234	9.37679167	1.73591538	1.61620941
Msx1	281.649659	279.490733	170.491415	113.383652	45.2482837
Rnf223	10.7468864	20.1541351	11.9320725	2.20320837	1.88126776
Lhpp	15.663791	6.81878461	7.51524356	3.83732959	1.20318929
Slc16a4	4.56213466	3.48856293	2.12603732	0.27375305	0.14336746
3110001I22Rik	27.5662077	20.5962683	17.8043864	2.86156055	3.82767079
Cdkn1c	60.7120158	74.0647548	161.285737	13.2564423	10.8174511
Gm15133	4.38924833	3.87727227	4.86273293	1.15134217	0.37685652
Zhx1	6.58122577	12.8699235	6.55754026	4.13528883	2.08548485
Acp6	9.91544482	7.66902781	7.70286787	4.05723539	1.48796804
Ccdc104	25.3910255	18.5923507	18.3573517	12.3041662	5.1125136
Col4a5	12.7087948	6.20040435	8.26095579	4.45495454	1.25591747
Ilvbl	54.1237423	41.1131917	15.893514	14.8581968	6.79862758
Tmod2	7.74301402	17.6305993	13.7099457	7.7332302	1.92055508
Ttc30b	9.8079579	17.0486077	13.2879314	6.07175472	2.75986468
Nt5c3	66.8621541	69.8087617	38.5132563	22.6616257	19.839016
AW112010	50.234037	51.1385411	48.6196501	38.6256728	6.47317938
Aldh7a1	13.8210458	20.9684405	15.6483745	9.88160381	2.09095093
Jam2	268.806998	373.505592	278.758739	42.9914082	164.431385
Ppp1r36	10.8611348	12.9333052	5.07017743	1.11251508	0.25175641
Lgalsl	31.3231389	25.8347339	4.55171529	4.00229977	4.35598449
Unc5cl	9.63459024	3.64715498	3.27901525	0.08444259	0.11055875
Nudt7	46.1941131	48.0013792	46.3858878	16.7437139	11.3456811
Ccdc3	3.87639088	4.64270556	5.70226881	0.56310941	0.23592523
Scara5	3.11142264	5.42607838	9.6685032	0.06419206	0.22412053
Tyrobp	41.3621847	32.2064341	34.6733554	27.1846939	4.70870905
Il18r1	4.20104589	1.70289338	1.70507688	0.46333034	0.07651162
Mycl1	14.885277	6.89856179	7.32357906	3.95592738	0.65770231
Zfp273	12.7557549	11.7587497	8.63757606	4.02399297	1.48599349
Lgals3bp	93.8170017	70.5008143	62.5575045	54.8438231	10.8950009
Lyplal1	11.2476725	6.461457	4.55663466	4.97333092	0.9278429
Zfp932	22.9419438	13.0540913	14.6961041	11.0795183	3.17322272
Tspyl4	4.23039935	5.51434278	2.84893249	1.25880077	0.24118817
Apbb3	11.8911713	17.4820788	11.6260126	5.74889378	2.15416208
Sybu	13.2342435	32.5253057	12.3385194	6.88120328	1.37825002
Zfp40	10.1358694	4.54426805	4.29312204	2.65663949	0.51245342
Pdk2	8.50086099	12.3995259	6.27064551	6.03195438	0.83131585
Gzf1	21.5742663	19.6521689	16.1367364	6.81950965	5.24470052
Ctsd	6398.45484	15401.8964	15034.7171	4629.46083	2842.2002
Ptk2b	28.3704797	44.7564354	22.9325217	7.99945917	3.0572026
Fhit	3.83209523	2.07516156	3.60963925	0.60658351	0.21178293
Ivns1abp	214.770045	293.016026	255.398383	134.228622	58.1813998
Kcnk1	244.07723	210.391888	287.41785	234.10491	38.5472253
Oasl1	10.7151034	4.67688071	4.17918661	0.87380583	0.34444639
Gsta4	64.8032656	168.312444	150.584261	66.9316786	10.0914676
Ckb	88.5520033	321.050836	236.596367	32.7299003	27.1894246
Anxa1	1513.79939	890.005889	648.045567	188.192861	158.6828
Tnfrsf11a	6.72633516	6.37253728	1.51503539	0.39340963	0.26014256
Casp4	24.0900725	25.7810928	46.0899339	21.9912786	6.2037461
Dlx6as1	14.6330916	5.61840098	5.51668779	3.77798551	1.35350086
Rab19	30.8533732	18.5282395	16.31903	12.5540746	0.63294398
Dgat2	322.697916	329.793184	263.402994	74.6843084	45.4089508
Ifi44	13.8137554	5.95156764	12.9574377	2.12056006	0.44925583
Sh3bgrl	212.466859	141.227952	104.971072	57.2942939	29.0400994
Slc25a33	80.2899385	102.900264	42.0475075	48.8335923	18.4306999
Ppl	21.9103797	26.6750167	14.3338575	13.7442045	5.12833864
D130040H23Rik	4.42996492	2.30190561	2.11136301	0.74762479	0.13051308
1700102H20Rik	3.01155227	5.45697459	4.93376407	1.48876927	0.27845882
Tmsb4x	11725.4511	9745.07825	9595.53415	1912.43777	2015.34318
Enpp2	4.50993144	4.6563346	2.52344539	1.83238919	0.15378881
Pde6h	1.2855351	5.2305786	10.9298982	1.18022889	0.09365132
Sectm1a	7.99499352	4.56561242	1.6451636	0	0.06471509
Zmpste24	289.783257	407.979833	256.169643	80.4663979	38.1494531
AI987944	10.9687352	8.82235716	7.49919258	5.20873325	1.60462961
1700001P01Rik	2.50814191	5.82616574	1.64510368	0	0.66996715
Rhobtb2	17.4778797	14.8004918	15.364636	5.84849247	2.29768891
Laptm4b	105.50062	152.210229	134.393987	57.1871716	31.5693324
Sorbs2	11.1877033	4.42591935	1.98080965	0.90339855	0.11999412
Ssfa2	45.8901817	75.1832093	41.1893634	22.805301	15.0010552
Haus8	49.9435634	103.022452	79.0066981	13.5872165	15.3017288
Xaf1	48.8460153	53.6682668	57.479623	26.846924	7.62339064
Pou6f1	3.84855873	1.76837784	1.37268593	0.44493398	0.05711195
0610009L18Rik	12.0099073	16.047972	14.7304237	5.9966513	2.19498123
Clec7a	3.7517485	6.34154609	4.7204773	1.44613026	0.39015215
Gstm4	11.9736774	10.6880439	6.97027604	4.1894532	2.03153915
C2	5.87003862	4.14439745	3.56280529	0.74724023	0.03952908
B930041F14Rik	20.3063478	29.259778	25.456414	20.2640522	5.07448508
Abcb5	1.71609709	3.02524666	5.82662107	0.13901655	0.02696462
Serpinb12	4.54790457	5.37131948	6.99560887	1.75986134	0.06023912
Tex40	2.25443741	8.91655207	13.8398358	0.68992079	0.2007328
Id2	1369.39421	764.124164	480.278981	415.276086	237.828858
Lurap1	16.81703	22.2113907	26.9976304	8.68468613	3.40570547
Nt5c2	17.4520431	17.5876211	13.8632785	5.7337819	3.27412901
Olah	10.3785182	14.0235441	2.20237004	0.59552181	0.20792084
Pik3r3	17.6843051	23.7092496	15.0479761	3.68503121	3.58199463
Tesk2	5.33393767	8.22448741	12.6465909	4.06767062	1.34435361
Tmc5	2.37711522	3.84004865	0.42060587	0	0
Gtf2f2	100.418	71.2047768	83.1065295	35.423981	26.2533958
Rragb	5.46051412	5.65697593	2.99145242	1.31235846	0.06872964
BC017612	5.03751676	6.71891199	1.91698356	1.83727406	0.38709213
Porcn	22.9410321	35.8108201	12.3254156	29.461243	3.96891932
Snx24	26.9232108	26.6089852	24.2887521	11.5476805	3.60970611
Gk5	4.67833315	5.70597638	1.91314401	0.89390441	0.95151926
E230025N22Rik	2.78184787	3.73366959	2.09670077	1.09880076	0.0518427
Gm10638	5.84128203	16.6546571	11.2612228	1.40663243	0.6138906
Rasgrp3	11.294014	5.18172808	2.16754042	0.58279255	0.8478189
Bst2	209.256309	114.775872	143.788782	86.6783292	29.4715576
Irs1	15.9702097	9.08081346	4.94915656	3.52828638	0.50757524
Cbr4	53.3975539	67.2318846	44.7877849	12.671992	7.17075154
Amigo2	83.0667881	69.04816	38.9289617	18.087607	3.05675118
Cnn3	625.61549	1110.3706	1705.7024	933.586175	190.740952
Slc25a19	19.29257	15.7355146	11.7655552	6.47588733	1.89040158
Cspg5	9.53495641	11.4155463	3.50984783	1.74768447	0.02699945
Arhgap42	14.4566255	9.67429837	6.38042649	3.64492262	1.26678837
Ccnb3	2.73505329	2.30499459	2.55913516	0.24978614	0.01147506
Atp6ap2	170.244369	160.533377	181.562953	53.523797	36.3242156
Adrb1	1.51837736	5.34451987	1.79385334	0.31244773	0.041957
Magix	7.10870206	21.886978	16.0514412	6.71167553	0.82663072
Psg27	2.51847411	2.24119052	0.62188447	0	0
Cd48	8.96517974	8.5639937	7.2969593	4.77071028	1.32702775
Cttnbp2	56.965396	79.5998176	79.6580846	8.40862205	18.8112678
Mc4r	5.78774248	4.67336375	0	0	0
Lgals9	48.4770378	24.343176	27.5654016	14.0048506	3.52556484
Dennd2d	6.06620368	5.05225	5.39906365	3.02795972	0.60174516
Ptprr	27.178278	33.3446056	50.9924284	30.7443491	0.73894169
Dnajc12	18.7274596	5.92698068	6.81266464	3.37727036	2.10561104
Pla2g16	10.1991601	19.9993033	12.4609373	7.74925504	1.88381907
Rgs16	6.75855626	17.7626276	2.24989012	0.23076151	2.03658537
Mpeg1	6.13615529	4.20406866	3.27271005	2.33348593	0.67491156
Pdp2	8.98571594	18.69305	12.2220032	2.22500659	1.98691801
Ctnnal1	24.8678154	18.2625615	24.9270254	10.5060363	4.81896632
Wfdc10	86.6647726	62.694971	36.3099238	43.8262535	3.87092131
Ccng1	161.635972	235.391432	105.323046	67.3900019	53.5912238
Ebag9	45.4771219	64.6364034	39.9862165	18.4244212	7.92387251
Slco4c1	4.73969673	7.16522342	3.4556151	0.52517362	0.08731402
Man2a2	3.99986256	3.81261068	4.11841425	1.80869987	0.36677481
Hddc2	538.132675	586.927511	676.177236	116.654373	59.3172865
Aspa	36.7425655	10.8938894	5.46097105	2.64876116	0.95198976
Cd52	64.1745255	59.7556799	52.4617041	43.6823339	5.29846784
Zfp119a	37.3577808	37.0800919	28.5328937	19.7754485	8.40651671
Gde1	237.431966	220.022432	126.180522	62.806506	79.360216
Bmpr1b	3.28747086	2.88841606	1.47114836	0	0
Itga9	6.98006943	7.29328129	4.14715643	0.19419056	0.39058584
Fuk	9.785037	20.1879209	12.8766565	2.4568923	1.01304701
Pex13	47.8092493	78.9683929	63.6225078	22.120741	10.8901356
Ppt1	113.473048	142.93337	97.34747	55.8592611	23.0578345
Sh3rf1	16.6018565	21.7231685	18.0770328	8.52806545	4.96248974
Igtp	59.3448939	58.1398876	53.1614696	16.6913313	8.5761094
Barx2	6.22624171	3.88823404	3.23671596	0.93598783	0.35013359
N6amt2	57.4112482	38.8934549	29.1090284	15.7552086	13.2518861
Gm20337	5.76740862	4.70795465	3.8071092	0.99608237	0.8236724
Nrg4	11.7029277	10.5702466	6.89040252	2.45317097	0.05420896
Emx2	51.732599	28.0659611	18.2688976	16.1772881	6.05446064
Sv2b	4.65226251	6.32765138	6.86938709	1.07041523	0
P2ry14	14.5076061	3.55983117	5.57503171	5.56076496	0.26157783
Alox5ap	55.3612118	27.8807311	8.62642601	3.43515205	6.97581721
Padi4	17.9826174	5.35320939	7.63961515	3.03884554	1.12828559
Plb1	8.83623908	5.72575528	2.734971	0.2137805	0.12767275
Thap3	38.8109068	33.4622596	31.5476298	30.6335565	7.55246631
Sesn1	8.3980313	17.6662811	3.79779761	3.08403844	0.86078439
Nf2	16.3090768	28.975354	21.6328229	8.39623557	5.89844238
Cdo1	560.223822	485.138594	262.141863	198.079014	45.2897793
Ammecr1	8.97735707	21.8224864	37.5051143	3.11962253	3.41211317
Ccdc129	2.95875205	2.57270429	0.01838763	0	0
Fcgbp	7.41752131	4.82343566	1.95699122	0.6417317	0.63921456
Apoc2	9.0944987	2.8401924	3.06231288	0.37952325	0.57971801
LOC100504703	13.4784039	11.5171467	7.15809818	4.00345119	3.33558113
Pdcd4	25.1049697	28.4870726	23.0578022	19.9800208	4.96813668
Chst9	33.6299874	19.1877032	0.88217651	8.17797754	0.03817198
Wfdc6a	2.9333978	4.54758372	3.90029872	0	0
Tyms	50.5698842	34.5577342	19.9486101	9.57476465	4.19925839
Serpini1	2.9556527	3.43216356	0.50112018	0.43951651	0.2501952
Cyb5r1	27.592646	27.523266	31.009508	24.1172905	5.46356975
Chst10	6.58598339	16.4826643	6.292119	1.76595734	0.21780913
Fam171a2	16.8132107	29.2621384	19.6022239	5.15819125	3.94586654
Hopx	174.516846	298.648354	249.258009	59.9490905	51.1153877
Wfdc17	10.7063968	11.463719	11.4589838	8.4251734	1.00280797
Oxct1	49.6298039	29.8669058	29.4814674	14.4602228	10.5205238
Fsip1	2.26077585	5.87644077	6.71345287	0.6799319	0
Tlr1	2.56234537	3.62154423	2.53087024	1.01939398	0.11977802
Irgm1	72.4737008	55.2513164	84.4088231	33.927112	8.21288873
Alcam	76.3761833	62.9861122	53.2371731	11.5900385	29.43235
Hmbs	37.2638226	58.5486992	58.8384116	23.3413819	10.4395906
Tmod3	195.507915	161.743815	119.340236	50.6308331	54.1934375
Grrp1	3.08505546	7.6431027	5.41790214	1.90450401	0.80986241
Slc7a4	9.06222488	6.57688449	3.40144436	1.46385442	0.14556389
Sorl1	4.41762671	12.5981943	22.4637703	6.40290952	1.70224574
Ebpl	16.0124996	28.7948899	18.1707336	4.29494421	1.91216431
D330045A20Rik	9.52530297	7.95876921	7.17253398	1.20123982	1.14636434
Gadd45g	68.170368	100.370521	181.434506	18.4598528	23.5642335
Pdcd2	42.7369465	72.8294162	51.2733196	24.7497447	12.9243841
Lactb2	20.3203357	62.9271299	57.6009368	13.1986401	8.19724513
Rnaset2b	25.0739912	14.5071308	16.4225692	11.6134396	3.68845686
Nptx1	6.72603535	8.48887958	0.89137559	0.18128628	0.39558999
Tceal1	25.0135091	15.8050335	18.6386858	9.88824195	4.00891896
Cacnb4	2.69352631	4.44020665	1.8378038	0.41214712	0.1262257
Grn	107.055624	185.855401	227.045462	93.050951	37.2275446
Anxa4	160.961854	275.272719	188.932942	76.4288867	76.0250492
Mogat1	7.51610684	5.30727731	2.05288911	0.24821639	0.30331846
Pdlim1	395.34316	437.53575	164.343446	53.5844591	48.0212866
Rmst	3.25209021	2.47733513	3.41589776	0.42334427	0.42786142
2010003K11Rik	4.02334331	2.93976776	2.48370106	0.30781385	0.26867577
Trim12a	23.7755406	21.6570324	32.033738	7.61622282	7.02477031
Bbs2	5.26650224	9.78221795	9.67143248	2.70196214	1.82643912
Csf3	9.47315813	13.9375722	12.7592625	2.98690089	0.84348029
1700096K18Rik	10.4860757	9.32920459	8.04299623	3.80125668	1.33438592
Naip5	12.6923734	12.007733	10.3608397	1.88931508	1.4022215
Fgfr2	79.1054633	89.2297346	94.0564432	74.8876364	19.2373059
Itgb3bp	6.93082602	3.57197984	4.12417518	0.94024875	0.19085989
Mpped2	2.54844806	6.09667763	3.77130458	0.45919125	0.47714972
Slc22a15	5.2361902	10.2980049	3.24758038	0.85682891	0.17319428
D3Ertd751e	5.18190258	5.20069222	4.93352399	1.97667475	0.92115797
Rab33b	15.4201605	37.0171587	18.4702912	11.0186629	5.16252913
Col3a1	45.2989181	29.0165601	36.9320801	28.406947	6.84681382
Myb	15.349005	7.53766588	4.12478829	1.53756153	0.67794928
Ptplad1	111.167677	215.591462	199.979683	50.6472598	24.6468486
Ncr1	3.34304629	7.55994265	4.62277375	3.5193453	0
Ckmt1	13.3788614	13.0831166	15.0487432	1.20568494	2.69673373
Sec23b	249.817817	194.928842	93.5501128	125.695757	32.3225249
Dclre1c	6.33550917	7.59379955	3.48692433	1.74303241	0.89099057
Slc5a11	36.4639477	45.2989109	33.9197998	55.9057527	1.47329812
Zfp964	2.02381106	2.9722217	3.95625744	0.41289512	0.3003301
Cd14	351.498412	552.715643	403.938016	150.414356	32.7438053
Ildr1	36.4804796	42.9136633	54.615048	16.1427933	3.14125668
Pcgf6	49.3905504	41.100365	32.2935891	19.4904998	11.9310356
C030046E11Rik	9.33226821	11.5336717	4.40247083	2.34447135	2.00478231
Cyb5r3	240.682829	435.6804	299.338257	270.54897	74.9254843

**TABLE 7:** Gene list of cluster 7

	D3AM	D4AM	D4AM	D5AM_mesometrial	D5AM_antimesometrial
Cdca4	35.6655913	15.9604287	14.1539003	27.2742917	33.0092455
Ascc2	4.18055745	2.31944114	2.27168071	3.49336219	4.89474656
Ghdc	1.29699475	1.70524146	0.97755521	3.04610417	4.85834643
Plvap	1.29388273	1.06676274	1.55754914	1.75214175	1.63304493
Insig2	23.0990272	14.3227257	11.4921639	27.4107551	25.3412295
Napg	9.21164262	5.04254429	8.00972199	12.3933737	10.6225283
Zfp710	4.04603029	3.86993004	4.32488733	4.99849821	6.61514098
Mir425	1.5343927	0.60240719	12.0769964	3.52175252	3.68876031
Fzd5	1.92409382	3.21417434	8.62892136	3.49832171	6.4547081
Scarna9	0	7.3149445	4.313213	0	4.97689883
Slc25a35	1.39203935	0.3643459	1.81782924	2.19555343	1.68756914
Muc4	3.04776165	0.2951667	0.573004	0.97694589	3.11894782
1700019D03Rik	0.39095737	0.49117133	0	0.43071793	1.50381116
Sash3	0.55520788	1.22859362	0.88801444	1.57552086	2.58860373
Cpne2	0.53823723	0.13346119	0.36724142	0.8842628	1.72526727
Got2	65.8234836	51.9906951	27.5273116	67.6056338	48.3130597
Chmp1b	69.4663075	72.1399885	106.754154	63.2314023	134.14602
C330022C24Rik	1.15043835	0	0.7849487	1.05619876	2.1349371
Tmed9	73.8856083	139.745184	134.336817	129.147106	172.114607
Hsbp1l1	1.19702223	0.36779051	0.57830789	2.05458986	2.62746893
2610203C20Rik	1.79597749	1.35062591	1.24142903	1.69530445	4.05410688
Zswim6	2.41391997	1.88604122	3.62951697	1.62373636	3.74430239
Odf2	5.20757323	4.34171	5.10658103	8.29958096	7.90074316
Tma16	4.9148516	2.16490086	3.66305957	5.33765616	5.23534992
Pogk	2.90222987	1.42428024	1.12248881	2.74572455	2.19098348
Adsl	8.41497826	4.23486415	6.55580489	10.7739821	7.54579381
Fut8	7.68063345	5.90228948	6.74819613	8.07385202	11.9948011
Mex3d	14.4114231	9.85429448	10.527523	16.8510129	25.2946529
Rac1	164.480608	140.344416	158.32096	201.562515	262.530865
Srfbp1	11.9458187	7.97993946	11.1029119	19.7247028	12.7160956
Dhx34	0.84066635	2.0207029	1.5568872	1.35459147	1.37483393
Pvrl4	5.56458196	4.80773893	5.91097284	10.8326052	15.3163209
Aacs	9.26897951	8.19790188	5.7853875	9.92483801	13.9495902
Kat2b	10.0837107	13.6821158	28.2942376	29.2609161	25.1519443
Zfp655	10.4068637	10.4566361	15.2417645	22.5393418	22.8917801
Peo1	4.59921893	2.12863207	2.92576318	6.88296643	4.34479843
Zdhhc12	24.7411184	29.2904101	27.7649541	40.364475	47.8370012
Rsl24d1	44.5170469	37.3100619	50.2282439	75.4469531	72.7209284
Rep15	0	0.4692887	0.79939386	1.09740964	1.5964594
Ralb	73.1447761	48.0563262	38.983717	59.7383264	88.449787
Gltscr1	1.216636	0.99352231	1.29557331	1.36270797	1.56967304
Ahr	10.3028297	5.65729873	7.15519172	11.3767863	12.0133134
Fnbp1l	8.25851603	4.59208336	5.92520556	12.0975172	9.500461
Bnip2	15.7806448	7.86677807	8.51796423	11.1160242	17.8031712
Ebi3	1.83773335	0.83081778	1.18604149	1.22704955	1.65117438
Dip2c	2.46250523	1.09760633	0.7676057	2.65623707	2.11660858
Papss2	0.24050676	0.13489097	0.22270541	0.70973147	2.20263173
Nutf2	21.0734976	9.30282255	14.0437337	22.851066	16.4883518
Dnajb9	35.9451029	41.4304337	55.301635	58.6499257	56.7966897
Zbtb49	2.00987452	1.47524174	1.27445691	1.70483497	1.9432414
Zfp90	5.62426046	2.45128459	5.00149167	4.3674013	3.55398228
Hgs	10.5621409	14.403498	9.44813165	15.5027352	19.5336433
Plxnb1	7.59033735	7.10910537	13.1512874	15.5174149	20.5687705
Dhrs11	2.29457036	1.18912891	2.67716669	3.45483674	3.01555939
Anp32b	102.087063	55.8868181	61.975223	96.1770775	134.217896
Cdc42bpa	1.8532897	1.87521509	2.14165775	4.82776287	3.19364424
Mfsd6l	2.18427504	3.50478166	3.1071884	3.2550567	6.90001119
Sval2	1.4991193	3.64906427	1.66579261	2.61500244	5.04554571
Ddhd1	4.74221213	2.57106649	3.09011259	7.25624653	7.17810899
Gcn1l1	1.9381897	1.77951903	1.46263647	2.29082872	2.14630946
Helq	2.19258392	0.63866923	0.27834726	2.07791038	1.37444996
Arhgef11	1.05968996	2.45965009	1.11065235	2.49101102	2.02497571
Slc25a25	8.88128937	5.59182769	10.6758821	6.43241658	12.5997757
Eaf1	17.1876964	14.6557796	15.5593205	20.5405094	28.0029828
Cbx2	4.53343296	2.72452345	1.35624024	2.86542591	3.80054092
Dgkz	3.17632972	1.66271549	1.03564456	3.34055098	3.68060618
Phf13	10.5697975	8.49590581	8.16587193	14.10615	16.656223
Bmpr2	1.00411498	0.93444401	1.29138114	1.65593667	3.14408878
Ighmbp2	1.37176693	1.45457185	1.8022127	4.21951794	2.90319098
5730559C18Rik	10.9732249	8.37831845	5.6717391	14.1676609	11.5684404
Pop4	32.2298301	16.6183006	17.9065845	21.7484173	34.3934866
Fam109a	11.7855816	6.22788423	2.22402932	9.3586614	12.7790137
Rab24	14.2661498	14.8260001	13.9225428	23.4984924	23.4808219
Ctxn1	47.3928347	41.8491197	52.5536508	53.2284455	77.6189185
Mpp1	2.75916018	1.77070463	2.99713906	5.06628027	6.84576686
Ust	1.47654853	0.44224338	0.63429603	0.47515709	1.52478518
Ccrl1	1.14406473	2.59786317	3.77942989	0.96518395	5.94679234
Carm1	3.93134486	2.41861434	2.25177062	4.93023465	3.54012474
Rarg	10.4623116	6.54015287	7.01669729	9.00844546	14.0492738
Swap70	14.0120285	15.6596012	13.0198103	14.2441143	24.9436989
Sgtb	1.88041101	1.23042505	2.90205089	1.9477049	2.39553504
Ip6k2	3.68187607	5.0871031	6.78593446	9.84828839	8.82305273
Ptrf	100.978822	52.4775665	40.3629883	54.1023485	103.459334
Itgb1	79.0967727	54.1836113	58.2108923	109.532885	152.560817
Mospd2	1.3592511	1.4230562	1.64822532	2.10955597	1.50418431
Gas2l1	6.23994882	6.60551809	8.03568427	12.0337951	19.4599231
Klhl26	6.31080867	10.6947397	5.17473127	6.22302506	8.46267401
Urgcp	2.80092981	3.21226577	2.58285434	4.55612217	5.11305174
Polr3g	2.00323491	3.16263777	3.88752989	2.68044497	6.5577961
Rusc1	14.8686726	5.9307788	8.4715006	16.673784	21.3001425
Ttyh2	2.01899618	0.56301367	0.73384527	1.02208269	2.34372189
Ubald1	33.1233979	38.4219307	29.7132451	50.3666511	65.7553906
Mdfi	0.31412182	0	0.2617846	1.15356056	2.24032368
Hdgf	162.869463	77.5938033	106.944627	200.677145	190.501056
Strn	1.07528154	1.03916005	0.70464464	1.35170258	1.76312512
Foxo4	1.01055433	1.0849807	0.45833003	1.57153473	1.12381586
Strap	83.9070226	51.1465125	86.6035748	84.0079165	101.767605
Slc18a1	0.20914589	0.24633392	0.77466302	1.40169816	2.91622648
Camk2d	7.71839769	6.79514235	8.67670332	10.9378797	12.5811451
Dnajb5	1.073211	1.5966791	1.3076003	1.42608861	1.65214073
4933421O10Rik	1.21954329	0.74479435	1.67823197	1.46175598	1.31661596
Fam81a	2.85004262	0.92356566	0.48173936	2.15971504	4.80340652
Brwd3	1.84465768	1.49E+00	0.90098823	1.79590768	1.53507239
Neil1	3.73434461	4.37646252	1.90961054	4.2983441	3.48382918
Dnajc25	8.42362273	4.68044342	6.74249389	8.97883044	11.2981324
Hivep2	0.94799958	1.21091987	1.93494056	0.52710915	3.24201548
Il17ra	1.90542972	0.86415843	0.98867099	2.8803855	2.61945343
A830010M20Rik	0.7818453	1.50824714	1.72502983	1.58624821	1.53925845
Cxx1c	7.32946511	6.66950822	2.60061372	10.8796997	13.0643594
Gabarapl2	73.5960496	73.149445	78.2540073	97.1967758	121.685176
Sulf2	5.8853037	1.10263983	6.39937627	1.75278126	6.75186831
Mul1	7.00693663	7.97156466	11.6660161	11.7750343	13.7784884
Gopc	7.52268274	7.19972184	8.99410319	8.04365991	15.0223489
Rsad1	1.94802445	0.67845185	0.48005406	1.49998999	2.03943746
Fmr1nb	0	0.05505872	0	0	1.51715142
Lpcat2	1.4905529	0.73149445	1.05673719	2.77966895	1.99698066
Atg2a	1.20343339	1.63951674	1.46676455	2.97460327	4.78064887
Rab5a	53.1844913	46.2660957	54.8931161	53.2344773	92.4894752
Garem	2.5359344	2.74061897	1.96948718	2.67868193	3.57269123
Fam122b	4.40863522	2.06756046	1.71374084	5.16642882	3.87047474
Zfp407	1.42660879	1.56695953	1.5042794	3.16458945	2.41859158
Cenpt	6.45842992	4.75243706	4.06667395	6.30208345	10.8832694
Nudt9	20.6780695	11.7839698	13.8061616	28.0050605	27.813179
Mvd	4.47805593	1.31857369	2.13809486	4.30394433	7.59751374
6820431F20Rik	1.25972356	0.5605141	0.49251542	1.28503526	1.99652799
Nfkbiz	12.8472746	14.3790155	30.0212082	26.7700414	30.4138826
Smim3	7.78205963	6.98713481	9.85132638	11.3348651	13.3143321
Cxxc5	30.4818725	14.7618929	14.8570444	20.4221344	36.0419916
Ppp1r12c	5.90447893	6.57658156	6.94569042	9.93814468	17.4191459
Ppap2c	112.159352	66.8521933	92.9620562	136.798476	144.20005
Itpkc	5.24854804	4.20075123	3.56530348	9.15349225	11.0750692
Dpp9	6.35577025	5.38458297	4.73236536	8.80387947	9.73963074
Mafg	15.3064112	15.3363445	20.8542267	26.8974925	35.5197254
Celsr2	2.98750318	3.07431652	2.72056023	2.6405628	6.12635806
Sc5d	21.1089862	10.9512753	13.8812965	14.7744486	28.7195167
Appl1	5.61152163	5.72291162	7.84198014	8.51473852	10.3824215
Trp53inp2	17.8196661	18.3133931	14.0908524	24.4562168	48.6310319
Cdc42bpb	1.62107058	1.6539745	1.10558988	1.98734204	2.36473073
Arid3a	1.13869454	1.92281681	2.38093245	1.83228994	5.92630349
Lrp10	30.267577	27.6593303	29.7113358	41.9406583	79.0454469
Uchl3	65.9128905	28.3552419	40.451445	75.2556858	105.582501
Lmo4	25.1426231	42.2831638	48.6852076	39.6210049	100.459759
Heatr5a	9.99055519	13.6242137	21.7933687	15.0658041	20.3010146
1700030C10Rik	2.45867739	2.73249667	3.39753089	3.92418015	6.21390646
Tnfrsf1b	4.91111849	5.02353404	9.27959728	7.6040608	17.9119734
Gm6654	4.79191329	3.27186016	3.18323388	3.53862993	5.50957011
Ppp1r13l	1.62508364	1.89767889	1.48550914	2.02753931	3.57287274
Rprd1a	3.79402429	4.42097065	3.17593808	4.65359804	7.12656702
Sgms1	15.3726609	10.2544596	14.1417131	23.0958377	35.2297333
1700086O06Rik	3.47840705	1.48496025	2.12645198	3.47250999	2.73329943
Ccdc68	0.89799704	1.17518781	1.14830458	1.72739074	2.2273662
Pkdcc	17.5651698	22.1385871	32.3824924	15.8091384	46.2735991
Dsp	10.4765652	8.09280558	6.15685075	6.32277941	12.023787
1110008F13Rik	20.9919534	14.142226	16.0451524	32.4437258	31.7028455
Map2k7	8.38593639	12.1152479	12.2779391	18.1331521	13.6464713
Hspb8	38.256085	50.2720772	38.6570525	18.2054392	58.9770751
Sh3bp2	1.84997701	1.39208755	0.62454916	2.29996249	5.21956677
Tmem30b	42.2226814	30.7820532	29.401341	67.6408262	54.6298808
Pnkp	2.82718616	3.31768803	3.19329824	4.5351582	4.97926991
Slc5a9	0.40383386	0.68116226	2.90845097	0.30209529	1.91770414
Ncor2	2.04244066	1.18986917	1.51843691	2.59426808	4.20030595
LOC106740	2.75711187	5.0828107	4.2180686	5.94295737	4.41882745
5430416N02Rik	6.14614965	2.94467415	7.71693061	10.2333352	8.51479017
Esrp2	3.90489159	4.67587021	6.0113792	9.09698199	9.03553002
Galk1	14.5059203	12.5801502	9.42262336	16.0209339	18.1131358
Syde2	1.78011891	1.03434247	1.76924151	3.22501523	2.87201932
Itgb8	0.73721225	1.78620738	1.1507474	2.3205346	2.0423611
Vps13d	1.26317046	1.80923607	1.29921628	1.76409825	2.14318471
Anks3	1.99600822	2.47464897	3.3560723	4.19546675	3.17795936
S1pr1	2.49737735	8.01287087	23.7233835	6.56215622	14.4919933
Tpcn2	1.63645697	0.77449395	1.2039632	2.96364736	3.44909867
Meaf6	17.4195609	12.1581726	15.0962455	24.1939574	24.8163174
Hist2h3c2	3.19665145	3.26303897	3.78886162	3.93262364	7.37752062
Serinc5	17.0693253	20.0370058	21.7899174	29.8110589	55.1071287
2310034G01Rik	2.81370234	3.11906263	1.99239788	3.41896025	6.3000718
Gtf2ird1	3.50166032	2.36882135	2.33209245	3.16535873	3.86372629
Lrp6	3.48826142	2.6952673	3.54598976	5.42701837	4.5919145
Pcdhb7	0.11160015	0.87629113	0.48225314	1.98086023	1.7289968
Gstcd	0.92128007	0.70384346	1.59089873	1.7830637	1.25705177
Synj1	1.89437748	2.11080014	1.82934169	5.06023563	4.17105292
Zfp821	4.18542935	11.237451	5.07583907	7.13975169	7.5735417
Lrrtm2	0.51755309	0.53281695	7.51883551	1.68944742	3.56677749
Hgfac	0.77522218	0	0	0.34162507	1.56548672
Nras	54.036711	49.154136	48.0513157	67.1693536	87.4464506
Unc45a	4.45669837	3.71912134	7.32836435	7.17410021	7.54313612
Lepre1	1.05337458	0.99560268	2.14951028	3.25943832	4.48635496
Iqsec1	1.19663259	1.30041072	1.30308167	1.72261882	2.52388388
D4Wsu53e	135.631097	160.127495	194.554864	303.664666	226.90438
Carhsp1	50.4709636	59.5260349	30.9873091	52.1155093	88.3185548
Mob1b	5.24408099	1.6470736	3.00877552	3.56839825	7.51478764
Mctp2	1.05888734	0.41572236	0.33379158	1.11693516	2.42826926
Hdgfrp3	6.05664124	3.50788239	6.61682745	5.99922259	8.33554221
Smad1	33.8261554	45.84208	60.9004152	82.7288108	98.235748
Yif1a	10.6138249	10.5976055	13.0234188	16.0000317	20.5109568
Pard3	3.27725538	2.80958088	2.6054625	4.24814966	4.54214167
Tln1	2.30069279	2.47050287	2.88521702	4.46925205	5.57982454
N4bp2	0.72824082	1.04535675	1.16955732	1.52521196	1.98780691
Tcf7l2	5.41401683	7.34386368	5.5703256	4.10265217	6.69143118
Sox13	7.59048553	9.86098438	12.0564748	11.2753929	14.0152346
Sult4a1	1.69665327	0.4082617	1.11495561	0.87932973	2.25871426
4930413G21Rik	4.69784639	8.83845648	7.92499051	9.35198618	8.75927094
Herpud1	17.5315232	7.88184293	11.1668647	21.3112143	26.0588933
Rfx1	1.84443417	3.42427771	2.96714317	5.1948382	4.27128146
Chst13	0.15799319	0	0.29259834	1.16040786	1.64590355
Cd248	1.01813723	1.63886735	1.76771025	1.89283888	2.16209539
Wdfy3	3.41200057	2.91224659	2.34315893	3.81603358	3.55410605
Midn	2.35662524	1.91948247	2.13334214	2.24430992	3.80515323
Map7d1	5.81628182	2.67667343	3.63807925	5.49896797	6.62092167
Pim2	3.92995483	2.45862417	2.48522219	3.57869413	3.5077261
Shisa4	16.0015238	7.87432261	9.13386283	15.3447788	19.6733883
Eftud1	2.61779122	2.22445109	1.99235575	5.10300681	4.16680147
Zc3h15	99.5458767	85.3932156	114.491403	82.6642552	180.370728
Fam83h	8.08805311	8.06572208	9.77276395	15.4072868	16.2147106
Sprr2h	0	0	0	0	1.539041
Zfp281	7.66469147	6.70756143	8.31080511	12.0988055	12.8885169
Tpm1	95.5602831	111.622618	60.2008253	63.6493131	200.726572
Dennd1b	2.7455902	1.37358696	2.42614989	3.45693499	2.21275329
Lnp	2.92802344	1.45298556	1.06662931	1.07799741	2.58458438
Tnfrsf9	1.26254059	1.59077812	0.73408128	2.07562992	4.25871059
Tpm4	298.11953	244.931185	415.095996	436.543383	489.593458
Atxn3	5.43028503	3.3447868	5.08422776	6.64727529	5.60868132
Samd4b	11.4651388	11.7778769	9.72265596	17.2342105	27.3485748
Snhg5	44.2922961	22.0534713	40.6552354	60.1069009	44.341212
Cycs	40.0991134	13.5418538	30.2615814	38.986802	40.401713
Pitpna	28.3063658	19.3050756	21.7749935	32.5771585	39.8962193
Slc30a4	16.5887296	12.9414139	7.23384737	9.19475817	20.0689174
Filip1l	4.93441568	1.92460459	2.34432299	2.5167816	7.61118467
Dcaf12	24.9594249	22.6077107	23.4616809	27.577545	41.8029243
Dtx2	3.04972461	3.83520108	2.89018365	4.70296144	4.71596168
Mir3960	1.78662163	4.20859821	0.82719154	1.6402683	3.57927656
Taf3	2.97218731	1.49191376	1.9981711	2.06834248	1.79448017
Gga2	3.79990422	3.47575772	3.50630442	5.60629014	5.91916183
Nudt4	81.4591083	130.143073	452.317343	143.068607	351.682366
Ssr1	40.7555541	21.5470942	25.5400053	34.0072775	44.227335
Zcchc9	27.0296494	14.9402489	19.3531503	30.2866119	52.1551059
Gm11110	0.31779576	0.3493492	1.53022372	2.10069448	3.61625544
Lrwd1	3.02266207	3.52487	1.99528165	3.18719411	2.82991173
Polr1e	2.5030302	1.32185818	1.84600828	4.12824998	2.80457716
Npat	5.28119712	2.11871538	3.95333828	6.22574135	5.12118622
Casz1	5.23227909	6.53611806	9.83920001	14.2419672	19.1938495
Cmtm4	6.62912416	5.45688048	5.19348332	6.59713702	10.7123692
Serpinh1	66.0388427	48.1457436	54.9987602	82.35771	146.188258
Ccdc41	13.6931445	6.05332481	7.30039468	8.26066799	12.7580951
BC020402	1.19216983	1.68497808	1.51790403	2.10693512	1.76739049
Tmppe	1.5503946	2.22563802	1.77913745	4.05186462	3.40709957
Hras1	32.2040122	18.5932238	28.8635962	42.7942533	43.0111833
Scube1	1.68537523	1.21362418	1.65198451	0.97367666	3.82114396
Pfkfb1	1.66723669	1.01159604	1.68418311	1.42629954	1.24494767
Esco1	24.4184741	16.0606535	21.6401687	31.4777963	37.0866661
Ctnna1	69.8772702	52.6950613	62.4711879	89.7884025	158.065955
Manba	8.21802184	6.03472967	4.7170631	9.80991161	12.3176214
Tmem138	6.60641299	5.91251506	2.30225966	8.08698726	6.54641104
Fbxo30	8.53082133	5.17653191	5.41977058	11.4494833	15.9204317
Usp12	5.24867132	2.93521989	5.4818374	3.81015059	6.33349385
Zbtb10	4.87686776	4.02184449	5.62663501	7.7972206	12.0610279
Dot1l	3.09222975	2.11329184	3.02243061	3.42774016	4.77236874
Hs1bp3	3.90211935	6.90691007	3.61330014	7.89358323	14.1685142
Luzp1	9.4928957	5.76043379	5.87087354	13.4705046	12.8891037
Adamtsl4	0.40588189	0.38509692	0.09396003	0.83842552	2.12770169
Pim3	32.3497426	53.8717389	37.7437341	37.1989659	38.842031
Ccl25	1.27966424	1.84883213	1.99071369	2.25569078	1.84582786
Rab35	12.330828	10.6291588	10.9993911	16.4136787	19.4968804
Xpot	8.37331596	4.49718245	6.39239917	10.8163493	7.4603622
Otud3	1.67910338	1.22645776	0.57853875	1.25475614	4.59990619
Cpsf3l	10.7250584	6.89418715	6.61224558	11.9637549	17.1000052
Wt1	2.29885969	2.30188907	4.35506177	4.56962196	5.44207469
Ap4e1	2.51890646	1.13020602	1.11995445	2.14738374	3.27610239
Rab9	66.8780103	51.4875099	50.3764215	73.1006862	98.4879472
Cygb	2.23806742	0.70293761	0.85487105	2.38862751	4.97689883
Ankrd11	7.50052781	8.85161388	8.69883475	14.9810303	12.6553573
Spata2	4.16140661	7.29114714	3.44538937	4.73444808	5.53175579
Pgd	37.180046	28.065837	23.6761261	37.5532198	61.4917781
Coro1c	15.0882693	8.85201957	7.18785147	11.987898	24.0299044
Ehd1	24.6746933	37.864378	24.3096047	29.5585935	47.7411601
Btbd6	14.1426686	7.44394972	11.16748	16.2087904	13.8738765
Mir3064	60.3451455	112.344446	122.572501	148.334114	113.094455
Kif3b	3.6942868	3.47365683	3.63532391	4.98649135	5.63314947
Pdxp	2.18349985	0.84445386	2.95738543	0.92751803	3.34280461
Eif2c2	1.11243948	0.82886309	1.3007847	1.93825752	3.20793385
Homez	4.49967374	4.2978124	3.99042535	8.1593916	6.00969503
Zfp593	4.78527074	3.57603056	6.3257547	9.63491605	7.8544833
Pak2	29.7183303	24.8465412	19.6372728	30.9938278	38.2974733
Mir684‐2	6.82447914	1.78620738	2.80860382	3.48080191	7.29173549
Fbp2	2.26779446	1.22669471	0.04666537	0.04626723	3.35190675
Gorasp2	41.0592119	23.9325902	24.2123358	33.6225286	45.0415355
Ocln	62.2493791	51.18857	57.1122371	99.6141822	137.977972
Sall1	20.8151446	34.587707	39.1347981	40.8166896	34.9708324
Ypel5	46.5050947	67.80283	71.4980077	74.2685927	101.891986
Mir707	2.67993245	0.70143304	1.65438307	1.6402683	3.57927656
Slc28a3	4.71513093	2.92822165	5.71566876	6.84750917	6.44630531
Thoc5	3.67636371	2.4743168	2.43161002	4.50027973	3.6007362
Arpc1b	96.7033436	79.0353578	99.901911	136.723924	216.138919
C630043F03Rik	6.62552707	8.48010872	23.5966621	6.23875771	20.0122454
Zbtb44	4.45143543	5.10717326	6.30898041	7.15357222	7.24634897
Tnfrsf18	3.49695257	1.47461145	2.5785047	2.67541279	4.25532263
Mfi2	1.64133972	0.21066757	0.10229789	1.37648362	2.13734438
Tbcel	5.7373683	5.082318	5.66527112	8.61826089	10.5735303
Orc1	3.89711292	2.51603005	3.04731649	1.98770892	4.18128901
Lsg1	7.84693223	5.3606913	8.44655266	12.4316946	10.3970054
Ccnyl1	4.6372757	1.55802066	3.6127767	4.44162223	7.87881368
Ubtd2	9.91510187	7.04232289	5.29762705	12.8941209	13.4780067
Pced1a	1.42773267	0.784745	1.56994343	2.7198647	1.91639208
Twf2	10.0640374	6.48891564	6.06122781	11.0800432	21.047224
Paqr5	8.16077715	9.36312897	17.8567018	14.6766521	22.1571536
Nol9	6.26215822	5.41909036	6.36030857	10.0836504	10.19401
Cars	3.15736299	1.23959022	1.2931607	2.26695057	3.87632763
Kctd3	8.40020954	6.44378854	4.98995531	10.6708245	14.1545261
Tob1	36.9580472	29.8488151	27.6423156	32.5943205	66.1622614
Ulk3	4.19976203	4.34858697	2.69232404	5.65648785	6.65699843
Apaf1	1.26306002	0.78091523	0.8196223	1.39699227	2.14385401
Snord19	1.20762388	0.94823355	5.59120204	1.10869987	1.93546066
Muc1	70.8137676	34.5029731	46.7409481	24.5104521	97.43031
Prep	12.1117702	5.44249089	6.30722137	13.1453719	20.1995465
Cux1	4.24431313	3.54289815	2.94762458	6.25464532	6.23815216
Slc4a4	1.39266822	0.83381775	1.06391052	1.19068328	2.05765405
Fads2	0.64865739	1.25634657	0.4805171	1.54835671	2.04455492
Zkscan6	8.49848256	6.33940673	5.37746365	8.29646564	11.1915364
Gm13375	1.74211734	0.96124181	0.65398898	2.07490979	1.62243308
Fam160a2	2.04194622	2.36307465	2.50642197	3.57496669	2.87306503
Tpd52	122.473861	118.437092	137.059424	143.141306	228.099708
Tle3	18.3901477	15.837797	22.6356622	17.6778166	33.5524494
Cog6	10.4964021	9.67977588	12.6973901	21.2824811	16.2857083
Eif1	446.681226	450.524472	503.573851	735.444292	917.374788
G630090E17Rik	0.21956798	0	0.64383539	0.80632718	2.34601292
Fam181b	4.3545265	3.11341829	2.0652844	4.87539029	5.617249
Erf	1.02387751	0.49699024	0.94809421	1.8116466	2.13326109
Serpina1b	3.93558267	11.1751819	10.1277268	0.33611112	13.2752228
Myl9	4.33540318	3.21506333	4.90662827	7.96052646	6.51408503
Cd164	104.131316	63.7253151	95.3997366	122.807119	187.332758
Pcdh7	1.17614837	1.0699303	1.50082546	1.01396108	1.36203813
Wsb1	19.8664558	9.60658287	12.4618203	17.3297911	29.7546816
Agpat1	28.0760492	21.2500274	16.4832508	34.3859351	29.5035334
Usp28	1.85436084	1.2619146	1.86020087	3.23467127	2.3652063
Ccdc109b	15.140195	7.18729373	40.3177422	13.1731714	23.3929349
Rad23b	27.1387404	16.0425889	19.0139015	23.2661147	33.0328437
Rybp	3.6510201	2.57427206	3.7119653	4.96634707	5.80372823
Ext1	4.27339882	3.28158885	2.03956204	4.42455743	4.57101721
Stk11ip	2.70618272	2.58435119	1.3951667	2.8336757	3.9034829
Prelid1	115.5726	81.2973125	73.9147668	90.4918998	166.417625
Gpt2	42.0539233	44.7114795	43.8949786	78.8330002	103.148858
Hsd17b12	66.4173434	42.7679627	65.113333	92.84864	123.809492
Rap2b	8.29712705	5.96594116	4.15803188	8.68732519	14.4682255
Klhl18	1.61229784	2.11374445	0.8797812	3.13226068	2.99932314
Thop1	8.12308086	3.90224699	4.85883028	9.24102411	7.50000363
Nrn1	0.08171891	0.67374489	0.68103363	0.67522323	2.65216319
Ecscr	2.34785561	1.79746161	1.68490949	4.84993821	3.33958423
Sh3bgrl2	35.9595888	32.2003857	35.437358	20.184673	61.6637765
Jund	270.204794	191.741521	220.012829	149.154418	244.636712
Abcc4	7.59973379	7.40925083	8.85031798	13.2434622	22.4897573
Proser2	12.0349297	8.03735833	8.22901548	16.6314155	17.2701822
Phrf1	4.92667398	4.37344933	3.17993113	5.18039703	5.5913708
Csnk2a2	3.99997352	3.90221017	3.17478132	5.21436326	6.11936538
Ctsk	5.80832862	2.21126528	2.77070093	6.38287939	3.84349576
Crlf2	4.79497717	3.57679272	2.26443683	5.41469449	5.03362084
Gm3414	1.69787766	0.88663192	1.27455662	1.92200801	1.90219187
Exoc6b	5.14261302	4.14154946	4.87297925	7.18657623	9.95196791
Acly	22.6112364	17.1645908	17.2820809	28.9273745	38.5999145
Gpsm3	2.76773366	1.44882743	1.70858475	1.91690327	2.25683648
Zfp553	28.0995467	26.0967085	30.2267877	37.7444926	44.1249853
Sgms2	83.4574472	83.8818204	45.2000885	126.274596	100.868588
Dstn	593.563663	694.307583	809.730828	632.760689	1061.34856
Dnajb11	30.5704985	19.0672302	21.7580003	29.6388776	44.9258936
Cast	7.97682726	8.49281739	8.86787055	15.9707948	25.4786609
9330151L19Rik	1.02453558	2.55419704	2.82239311	4.20922738	3.16089765
Ube2o	2.73808712	1.61740298	2.00042699	4.3011234	3.97567178
Fut4	7.72431039	8.46888499	9.32802943	12.1406267	14.5761739
Rps2	430.37104	217.991204	190.342651	478.179256	364.180462
Gltpd1	13.5224841	9.81004954	9.25509867	13.4718279	17.0199844
Slc6a2	2.75965819	2.68965439	4.05682079	5.43195387	6.90971883
Abce1	34.683794	17.3904988	32.4650441	41.43624	37.9767532
Ino80	2.76548938	2.27720636	3.30889752	5.08741092	5.54446766
Tmem170	4.09460943	2.42451069	1.86469322	2.24935403	4.33013251
Siah2	6.47399008	3.78683622	4.75701627	4.83674773	14.0725415
Plekhh1	2.01539466	1.99601763	2.31636754	4.35146188	3.91180074
Pprc1	1.92750036	0.92761977	2.85573523	3.01403991	2.63080922
Kcnq1ot1	2.44474472	2.77879695	3.94716603	3.13495361	2.68185995
Rai14	3.81453884	0.27600809	0.94031316	1.64943729	3.58885178
Mllt11	8.06715809	7.59718381	5.09213055	10.6212918	14.6961052
Prkd1	1.86467103	0.55583507	1.34295433	2.52033282	1.68795536
Vit	0.28769863	2.54140535	2.81944585	2.68533629	3.24687756
Rnf208	5.03886273	4.87845886	3.75990511	7.68022098	11.9568781
Wdr1	96.2087678	76.3443322	97.5643386	134.488607	166.795282
Pxk	6.88855875	8.70838992	8.27104155	14.2296162	16.247647
Ap5b1	4.66099075	3.16302743	2.7341195	3.17550001	5.18856189
Slc44a2	38.4698135	32.4305377	23.1150753	60.8365099	47.005507
Dvl1	10.6738369	6.94351662	9.12628462	14.1627467	16.3421557
Fbln7	0.17933775	0.05280641	0.35288577	0.74091184	2.19161478
Bpgm	22.4835274	7.30433288	6.39473456	13.6067711	27.1142315
Fez2	8.66759243	10.262358	12.0706767	10.8711764	24.693075
Golga3	1.93590858	1.21181767	1.15329424	1.80218392	1.71406999
Serpinb1a	11.5939448	10.2516011	19.3622336	10.1447772	16.3474779
Pkp4	8.86166652	13.8831749	20.2461134	18.9055674	34.6684042
Fus	12.801861	6.0534736	7.46625591	12.2726582	11.5907224
AA414768	0.46886775	0.88971464	0.25326236	1.72183946	3.53810094
Spats2l	0.87056396	1.87349382	4.64270196	2.60496503	6.60161945
Mesdc1	21.7824333	34.7264964	19.0086335	26.005309	45.1394503
Taf4b	1.83641386	0.60661901	1.51284962	2.52315887	2.4053239
Aen	5.78816934	6.54942706	12.2174266	13.1110205	17.4191459
Rere	7.61589992	6.63229482	5.39238375	14.1526475	8.94780745
Hoxb9	1.54633128	1.13830185	2.08069779	3.0833276	3.89171729
Zbtb48	2.22645481	2.20828513	3.22812797	3.52333462	3.89700569
Alox12	3.68464578	4.36548845	5.79421259	2.10174799	9.7491308
Amotl1	3.90541515	6.24181354	8.34594668	4.94343876	6.21250433
Ncs1	7.28884064	4.64458186	2.69375385	7.43364713	15.062057
Gm527	1.60466222	1.43498911	0.41274766	2.86458339	4.00056939
Prdm15	1.34616	1.65410422	1.60810936	2.86801403	1.95985978
Dock5	2.00020373	3.8248631	5.48051409	6.0478049	6.00189439
Cerk	6.7851142	6.09364163	12.3532849	5.478626	13.024126
Blk	0.10539263	0	0.14638784	0	1.58355872
Nolc1	10.973207	4.19548371	6.75740657	10.0743231	8.11807872
Ldlr	2.66140802	0.982749	1.65182832	1.70377306	5.27992642
Ogt	37.3537984	31.9648402	39.0640588	51.1183948	65.7003335
Mafb	2.34754386	0.6799078	1.06907611	0.74196852	3.14562328
Cmip	6.81871278	6.81430191	7.33446656	11.7235526	14.5601099
Myl4	0.22155727	0.52190431	0.95740628	0.40681626	1.65708749
Cyth1	10.4402461	12.4843508	14.0400157	23.2491618	15.8704226
Thsd7b	1.84590901	3.36559977	1.13952149	3.35109987	5.4238089
Dnaja4	3.16835497	2.23221435	1.82863007	2.17164972	3.56498327
Wwc2	1.37396921	1.30276094	1.14024514	3.24874845	2.36825597
2610005L07Rik	0.39039176	0.28099313	0.33137182	0.85122928	1.79883422
Scaf11	11.3470366	8.47222858	10.8902486	18.2539309	16.5418741
Sema7a	0.07964787	0.34396991	0.03687633	0.23765109	1.97860222
Ccdc84	2.19624861	1.59185321	2.81587999	3.25716489	3.79069496
Ubash3b	8.16402897	12.7893109	9.77555213	5.15068523	11.6181352
Prune2	0.89124032	0.76119387	1.07140873	0.37322921	1.70404688
Ptpn23	2.17535246	1.65052346	1.43719879	3.68015218	3.33954015
Irf5	0.43619859	0.49235203	0.58062483	1.6769549	0.96125722
Rhno1	24.6584825	11.6695231	11.5709598	25.8201866	44.994268
Arhgap8	8.89441919	2.71414231	6.83088032	5.88248772	11.3500317
Ccl7	0.32646653	0.89720221	0.45345418	0.37465452	2.09291365
Szrd1	22.9041057	24.6755965	16.4854706	26.3537278	30.3285497
Prickle2	0.60882283	0.57838312	2.01144073	1.96667715	1.72866351
Mtrr	2.36683814	2.66286171	2.64961189	4.03780563	4.11888255
Cers2	59.6184212	62.3746805	73.4978339	130.365954	125.319398
Ube2n	54.4587883	38.3868381	48.7658515	53.9776475	79.8177742
Vamp4	2.4209711	1.57739325	2.33717545	3.71230548	6.99655268
Vezt	5.4306676	5.75037276	5.13656579	7.08439164	7.94537063
Tmed5	44.7146002	15.562052	41.0025458	43.89281	35.3305417
Krtcap2	78.9870271	41.5789384	51.2570196	81.1026844	83.4498618
Sipa1l1	5.33619488	4.34298108	3.43533214	7.48080798	5.51828768
Gas1	1.4579426	1.12843124	3.20150336	1.64445934	1.96945949
Ogfod1	1.05596033	1.48475055	2.09204231	2.25455819	2.00725236
Fam134c	6.09510347	11.1981047	7.65314806	14.6686425	15.3800626
Ccr1	1.35905676	0.23513244	0.89585632	0.23262724	1.84590031
Ceacam1	7.06925435	7.62038651	7.62893777	0.86535568	14.1973499
Lynx1	1.48508402	0.49235203	0.38199002	1.12104369	2.08923967
Gtsf1l	0	0	0	0.6123047	1.54396975
Susd1	6.86751328	5.09715101	6.4325125	7.10442761	10.721101
Ano9	3.68060665	0.45900548	1.08259928	2.14672564	2.86270824
Dcbld2	7.20776652	1.51553652	1.73140229	4.17159648	8.66795174
Cldn12	7.31151671	3.73105241	5.85183217	9.66494692	11.8592075
Rcn1	22.2056129	12.5712922	12.2031681	20.0543282	33.3710572
Grpel2	16.7783907	11.9215878	18.2826503	26.2207792	31.1917232
Tagln2	270.803102	99.0375735	151.268313	209.197706	324.434743
Jak1	25.5973418	29.5593332	52.3853109	51.4525451	54.8116321
Dppa2	0.53755128	1.53007082	0.71553559	0.09253446	1.53460791
Mfap3l	1.25831954	0.21118414	0.17789065	0.72312903	2.67098702
Dock4	0.4528028	0.43173138	0.68883055	1.30651997	2.416866
Eif2s2	78.497955	43.2987662	54.8583024	65.5875605	89.5841789
Heatr1	1.28960543	0.85391398	1.45259033	2.20005584	2.39847449
1700052K11Rik	2.94574147	2.92840298	2.02701349	2.25783305	4.15807213
Plekhm1	2.44162332	2.47635407	3.65041818	2.75531808	6.80732613
Ppp1r3c	0.02436909	0.22961709	0.72209246	0.58169455	2.10904457
Appl2	11.4701715	21.1077754	23.9489504	35.6575948	46.6324436
Cpne8	1.80802262	0.78564276	2.48691851	3.41652653	2.86958743
Dll4	1.09599478	1.37989825	1.41732354	3.74728347	2.46825916
Cblb	1.07276427	0.60745743	1.24170321	1.97924497	2.56244949
Vkorc1l1	7.73400092	5.9347663	7.16156121	8.76799301	13.3343327
Crot	77.26294	55.8080943	136.884116	140.05217	169.468466
Hps6	2.90259746	5.31798636	2.46377894	6.217931	4.49693084
Nfkbib	14.1822852	12.0574355	9.7174804	15.0213156	19.6939341
Rgs11	1.13779119	1.02102914	0.36122621	2.47716491	1.84959027
Rin3	1.82273752	0.35780612	0.90419239	1.25506928	1.87798129
Plekhh3	3.92658543	4.70142142	3.93727761	8.2057068	11.6649171
Pkp3	7.02444472	9.28241234	7.7935297	13.7041857	24.4211277
Snx25	2.72158339	4.55782815	3.89834576	2.83049232	6.44059715
Cd38	0.95803484	3.59030665	2.60088904	3.17839635	3.14068073
Calr	175.933956	114.215536	97.4612989	135.793194	229.12049
Esyt3	1.61177857	0.80951401	0.51093951	0.65322197	1.61740901
Bag3	33.5740862	46.5640085	64.8795064	60.4470179	105.865145
Sars	17.0775753	19.3537949	15.3896931	34.3152187	35.9058259
Foxf1	0.16134851	0.31672956	0.34861433	0.37032862	1.98255021
Hist1h2ac	2.01415969	0.45186581	0.43599265	0.55234867	1.86558843
Slc6a8	3.09278447	1.35632801	0.71596724	1.54054462	3.91535293
Ruvbl2	6.46277043	3.69896	4.47029121	6.1478235	7.83081604
Krt12	0.45725564	0.60760596	1.30280436	1.09793579	3.41054475
C030037D09Rik	1.0498094	1.50316568	0.40027924	0.96381295	1.92996219
Tead1	5.02736173	5.5357726	7.67996108	5.85356135	8.62913923
Nop58	37.9191454	17.69876	39.2685739	38.9032459	30.0956296
Adam10	52.3796661	26.7794874	30.3043394	26.5624097	53.7833685
Khnyn	1.69927261	1.25501499	1.13728165	1.42105803	1.94145279
Ralgps2	3.86717295	2.84988515	4.68146573	5.48086004	8.34448641
Sbsn	0.11410619	0.33598827	0.58113281	0.57617473	2.05737944
Eng	0.67548503	0.98911484	0.64239343	2.56441162	1.55075263
Rnf113a1	3.19380492	1.86944708	1.88198965	4.69148866	4.60684446
Arsg	2.17052163	1.85513026	1.20947829	1.42841037	2.07798353
Dusp1	12.0215164	9.86123827	21.8281449	3.79195907	17.8945397
Ginm1	36.6894284	33.873314	44.9493861	70.5979059	82.9003668
N4bp1	5.01008034	4.39303747	6.33813616	6.92264724	7.9327259
Elk3	8.90230433	4.53512738	3.7794096	6.49037196	12.9594496
Ptpn3	3.15358775	1.6802895	1.34631299	2.03984064	2.57635978
Fam20b	21.0191524	24.0799412	22.5980608	26.2053954	38.3237651
Fbxo34	9.5646204	6.8479475	3.35656752	7.33133126	8.21106134
Ltbr	27.2754438	36.6901112	34.8108453	53.5737343	52.4073156
Tada2b	5.24652211	2.9850222	2.86713183	5.10101374	5.72061109
Cldn5	0.27632072	1.04868202	0.93818475	0.67649483	2.76787276
Ppme1	12.6225083	11.8197854	12.8391618	16.6347243	27.1357978
Sdcbp	374.973601	387.975717	1327.45255	960.496268	1056.89798
Csnk1g1	3.81301818	3.92784922	3.55601384	5.91779937	7.31880584
Fbl	22.9000497	11.3984381	20.371465	33.5766741	21.7776897
Pi4k2a	10.4103519	6.96578248	8.56694919	15.7017303	16.1026631
Actn4	44.7292985	34.2483744	35.9204288	71.5264147	70.5495977
Eif5b	13.0724472	8.97738925	11.4001146	15.0653499	20.810484
Zfp82	1.67061749	0.8895972	0.94240402	3.08516306	1.80038876
Fam57a	23.0695117	12.0028014	7.92147076	16.7377915	23.094416
Snapc2	38.5498429	84.2474817	97.2381679	52.3168665	74.9345424
Adssl1	0.91026926	0.4574393	0.87661057	1.20341292	1.98409032
Ppa1	30.5364336	8.56052329	13.6006422	23.4937964	34.1776586
Acad10	0.57629773	0.96158895	0.967227	1.57073469	2.55442322
Rcn3	6.01401679	4.22887363	5.36108925	9.06493765	11.4009689
9130221H12Rik	2.24138751	1.82636422	0.86152374	1.47539048	1.38946495
Syt8	6.58616399	5.9540246	8.10482815	5.41016068	13.5417945
Lcor	1.06285404	1.22987701	1.35973046	2.06713203	2.47670893
Gm12238	0	0	0.48697566	0	1.6857238
Polr3d	14.6696055	13.3948984	26.4954513	20.2435431	16.5788903
Cdyl	7.03735209	5.86461573	7.45398735	6.2932615	11.518183
Il6st	4.34186414	4.42358254	6.83373696	4.51329509	8.59774791
Rffl	6.47197579	5.41981862	5.21001967	12.3634933	7.99506767
Klf2	4.91960433	7.66941318	7.81413589	11.4069099	12.1435235
Tax1bp1	37.0141868	46.7140731	49.0214257	69.7508609	67.9314865
Grtp1	1.79207696	1.48532461	1.42895759	1.96519167	3.11151156
Gfod2	6.43789273	6.03590706	8.21544206	11.6152103	11.9606906
Clmp	1.89245258	1.83560403	3.77277286	4.47643722	5.0312726
Pi4k2b	14.7626089	6.48240483	7.26803962	11.7984749	14.0461214
Nup93	5.38976396	8.11439199	10.7034856	4.96870207	15.3309901
Mir1894	1.61016517	3.16077849	5.96394884	5.17393271	4.51607486
Arap2	3.7911713	4.40963376	6.15168929	5.22091918	6.53040515
Pde9a	0.5687066	0.17365905	0.14628145	0.1740401	1.59506714
Lrrc8a	9.58093699	10.5683237	9.85124061	17.8173854	26.4849637
Hmgxb3	3.5873328	3.06301869	4.57620368	6.94392865	6.45302462
Fam129b	13.3672486	11.3556937	12.5577217	19.8431157	29.8431996
Ahnak	4.30454123	2.87185439	4.74144064	3.65811763	9.38734428
Antxr2	0.73017621	0.61429153	1.1429842	1.34072583	1.58820259
Lgals1	183.978479	104.009367	160.322127	138.823082	354.828002
Parp4	2.4896968	3.36503471	4.30848878	4.00006283	3.54869785
Ing1	30.4846682	23.481674	25.6952803	25.9868528	41.5198625
Mark4	1.70318268	1.26934849	1.1494264	2.51776464	2.52149655
Arl10	3.11124473	1.4169215	1.72857773	2.91351091	1.94469472
Scyl2	3.64926972	1.79089285	2.11197839	2.70469772	4.7063679
Msrb1	30.3241717	32.0751235	30.8740371	36.2867706	48.9544845
Zfp568	2.47846182	2.10293198	2.58924885	2.39212453	3.98678489
Asl	3.07079244	3.48285128	2.48806662	2.78008848	3.75952789
Plrg1	15.317935	11.0736116	16.9118866	20.046746	28.3270131
Gpr116	3.78204878	1.9137986	2.40549431	1.0521932	6.24517709
Frmd5	0.37263823	1.03628381	2.5304183	2.56584826	1.55528088
Pla2g4b	1.03108166	0.79633922	0.73563871	1.53631661	1.70669703
Stk17b	31.113814	26.6488918	54.6006976	40.637672	42.9314459
Rbpms	12.8966922	9.99744216	12.9427465	24.5215974	15.3233265
Spns1	6.4461591	5.83460167	4.2760034	10.3082785	6.81863763
Inpp5a	3.48441795	4.81968389	7.35047198	8.70717793	6.34264017
Epcam	305.871617	174.309342	344.554774	344.171424	665.401231
Kctd20	14.2922286	12.5024593	9.63643672	15.9652781	19.2699302
Eva1c	5.08419802	1.21915742	1.94517449	3.1583971	4.85491601
Paqr8	3.39336846	5.38691049	14.8913191	8.43866084	10.8652908
Plxnc1	0.82382404	0.11629152	1.69712228	0.41641162	0.91979025
Pofut2	13.7241366	11.4454652	11.8103119	22.0161113	24.6803942
Sdccag3	31.967097	24.984118	33.5624957	42.6862076	54.4715342
Zfat	0.84652596	1.19187099	1.1217443	1.01837606	4.25732035
Auts2	1.92653778	0.53241303	0.8371577	1.26478519	1.7853259
Gpc6	1.08975465	1.93096361	2.49148329	2.87750409	2.31070303
Cnga2	0.04293067	0	0	0.94593484	1.78893138
Tnk2	3.9091875	4.42608339	3.02331634	6.156346	7.80224429
Vgll3	0.15661769	0.23058216	0.10876911	0.17973519	1.8041445
Cxcl1	0.13743243	0.37769471	1.27260236	0.94630863	1.54184221
BC022687	2.1453454	2.83430226	4.25690474	5.0334395	5.59413824
3110045C21Rik	3.10258259	0.54137034	0.47882365	2.37369223	4.23584517
Fbxl22	0.49716663	1.83477769	0.79797181	0.63901685	1.44753766
Scamp5	8.7670996	12.6928385	9.51853524	16.0415772	24.6138918
Xpo5	1.45930715	1.2701063	1.48154041	2.79252471	1.71890197
Rgp1	1.7558703	1.86738018	1.6567795	2.9996113	3.60672499
Emp1	39.1605662	12.288354	14.6468534	24.4671847	36.1442476
AI597468	5.92048955	4.58103724	4.81097925	4.81747406	8.71418363
Man2a1	3.27238653	1.88710645	1.98700171	4.89557207	4.09255353
Ppp2r1b	5.48644473	1.95560048	3.7879878	6.88171234	8.94739893
Rnf214	3.46577197	3.91277968	3.92773812	4.93902045	5.19534547
Pard6g	4.25338732	2.29712066	2.11337831	4.41927837	4.35617203
Yap1	16.2520857	13.2156781	17.2362985	25.992882	36.2254725
Sh2b2	0.6182288	0.3056455	0.23322851	1.3453886	2.25690479
Zfp653	1.05180144	2.76912985	2.03383953	4.91341279	3.79287854
1700001L05Rik	4.60598973	7.30154047	5.23074974	4.22868435	5.83249763
AI429214	0.66576508	0.70572972	0.36989269	1.95592993	1.62720965
Mfsd10	7.4955965	5.01676271	4.9577161	12.3213148	9.83938619
AF357399	1.38748276	1.63418973	0	1.27382538	3.33558113
Smpd1	17.6547402	20.8257562	39.2802806	34.9985107	30.5268365
Usp43	3.21527828	0.96395952	1.69164562	1.74430145	2.61170072
Cpsf2	15.1772079	7.82279296	9.7365813	13.8438336	15.632987
Mmgt2	25.8699586	17.1588475	14.3987557	23.7825836	26.7531439
Hif1a	311.101261	355.152002	154.621575	192.825751	265.293482
Leo1	9.54970454	6.68089366	5.39938708	7.2652212	13.8034017
Gmeb1	2.88354379	2.55984154	2.14710315	3.17498217	4.08943632
Ankrd50	3.42311616	3.24000696	2.34659849	2.07077615	3.94455939
Gpaa1	15.2139256	13.9831847	8.57134568	17.4081126	19.2500384
Mapkbp1	0.3102185	0.55360327	0.3917146	1.03566025	1.93601867
Mthfd1l	2.45371777	0.79194575	2.16058915	2.32182022	5.19920491
Nsf	5.16150956	3.8352458	1.8444284	5.8359134	5.66295739
Spa17	3.87489749	1.70682038	1.22520253	4.25162297	3.48382918
Paqr7	3.69122771	1.78361492	1.19776694	3.64953744	2.27536013
Mir141	0.90571791	0	5.03208184	0	1.45159549
Nom1	7.73261141	3.2382363	5.45544728	6.98086517	5.00148825
Fstl1	16.6959272	16.4723065	20.3625722	32.4315447	49.3904461
Fam65a	1.32537671	1.89554937	1.56331795	4.11396592	4.03341946
Ccdc64	5.13810398	7.18467296	3.12841785	3.52175252	5.24548484
Tmem98	49.3886091	16.6516073	23.7551832	48.351412	33.0501096
Flii	7.07055956	8.2575732	8.27810073	8.68499985	11.7097299
Nid1	12.4900987	10.9929847	16.0900109	10.9520329	18.1077294
Eif4g3	9.85650201	9.86852513	11.0077287	19.6505438	13.797616
2900060B14Rik	0.9830908	0.68615895	0.50573687	0.60170646	1.92573472
Fam213b	3.40596086	1.36980489	2.92309467	2.28801756	2.86250933
Ttll12	6.01954057	3.43627764	5.04542953	6.25694917	7.18019966
Elmsan1	1.83233606	1.2163441	1.55737024	2.72246241	2.95087472
Ghr	1.35581366	2.36061938	2.7702037	3.61248241	3.01144556
Sufu	1.07810763	1.80160354	0.84472421	1.64965516	2.28169397
Uba5	19.0313882	14.1974485	15.9886963	16.1763095	27.7386482
Socs4	2.90637056	2.63867775	4.82182772	3.14080157	5.65273119
Dnajc3	23.1821902	13.0527362	27.8306122	25.0207284	34.2442284
Fbxl18	2.23438145	1.48314311	1.51442378	2.70693517	2.12278535
Cables2	8.19297967	9.21509138	11.8309456	13.2140459	21.3643311
Ahctf1	6.04044412	3.10207985	4.47878646	6.1422299	6.5684244
Polr3e	5.40607063	2.2857042	4.37841037	5.8257805	4.49260919
Setd1a	1.46234273	0.98420599	1.43555597	1.917933	2.19391367
Fmn1	0.50856915	0.42938878	0.49118183	1.47603514	1.89309963
Bend7	1.42762104	0.42703872	0.83409019	2.13764963	2.2471924
Ncoa7	31.9461536	31.4633057	43.6767358	21.7473826	41.5377123
Arhgef17	1.43147611	1.50366713	1.54349905	3.58050566	4.7210134
Crabp1	0	0.20730612	1.54833287	0.80795942	1.26941144
Sgpl1	25.8758972	23.4724026	18.6588569	38.9741751	44.2090572
Taf1c	1.3623333	1.14836763	1.18729304	3.36595148	2.11919563
Mrps27	11.71552	6.62224143	9.86810854	14.5786833	21.7456545
Klc2	1.53740722	1.00598451	1.40384208	2.97976703	3.93556342
Chd7	1.73336165	1.16041373	1.79690564	3.13202135	4.42718947
Pard6b	12.7200206	10.3041937	11.3876945	18.3713427	21.5240991
Eif1a	128.203599	76.6379545	146.274701	176.885188	202.477151
Csrp2bp	9.99646001	15.5210993	16.9837793	21.6271845	16.1467263
Ccrn4l	35.1662907	51.5549852	48.2843747	13.1873509	64.6534672
4931440P22Rik	1.08744614	0.38561838	1.20185279	1.22380426	1.74285161
E430018J23Rik	3.01487238	1.37303165	1.47961352	2.76790535	4.46954506
Canx	160.877801	88.0948539	98.5391675	189.966894	194.201749
Rarres1	0.69324971	0	1.32667218	0.21215377	2.18510902
Sord	1.78978519	4.17071648	2.21866025	0.63606686	3.28488542
Hyal2	33.3288516	16.1450993	23.7424997	37.6250401	39.5958195
Phf7	1.61057096	0.59360185	0.33479577	1.3277575	1.02723794
Mfsd4	1.18249097	1.02345916	1.28160996	1.99853831	2.035165
Sowahb	2.70879326	3.09853547	2.94183246	5.37293012	7.23564522
2310047M10Rik	2.27987594	3.43826748	3.58556775	3.98688964	4.00195694
Zwint	12.5257132	7.71130734	10.6734292	13.5579576	17.946407
H13	7.41638579	4.38042671	5.04423662	10.1590651	16.5723819
B630005N14Rik	12.2990412	8.89063407	9.02639553	7.25782081	11.9380672
Alg11	15.1337362	9.36619405	13.4175333	20.5274753	19.8439303
Rest	8.95781764	7.96996298	4.65697588	9.17835079	13.0459848
Nckipsd	2.9538396	3.55042428	3.59785085	5.25685985	6.84627058
Hfe	3.7491041	1.36783515	1.82347216	2.12082309	2.36706164
Pla2g15	2.79547524	3.44391696	2.38772841	2.67710456	4.53824755
Atxn2l	7.01578155	9.58537107	10.4431263	11.2235733	24.5123645
Wwp1	22.463254	27.0091125	35.8015932	19.5357797	55.8849742
Fam63b	6.40738541	5.97524424	6.23203502	3.91179936	7.69537815
4930404N11Rik	3.84702053	5.3783261	4.08358871	4.65175368	7.06791244
Armcx1	2.06349362	2.40962878	1.86176821	1.89445997	5.29994297
Ppp2r2d	23.0607339	15.1643467	22.2068492	36.6275985	31.3340993
Tec	9.7473798	9.21528777	7.4117109	16.0494324	25.0465806
Myo18a	1.93742287	0.93940552	0.94248192	1.77871646	4.03521288
Prpsap2	16.2767259	11.0527362	10.9967295	12.7948834	20.7686228
Snord16a	4.29967184	0.56268804	1.32714246	3.28954905	4.0198029
Slc5a1	4.85812994	4.43955569	23.444665	6.37819557	14.6023199
6330416G13Rik	0.93416194	1.69271443	2.3954373	1.33593753	3.35426529
Yaf2	25.4319963	14.0713277	20.6319697	25.0017081	28.9918313
Ip6k1	13.9084769	18.2558599	25.0052952	32.2281863	23.4755761
Tor3a	16.5024686	8.19461806	13.4147788	19.3092906	28.1435405
Plcb3	6.12476893	4.30875515	4.69261124	5.35188851	10.1400606
Pcgf1	2.98451668	3.22225816	2.27998216	4.65806168	3.70704936
Bcar3	2.52942311	2.00163481	1.7749525	1.59652781	5.79582491
Smg7	16.5688623	11.3056812	13.7604927	22.9555129	27.8206922
Zfp503	10.6648123	5.21404661	4.3285698	12.8322865	8.19894762
Bicd2	4.84646787	3.12016764	4.39267229	5.79750001	6.12179714
Atxn1	1.58003245	1.12479254	0.63760894	2.66978524	2.91167584
Cobl	28.2878048	47.7684886	56.762743	48.594919	49.6343339
Txn1	99.5858817	42.7760694	56.88024	75.1932697	124.851024
Cdv3	40.74298	43.769144	38.907986	37.9578695	64.0589856
Tuba4a	57.0165527	35.1174345	116.652138	59.1115782	148.346979
Zfp259	10.580777	6.79752805	6.27534175	15.3939427	12.1052261
Vps36	11.2856565	5.49336866	6.19454397	9.56416423	15.1002306
Glg1	4.85296294	5.66293895	3.69799502	10.0092393	7.81836083
B3galtl	2.41152627	2.01752707	2.10011604	3.7426857	2.65715785
Phtf2	1.9136309	1.13223671	1.11061447	3.37765105	5.41041047
Tuba1c	115.085273	40.3006452	39.4625174	54.6610905	134.172359
Esd	81.6556755	66.3001614	127.301096	168.249659	127.995195
Rab30	1.15059546	1.06288763	0.75207035	0.34175803	3.28134404
4632428N05Rik	1.38605118	0.66568108	0.29905893	1.24780212	2.12437376
Fam214b	5.46695995	7.82353721	5.09687259	9.61000107	13.995125
Akr7a5	18.9145556	10.6261997	10.4794326	25.8660755	16.1401916
Tanc2	0.70936065	0.89809927	0.54992647	1.36308661	3.21238486
Klhl25	4.50458257	2.13866651	2.24725904	2.80514424	3.06409072
Bcl6	2.99755407	4.61387063	4.7425648	3.99131952	4.75646084
Sav1	10.2612131	5.54057033	7.58701518	10.5122149	17.5434204
Cib2	3.27830505	2.43500191	0.90249294	1.13882758	3.83410548
Nfatc1	1.36862957	0.6682157	0.93424902	2.27139534	2.37629678
Arv1	2.46293076	2.85690966	4.30210602	3.80288813	4.08191144
Zc3h3	0.46886775	0.65961603	0.75978707	1.57835284	1.08021666
Pacs1	5.61703636	6.92241063	5.17347152	10.8653608	10.5309208
Rnf121	13.0160429	9.77292317	15.9890342	23.4570492	22.5114365
Srsf2	288.363829	130.957879	195.307181	313.128769	330.075881
Qpctl	5.9932648	4.04760263	2.87547534	4.24790435	5.82297163
Abcb10	6.08803976	4.87549715	7.30621822	6.08986745	9.98789392
Rnf19b	12.5942007	12.9224908	16.2171358	10.5930755	30.4009501
Gm10560	0.46414014	0.42518657	2.36382492	1.0652988	1.05382733
Ano10	6.24341091	4.32334451	6.35032416	10.9900449	13.7685447
Rcor2	0.42092948	0.40678937	0.38977397	0.41617532	2.04982005
Mef2d	2.20112657	3.18597217	2.65212609	3.87120661	3.46399445
Ccdc23	63.1488708	50.3686179	46.5210831	62.2523662	87.8244897
Gm8909	1.53778487	0.2641358	0.35599105	0.70590765	1.07826695
Orm1	0.59437738	0.53338137	1.33664674	1.87093102	3.81043817
Sap30l	17.3869773	9.5202893	10.7983473	16.1559544	20.8152609
Gtf2f1	11.4499594	8.36540456	14.358987	14.3060958	22.209411
Sema6d	0.12960587	0.25050414	0.25848945	0.50341517	1.53392876
Mir128‐1	0	0.73149445	1.7252852	2.56584826	1.49306965
Git1	9.16536897	7.22476026	4.3675713	11.0062003	13.4008114
Atg2b	1.44886373	1.13765653	1.77498129	2.37784077	2.15769994
Arhgap10	0.45077205	0.40451306	0.6559264	1.65538598	1.34169853
Mll2	2.17076285	1.96791819	1.51670556	4.62906099	3.4052862
Mir7‐1	1.20762388	2.37058387	2.23648082	3.3260996	3.38705615
Clic1	273.39396	194.524508	238.570175	327.759996	498.001207
Vcl	19.6670175	13.1610247	9.31931163	12.6861217	20.6971034
Ddx11	2.0899168	0.48119996	0.81483349	2.22167424	1.30958398
Tbc1d10b	9.7979932	4.70391008	6.75028576	11.5962521	15.8044769
E330017L17Rik	0	0.30846152	1.89157775	0	1.3851369
Msrb3	1.14313911	1.2566396	1.80764105	3.22922291	2.31127826
Mir3473d	0.80508259	1.2643114	0	0	1.93546066
Prr13	418.331131	400.595883	474.235839	428.441682	951.348992
Derl1	29.2156663	26.29426	26.131625	52.0077182	49.266551
Tmem45a	3.16631999	3.51106491	2.19337592	0.37723739	5.36520024
Hnrnpd	10.7728562	5.00015663	7.3862981	8.46616894	9.73783971
Pgp	74.0582366	41.5293216	38.6768151	23.3793861	69.4233983
Gtf2i	25.1208553	26.1313741	21.1455946	16.8232504	30.0139866
H1f0	67.8326924	52.3231046	41.5492996	33.478958	73.9000987
1810055G02Rik	10.8108646	7.10117089	8.27806874	11.8339867	15.2996005
Krt80	2.16882169	2.02047509	0.6807777	2.66612944	3.18139982
Adamts15	0.57203236	0.06910204	0.19354161	0.40397971	2.15094717
Snrk	3.98403717	4.39651567	4.52419989	8.16799443	5.66257065
Jmjd6	13.8789867	11.0820532	14.8066293	14.6803029	35.1829152
Ubtd1	12.8117268	8.38320425	12.4170821	19.4469006	25.1876059
Gan	0.8360473	0.71439315	0.22769601	2.05435564	1.9507867
Gmppb	9.11286886	7.52756447	8.13458953	7.12815308	12.1807443
Myo6	16.9328269	23.2464251	34.1833643	30.4903736	35.7460002
Nrbp1	29.2464547	29.6485443	28.6863854	34.1355509	51.6971015
Scarna13	0.94853367	2.42058164	0	0.87083335	1.14016228
Tex2	7.23108172	10.526889	11.3145474	14.7554447	13.7569661
Arl4a	11.3054151	12.2127101	18.7007864	8.91677765	23.5275146
AI846148	3.96533214	3.31174104	3.68852433	4.5837293	6.28302526
Nsdhl	8.96734234	5.37902366	6.45231774	10.1762452	13.6887157
Pld1	1.94888418	1.56008617	1.10153082	1.87056795	1.54145751
Prmt5	12.0250257	7.1349099	9.16863983	18.0901532	12.2283591
Fgd6	1.54030594	1.46408014	2.68744699	3.12597128	3.41063585
Mob3a	8.86898687	6.92529314	7.30003561	14.2719453	22.0717096
Card6	0.84914802	1.29960943	1.10614732	2.56339435	1.89146321
Esam	1.93454311	2.89993755	5.53691853	4.1011131	6.08824517
Fzd8	3.09882207	0.55091632	1.29937798	2.55869108	2.31144674
Liph	0.99507445	0.52538275	0.34950529	0.33077234	1.59480736
Fert2	3.71565886	3.96375266	5.26522289	7.87352383	5.9851684
Mir697	0	0	1.66197198	1.64779246	0.95885207
Snapc1	15.5280541	12.0481439	9.15010081	15.5802331	22.5506214
Gm6402	1.10609798	1.51046052	1.51407772	3.35553411	2.50496567
Ubqln1	48.6168537	53.038309	48.1638218	46.5184717	75.8200697
Psen1	14.3237055	10.7389096	8.84900663	18.0223326	29.5428254
D430020J02Rik	0.51862685	0.12393924	2.54736093	0.765969	1.01190059
Ift46	6.29252743	4.97161946	3.438162	6.56648012	6.42060217
Wdr36	9.38416597	5.17669827	4.13223054	8.71661021	7.96149642
Il1f8	35.4124238	27.9356805	67.2642838	42.9698386	80.7013595
Trmt44	1.93015572	2.16509985	1.35642798	1.97772836	1.60197219
Atl3	4.86727045	3.83709733	4.82250566	4.69199003	12.4032432
Gabpb1	7.51020521	5.49787289	5.94862686	9.16504269	12.6199305
Adat3	2.03353249	3.08461515	1.88301858	2.67313745	3.7776461
Gdap2	5.65576827	4.86898605	5.34231468	3.71188545	6.58906041
Atp13a3	9.48420112	5.54150331	14.7554492	11.5414609	14.0063375
Calm1	244.173037	171.057445	169.27717	365.593296	332.300357
Psd4	2.18625566	1.3886367	1.35392336	0.90769919	2.46613148
BC021891	4.83473875	2.21298918	2.3763591	3.20806163	3.0480445
Zcchc14	3.09141271	4.82316446	4.36383132	7.00761318	5.03530592
Adamts2	1.237645	1.08130562	1.04919108	0.56012904	1.60641682
Pcdhb17	0.33902102	0.37581366	0.97871683	1.88580693	2.90851794
Hspb1	163.715283	98.3831683	133.743039	186.836186	248.844941
Tex264	33.0279269	42.2009959	36.5110922	63.5833991	95.4388803
Frat1	4.21605797	6.05287872	5.12833436	8.17655548	14.5737078
Nkrf	1.91681635	0.87797252	2.625434	2.27307235	2.27206251
Ccnl1	41.4375445	37.3590755	62.8623146	60.4559306	64.7553704
Ptpn2	7.55009209	6.36097268	9.75040702	13.2007051	21.9003683
Dpm2	60.7430604	59.7089779	70.9050138	96.7929751	100.387691
Gm20324	2.22046972	1.55999713	0.54108407	3.37974637	2.57541136
Creld2	28.2552115	16.930557	20.3644368	22.9120468	51.4911996
Nme2	232.00465	107.324368	173.377537	252.282432	212.903024
Pip5k1a	6.1224387	7.31205892	6.38389554	10.037342	10.0185857
Adam8	1.56903538	0.16876932	0.65679117	0.6511876	2.29078418
Gpatch2l	2.21310403	1.52869088	2.5269551	4.36916579	3.52027649
Arl5b	2.48581009	2.09434555	4.30121185	4.1812237	7.83716164
Clptm1	19.2272665	16.5847907	15.15837	27.8551783	31.233022
Il27	0.09249885	0.07263066	0	0.08492169	1.63072855
Lrrc33	0.9344749	1.19415082	1.13677825	2.72517742	1.5417339
Slc38a7	5.80189316	2.80829679	3.40378479	5.14420523	5.84353432
Ccnj	1.58886223	1.22075443	1.45544101	1.39596845	2.14944137
Pithd1	25.2705897	29.7639289	37.7700603	43.5204345	46.7870557
Il1rap	0.73772357	0.31980255	0.59061437	1.38280455	2.00137488
Mapk8ip3	1.62593705	1.22198004	2.09707418	2.19646969	2.75479992
Nr6a1	1.73677296	1.5666598	1.37847771	1.8127981	1.98823479
Alkbh5	17.6423326	15.663087	19.3231943	25.8160534	44.7115504
A4galt	56.6966919	101.655868	65.271307	58.5210021	77.6773775
Pecam1	0.51301178	1.9521274	0.89526902	1.97452569	1.96064214
Gpsm1	1.81793559	1.08825699	1.46670671	2.89186137	2.25011324
Ubc	79.5376795	98.0074977	89.474093	146.641212	135.76517
Zfp143	6.66618001	2.53218955	4.80995542	6.04062961	9.38309784
Actn1	57.6423199	57.6822209	64.3255907	65.4100304	97.9040789
Slc25a28	10.3658972	10.5411448	14.8307089	10.6478524	17.0900545
Gpld1	1.51166827	1.11003773	0.79061685	1.2207835	3.19668808
Pold4	24.4990247	35.1085288	34.9214354	22.0331329	44.1297749
Ap3s1	140.344022	86.93359	80.2683399	144.189454	242.808054
Ispd	1.21211319	1.10403995	1.54890846	3.07138714	1.84552289
Shb	4.11137839	3.6947934	3.58700877	8.16009566	12.7025186
Usp9x	2.07108206	1.91877472	2.01418567	3.47085843	4.03939192
Ctnnd1	33.064411	24.0544769	22.5988727	26.5067357	44.6311925
Plcxd1	0.45962567	0.31759274	0	0.3038219	2.04786689
Acer3	14.4147081	4.5299299	6.10099621	14.6207404	9.78700164
Tpm3	203.108983	172.925717	158.292232	144.029491	240.391867
Lss	0.63067398	1.7002176	0.97332337	1.91073807	4.24528507
Trmt6	15.0310632	7.4446421	7.3875244	12.8019451	15.0101151
2310008H04Rik	2.13972732	1.72723314	2.31466506	3.89217911	5.88116518
B3gnt7	2.2114176	2.82167994	5.04953414	5.88315883	4.22892559
Eef1e1	68.7892317	32.8714879	71.9505368	99.1107253	91.5385338
Bola2	40.8298709	20.6641756	36.5535203	52.5859308	37.2159794
Lats2	4.27386658	5.54086632	6.87131953	5.87487164	8.47748494
Ing5	6.10730896	8.94E+00	9.23472401	12.2430956	9.12029323
Tmem144	1.87782352	3.01597843	1.52806674	0.80975723	2.41679249
Dnajc21	14.9926534	12.5455039	17.0595663	14.8726714	24.5632866
Scamp2	34.3043559	43.0713428	37.1024719	46.9602089	60.4240569
Slc30a9	19.5386534	19.2899226	18.8183043	22.694706	30.9469435
Slc26a1	0	0.0158774	0.22468831	0.85395673	1.97687051
Hic1	1.05976254	1.2652965	2.05674583	2.15915103	5.46771944
Stk38l	15.0705811	14.0737821	14.2559304	25.6881221	26.8700795
Tpr	6.55571819	5.09333288	4.17496167	9.1985377	8.85261759
Dnajb2	8.06238809	12.6932443	23.1510328	12.9347164	17.2288024
Gata6	0.12250161	0.224441	0	0.37488912	1.75064052
3110043O21Rik	3.50732039	6.06833889	6.02339252	8.27468681	15.915805
Nlgn2	1.06904409	0.56302552	1.08649508	2.22626579	2.24617135
Tmem5	28.4413827	19.8427159	28.7025858	45.7495586	39.7050343
Rnf152	0.87927592	1.05062809	0.96995457	0.58262315	1.97290362
Nat8	0	0	0	0.19271393	1.51389573
Clasp2	2.22052325	1.9624916	4.57336251	4.00411807	5.64945272
Elk4	1.54546908	0.83690457	1.21724017	0.99483992	1.92175268
Eya2	8.15737233	4.41867206	6.76539501	3.95079851	9.51168735
Snora31	9.89820288	4.57184032	16.1745488	17.1056551	13.997528
Slc7a7	4.11151115	3.39184578	8.79509914	2.07848044	6.1724667
Eprs	17.4121934	10.1459034	21.7385935	25.6020516	19.9763484
Trmu	1.80190195	3.23397547	3.65488049	4.0175782	6.39466014
Capn2	35.2163598	23.2952848	16.8547093	26.0720259	31.8630907
Dhx35	2.34427768	1.68067653	1.22695337	2.54526159	1.92759539
Tmem120a	12.0932476	9.61588949	8.26867907	7.77651456	13.3301445
Sptlc2	16.5374343	11.6485717	11.9193585	14.8797327	27.9105388
Cradd	18.1407064	16.6647736	11.564639	9.94201407	15.9939431
Kif1b	3.67142383	3.25179801	3.72907459	3.63274856	6.34169996
1500015A07Rik	3.53346601	3.18655308	2.52683938	4.59301522	7.17698715
Dcun1d3	2.85476066	1.69656481	1.36838071	2.87786128	4.97903484
Pparg	0.82171988	0.1032351	1.70441482	0.78458398	0.86920133
Cfl1	1040.24762	758.90078	836.362602	1106.78212	1612.65394
Pde4b	0.87539722	0.23760841	0.34025346	0.65485687	2.57216755
Ercc4	1.57605456	2.21532707	1.35129752	2.07217543	1.61379824
Naprt1	3.07081501	2.95307019	5.55925232	4.56150802	4.94924939
Wdr91	1.13755697	1.20390061	1.7403332	1.36222509	1.12956919
Ppp1r3e	0.7746529	0.02764828	0.06521056	0.93748596	2.00338989
Fam162a	77.9459407	56.68794	79.7842519	45.9158883	114.225706
Pik3ap1	6.43388753	12.6298181	11.1764173	8.65421861	10.711276
Chd1	4.66974337	2.81955173	4.88082704	5.91287926	6.07184171
Jph1	0.70543122	1.18238233	0.79139877	2.05502721	2.09812471
Tesk1	5.76456924	5.30434512	6.20530755	8.91431445	13.3097673
Ttpal	6.30057668	4.24535249	4.55257105	6.98900079	7.74098679
Sec61a2	6.96435519	7.19760897	6.49986088	9.57815596	10.5441348
Mcl1	54.6080107	50.0445415	67.3638027	105.145572	129.512124
Rab3il1	0.4216273	1.21390243	0.65070024	1.57416266	2.34257479
Camsap1	2.12418558	2.58750081	2.87939288	4.5009864	3.70863411
Patl1	3.33705874	3.62714495	4.10577822	5.56004709	4.38264225
Wdr3	6.2776714	2.91274774	6.67616925	10.1471265	6.40260322
Cactin	8.8098024	5.78965505	7.67004544	10.3079783	15.2321606
Pdgfb	1.90625605	1.2879474	1.29308425	1.77045274	3.92552472
Scpep1	18.9598114	10.7890141	9.43333374	24.0171975	15.0422808
Ptdss2	10.0579724	8.36907785	7.75663443	17.1725971	19.3920326
2610305D13Rik	0.22917033	2.35929097	2.40502467	0.86496772	3.5708909
Strip1	11.0603547	8.25676342	5.79366897	11.747895	14.8313434
Tbc1d13	3.00687906	3.24640188	2.81912596	5.79719031	6.08415125
Creb3l1	3.30222118	6.51613066	1.86127201	3.16352934	4.09591542
Brox	12.9253493	13.9123641	11.9337219	26.2796515	22.0461065
Nuak1	1.35153761	1.20409409	0.67388581	0.36986122	1.55168558
Gnal	4.97012703	5.32492401	5.52480652	8.78298175	8.13800723
Ly6g5b	1.91799087	0.09716245	0.22916502	0.8520369	1.33866302
Adam15	3.09804614	1.33621937	1.57578742	2.60390534	3.02460245
Gpr180	9.49728812	5.0012151	6.90533858	13.1309834	16.7107552
Agfg1	14.7178439	8.40624002	10.5871024	12.4434507	16.9582632
Rpph1	2.20716488	0.78776325	1.11479967	2.02636222	1.92950539
5031425E22Rik	3.25290046	4.64582296	4.53005953	3.88001407	4.90554894
Rrp1b	2.81295253	1.45232698	1.78407471	2.88912729	3.31433818
Zfp59	3.88246624	2.37408104	4.19958779	2.93355676	8.85182625
Snora21	0.50946632	0	0.94351534	0.93546551	1.63304493
Prkcsh	11.1464592	9.80459228	9.2620574	18.2460321	17.1310097
Mest	2.93534793	7.35709039	6.4146812	4.48430569	5.08084558
Mfn2	4.27593212	5.6738032	5.42181813	6.66131805	7.13958139
Rbm19	1.63359243	1.515926	1.19180886	2.39357977	1.63965645
Sars2	1.80953903	1.82299141	1.39106765	1.69265383	1.55951516
Ephb2	4.7086716	2.58273574	2.51498774	2.81931841	4.69201356
Gclm	32.7317361	27.6040304	33.1359675	40.2791946	73.493327
Degs1	51.4212874	52.6092268	28.7016897	56.4657248	120.36152
Sec24d	10.8545255	11.2047426	11.7732711	17.2764012	17.9791599
Med8	32.416376	25.1289404	28.3901923	44.4042203	56.8399705
Rngtt	3.42310338	2.88511416	3.63502179	3.94999355	4.64314821
Etnk2	1.08686149	0.22458163	0.29133105	0.47265626	1.65023488
2310022B05Rik	6.68483905	2.2761046	2.31898782	3.89235121	8.40669999
Dok2	1.25334057	2.08754968	1.30124889	1.46449115	1.82611896
Fen1	29.3188469	8.24988791	10.4355685	18.9955913	24.7176269
D930048N14Rik	3.95568137	3.73320294	2.65912286	5.20303244	6.14165862
Idh3a	48.9630198	18.6372858	23.1090614	40.8429535	39.1496029
Plekhg3	4.79052573	2.85216403	4.14347001	5.32847197	7.67778298
Spata5	5.22141707	1.82999299	4.81419788	5.08173155	3.91481836
9630033F20Rik	1.83870901	1.0232512	1.32241954	2.13059761	3.24731409
Tsr1	3.35209276	1.78497612	2.80072147	5.76589437	3.95211346
Ociad1	22.0476114	12.156758	18.355164	32.992139	26.343718
Pabpc1	120.849748	65.9667921	77.2799291	119.081442	131.347773
Ehbp1l1	0.71872554	1.16048949	0.64678109	1.44981181	2.86353237
Spata5l1	1.82651969	1.20162222	0.45711569	2.17543532	1.54280096
Zbtb8b	1.26932729	2.36712316	2.27729251	2.82232904	2.4634741
Cystm1	89.5427737	98.7087217	118.841704	68.5232418	160.988039
Gabpa	9.18142921	6.49043612	8.52607849	9.44996529	13.8451816
Taf15	1.97321852	2.29908456	1.38511184	1.51938957	2.06581379
Bcl2l13	6.01982999	4.38706754	5.62652508	10.707997	9.27111616
Sigmar1	18.0346596	5.02069101	7.21128007	14.8263346	17.6381169
Alg9	10.9155636	6.2669431	6.76016178	15.1290837	18.6418721
Uvrag	5.40585559	6.12131918	13.0500412	11.8938535	19.5015394
Snora17	0.9880559	7.75827447	1.37238596	6.34982651	4.35478648
Rad54l2	2.01382871	1.46047895	0.83526299	1.1812183	2.00602216
Smg5	7.69870407	5.44631915	6.69435488	8.30664766	9.54194725
Tmem245	2.24150405	2.87399342	2.25951057	3.62084171	3.02403447
Trmt61a	3.53799549	2.08354153	5.36093552	5.38901957	5.26225739
Ap2b1	5.22031088	10.6688107	11.8559532	13.2822613	13.7575506
Abca1	9.12073825	11.0494162	45.0062337	23.5336135	19.7977252
Rab8b	9.47086209	8.02934868	4.96959124	5.34304192	8.72240541
Foxj2	2.82284792	3.22773933	4.30030692	4.41887559	9.38194573
Snord34	0.9880559	1.5516549	0.91492397	0	1.58355872
Atp6ap1	78.3493575	60.070097	79.0604653	124.197683	131.753677
Grhpr	4.6807738	5.0954018	4.08805343	6.34834671	10.4003383
E430025E21Rik	5.48570211	3.73393509	2.6901224	8.62732952	6.97112658
Mtfr1	19.4014772	20.4719308	23.0316388	36.2039341	45.6641308
Ccdc74a	1.73489628	0.88145654	0.28349757	2.15493777	1.43115049
Dpep2	0.40618833	0.20296279	0.61547547	1.18654742	2.337677
Dlgap4	6.0268332	4.6082378	3.27111447	6.02013666	8.53773299
Malat1	253.37478	327.74765	432.294893	481.796627	432.886483
Ddx21	14.299	7.02808394	14.4883216	16.4474679	14.0366805
Tmem184a	9.01731018	12.0294087	11.701396	20.7395837	24.9785552
Ly6d	2.17057722	0.14820438	0.17477564	0.60649573	2.26877443
Nfkb2	3.36755383	2.39535546	2.05441008	2.36423848	3.68278419
Arih2	10.2563376	7.20493132	10.174477	11.9037569	16.317961
Tfe3	6.44398474	5.7363823	5.43514702	8.63522472	13.43475
Eml3	5.65988249	4.3153572	4.89915892	10.3736654	6.49109682
Furin	12.9563575	8.1829602	9.01862542	14.4945054	23.5211927
Lnx1	2.97115318	1.35419371	1.59698434	2.3672005	1.902012
Hsf2bp	0.15921933	0.03571999	0.04212416	0.08352953	1.5128592
Mansc1	9.74332903	13.059762	16.0619986	14.4634937	17.8150356
Samhd1	5.51290842	4.34153814	3.80982555	3.40406802	8.40549808
Bmp4	0.09811187	0.38519016	0.6662335	0.6305244	1.96555057
Mir200c	0	0	0	0	1.51470834
Tes	104.546996	54.0353333	78.0158142	50.3327947	82.280855
Gba	9.39717167	6.66972889	6.16236483	9.33354718	12.3005969
Capzb	139.388759	125.66069	115.195034	178.437817	262.545772
Asb6	14.1348575	11.4865054	10.3451791	18.9554621	19.785116
Qtrtd1	7.16E+00	3.53698167	7.63635798	11.1553351	8.31157971
Tshz3	1.34282368	1.50481365	3.81480494	2.04672822	1.77604247
Ahcyl2	9.22772654	9.62285364	14.420629	12.4075559	14.5272766
Pepd	17.2091291	13.7061453	17.7928765	32.7619913	20.9819394
Cd9	710.544819	847.968986	980.445421	557.763464	1482.89248
Nup50	12.9214992	8.10820549	13.5417995	15.632458	23.5263007
Smcr8	2.03458534	1.90186746	3.59603521	3.92856137	3.82371495
6030419C18Rik	1.18899294	0	0.08469142	0.36386503	1.71015458
Zfp37	2.95174454	1.21609164	2.81762839	2.87722949	4.01531025
Cdh1	135.580614	123.190428	132.83875	108.926936	268.309319
Bcl9l	3.21897041	3.8249691	1.64336006	2.96652126	2.55010019
Exosc10	3.45801865	2.97210572	2.03374716	5.01953834	3.52002805
Heatr5b	1.10425263	0.62046912	1.66042284	1.7857698	1.3232814
Exd2	6.88814416	6.48725292	3.32544439	9.15252926	6.44765256
Cep95	2.67829455	3.07941191	3.91937727	4.25916384	5.28898159
S100a9	1.60356614	0.83941986	3.71219152	0.36805201	3.74797197
Dgkq	0.8752284	1.21674324	0.67758726	1.71244732	1.56369891
Pgm2	13.9140623	30.1655274	36.5311031	16.8784592	26.9123565
Ptgfrn	7.30334857	2.88501614	11.2921795	9.48852402	10.0522862
Rnf24	2.89439125	6.07278412	9.76576529	12.1998823	11.7379689
Ripk2	3.67051593	4.01340722	3.87531184	5.41694075	9.40139069
Polr1b	2.70763894	1.38321612	2.49212657	4.56735538	3.99969383
Dennd1c	4.31985409	2.53890379	3.20967378	3.49101925	6.61250402
Prrg3	0.03316973	0.17363381	0.20476427	0.87297426	1.84292083
Snora70	3.53861881	4.3662847	8.42581145	7.88981765	7.69683191
Nol12	6.80583614	3.39137417	5.02955696	7.14952869	9.78024298
Tmem33	22.9617134	17.3231186	20.5759866	21.5375748	42.5440016
Cpt2	24.1422	42.3581978	56.4278385	29.3489367	48.8996194
Txndc17	103.905763	110.482979	106.613319	130.835785	165.879418
Pdia5	1.40589049	0.82085889	0.26704249	1.05905659	2.10878549
Fbln2	0.55350552	0.51467669	0.28324428	0.3610642	2.21776037
Rasl11a	3.3011246	0.59117225	0.9116738	2.02047258	3.43432539
Ppp1r9b	5.34645492	5.55800633	6.8752416	7.5079209	8.50842808
Pnrc1	105.287237	117.544508	137.489546	149.938842	255.803551
Pef1	37.4620344	39.484665	34.5724462	40.4150052	64.4205164
Npepl1	13.0298989	6.91030284	6.91101368	16.4734718	17.444066
Pi16	0.78428313	1.66500519	0.80692626	0.08000418	1.86218041
Plekhm3	1.36333712	1.44726886	0.87453139	2.64316534	3.14325863
Snora41	4.61675678	7.25021048	5.34380372	12.7157082	9.71155922
St7	2.99991247	0.86903762	0.97090637	2.67395234	2.19392548
Mtmr7	0.73803131	0.46063348	0.28037136	0.57333232	1.60745456
Tac2	0	0	0.13661761	0.13545202	1.53698346
Meis2	1.0771629	1.05967394	1.53628016	2.06878722	1.44856387
Lyrm1	8.63697142	5.05625474	7.52446093	9.00862086	11.5900117
Psmd2	30.224219	21.53512	18.1372514	38.4358971	47.8070827
Map3k9	0.75138784	0.55123552	0.25901044	1.27896782	3.32707152
Rabl6	11.8848563	7.9038497	9.87595903	18.5776631	16.0995973
Nat6	9.10613683	17.2475994	13.932671	16.4507088	17.0006679
Dus2l	2.27890313	2.56023058	2.59720353	2.31754037	3.56811537
Arhgef16	6.64837615	4.57958064	3.95602753	7.80048091	9.98218998
Wipi2	16.5738735	16.9809208	14.5905927	25.5491394	27.4715653
Rnf25	6.58748593	10.2756373	13.3051655	11.8521895	12.1874973
Hyou1	7.136207	4.2054329	5.95402463	10.5366513	16.8846234
Map2k1	38.7937156	37.5400378	35.5428989	52.9723512	94.9697381
Mdn1	0.70444166	0.33929221	0.66911362	1.80352792	1.29486941
Mpzl3	1.00299523	0.4805438	0.83430763	0.59307928	2.64284226
Scarna17	1.05180144	0.82588083	1.94790265	1.93128364	5.05717139
Armcx3	1.11736425	0.83198039	1.05250043	1.11426793	2.5009468
Lzic	14.7157499	14.5228611	19.6072789	18.2944837	42.3176437
5830418K08Rik	2.03482412	1.77837207	1.13052876	1.98997412	1.69441427
Phldb2	1.19443743	0.58617467	1.61650533	1.46563952	1.44494852
Surf4	66.0772307	68.4099333	59.5413218	101.525618	136.055515
Dis3	2.7857761	0.79792248	2.44979374	2.47714887	2.24642397
Nudt18	4.79084802	2.78345843	2.37249929	3.93117046	4.443851
2810408I11Rik	6.12788667	2.80081325	2.20197691	3.10684759	9.16154237
Abp1	4.55914769	0.87270468	1.55425693	3.31105984	7.05248203
Aloxe3	0	0	0	0.04717872	1.52366052
Actr2	179.603195	126.915982	135.022729	201.475152	249.422799
Lmcd1	1.50952985	0.32595528	0.59406522	0.31182184	1.93546066
Mtm1	6.41840706	6.93133982	7.95565509	7.54752231	17.1986504
Fam134b	19.489447	26.0614027	27.5756882	36.6626436	50.742503
Arhgap27	2.88619102	1.27419936	0.69765799	0.74491388	4.23558329
Fchsd2	3.20810171	4.6317479	5.10623403	2.57883941	7.27410264
Uhmk1	1.03101485	1.9227028	1.46188939	1.8339561	2.78844035
BC021614	2.99490722	4.32394497	6.26214629	5.05567139	10.4514875
Haus6	3.72386103	1.37192329	2.3042713	2.91652577	2.85683421
Rabif	30.4254641	33.3522636	35.4172253	46.8506342	48.3361292
1700066B19Rik	1.38027937	0.34763514	0.24115408	1.50630868	1.37739253
Arhgap31	1.76498875	1.12727065	0.91492397	1.39556627	1.83395048
Zfp318	1.75527739	1.80280596	1.85029685	3.83448685	2.48037586
Sgsm3	1.74071911	1.64018776	2.3372232	3.97533826	5.09148209
Tmem63b	6.68837841	4.16027582	4.45845249	10.5054193	10.7840496
St3gal4	5.1745106	9.23651445	17.0530284	13.5732404	16.9984765
Mphosph10	15.4292871	7.90625246	15.0276885	18.488401	24.0403199
Krt7	101.39472	41.71271	65.9056771	55.8684069	103.460501
Lpcat1	1.99088647	0.8294828	0.82770692	2.35002925	1.79075332
Cdk13	4.36525079	4.59185543	4.64762254	5.774759	7.21021277
Cirh1a	6.2631058	4.89506068	6.55132753	11.4468701	12.6403051
Polr3a	1.1975668	1.08248058	1.83102017	2.54411462	1.75195766
Mir181b‐2	0	1.19080492	3.51075477	2.08848114	4.25351237
Arpc5	340.393332	268.925432	272.862449	368.749952	612.54422
Asap2	1.64206227	0.47787364	0.57418366	0.93826581	1.61952968
Sap30	18.7972833	3.6358304	5.51274561	11.0326414	18.8179784
Pan3	12.0193542	13.0666911	17.3013151	24.6827063	25.801263
Rinl	2.72282629	3.83054679	2.43725049	2.72892932	3.7092962
Matk	1.0530146	1.12213105	0.34824096	0.41432383	2.23013287
Angel1	1.86319113	0.39899697	0.53326997	1.18184526	1.14016228
Obfc1	7.22259394	5.91661379	12.8293971	5.89771654	12.9391419
C920025E04Rik	6.27327347	11.2816987	7.87019161	14.4913696	20.4732398
Zfand3	12.2349788	15.6529191	21.5973799	19.8803401	28.8943494
Gpa33	0.73942605	0.87090268	0.10533795	0.44386676	1.66366897
Sbf1	1.56058606	1.42144263	0.80924354	2.34970789	2.70958316
Smn1	13.1909685	6.74704639	9.63407121	10.1062257	18.3868762
Ist1	49.8137116	47.5752497	44.3218549	74.8755863	87.4006761
Tbc1d15	16.7213885	13.1974839	23.7928654	31.2894524	25.693125
Mgrn1	13.4463217	18.3484487	14.1197802	22.1989119	25.1844985
Tmem212	0.63665527	0	0	0.25050123	1.67631948
Csrnp1	2.70775182	2.51593593	3.96994692	1.82302483	5.71396205
Tomm6	50.5115371	37.068681	45.1538148	72.4145061	82.0446961
Mospd4	0.36926212	0	0.47870314	0	1.65708749
Ppp1r9a	2.93715581	1.70020549	1.39782979	4.41482498	2.99958897
Rangap1	30.346679	11.103998	16.4942323	18.946137	27.1905788
Snx13	6.14829132	5.46094797	4.72333541	10.4526927	9.59367994
Snip1	18.5958495	9.6683116	16.1026619	20.5746084	25.8028123
Etl4	8.69381062	5.19623965	3.31925906	7.36366435	9.48835456
Hbp1	40.8649668	58.8732267	52.9792767	83.2670208	75.5267873
Gga3	4.65924324	5.870238	6.49618389	8.76659014	9.24194293
Gpr39	1.23681524	2.17081655	2.87440599	3.65141168	3.67298465
Exosc2	18.3734538	6.97670724	9.79115307	17.5327692	11.3084837
Snx15	11.2565279	13.1915839	13.12717	13.4425362	21.1607016
Lamb2	4.672886	3.67828291	2.55540998	8.17567583	6.09546215
Fam83g	2.00294993	1.30525754	1.75376358	2.55190926	4.79335879
Phlpp1	1.29269568	1.59385259	0.88044944	2.19705662	1.58381815
Cln3	9.95427769	9.94075062	13.8607325	18.4600427	16.8687884
Adrbk1	8.93535407	7.48276064	7.32514238	11.3267176	16.3571364
Efhb	0.88223255	1.05834409	1.54309612	0.83246236	1.45323201
Pgbd1	1.2750889	0.37187707	0.86023298	0.96995754	2.45230434
Polr2a	3.37360008	4.35314252	2.4158158	4.19160819	3.61359416
Ddx39	22.7199256	10.4322837	17.6896331	27.6794039	37.3582406
P2rx4	9.84883018	10.8164282	12.1194144	22.4999874	18.118359
Smtn	1.86759194	1.17920275	0.74879517	2.08580914	5.2766589
Osbp	10.7762068	9.22062549	7.1482549	17.2156653	15.4026719
1700025G04Rik	13.2977292	11.4388813	8.59900964	9.22537406	16.8173157
Psph	8.23101351	5.76343383	4.20376908	4.40161782	7.0719239
Trafd1	7.79077707	7.33259924	7.38416514	7.22483421	12.2760835
Rp2h	19.0674433	8.2095085	8.22996586	12.7614693	15.1140767
Blcap	23.984107	26.7100978	45.6371114	34.3675637	52.8352844
Rab6a	86.7983301	75.927802	90.5592848	97.1261156	146.147683
Tom1	0.52545467	0.86861088	0.66582253	0.86326249	2.06104398
Tnks2	29.2602366	30.2202807	34.9209864	60.6180497	38.824188
Kdm4b	2.91401476	5.48700176	4.44045746	5.67529272	6.08725467
Higd1a	30.0468169	16.8950614	19.5372659	22.2793623	55.2427798
B4galt5	7.33709066	4.71365002	8.93421951	5.35467224	16.457861
Zc3h12a	0.67420285	0.52938814	0.68888393	0.59763073	2.85040569
Hace1	3.84206255	1.86690526	2.05803506	4.77692931	3.32735601
Cnih4	20.0144812	10.5292224	15.2625444	18.5215301	24.8014951
Mtf1	2.70896417	2.94572461	2.73002474	6.01757713	5.84138309
Lbh	1.67917975	1.08484347	0.8069701	0.93668515	1.58407487
Hist1h4c	1.25407095	0.49235203	0	1.53512289	1.00495073
Sympk	2.19956924	1.66498858	0.93779152	2.30994833	2.63759938
Snord88a	0	2.84470064	0	1.6630498	1.45159549
Itgb4	2.39958721	3.44462859	4.99211231	7.88041364	10.1058718
Tulp4	0.90580974	1.34462074	0.85101009	1.97886571	1.63188592
Gm16062	0.60945504	1.0368535	2.35144011	2.2381231	1.70935544
Tmc6	3.23569452	1.38434859	1.34444935	2.41840448	3.95587474
Tex30	41.7561016	16.1507036	28.4978927	36.9630895	31.1066013
Ndufaf2	24.6461827	6.77708094	15.4514513	21.7468218	16.1383264
Tmem43	18.099104	9.43242844	20.742771	27.9392366	25.2669295
Bzw2	76.7831919	102.628163	268.779332	97.3084125	121.943331
Ell	8.01141988	7.28649091	9.64985288	12.4979405	17.6167699
Itsn2	1.47922269	2.05938753	2.52575536	4.12230877	6.06139199
Map3k8	1.84684083	1.38887658	3.10716501	2.62691552	2.06361641
Unc13b	4.04065546	3.46919055	3.76992769	6.28581301	5.66646915
Tmem47	5.01628381	5.11795235	14.1716837	4.5907019	16.2840423
Rnps1	22.5967665	14.3596699	13.0069628	24.4213931	28.0319554
Rhoj	7.24574328	7.96950485	9.8592952	11.6286536	14.693249
Usp15	13.6305615	9.52421701	17.6928937	15.7169845	22.2033157
Htt	0.97773092	1.07403282	1.96162027	1.83154599	1.53139697
Imp4	19.5722056	10.2910113	14.4988396	24.916908	19.5181181
Ssh3	2.15220098	3.49250376	5.13726287	4.30307275	5.46144838
Chst11	6.29784934	6.40173618	6.37877234	4.04628243	7.79269274
Pcnx	1.50418264	0.79723796	1.50228722	1.35630555	1.00304663
Gnrh1	0.49970643	1.66758313	0.92544034	1.03223781	1.30143044
Lnpep	1.66144432	1.58130435	2.57577322	3.67469487	4.54894893
Kcnk6	0.98878082	0.08765769	0.20674731	0.20498339	1.86588063
Cox10	5.71523195	3.41831539	4.2792507	5.08309093	5.81433319
Myh10	10.0125876	4.42768901	4.85686737	10.1000948	7.94973741
Rhof	1.88666577	1.81593583	5.35377816	5.08460209	9.07128643
Gng2	1.65330211	2.39561565	2.88825189	2.4724065	7.08876377
Slc41a1	3.48373969	2.21912283	4.88417469	6.6536264	10.3147056
Smad3	11.5433659	12.3031906	17.2969163	25.1241409	18.9831046
Tmco3	5.00632428	2.8465833	2.03814301	6.60377154	4.93407971
Pa2g4	23.1404967	11.7063174	20.1117745	25.6268315	24.7535231
Klf16	5.09005269	5.90822441	5.94286588	6.54684763	8.84750736
Slc35c1	14.7898669	10.8420336	8.75897711	22.299382	22.3386896
Cep57l1	2.24020048	0.53628625	1.99849752	1.40456992	1.11651836
Plcxd2	0.55994772	0.15353707	0.0329208	0.35087925	1.666654
Rasa2	6.25913216	4.28576938	4.37603046	8.68789588	10.3891344
Prl6a1	0.3005147	0.17697446	0.62611157	0.8966674	2.58879012
Hic2	1.0801747	1.05818017	1.60988515	1.07669291	2.61328408
4933426M11Rik	6.21860731	10.2510318	4.66150602	9.97632875	9.28562565
Tmem184b	17.1192482	14.1238769	13.2479932	18.7493823	34.3038694
Nrd1	5.10079401	3.04393238	3.83673532	5.96536563	8.28824343
Fhdc1	2.21202174	1.21413866	0.73579593	1.34073634	2.86542512
Arf6	68.0257024	45.1522841	49.6707344	50.8195212	85.9845645
Uchl4	14.6473761	6.03703251	8.67839243	13.9627486	20.2823618
Rtn4	50.0568603	34.2105728	35.7840282	29.1544541	62.2798411
Crk	51.9458511	33.4712289	47.9341262	49.0806538	75.078541
Gskip	31.927044	24.4260333	32.3382603	22.3920761	39.9527431
Ube2z	48.834924	38.4356709	39.0815737	55.0488734	98.3739032
Znhit1	19.9679204	10.6676274	13.1653304	16.1277155	26.8882746
Cdk20	2.5699188	1.37330376	2.04918932	4.09618177	2.74587522
Hbs1l	7.33015108	5.08592708	6.67653286	9.29921163	13.2325701
Pyroxd1	0.84729805	0.99795531	1.45708541	1.722472	1.74595616
Zbp1	11.3411634	4.80783355	23.7126965	6.14759204	14.2849888
Neto2	1.54770925	0.34462898	1.0802131	1.14522452	1.70304083
Cxcl3	0	1.51642482	1.60947139	0.29550737	1.80553832
Brap	9.02985141	8.99391345	10.0533187	13.5965074	27.2408788
Adipor2	76.335359	122.785118	110.454387	126.719107	144.258483
Zfp296	0.93768443	2.14189225	0.39467309	0.31304467	1.60529384
Leng8	9.2300016	15.2689056	10.7042872	17.3474311	18.3013877
Ifrd1	22.2639028	12.3218146	17.4238605	11.6706534	21.5533154
Lgals3	94.0606731	48.3275388	61.4586837	60.4659358	125.179979
Acap2	5.94568308	3.42257838	5.2266071	8.34470391	7.8309698
Sorbs1	1.31556493	0.37700347	0.96032662	1.56044078	3.79368529
Rras2	43.6560014	27.9093267	39.8408167	60.3698042	59.9065484
Dok1	7.40631377	4.79422679	2.17452844	9.61896948	10.9726143
Ldlrad4	1.41252757	1.0475071	0.72665442	2.54560691	1.86558843
Hs3st1	3.87012994	46.615949	90.9537593	44.555917	53.3118905
Sp140	2.37526022	1.25044374	1.49962704	2.42849325	2.70371677
Epn1	10.4237009	9.48233547	7.15673861	15.9652781	15.768911
Pdia4	87.0277727	44.6827827	45.4651941	79.2231075	94.9311857
Shc1	25.7474648	19.7481831	19.1579609	31.1845352	39.3795612
Smcr7l	11.1677903	6.97695552	7.28988969	12.3247489	16.0594524
Pdp1	2.65519909	1.35814931	2.19173039	3.34312477	4.12174765
Cttnbp2nl	15.6377632	11.5312785	12.6325382	14.0334794	25.8674317
Snord57	3.10531855	0.81277161	1.91698356	0.95031417	2.48844941
Kdm5b	8.3533069	7.26617821	10.0833335	13.3139015	19.9490695
Entpd7	2.86715858	2.37826378	4.11216737	2.22656254	5.71808657
Mtbp	1.75812239	0.87430925	0.75973017	1.60065274	1.66716606
Rassf8	0.07525873	0.05909361	0	0.10364073	1.598179
Gale	5.98233456	3.66461111	7.62178693	8.53056904	13.5998537
Cltc	78.0544865	67.5810864	76.1790358	119.408275	125.08954
Rgl1	0.28520677	0.28981257	1.19620366	1.63267251	0.83353773
Mir194‐1	0	0	0	0	1.55992351
Strn3	40.4832415	39.3134349	50.4948385	67.7776665	61.8454978
Acvr1	4.13257826	6.3035392	11.9012245	12.3442872	11.6779053
Sms	11.2580441	12.0392246	10.5678189	15.0339308	17.6409473
Sin3b	8.96490079	10.3505018	10.1835571	16.6838827	16.4345326
Tax1bp3	73.3113772	44.7724162	40.7396209	64.728668	77.2613493
Zdhhc15	4.84233497	3.38389227	2.03914427	1.69565862	5.36046701
Rpl34	4.80622167	3.25925501	3.23671596	7.52133075	5.16447039
Fam43a	18.8106565	27.5755567	18.6555229	41.4902531	66.991486
Tmem171	9.20303497	6.73861554	1.7252852	5.49454375	11.5826009
Coq4	9.87856998	5.05088267	6.65541574	13.8601433	12.781638
Ak3	137.084406	110.673435	85.0569994	179.065305	207.548079
Srgap1	1.95109773	0.89694819	0.29339334	1.04108066	2.79294322
Tmbim1	32.4505116	41.6910653	41.9478178	40.9935654	70.7252026
Sox17	562.070594	625.41607	555.00165	271.824161	654.753973
Snord70	0	0.96612475	0	0	1.97197878
Bri3	8.33704612	9.28139631	9.62352603	17.5100795	18.8987056
Rab22a	22.4090458	22.6115702	19.3586218	25.5285549	43.5524271
Flnb	11.1203697	11.9454835	8.19919622	8.03678996	14.6754287
Pdgfa	35.0400729	13.832287	21.7694385	18.4667141	42.8634189
Pum2	21.9940712	21.7347723	27.0856358	19.6461749	35.4218827
Cdh24	0.5498053	0.3003203	0.3098936	1.55819475	0.82370595
5‐Sep	0.24527199	0.84257706	0.42584614	0.42221293	2.21117508
Cybasc3	7.00141008	5.25938839	3.18308432	7.07763054	7.69683191
Rapgef2	3.31888929	1.96429123	6.17416368	5.31626924	8.13053211
Srprb	20.341861	14.363313	18.3920825	27.0825241	34.59369
Dvl2	5.04362982	5.96660209	6.43969314	12.8102998	9.6680711
11‐Sep	25.9778995	38.5466025	18.1830178	31.1184246	51.0156975
Arhgap21	3.70393417	1.83608045	3.23924029	3.32323721	5.46409525
Ampd2	3.5017784	2.4971016	2.56429376	5.28166391	4.8391819
Smpd5	0.50080288	0.11235241	0.4306115	0.95239934	2.3505814
Camsap2	3.15096749	2.27779686	3.51981999	4.04846228	5.37737918
Fam211a	8.97318107	4.723006	2.76967408	7.69495825	10.3513977
Tfb2m	6.02036023	6.27507494	9.52148818	11.0788465	10.6734963
Gnpnat1	64.1573996	57.181175	144.700132	143.08786	132.518763
Gsr	106.530324	100.109615	132.227743	71.3642552	143.082116
Atxn7	4.273212	3.13116349	8.46024267	5.27748903	5.21659185
Letm1	10.4563571	6.0915831	5.06678585	7.2944805	9.80577782
Prdm1	3.93070654	3.56431089	6.45765898	5.50620494	11.156653
Thap11	44.1449262	42.0332228	46.7264742	53.0988043	84.3527796
Plxna1	5.57698287	3.26162087	1.27098112	6.70525762	4.21440286
Gemin5	3.35818163	0.81577994	1.70057481	3.22761194	2.28741922
Gab2	1.44360098	1.33806991	0.76385796	3.21869891	3.02689553
Mon2	1.26175472	1.39804056	1.36309215	2.21382444	2.24690692
Bloc1s3	10.2404398	13.9598178	7.50696614	10.1197578	17.5521165
Pex26	4.64429801	4.17306305	5.82273199	9.78781732	6.91905869
Kif21a	3.89006825	2.17385553	2.19870945	2.69942822	3.84603082
Zhx2	1.20762388	2.18913177	1.0768241	1.58776771	2.67619251
Chia	0.72742698	0.53758122	0.9509446	0.23570785	1.68019321
Snhg3	96.1014372	49.7985352	106.260987	90.8322028	85.7404641
Cd24a	1295.40005	837.770605	1200.48403	714.876975	1143.44799
Lclat1	4.35813482	4.70386574	6.33003053	8.99251246	9.40260111
Stau2	3.47494816	3.89792891	3.02969633	4.88902114	10.0717622
Gja3	2.86273231	1.59295369	0.77229324	0.16555767	4.22683734
Tti2	1.245429	1.86510202	1.77148303	2.62865993	2.25327404
Cttn	63.2288333	53.8418953	58.3802681	102.649853	132.970291
Nus1	20.8044228	17.7340636	23.9236557	23.4989585	48.3047186
Uxs1	5.88609074	1.94601849	3.8248603	3.69742188	5.62708751
Nmt2	2.11929305	1.30944132	0.86861722	2.80689492	5.48466448
Sgsm2	1.69879191	1.29087256	3.0192491	4.43992371	3.09592383
Sntb2	0.28013161	0.19680723	0.31400737	0.920449	2.0912427
Herc4	13.9843025	13.9061745	19.0280651	9.69940991	15.6268222
Rad9a	4.36656963	4.20448423	4.01386	7.22768271	7.96887613
Tbl2	5.59472982	2.52716487	6.23903874	5.73015775	8.36408251
Erc1	5.1105922	11.5781961	8.36023814	6.63112858	10.2357728
Mir3058	0	1.12537608	3.98142739	1.31581962	1.14851511
Gsk3b	20.880629	24.244748	18.2144625	13.5064175	24.3339045
Tnnt2	0.3646021	1.14515106	0.48230816	0.52601256	2.83826339
Aimp2	36.0452799	15.6854633	25.6317688	39.3574418	48.2003202
Plekhm2	1.70405576	2.19289045	2.19156722	4.09948013	3.18627493
Crispld1	0.84648224	1.94286095	1.70332076	0.79208662	2.3480626
Tmem127	22.7744528	20.5353645	20.6497155	38.7432788	39.7422504
Orai1	10.6653004	13.1911399	14.5556531	22.3671754	19.9142454
Efhd2	35.9335307	21.3764737	19.4013067	28.2124769	46.7705165
Atf4	63.8129461	52.718487	62.4853292	94.9586974	113.906067
Dgat1	3.56713515	3.79293419	4.71381341	6.10637773	7.47385576
Jmjd7	4.85669127	7.25676346	4.45355616	8.31164151	9.37082035
C130074G19Rik	0.61714533	1.22043939	1.24876058	1.46893989	2.83908267
Synrg	4.70017298	5.72077254	9.03753177	10.3516499	9.75581941
Grsf1	22.703329	14.5395811	20.873821	27.4746386	30.1010214
Dnase1l2	6.64779359	4.34990632	3.27329595	2.22816381	10.823547
Lrrn2	0.19489447	0.12242585	0.6677359	0.46521656	1.63987775
Bcl2l2	4.97425196	3.9354021	3.3324275	2.14500269	7.48907515
H2‐Q2	2.84819026	1.07161607	1.64836166	2.28801756	2.04464952
Armcx2	0.82852332	1.85681095	2.28561473	3.45463601	4.39858793
2310057M21Rik	5.97921092	2.26650945	3.2456246	5.92207977	8.2139062
9330133O14Rik	1.89555425	2.99495246	3.38207273	3.56544672	4.79782927
Arhgap29	11.8380356	5.83471015	5.7537753	5.10481141	10.3180206
Arhgef3	4.47477702	2.68520696	6.65329649	4.94322417	9.78095975
Stat5b	5.93160351	6.62404383	3.78377047	3.83949976	7.28838574
Tmem86a	11.6150357	6.31838032	17.3636325	17.2154904	24.1592174
Cep76	2.27806606	0.73351668	1.24442537	1.62501574	1.75986345
Hecw2	0.47755164	0.60086583	1.44915433	0.33278604	1.59529499
Thap6	3.23128982	2.88574859	2.4660352	3.19479428	3.74182034
Nrbf2	27.7031213	21.5236824	18.9357043	43.6724872	38.2072267
Soat1	20.9740418	24.3556313	30.6278686	10.0596266	28.8098476
Fem1b	11.5150379	7.88977065	10.5231764	12.9425762	24.671308
Ywhah	249.201814	150.769134	170.166523	214.906762	343.998507
Brpf3	1.09177448	1.20874774	1.56699686	2.28533614	1.71479287
Pbdc1	34.8841455	14.2332788	23.1026163	27.8203657	21.6891313
H2afj	102.796752	114.74093	197.713718	154.933979	147.476381
Xylt2	3.04727521	4.09756529	4.35604281	6.27723587	5.26542524
Btrc	1.21659753	1.81341233	1.45114898	2.12975547	1.7432916
Zbtb39	0.94255551	0.77452354	0.80175018	1.09677436	1.6687249
Aldh9a1	7.89537928	2.62077962	2.72830134	4.90285631	6.36386022
Atp6v0b	100.240057	124.824093	151.144338	208.161739	164.485007
Cmas	20.7491739	13.5284911	17.0518955	32.1459967	33.9673345
Cnot3	5.05081933	5.69331825	3.32585099	5.54388098	4.92892872
Glt25d1	29.3155348	12.9580988	15.7179394	32.4098658	22.0956472
Trpm4	2.04314599	1.43541785	0.93884778	2.18605839	1.85886292
Cdon	1.80792042	1.54735311	2.26841283	3.25325184	3.53501935
Rce1	33.8685884	37.2525943	32.990412	48.2353448	54.064403
Ifitm2	1203.97212	633.973524	753.608428	1521.95315	1798.83936
Pwp2	2.12262281	2.6587773	2.57388841	5.75343914	3.88792097
Lsr	75.4562453	46.0300527	46.8212447	81.2381111	148.154757
Fam98c	2.67218901	2.01751819	0.85652457	2.31175717	2.18254074
Prrg4	5.27009438	0.80963019	2.16418418	4.83838803	4.11302391
Ppp2r1a	52.1751328	63.0978812	58.2436706	96.067664	93.3731079
Apba3	11.0801067	11.4220064	9.34290657	14.9461372	24.1817759
Pik3cb	5.88436754	6.49815424	7.31486803	5.43934459	9.01101676
8030462N17Rik	1.25920758	1.06364173	0.9893385	1.85669925	1.69706717
Dusp10	1.4667821	0	0.9574315	1.63361529	2.17739324
Rbfox2	2.37520152	2.67227111	2.83952212	5.76891589	7.9543803
Flrt3	0.83365175	0.21443412	0.98489942	0.47505236	1.54342003
Notch4	0.37523383	0.28688229	0.32002943	0.21757647	1.50346959
Pon2	41.0304901	25.1317769	17.0709976	38.705012	49.7999258
Dyrk1b	4.70217116	8.86121551	5.13329639	6.54364339	6.22726203
Arpc2	181.751553	147.746306	168.752771	240.416394	307.949121
Hspa13	10.5003201	6.31289731	10.7099536	10.4027542	12.5588651
Cyp51	15.4302872	6.42263218	15.4811946	7.96338388	20.9726036
Mrps18a	168.91639	89.489541	108.88866	225.551129	141.288628
Ppard	0.44582261	0.74786101	0.48788363	0.70697704	2.38714834
Smg6	3.68846942	2.79024957	3.6966147	5.19305152	9.11058278
Hspa5	324.487983	142.496014	199.505887	278.865988	529.566007
Lin54	3.48585621	1.92571748	2.14339256	3.66833719	5.07889739
Gm5662	0.21241593	0.5003708	0.88512189	0.0650052	1.98589611
Ostf1	38.1986793	36.8051817	39.7399415	42.1254201	77.3800332
Cdc42ep1	2.30883244	1.55104449	0.90268659	3.72125384	2.11743355
Pkd2l2	1.56605934	1.32574792	1.63141415	2.65089514	2.31383777
Trak1	4.28514889	7.69379424	6.79871855	12.6480299	17.9718517
AI661453	3.20097125	3.32748569	2.02803298	3.14102252	5.80522808
Pom121	3.73906838	2.35792603	2.85641198	5.10625719	5.7397974
Tmem48	3.41753808	1.23240913	1.66433763	2.11496551	3.20523104
Rgs1	0.09806269	0.07699942	3.31436368	1.66555063	1.49306965
Crb3	93.5302606	72.7761045	105.725955	138.798482	194.518819
Timm8a1	17.4743073	6.53583799	13.1182928	21.4073731	18.7738048
Mtfp1	2.14880925	0.14671809	1.34092725	1.41526015	1.60964887
Sema3e	5.72182438	4.56744239	1.03854072	4.40668202	11.0212086
Nr4a1	2.6337516	0.41360752	6.09702969	2.34546442	6.54273251
Tnk1	1.74167867	1.9400505	2.1003472	2.06383447	3.7164451
Gpr137b‐ps	2.83818302	1.96739922	2.19693746	5.39459201	3.74917353
Artn	0.89864524	0.11760361	0.24963934	0.24750948	1.80032514
Hip1r	2.64291456	4.39529566	3.43721437	4.13036139	5.61600879
Ppp1r3b	2.93676725	1.68699716	4.15066243	0.70952587	5.00403054
Dnajc16	0.83326974	1.64880681	1.29627868	2.72344061	2.17683679
Ikbip	4.04598074	2.69104528	3.62897581	7.06492689	5.93776725
St5	4.84116829	8.17961426	9.15247408	4.76206925	11.1650336
Gm7694	1.34404474	1.01527537	0.86646857	2.37417389	3.81739696
Efs	2.50182063	1.08976491	0.81167156	1.24065099	1.84386367
Tmem63a	6.6037154	5.26432098	10.4774189	5.15704606	7.35702118
Dyrk2	1.86144739	2.15648809	1.3845878	3.3899134	3.66804663
Ero1lb	0.81533842	0.8817968	1.58120618	0.57906617	1.70123293
4930539J05Rik	1.69890173	1.74444568	1.21011988	2.03965226	2.19921081
Mdh2	193.905585	166.075487	210.122781	290.149004	356.630517
1810019D21Rik	5.50694098	15.7720158	19.1867227	12.9487523	12.0325449
Pdgfra	1.61649876	2.69817977	2.6351766	5.74970401	5.03415126
Ubp1	12.2568181	10.3020701	10.314984	16.1797649	20.4444337
Slc38a1	0.9403664	0.37834417	0.51094431	0.57793508	1.9617558
Slc35f6	4.35873071	5.01745382	8.01345154	11.0531837	16.8200448
Mrps18b	6.13101355	4.42508989	5.21845524	11.2575679	8.98252253
Jup	24.4495015	18.9627779	13.9961997	30.2635886	37.0674139
Stac2	0.25665473	0.35267197	0.5743406	0.70689162	1.79961658
Vps37b	5.08817323	3.70988826	5.86542023	5.10037247	7.82197316
B230216G23Rik	0	0.10000901	0.47175767	1.0523987	2.00728439
Nmnat1	3.07602309	3.11306863	4.81054364	2.63577704	5.09427852
Eepd1	2.02490469	0.65131193	0.65513074	1.29908267	3.55811959
Nhlrc3	1.08686149	2.92128874	1.97412441	2.37944048	2.31138667
Tinf2	7.01241285	1.85502618	6.31976235	6.61012088	9.3756875
Csrp2	8.36235389	4.08945716	2.92075841	6.59981968	6.46604966
Slc19a2	1.49744009	2.06481772	1.03149927	3.52076631	4.68282836
Relb	3.43843454	3.3748494	2.49774244	2.23151046	4.60815587
Lypla2	42.8194773	42.0437112	47.3072552	85.4638473	119.670517
Fam210b	5.02880713	4.58781956	3.19931528	7.63941877	5.56639558
Kcnk7	1.80981412	0.5959355	0.91901942	1.17917228	2.61988945
Mgat4b	26.551816	16.4849758	10.3417672	22.7746762	20.674927
Foxp4	3.28893739	2.77331809	2.29537944	5.90516962	6.63442253
Tom1l2	2.29618625	3.76986577	2.23254335	4.59947192	3.36227432
Ndel1	47.0207702	50.9016257	35.373451	32.9714032	54.3335996
Slk	11.1778216	8.2233295	14.4391401	12.7489445	21.3043836
Plod1	2.4263882	2.42481863	2.74814801	4.06864213	3.37455779
Snapc3	8.0043229	7.68993444	14.4313567	20.2735977	14.1491257
Tmem87b	10.6946042	11.6766899	11.2878893	16.968721	18.2256576
9‐Sep	19.5497394	29.1276526	21.2898494	10.7858318	31.8227324
Camk2b	1.58098431	0.06145537	0.7827146	0.70418143	0.76517132
Vmp1	88.21098	88.9110005	185.466604	191.721515	254.40873
Dusp4	0.24182332	0	2.51293086	0.74004688	1.72253606
Isg20	106.178007	200.660807	494.743964	180.786306	320.199633
Hmgcs1	29.5965329	14.6454859	7.94587641	16.3032576	41.3698083
Pla2g12a	53.1077061	14.4574085	19.8240902	40.4682154	39.6827943
Btbd10	11.0424788	8.41090186	9.02358491	8.26917934	13.9258018
1110046J04Rik	1.67855057	0.2636016	1.78745764	0.61642	0.87432006
P4hb	237.953293	158.597105	156.244357	248.254413	381.100439
Plekhd1	0.48072127	0.05807158	0.75331421	1.71444544	1.0223315
Btbd3	6.06056594	6.01110681	7.59685843	5.30640112	8.67037902
Katnb1	2.62750749	1.36047289	1.09310041	2.15006847	1.76988694
Sidt2	10.6137007	9.13458244	9.57061194	19.4743486	20.6875571
Kdm3a	7.70936488	6.92713209	6.83210136	9.78979955	13.6720658
Wasl	35.6664181	31.7789016	35.0401944	55.6303509	66.553472
Rom1	4.35884163	3.4561435	1.42454741	5.17877631	3.62993997
Tbpl1	22.2059979	33.4477428	37.4491906	52.7687148	37.4617666
Ehd4	13.015383	13.9841351	14.9759034	26.0549379	23.0815255
Dfna5	0.36687308	0.48011825	0.11323954	1.59989601	1.64146663
Pde12	11.9188268	10.9257785	15.2801896	19.3232343	31.7187395
Klf13	18.4338234	18.5332514	17.3904217	21.6695701	29.4050216
Col7a1	0.51046332	0.33958266	0.44642083	0.33846806	2.22710541
Fig4	1.87860828	1.31816959	1.27691197	3.30265483	2.81866658
Zfp7	20.056028	35.9956892	49.4437671	47.7175727	38.5763874
Tmem65	19.6851972	18.7450471	23.1497859	17.2183158	28.1794361
Adam19	0.3267221	0.33671421	0.50108095	0.56242173	2.68364484
Tbc1d9	1.02590209	0.35023674	0.09293174	1.03400292	1.57272726
Srcrb4d	1.98699348	3.28957673	2.34978271	1.84620506	4.14376158
Spcs3	61.0912741	39.9177324	26.7254941	45.0664424	70.4103387
Col4a3bp	2.49270863	2.44422535	2.96126241	4.67750199	3.52735268
Ppp1r21	8.92370488	10.3389216	9.65004019	11.7256947	18.7026619
C430049B03Rik	0.91509888	0.34216246	0.6052621	0.5000818	2.40946422
Bace2	9.19875813	8.62090489	4.25086999	1.66661093	11.4418052
Gnb5	3.3997783	2.43739367	1.6196954	4.6140679	4.93553435
Tfdp1	42.7711964	20.1505207	21.028182	40.3945014	50.8434129
Hipk3	10.8831141	13.8531153	28.4121974	20.342631	26.630658
N4bp3	11.3080924	4.63867593	8.26419661	9.00840195	16.9247456
Atf7ip	3.74531462	5.76857784	3.30803524	4.66844198	3.947878
Tmem150a	3.5844836	3.45885455	2.23944304	3.52874938	7.95974217
Aldh1a2	4.60859997	2.89496037	5.06764425	3.8608612	6.18595111
Tmem88	0.9462895	0.22614024	1.25722676	0.83100028	1.61552962
Snora3	2.17372298	2.13352548	2.01283274	3.99131952	2.61287189
Pdgfrb	0.78307035	1.05001143	1.37213242	1.44890871	2.2107802
Zfp609	1.89900086	0.78730445	0.71041155	2.05726137	1.56437525
Trub2	7.03759693	3.18041065	7.38403313	9.13386202	7.03933652
Hid1	0.6648087	0.26891517	0.03730923	0.27743185	1.56594731
Dhx29	2.1482621	1.5618781	1.31143568	2.00150357	2.1040696
Ccng2	44.7121047	51.3483294	34.9622751	55.4287697	82.5139493
Gm5506	1.01325698	0.18013936	0.33635727	0.57921523	2.80360923
Plcd1	0.19686548	0.3671274	0.29622821	0.83591786	1.79450069
Actb	1036.58624	795.148795	863.7386	1020.57554	1604.21205
Ppm1d	8.76549662	6.12190587	16.2751507	12.8055984	16.8112213
Epb4.1l1	2.01128581	3.82639954	1.90874732	2.77438659	6.22245997
M6pr	150.585589	92.9840718	132.890442	234.790898	218.460549
Alg3	8.19150346	4.0245028	4.0680409	9.62118073	8.03114306
Syndig1	0	0	0	0.64094182	1.93263517
Slc29a1	3.11159699	4.24462004	4.56611065	6.85125409	5.72655302
Erp29	135.984473	102.532806	83.363338	193.962716	162.237693
Cbl	1.71913563	1.07788578	1.19985898	2.06122639	2.89919288
Numbl	1.74184169	2.84782325	1.03845377	3.00115683	6.74968002
Wdr5	19.6556074	11.8001671	16.7655169	25.5111725	27.5418118
Fn1	8.1427534	6.49705991	7.28203463	5.85551445	13.7637097
Fkbp1b	1.66356351	1.25399049	0.06161733	1.46619901	2.61287189
Fbxw9	8.74445565	7.89473924	7.21210426	11.6684341	15.830429
Twist1	1.04018646	0.97383004	1.00024203	2.49763553	2.21212466
C1qtnf6	2.09493896	1.47624498	1.39273435	2.41649081	3.3575619
Rnf180	3.63531479	0.91992433	1.99422001	2.68899994	2.77509775
Creb3l2	1.76885234	1.60496673	1.18294871	1.84048188	4.2209142
Tmc8	0.67274783	0.79236847	1.16284697	0.63822681	1.54544004
Fcgr3	3.47024512	3.21684551	2.76708713	4.73471384	5.48452044
Edc4	4.21264144	4.22294708	5.18811538	5.9302551	6.72910158
Il1r2	0.72780904	0.72387472	0.31450512	0.22272988	2.83838761
Snora74a	0	0.77582745	1.82984794	1.20949076	1.84748517
Acrbp	0.84769142	1.64025914	1.59793128	1.77886107	1.35859634
Pld2	4.42636567	5.64449677	4.06697064	8.71033818	14.8619548
Cmtm6	40.6989423	44.2388291	52.8008301	54.9044579	81.0676241
Plekhg1	1.83831172	2.76949679	2.53300909	4.02146706	3.26344668
Ttll6	0	0	0.07533997	0.54155458	1.79298757
Gjb3	15.6413054	5.70124187	9.14257532	2.83462599	16.0415652
Hist2h3c1	5.60999192	3.78773839	5.72416542	4.26469757	7.38762681
Hhex	1.06783681	1.32998991	1.60253764	1.52125391	2.89171592
Ahi1	1.57460499	0.50259729	0.8890604	0.95199317	1.77474219
Arhgef1	4.02474147	3.35954638	3.79399665	5.12646544	6.15989813
Tmem50a	64.6761921	76.4798805	69.0901163	107.612675	102.130868
Lef1	4.43941798	6.26343363	9.84306325	13.2827476	14.0316961
Synj2	1.43309591	1.1984642	1.31623509	1.78635973	3.31452392
Nop2	7.13524777	5.60263954	6.97546685	14.1058071	9.84185064
Map4	22.9254068	24.7893498	19.0783125	45.9445325	35.5869714
Akr1b10	10.406638	3.47473501	3.73747465	4.0181069	10.4826627
Tapt1	15.8309834	15.116294	17.0303303	18.2406391	25.342386
Lrsam1	1.94881744	2.35320038	2.60829154	3.78884676	2.62468296
Hira	1.69754674	0.76812565	0.90584134	2.06037672	1.77534644
Sema5a	5.65904013	6.73158969	5.75972953	12.3350938	15.7705395
Lamc2	51.8384945	31.3930674	18.1716232	14.237046	62.5895144
Sf3b4	44.3070584	30.8374266	29.7476989	51.9671213	68.2087357
Palb2	1.54892991	0.40996486	0.80577772	0.83085915	0.86468138
Gpi1	25.8700435	24.6162226	22.0312154	27.0629911	47.9575435
Arrb1	1.15532521	0.45358416	0.50943461	0.91757697	2.36598635
Dnajb1	41.8230447	33.7443818	38.5140963	39.6134936	69.1533937
Tmem181a	1.67477854	1.20545902	0.8400265	2.43451271	1.98516751
Nsmce2	7.03165949	4.17996829	5.11331861	8.09691989	8.02246378
Rpusd3	2.31247126	1.69471528	0.57101638	1.65125512	1.72956059
Trim41	3.94652876	3.68909305	3.37599035	6.26303596	7.22869455
Mir30d	0	3.74667889	0	0	1.27457165
Arl13b	4.20063948	4.40266666	5.80888448	6.21803183	6.88066149
Ercc1	3.50836258	1.99722089	2.19286418	2.89887363	6.99342979
Ormdl3	43.5819102	63.5058677	85.6672756	54.3060157	96.3779041
Ddx28	11.8154798	11.0291066	13.1678635	14.1114798	23.6010341
Hip1	3.46234715	2.90120348	10.0026562	5.67139366	8.54323275
Nat10	2.21543862	1.27480361	2.14542078	2.91896293	2.16835841
Slc25a1	34.0049037	47.1155663	60.2050506	41.6020133	45.9043285
Fibp	6.6263491	8.34139639	7.06114709	14.5329094	13.1065025
Bnip3l	75.687463	77.8430316	78.8530488	89.7029642	164.037017
Ralgapb	4.34172975	3.31122339	3.5439907	6.83428784	7.12714994
Prrx1	1.08569408	0.86624343	1.43503515	1.44690692	1.71899466
Nr4a3	0	0	1.57052236	0.15889011	0.36058742
Tmem185b	9.17111394	7.97923452	10.4980252	12.2701342	17.3945252
Pcp4l1	0.53825522	1.17039112	1.68694553	1.29242727	1.19445572
Nudt17	2.10669188	0.33835646	0.84237493	1.93411959	1.57309467
Mier2	6.82151944	4.73672636	6.03378431	12.5254524	8.42401318
Pacs2	3.01068567	3.56002865	2.95313416	3.68177375	5.7311533
Gpx2‐ps1	1.34523132	1.71017367	8.30441796	3.58748267	7.28934789
Ccnt1	11.4832538	13.1601737	13.1319984	21.277862	20.4224469
Ralgapa2	1.49966529	0.40494802	1.25671139	1.52010712	2.24581912
Tle4	1.70366434	1.31524469	2.46577534	1.06464396	1.73235414
Snord104	55.3852706	24.5501562	66.1753228	78.7328782	51.5415824
Epn3	11.5514012	7.80461638	10.0278871	19.7613477	17.8945397
Eif4ebp2	5.40388021	6.70877889	4.09103182	10.2911682	8.89488301
Spag4	0.04341657	0	0.52263966	0.59790073	1.60042752
Snai3	1.35430192	0.2577954	2.12810509	0.56516482	1.84167181
Rps6ka3	2.34679944	1.01349658	1.16804059	1.37353101	3.00897527
Gm19710	2.54733162	2.24020175	0.75481228	1.59029137	1.95965391
Casp8	44.0067361	42.0220777	26.9199164	18.9720625	42.7355567
Rimkla	0.09625342	0.1259646	1.45577571	1.39917105	2.57109165
Plcg2	1.42622122	0.48845721	0.67437858	2.11731236	2.16422148
Zfp385a	1.05317875	0.24585366	0.55350704	1.17596712	1.55107192
Cdk19	5.06465851	6.6133044	8.61008526	11.5058875	12.7786398
Akirin1	65.3492669	38.8031571	55.518513	34.5577538	80.7013595
Dusp14	31.8149892	26.6845931	39.5452585	39.7057462	70.364658
Ntf5	2.95661761	0.39127314	0.18456948	1.37246086	3.99318169
Mtmr1	8.51382654	6.1768187	5.86388475	12.1832574	13.4195837
P4ha1	6.43568245	5.33196672	4.64499862	2.76914451	13.4036515
Fdps	23.0762789	15.1756309	19.4670271	21.6367218	41.0619702
Asns	16.8866804	12.7280794	27.9832844	18.5792092	33.6269968
Usp24	2.87273456	1.46837109	1.86834017	3.14004384	2.28235012
Efna3	2.71493565	0.62699524	1.23234657	0.3421131	3.58336716
Sash1	0.989569	0.47761506	1.05923817	1.95037331	1.33135335
Ptprj	3.60311771	1.75583486	2.42915474	1.79906805	5.66707946
Cflar	5.86003662	7.42637287	6.64760427	7.07370952	14.6765
Gatad2b	1.47119761	2.04173687	2.47115876	2.85842138	2.54981202
Cnksr1	7.850779	6.30976972	7.04940204	11.2119353	12.7307337
Alg13	2.0028012	1.00419775	2.07800308	1.46213	4.21540108
Pgam2	1.58350681	0.79124124	1.06640145	0.66081449	1.84573731
Lnx2	4.24887962	4.70004909	3.08371469	3.37368852	5.26830431
Tnfsf12	2.57921086	1.45089808	1.78231942	2.50930064	1.82006424
Tmco4	3.0819492	2.89779259	2.32669407	5.62293057	6.65409276
Gss	10.0784894	6.1489652	10.8608422	7.38297391	13.9859701
Jarid2	12.1252654	23.3987946	23.0341544	16.1389828	29.0139947
Bin2	0.62640147	0.67306365	0.76320756	0.81723175	1.61157922
Aebp1	1.93267541	1.08757634	1.02903526	2.09965685	3.78153353
Otud1	4.89925702	3.56346842	1.45649818	5.2554741	5.16582026
Slc39a1	28.9056934	35.148558	48.9468711	47.0478491	77.0485088
Map1b	6.92172675	3.81485589	3.39831109	3.74817636	11.3183296
Zfp607	1.31872115	0.71461862	0.85993993	0.98901965	2.87259626
Paip2	319.418212	313.304621	392.035926	409.881046	500.445701
Ggta1	1.72758241	3.18422431	12.2420195	3.50603918	4.53208676
Snora28	16.7071271	12.2721796	18.9638787	25.7291672	27.208418
Trim3	2.53784917	2.49525011	2.86087901	4.98408427	5.05774186
Myo5b	2.44286305	5.76215577	8.52130482	6.75527976	9.4498932
Surf6	10.5130995	10.6277484	14.9786735	22.9640755	22.9949005
Asap1	2.33509986	1.9204693	2.37662763	4.85131057	6.56545058
Isyna1	21.8909752	10.1684892	12.6486497	20.4560554	31.0758321
Arl8a	30.0004424	40.3740239	48.2866983	43.3721824	62.6352367
Ctdp1	4.51261352	2.72350173	1.68782989	4.92280793	5.89934711
Zfp703	5.99829156	6.07328524	18.8371911	9.40164068	8.807078
Lmf1	2.97850275	3.68695215	4.08839749	5.30817615	4.60516915
Rictor	6.02191056	4.76142957	7.40466547	7.75935143	9.68499275
Pla1a	0.99105911	0.36315327	0.55062294	0.63691269	1.87957349
Itpk1	19.2940748	32.2359564	21.3123466	6.1630669	34.8931841
Amotl2	9.39820102	5.03371509	6.39726367	12.4710878	19.1772701
Zdhhc8	4.90293311	5.42760467	3.68412894	5.68197294	7.9116129
Tmem67	1.41559384	1.70435031	1.1010865	1.38627595	1.60326726
Senp2	13.1823017	8.79203141	8.1730002	9.41447902	16.080092
Itgb3	0.04513701	0.38986034	0.13583748	0.22791754	1.70001701
1700017B05Rik	20.8428931	35.803792	33.0440721	62.6849107	42.6193614
Slc26a6	0.21537806	0.90195279	9.90535302	3.75696681	4.39154247
Ran	142.080539	58.8808273	85.7741222	137.585389	149.372221
6430562O15Rik	2.15982935	2.64800141	9.22791997	3.61793053	9.04864406
Dnpep	11.561168	11.1039805	10.0848506	17.2287174	27.3908461
Zfp217	3.15846451	3.26875237	5.0019393	5.553556	5.92960486
Ap1g2	3.12274458	2.37537293	1.4683965	2.73255321	2.44376345
Fbxw17	7.89116243	4.64714121	3.04462094	7.81493654	9.22190077
Rab11fip3	1.54607514	2.61550155	1.03590274	2.57343173	2.70957031
Fkbp14	3.35430778	1.39437671	2.30668804	3.07953548	2.82632889
Shisa5	80.172217	84.0593571	111.482372	119.801595	136.52966
Stam	6.51762677	6.04943129	9.05282729	11.4796507	18.2375903
1190002N15Rik	7.01644761	2.70930729	3.83712164	4.85021106	7.5806359
4931406P16Rik	4.60344227	3.85268857	7.89121924	8.41145849	10.1311768
Col1a1	7.13469741	2.94279377	2.94341441	5.83660389	8.85417633
Wbscr16	7.45276452	2.79297881	5.59410656	6.89409735	6.0401454
Trip10	13.2386628	6.99949124	11.4726553	12.8849147	24.7893297
Dync1li1	8.71625531	12.1280702	10.9494144	16.0927649	17.4362655
Rmnd5b	23.0888253	17.0243493	13.6533361	19.0131202	30.2910533
E330011O21Rik	1.23819664	0.32407982	2.93007297	1.57884475	1.81908802
Crem	9.75185388	4.50282735	50.5314251	12.5989806	13.528201
Mgat2	78.8971703	39.0493884	56.2069691	93.8555123	114.962368
Acsl3	10.7310375	7.09086646	3.11861679	4.06205176	15.055434
Vapb	3.7710304	4.65830445	3.04134434	3.42775826	5.13651095
Gm4636	2.09203404	0.81351659	1.03316804	3.19195809	2.71425738
Sipa1	2.05683199	2.11808114	1.70164505	1.98121341	3.76934465
Mtx2	77.0824979	49.576484	78.4812763	109.079146	99.7793047
Cdr2l	1.16323554	1.30087392	1.50146442	2.41097274	5.35285646
Cbfb	50.5038008	18.3689732	28.4615792	37.0769331	39.4128358
Map4k4	5.26147211	2.68279937	3.61865142	3.09671342	5.79957837
Magi3	5.29245785	2.18153461	1.9929037	3.12551574	5.03289454
Epb4.9	0.88858719	0.4790344	0.18421288	1.50983411	1.60481454
Mtor	1.74504812	1.51320202	1.22947898	2.74446752	1.90303409
Fbxw8	7.15258788	5.81169255	5.507188	10.9564848	10.7286262
Gm15708	0.51696696	1.73968103	1.64126792	1.45775826	1.33158816
Pgm3	1.01048231	0.8269623	0.71165191	1.65942027	2.08710413
Setd5	3.69902474	3.05486554	2.0719422	3.74764545	4.15959512
Rtn3	55.1024538	57.9939487	50.1849781	63.6369076	96.4553222
Iba57	1.91337123	1.05391581	1.32220237	2.70049865	2.36857666
Zfp652	4.01302705	4.51522841	3.17219418	3.70676015	5.8434208
Idi1	3.73028151	1.67883972	1.45328349	1.23206061	5.1036214
Stard4	3.18958348	1.49239571	3.05465068	4.7935948	4.58674998
Col14a1	2.70029794	0.17470556	0.18729833	1.31847248	2.55289902
Zdhhc9	9.73276301	7.11222651	7.64137168	16.2518002	20.7633893
Tnks1bp1	4.05090885	3.63519776	3.74056962	5.25045686	7.33804632
Ssh1	0.96564254	0.96795107	1.1985677	1.24492953	2.04156341
Eya3	3.07424744	2.86714296	3.34632958	3.8016224	4.5550547
Ccdc51	8.56974643	11.3645613	21.0352357	9.64110973	19.4248548
Eif6	69.093857	50.9648575	63.414329	96.232182	137.092756
Tpd52l2	40.2412117	35.7277623	43.1022031	69.3980729	73.5592553
Ctsb	405.802871	423.844202	844.422163	806.557063	1469.69774
Tmem231	1.10681427	0.90454977	0.52290424	1.51385344	1.62908535
Vopp1	27.6347527	12.419691	13.5583744	20.2055364	24.082603
Gpd2	11.357895	6.42323908	12.2174976	7.2881454	10.132533
Il1b	0.09828439	0.07717349	1.82019539	1.53396606	2.0871471
Scyl1	7.29520228	6.38844035	4.53402596	10.330207	9.84905064
Stk19	11.070259	16.0548782	20.3897342	15.4950577	16.0941274
Tspan7	0.51463566	1.03910147	1.53174983	1.95741149	3.41705906
Mast4	2.4446744	2.8937564	5.54047773	4.7020512	9.05567032
Cdca7	50.9871999	15.1163127	36.5737079	28.375391	35.1924295
B230120H23Rik	2.20474208	1.37145173	1.57756594	2.27386919	3.90066494
Tstd3	0.78073226	2.38761264	2.09273725	1.84853172	2.53549005
Bsg	279.4574	197.804116	188.186074	299.827376	515.654442
Renbp	1.05773544	0.72220891	0.2980923	0.97108973	3.24305678
Zbtb7b	10.4444739	17.4845015	16.5935967	10.4974948	18.9061462
Hspa4l	5.46179974	1.92844799	3.53009451	5.99034464	6.05117041
Nat14	1.94178728	2.28705461	1.04887066	0.81708154	2.13957251
Cldn7	451.365424	460.136692	589.304229	534.172053	927.146928
Tspan1	40.7614417	71.1564845	155.389462	79.2796343	138.981983
1700084E18Rik	0.75128675	0	0.27827181	1.37948831	1.6857238
Lca5	2.38645874	1.25281045	0.64400546	1.46481927	2.57899525
Rnd3	7.31238259	2.42710221	2.64865149	5.73919839	6.37736682
Ankrd28	1.79584687	2.72229528	2.95631199	4.77446999	5.19676183
Cd97	1.56363719	1.18055514	1.22514873	1.21469607	1.76708212
Amigo3	0.48722851	1.32894391	1.85215438	1.20069187	3.2673742
Zfp712	1.1662544	1.43166546	1.90129037	1.01039701	1.40844983
Slc30a6	10.3629815	6.88703204	15.4926002	11.885712	12.853741
Eef2k	1.44677513	0.36596506	0.71030577	1.84530183	1.15937436
Eif1ax	72.6765266	34.4497548	53.4726623	77.9729699	80.6434973
Kctd12	25.5269797	28.1173086	42.8387432	16.1807579	43.0000115
Tmem214	18.5813178	14.9424029	18.4303186	22.6516776	30.1397894
Slc38a2	68.9175397	88.734007	147.739661	71.6031338	138.118621
Chml	0.90631027	0.52745103	0.22708545	0.62649881	2.40097122
Kctd10	12.6929144	12.0160596	11.0517382	19.5378827	28.4059151
Plekha8	3.29417951	1.4801562	2.06525626	2.43317418	2.84918199
Fam46b	17.5284917	8.34760494	7.91761128	21.1428289	15.8964933
E2f3	3.82544539	1.32607587	1.84815719	3.40636451	2.31708474
Tmem238	52.9883757	20.6085163	22.1200933	52.2935012	44.1528113
Lmbr1l	2.85884195	3.85156976	3.84546725	5.00066105	5.95643152
Ptplb	8.19897179	3.24025852	4.92733409	7.74335927	9.73209567

**TABLE 8:** Gene list of cluster 8

	D3AM	D4AM	D4AM	D5AM_mesometrial	D5AM_antimesometrial
Entpd1	0.76134686	0.51241212	0.92080983	1.02707293	6.44969091
Kifc3	0.55469805	0.43555225	0.17656428	1.32089123	3.7922064
Arl4d	1.36840738	1.88960578	4.19461525	0.69313798	14.671408
Ero1l	3.23253961	1.72690971	2.7199211	8.40182696	34.6687528
Gprc5a	16.0274022	5.21688703	20.9933303	8.09441911	55.7648176
Fgfr1	0.53388164	0.28628776	0.27732719	1.35089588	8.31652912
Ctla2a	7.53162268	33.1323957	52.1546043	14.4305957	93.830966
Sorcs2	0.30776361	0.45646481	0.78106776	2.34414151	8.42184191
Dppa3	0.31888357	0.18779197	0.29528109	0.07319046	7.21893699
1700012B09Rik	0	0	0	0	6.10305842
Cxadr	12.7590128	7.60960491	16.2711943	24.1253474	41.6570943
Mir22hg	7.93913548	7.74158057	21.5074483	19.7644899	45.1260433
Socs5	5.17600174	4.55333252	9.39352461	9.61293466	29.0107813
Syt5	0.52169352	0	0.10351711	0.06842262	3.73267412
Sac3d1	22.9237599	15.577531	30.324336	35.6865795	189.93715
Tmem37	1.98777028	2.01394736	1.60314112	2.7079749	18.2412885
Il4ra	3.14920998	4.03083024	8.42829255	4.9950259	25.3695806
Mt4	0	0	0	0	5.74698164
Hk2	11.4898477	14.2436345	16.6439462	3.63933598	44.8232459
Plcxd3	0.86258849	1.73391277	3.86591684	2.5975254	12.6634426
Pgk1	56.738877	35.1978396	31.449813	36.8659635	243.036257
S100a6	203.616472	92.4389232	130.93211	261.46196	1010.62541
Calcoco2	0	0	0.02976096	0	8.19017506
Rps6ka5	1.7760787	2.35917752	3.65928057	4.2395314	12.3823797
Myadm	43.6716084	54.3056515	45.1385691	57.5576767	142.752398
Slfn10‐ps	0.54533649	0.37294969	0.26063116	0.98517868	3.21410731
Ptpn12	10.8342858	5.88707473	11.9015252	10.2682557	27.0365392
1600014C23Rik	0.12589129	0	0.4662933	0.2311575	3.83355721
Slco4a1	1.01365321	0.03316361	0.39109444	0.73673968	11.9474582
Abhd2	0.42680748	0.15874664	0.7904338	0.45371527	2.71817881
Sprr2i	0.10569156	0	0.39147476	0	9.99412909
Mfsd2a	2.24449536	0.97646003	7.44280011	0.83539246	7.41326442
Ttc7	4.1684612	3.58376804	2.23745004	7.92642327	14.0976186
Rhox5	0	0	0	0	7.77329386
Pdlim4	27.1852951	6.22233254	21.6061033	11.8223895	81.9802335
Gpr4	3.14300445	3.21360426	10.0920809	4.56704383	21.2544988
Sh2d5	0.12460832	0.0326144	0.28846329	0.55293758	5.45873872
Itpripl2	0.57953279	0.58187059	1.21403373	1.55256747	4.74306289
Got1	3.86768407	1.56825013	3.28785512	4.36580891	10.6953725
Adck4	3.87101353	5.54132097	3.77750801	7.08049148	17.1328038
Qsox1	3.33722422	1.78248722	2.42546045	10.580975	29.8369601
H19	0.27431303	0.92031233	1.31623097	0.11447379	4.75612626
Ano1	2.49288822	2.58024976	3.86915067	3.39400867	10.2153983
Fzd1	13.8377477	4.07017013	4.60680311	10.1332838	25.8422459
Apobr	2.1018494	0.59755312	0.95636121	2.31228152	7.0421943
Ctla2b	3.3543938	3.25888112	3.84315928	2.19226791	15.991596
Dok4	5.25900722	2.69394462	1.64644152	4.50632849	25.3460614
Tle1	6.82118153	8.50870862	19.2115187	6.81054864	30.1535822
Ier5l	13.343508	1.37946997	3.75711562	7.56532981	16.7934421
Rnase4	24.3073632	10.2357916	7.92628522	26.5154794	53.9068458
Ywhag	44.0614617	36.8924094	45.8266874	78.0374081	276.693822
Dnmt3b	1.08510322	0.49701726	0.3628402	0.66414376	4.27527916
Gzmd	0.13919251	0.1092948	0.7088952	0.51116152	4.51745193
Gyk	2.8429763	2.44051797	1.3503757	1.85275844	20.3623402
Nckap5	0.08588396	0	0.15905435	1.15119054	6.37990844
Serpine1	0.15105289	0.08471974	0.17983615	0.01981132	3.37200541
Pmepa1	8.24681044	3.79711973	12.3941808	12.6451979	85.9347094
Gpx2	148.51397	232.700245	1271.48022	538.996466	1278.5196
Cib4	0	0	1.1007679	0.3897773	4.96717832
Aplp1	0.76334754	0.49235203	0.58062483	1.27648806	3.97610939
Bhlhe40	19.3142235	7.94469238	7.93365167	0.73082304	27.647928
Tor1aip2	24.9509091	20.6965125	30.2892857	31.7583044	70.9889262
Tnfrsf11b	0.94878612	0.87218643	1.30712701	1.04102904	7.39911911
Mir374	0	0	0	0.63020835	2.20031317
Car9	0.59918857	0.67959158	0.6781366	0.48898248	8.26942608
Crmp1	0.1708106	0.07451195	0.24603893	0.40075822	2.78321045
Plod3	3.68034245	2.31186166	4.1447898	7.06821532	21.4736939
Dusp2	2.01519436	0.94940565	3.84403779	3.33021097	21.5747641
Fos	7.56754563	5.99098846	33.3934141	10.2928011	44.496185
Ralgds	6.06695818	8.61052672	11.821637	15.0270201	27.9096315
Asic5	0	0.030902	0	0.7587602	4.09985932
Rab11fip5	1.55468757	1.10369182	2.34677102	2.95242936	8.76361667
Oaf	9.25743834	7.05457169	8.95154256	8.17317942	23.5902504
Slc25a47	1.11437697	0.29167184	0.84080355	0.8715223	4.00199365
Mphosph6	63.7886641	41.4883665	75.2807082	90.5431212	186.56996
Slc45a3	1.1224043	0	0.19054355	1.45981996	3.50781423
Rps6ka4	10.2877424	5.90188375	5.79353659	10.4858877	28.9552641
Mfsd7c	3.13243924	9.95769447	13.5888184	2.57954275	38.7480914
Slc4a3	0.23494416	0.21906949	0.12237443	1.34811513	3.01236299
Trabd2b	0.31536558	0.02321503	0.03650294	0.35286715	4.38308568
Car4	0	0.6302106	1.30059961	0.23026843	4.82376348
Omp	0.68676743	0.04902308	0	0.22927637	2.67666148
Bglap3	0.32018833	0	0.19765952	0.2939597	11.033076
Igfbp5	3.84318977	2.56285466	4.27047874	6.19765877	21.4867873
Alas1	31.3991148	39.5310213	46.1491443	35.4078197	109.775432
Gm14023	0.51261294	0.20125339	0.1017153	1.27740153	3.11021246
Cidea	0.11204758	0	0	0.20573812	2.91815589
Kdm6b	3.56289032	5.24068872	4.41708327	4.28027567	14.3385474
Litaf	106.286017	88.4692998	133.780944	96.3483478	361.106603
Timp1	2.78431933	2.53146394	17.6405566	8.54321762	66.1731823
Arrdc4	8.50802321	5.98142365	16.9322238	9.23402619	93.2838205
Rgs3	1.24153196	0.6241899	1.48874083	0.50841353	5.99804586
Gm14005	0.78428313	0.34212435	3.24115382	1.18672863	5.34213005
Pfkfb4	2.03725897	3.15363124	5.24618112	1.90600054	13.1537615
Arrdc3	47.1309913	49.2265361	80.1097586	129.44464	240.56309
Rpl10l	0	0	0	0	1.99221903
Spry4	0.82402571	0.3012036	0.80250194	2.30870443	8.58433726
Rnf11	68.8821411	65.6389321	64.7184259	92.9779005	306.436438
Rusc2	0.39836834	0.38863182	0.31299139	0.7203881	4.47893255
Sall4	0	0	0.01190321	0	2.16322924
Rhoc	36.5580684	19.1047509	22.47282	40.9903979	230.704711
Mylk	0.75157537	0.65571279	0.91246355	0.69767591	6.07629062
Ngf	0.76913534	0.04313784	0	0.40350324	4.05028161
Id1	311.312445	34.0052579	28.9662318	290.411212	591.18019
Snord91a	2.17372298	0	0	0	2.61287189
Map2k4	22.4374817	21.6101758	33.3310716	65.3316553	120.189277
Pgam1	32.2498858	25.1271538	21.9192211	35.0656592	122.770749
Hao1	0	0	0	0	4.53292708
Pax8	42.4250169	44.0922971	83.0144154	100.430571	243.544062
Perp	121.945859	93.4618909	152.48797	422.334122	819.644158
Sh3d21	1.06445198	0.83581411	0.44095563	2.57172649	10.2584403
Spred3	0.03215567	0.07574647	0.11910253	1.16610297	2.48660885
Rspo1	0.17778541	0.02791964	0.52680464	0.35908818	6.01216977
Obp2b	0	0	0	0	1.64196867
Map2k3	41.5175069	29.361093	30.973091	37.978345	91.7038415
Slc38a4	0.03316973	0.01302254	0	0.03045259	1.68786739
Gm4926	0	0.03160779	0.48457084	0.03695666	10.7418066
Sh3tc1	0.53040943	0.49527433	0.98230131	1.0528871	6.0080545
Bmyc	53.9682948	41.0976371	65.9854917	67.4869959	228.168028
Sct	4.75903849	5.35275032	2.5011531	1.29895014	13.399343
Vnn3	0	0	0.06668689	0.23141278	11.7153615
Alox15	82.6086771	158.025831	744.356218	193.101163	833.56458
Elf3	132.086277	128.293154	160.020202	26.8216672	292.380364
Utf1	0.2248679	0	0.10411204	0	8.60445741
Tox2	0	0	0	0	2.05909904
Stat5a	6.08843706	4.58312881	2.29860528	1.37047622	10.806322
BC016579	0	0	0	0.24581576	6.26993892
Stk40	3.31040952	3.12983381	4.67627807	7.34998233	18.2041579
Hmgcr	9.08253103	4.97019931	8.11039827	8.33852419	21.2096779
Cav2	36.7854051	12.6717437	20.0225173	40.4780807	73.7478599
Rnf144b	4.82071927	4.51612893	7.70571032	9.15775697	29.3461026
Ncrna00085	0.11835152	0.27879099	0.27397905	0.38029814	2.51328875
Epgn	0	0.09014896	0	0.07026971	2.69874091
Macf1	2.34534487	1.25968357	1.77595329	3.44770503	11.0853828
Rbpms2	1.00017929	0.73299035	0.52481835	0.55094901	7.58748073
Kremen2	0.24643057	0.12093673	0.28523846	0.14140244	8.73837173
Fzd7	0.29832755	0.41525886	0.30135986	1.14535682	4.12930471
AU018091	0	0	0	0.13890903	5.68345103
Eomes	0.40373321	0.85455879	0.65017477	0.41900797	8.56656247
Tgm2	11.1901716	5.5114572	8.50732348	19.7541638	98.2863896
Crxos1	0	0	0	0	2.74761048
Aebp2	9.51168083	5.471835	8.28741894	8.46104236	22.4874433
Stk39	2.30962251	0.96195913	1.35759276	3.35580606	6.98482537
Lrrfip2	6.22111517	5.48172068	4.38995115	8.90700905	18.0015652
Flt1	0.31152081	0.19568641	0.34615595	0.21926835	8.28796305
Coq10b	21.2229619	14.3597468	49.7226598	29.3994193	73.2448449
AI414108	0.34894092	0.08430477	0.08699215	0.07392854	5.00096903
Tinagl1	10.4338703	10.5469729	10.2723878	3.60595074	33.3486545
Ak1	1.54412457	1.34236191	0.91917943	1.8733043	5.54613821
Ceacam15	0	0	0	0	6.27389798
Pdpn	6.08249224	0.20261859	0.17067547	0.67687725	6.41032447
Fetub	0.07310728	0.02870214	0	0	1.75753714
Ptges	0.17880913	0.21060301	0.6291827	1.16554848	18.1117086
Ldoc1	1.85621289	0.49862169	0	0.17938515	5.24531584
Tgfb1	1.37025518	0.83140248	0.5767429	1.25800902	11.030462
Rhob	26.9690014	21.4751267	25.054209	43.4439089	125.446009
Tnni1	0.35122993	1.14911606	0.37943885	0.37620157	7.3648274
Irak3	0.15374935	0.29318909	0.12203095	1.1695682	2.74575961
Eps8l1	3.15457023	5.92852758	7.70973998	8.2611768	27.1647505
Mir1956	0	1.57552651	0	1.84214747	4.82376348
Cldn4	85.4125497	28.6700586	25.5077368	87.4740319	246.368859
Zfp42	0	0	0	0	7.13788433
Snap25	0	0.09665807	0	0	3.99514153
Sh3pxd2b	3.38693651	2.438971	2.57562423	4.34201	14.6393952
Pof1b	28.8339604	33.6186575	22.524064	26.9798835	90.9333151
Snord98	0	1.82873613	2.1566065	0	3.73267412
Pfkl	5.49132602	8.54098982	6.82316181	11.5970543	30.3675183
Bst1	0	0.02191978	0	0.30755031	2.01334306
1700019L03Rik	0.38932352	0.09170975	0.10815221	0.5361474	2.33988527
Cxcr4	1.90530295	1.94769471	0.93207249	1.78223198	9.76586074
Ndufa4l2	0.47352887	1.27480361	1.50336055	1.24211188	15.9374343
Irg1	165.019843	333.047614	168.4853	351.49213	1860.97459
Prss27	0	0.089129	1.62918576	0	2.68336712
Prkx	7.11252457	8.04210789	7.71512703	14.2351673	25.9414454
Slc2a1	12.5758157	12.3631663	11.3867482	31.4479355	131.131222
Cald1	3.66866258	6.38312753	10.50564	11.9294457	29.7355829
Tnfaip8l3	0	0.02301331	0	0.10763109	1.87891911
Snord38a	1.10528287	0	0	2.0294845	4.42859642
Trim43a	0	0	0	0	2.61856442
5330426P16Rik	0.86230333	0.36675479	0.23288974	0.19791667	4.00208476
Lox	0.03618851	0	0.03350998	0.11628428	10.3383888
Defb1	3.55959004	0.44720185	8.96545585	1.24184068	26.5851157
Klf10	12.3141943	4.33851881	11.4622396	7.60906726	30.1359805
Clu	74.1205874	18.8901968	63.0902827	41.5909622	182.611997
Tiparp	9.73308799	6.31120874	9.15804976	11.1553249	41.5769936
Ppapdc1b	10.8538478	6.64407663	9.1479966	18.3432585	36.637008
Bmp7	3.64681538	2.29080113	3.65318397	2.00884917	21.8115691
Cdh13	0.62648785	0.15652065	0.31642785	0.52288029	4.49550883
S100a4	15.8128546	5.64379486	10.528043	5.99897723	26.495254
Vgf	0	0.10028322	0.14191535	0	2.80425733
Rnase10	0	0.24602816	0	0.07191567	2.41671033
Ankrd66	2.17207747	0.38762007	0.04571157	0.45321569	10.0875448
Crispld2	3.34700961	1.41892297	1.26590203	4.18367227	11.8618088
Pcsk5	0.31398738	1.21080881	1.56357433	4.75319776	8.72821103
Ppm1j	0.64453414	0.53586221	0.35107548	1.6359769	11.0287499
Plaur	1.40139734	0.22007713	1.98976302	3.81691372	28.8988123
Snord123	0	1.74561176	2.05857893	0	4.75067615
Pcdh1	1.68794993	2.88698476	4.68904397	6.29077782	18.4146532
Sox11	0.17747827	0.64225403	0.48588082	0.3046268	3.66067958
Hamp	0	0	0	0	3.05056108
Slc25a29	3.08792624	1.27750813	0.49193468	1.40224566	19.2373358
Msn	6.86081317	4.4003963	6.80903574	10.695489	25.9926318
Ctnnbip1	14.6006797	6.99998457	7.3016645	18.6891711	46.8760654
Slc22a21	0.36906921	0.44583902	1.89278133	3.04952797	7.00709221
B4galt6	17.2797504	23.9272238	41.452294	49.3451615	129.79783
Snap91	0.0153115	0	0	0.11245793	3.23924948
Rnf149	88.199212	82.6552665	221.692769	144.656636	438.391381
Sdc4	283.326235	308.747156	304.502318	410.254104	863.798451
S100a16	135.709152	60.9050139	37.3351067	86.1651023	246.06801
1810011O10Rik	10.9257752	4.77037703	10.6161426	9.31107972	39.5758807
Rbp7	0.43913596	0.3448122	3.15140479	3.52768139	28.7679834
Tfap2a	0	0	0	0	2.19071878
Plekha2	14.5990462	5.66999911	7.35279521	15.3847953	30.3585837
Chac1	4.1536108	0.84797446	0.38461772	3.85149622	15.8769413
Irx3	0	0	0	0	2.12302752
Stx1a	2.39308952	2.86805164	1.04966652	4.94337739	8.68013451
Mamld1	2.45227644	0.37029658	0.10480471	0.72737382	8.08728064
Mir28b	0	0.64005764	0	0	2.61287189
Trim62	1.01956988	1.32895021	0.71752011	2.09675322	8.33373772
Pgc	0	0	0	0.12977556	5.09736567
Plod2	2.40743774	1.98692106	2.22924887	3.32341076	10.6598977
Osbp2	0	0.03749422	0.04421649	0.14613081	1.93876752
Olfr1372‐ps1	0.44261328	0	0.0683088	0.23704104	4.81785201
Pim1	0.89280123	0	0.82671966	0.75136078	3.7262862
Gdap10	0.56035823	0.54999583	0.60536323	2.74376422	5.61304379
Sfn	42.1268323	4.50778353	24.9326708	20.1916722	116.372422
Saa3	0.36842762	0.09643053	0	0	2.16509158
Clps	0	0	0	0	2.4330386
Prl8a2	0	0	0	0	4.9331939
Zfp36	22.1313326	37.4533448	62.5745791	61.7693443	112.27207
Mmd	4.21511109	1.96274723	3.97120325	8.90959712	30.9891908
Fgl2	6.85163944	2.94810313	8.74773155	3.19284382	39.1549493
Apod	0.31520151	0.3299975	0.48645259	0.7395302	2.91878277
Btbd17	0.2885473	0	0.98860369	0.63578541	3.42216849
Rptn	0	0	0	0	1.96627117
Loxl1	1.07448859	0.44485732	0.36180337	0.55601066	4.50873039
Mak	0.0731688	0.44525749	0.20325941	1.54502691	6.28848269
Ddit4	12.4618184	11.2741311	2.329391	6.1113379	22.9188406
Myo1c	8.46725281	5.85143801	7.87258655	25.7250534	55.5853481
9130409I23Rik	0.17179054	0.67445484	2.38612943	0.69395966	22.1915146
Gch1	1.65152615	0.64839414	0.65540863	1.23465202	23.217125
Vegfa	26.8408439	12.5005987	19.703964	39.3442361	116.997066
Fosl1	0	0	0	0.28091398	3.55534474
Mcfd2	41.3055141	34.0763876	48.1487285	78.5544314	137.5232
Errfi1	28.4571886	27.0614166	40.4767458	56.4378928	127.163228
Itgav	16.9824034	11.0335993	20.2453193	24.6897118	110.493292
Khdc3	0.03867835	0.06074094	0.14326212	0.56815936	5.57908588
Satb1	0.30742105	0.59592874	0.58712564	0.16757897	3.91853798
Rbm38	1.65418604	1.27001291	2.31464418	3.94857145	8.10078657
Pgpep1	13.1037828	7.26849566	6.07313113	5.84962949	24.5980319
Frmd6	2.43389832	1.06538982	2.11127243	2.31157501	7.83525289
Nptx2	0	0.08028947	0.85215968	1.92447002	8.84955433
1190003J15Rik	61.2506832	6.22041207	59.4903897	67.4976479	272.822534
Trim40	0.11872861	0	0	0.0545014	4.6144483
Kit	0.50346798	1.39352446	3.42659423	6.96805347	18.4378684
Ivl	2.74539857	0	0	1.43139837	15.3458692
Ier3	42.8961297	37.4874128	113.568086	99.8562232	764.684524
Traf6	4.3244142	4.08776311	3.96021347	5.81854764	13.6801075
Man1a	2.71645505	2.11981035	2.32906848	5.55746855	10.2794908
Serinc2	30.4645654	7.76985399	7.30027395	30.7093315	125.079867
Spsb1	1.9431119	3.16885121	9.72803247	13.8010262	38.4487435
Itga5	1.98606703	1.2897887	1.38275663	3.01611047	12.8519551
4930539E08Rik	0.15614466	0.16718963	0	1.22502406	6.43833473
Mir1931	0.54799739	0	3.04462094	0	3.95224319
Kdelr3	5.67258686	3.98338562	5.16731459	13.506693	27.2004768
Gapdh	1.69381012	1.74561176	2.20562029	4.56798744	11.6646066
Rbms1	8.83141625	6.44873256	10.3900885	13.4349617	27.6415691
Slc25a15	4.43552736	3.70510937	3.9324518	13.2216069	34.0921171
Tmem243	36.5660688	7.82580601	14.1794695	41.3271242	94.5711524
Egflam	1.7469854	1.67181235	4.1326683	5.66369743	26.0521592
Kcnk12	0	0.4459418	0.03093493	0	2.78420775
Col5a3	0.27859085	0.3028863	0.29765847	0.37381731	4.36194189
Tnfaip8l1	16.0202924	5.23954165	2.32348134	3.13905045	23.6428999
Fxyd5	3.50447714	3.49544915	5.08689754	5.78263068	18.8233036
Pqlc1	7.92028535	4.92473452	11.6774082	12.0576237	28.1799253
Socs2	30.7447254	23.552603	25.4252556	68.0358727	286.001974
Gm129	0.65445423	1.32140933	1.68818229	0.94418311	11.7251455
Mettl7b	0	0	0	0	7.0698734
B4galnt4	1.21812496	0.01649102	0.29171489	2.00530063	5.53710048
Erdr1	1.09105787	0.79080481	1.47659544	2.23452251	6.65828357
Efnb1	5.24415674	1.32019256	1.946109	3.56499073	12.6232669
Gm12169	0	0	0	0	6.31571372
Col4a2	2.34421106	1.71319506	2.53718412	5.02175822	13.3693165
1700016D06Rik	0.36142662	0.36487847	1.00402583	0.09480569	4.63407207
Gchfr	6.22051093	0.48843827	1.2480203	0.190365	8.14185834
Mir1951	0	0	0	0	1.76148666
Lrrc8d	9.2560531	6.71915717	10.4116997	10.9359683	27.5412502
Ihh	73.6166332	86.6330068	213.15108	307.849299	799.662812
Nrg1	0.46513331	0.43827057	0.02871373	0.37009382	4.79585615
Fam219a	0.46381003	0.04855819	0.34358454	0.3122654	2.30438203
Gm10872	0	0	0	0.06165787	3.01381721
Bmp1	4.86086235	1.58649223	2.22342547	3.80403039	9.48002013
Slc41a2	1.1894517	0.54870423	0.92243361	0.95551425	4.45606058
Rdm1	11.5219196	7.7413165	9.84418195	21.5378581	68.7247177
Scnn1b	0.98493244	2.15825549	1.86648346	0.29440713	6.11230304
Lphn2	1.9287661	1.24373396	1.93567193	5.32219903	10.7761537
Tmem106a	5.0446244	3.29347349	4.61875569	6.8169864	16.2381869
Cthrc1	20.9528968	3.16729556	1.29692831	1.0286906	23.8390889
2610019F03Rik	1.27087337	0	0.53937053	0.29169205	7.74420007
Steap1	20.8613053	0.757963	1.04283275	9.89624042	23.1634531
Myo1e	5.15731606	4.67097083	5.71605413	5.61884787	28.1369942
Actg2	0.09918128	0	0	0.18211344	2.06645381
Parvb	0.51042888	0.23494694	0.09778945	0.93723292	4.24547605
Sik1	1.51856895	1.61256483	3.29445677	6.33353097	17.5118626
Gm3776	0	0	0	0	3.70704936
Slc23a2	1.12716622	0.5793105	0.41749516	0.79964368	9.05718947
Gja1	19.4689972	17.9587188	48.4052239	69.9155777	216.11522
Aldoa	160.13331	106.058675	155.89522	387.709426	882.875658
Ccdc71l	10.013251	4.83085625	8.83611771	9.69732143	23.9340322
Nabp1	5.10112582	3.28373478	3.53203207	3.56518498	15.8355872
Il1rn	1.69632786	0.38056196	0.02243961	0.5784521	9.04941136
Gsta1	0	0	0	0	6.00869493
Cd63	107.128981	63.5968215	133.688726	226.728586	396.964292
Aldh1l2	0.54072711	0.22927438	0.42059192	0.22835908	2.94652219
Mir3076	0	0	0	0	1.74191459
Gtpbp2	13.917397	12.7234655	15.9615057	28.8852574	48.8922419
Fbxo15	0	0	0.07614752	0	7.34767251
Nfatc4	0.97355098	0.52236603	0.88646776	1.32580531	6.96939204
Tmprss4	29.3409455	16.1948419	9.66905535	14.7099579	181.460322
Zdhhc14	1.48636596	0.75615157	0.15508182	2.88297558	8.11961472
Pramel7	0	0	0	0	3.03430283
Cited1	0.25758403	0	0.03975312	0.0788279	3.33704507
Baz1a	8.35857875	3.27331632	7.04605471	5.79416347	18.5298666
Hephl1	0	0	0.02295571	0.15931897	3.40701409
Homer3	1.1202008	0.39790905	0.59273602	1.64060373	4.87308622
Smad7	9.99981098	8.04792138	4.5822425	10.690553	24.9108299
Btg1	60.3676902	119.330875	187.581702	208.336837	386.932517
Niacr1	0.40192105	0	0.03101437	0.30749765	4.938556
Muc5ac	0.00766745	0	0	0	4.41776575
Lrg1	0.24098924	0.15138097	2.32078275	2.38948175	16.4535613
E2f8	4.89396857	0.48554901	0.58896217	0.69748065	6.58350272
Ptprn	0	0.01448504	0	0	5.44009535
Net1	15.3521367	11.945003	10.9526532	11.7080841	63.693121
Mapk6	23.5973464	15.2696449	16.3133403	62.6360538	197.634624
Odc1	29.3158165	18.2141257	12.5416786	18.9693454	84.6541767
Tgif1	48.2381159	37.4747288	53.2941502	142.465031	246.942776
Lrrfip1	13.4102395	13.7128143	13.763528	15.1368517	53.1491718
Junb	119.365745	131.072674	236.124971	232.678744	537.910799
Tns1	0.88336124	0.19010341	0.37566415	2.2948285	7.56647427
Dsg3	0.11112021	0.08725226	0.32338598	2.17151877	9.33713712
Tubb6	16.1159623	13.6526057	15.6502362	24.3392678	70.2059498
2310039L15Rik	0.20315168	0	0	0.18651026	2.44193634
Fgfrl1	6.85017371	3.18100938	5.57015083	11.1128795	23.846391
5730420D15Rik	0.11034127	0	0	0.10130253	3.27161624
Phlda3	20.7148244	12.1117929	11.6077471	14.8552178	95.03943
Socs3	1.38850147	3.27077915	31.6112278	9.4947689	53.0823532
B3gnt2	18.0037951	11.8653695	14.7426784	31.5707992	58.7849814
Pdlim7	7.29183271	1.62853636	2.83023016	5.47186255	22.2710808
Gata3	0.10141787	0.09556071	0.15025812	0.42830645	4.25861545
Glipr2	14.4053559	9.30762379	13.8475714	9.42971476	35.7959566
Pkm	138.853978	97.3458967	79.0650611	163.668672	503.151259
Cxcr7	5.70848339	3.17958778	5.88486673	4.3372683	41.0578057
Pi15	0.13187399	0.85797125	0.56695419	0.1729591	4.18180276
Trh	0	0	0	0	2.91370217
Tspan33	6.29148	1.33304502	2.83584398	9.53515842	37.479173
Dmkn	0.09040438	0.04732404	1.00455608	0.11066505	12.0984179
Insig1	9.55518714	5.47239617	2.91891152	6.73761263	19.6754779
Mmp11	6.29770211	2.5066788	1.63928968	15.773439	33.3033604
Fbxw21	0	0	0	0	7.42013416
Rrbp1	14.0342502	9.96449398	9.44926401	18.9993506	35.9788122
Akap2	0.26582142	0.21617903	0.35163769	0.35735355	2.784426
Hyal1	0.2098767	1.20065986	2.41539928	2.42231805	7.01571118
Pde3b	5.53916924	4.37844957	12.1775238	9.44597185	30.8048006
Dusp9	0.31398221	0	0	0.33260996	4.16124041
Egr2	0.13196295	0	0.57024604	0.04038435	9.90511973
Pls1	20.1967499	17.6591506	18.6692006	39.7566517	83.8352348
Gpr153	0.55249512	0.31180992	1.42289208	1.98139373	6.89017844
Prex1	2.13710591	1.32520358	0.92473173	0.81598952	6.60220308
Slc27a4	6.4593415	3.74325129	3.84185889	10.9193659	22.1390043
Ovol1	14.4590057	12.200823	10.827652	26.3633536	89.6665552
Mir1958	0	0	0	0	2.9861393
Inf2	0.49606806	0.54532188	0.64309153	1.67859232	7.64382864
Gm13889	1.01025081	0.39662751	2.10482122	1.15936857	5.99078294
Xdh	2.9144095	2.67911637	1.32960174	6.82622665	18.3419391
Xpr1	4.0570859	4.01552306	5.31949783	7.73901778	24.0762603
Slc16a6	7.58006329	3.44165867	2.37092764	5.83026598	41.5810623
9430020K01Rik	0.46503656	0.31950582	0.32296232	0.83609575	3.57130016
Fam89a	0.69426894	0.23363321	0.73472222	1.95771946	4.05342863
Mageh1	52.9436821	23.7709534	10.0209698	40.427099	74.8724233
Insm1	0	0	0.27974512	0	3.21637439
Inhbb	0	0.01203399	0	1.3929752	13.0551484
Lhfp	9.30257706	5.80923549	6.56169982	11.0912445	26.6665527
Havcr1	0	0	0	0	2.59045449
Bmp2	0.64822753	0.93654558	1.46460593	3.33271212	14.9603798
Wnt7a	56.033596	21.4266148	40.1172267	63.753035	126.776939
Mir3092	0	0.53338137	0	0	2.17739324
Mgarp	0	0.07166496	0	0	5.81450846
Fam3b	1.14944224	0.18050979	0.35478838	0	3.74583278
Pyy	0	0	0	0	5.86456215
Nppb	0	0	0	0	2.07689817
4833418N02Rik	0.29835414	0.53547306	0.27627116	1.36957042	3.31305324
Fam135a	6.63511547	3.51551686	4.40725399	7.74872685	15.7168264
Bex1	2.40966982	0.76866045	1.11565787	2.00487759	12.4307531
Itga2	0.6338737	0	0.10021216	0.51097974	2.79994806
Kcnc2	1.33665019	0.66939534	0.47754424	3.53653069	10.90524
Krt14	0.1571366	0	0	0.14426456	3.46284226
Elovl1	41.0313047	30.9540483	29.399621	57.888807	185.920107
Ldha	435.465417	338.587177	597.686172	348.609705	1726.36372
Btg2	45.180723	75.3536563	112.093173	180.71628	406.024866
Procr	1.99450646	0.64686677	0.76284219	2.82630006	6.87963608
Gm16516	3.44844767	1.85904667	4.86130084	6.42643395	12.5384855
Col4a1	5.68815493	4.86114831	5.92439204	9.70225516	23.6047202
Nedd9	11.8604452	2.76018067	4.83131117	5.98443812	18.3876977
Spink3	0	0.97070354	2.86184749	2.4118163	10.0304561
Tex19.1	0	0	0	0	7.36231871
Lrrc59	60.6609457	39.3244049	61.3407156	74.837241	163.238701
Adamts1	1.107998	0.40879833	1.29793718	1.49521284	4.16133946
Psca	0	0	0	0.20982404	6.95951857
Gml	0	0	0	0	3.03958251
Ripk3	4.81205852	1.35503298	1.41359246	7.73889433	49.7044026
Tmem125	17.1259485	18.45236	21.4363443	28.9381383	109.229832
Pdcd6	98.1170631	62.8634043	70.7265046	143.467509	239.478967
Fgfr4	0	0	0	0.22836539	2.47500261
Bcl3	3.76699678	6.16687692	36.1322672	20.1306061	64.8163096
6030408B16Rik	2.48646503	2.42096028	4.20576642	3.5002675	10.7862327
Nefl	0.03258955	0	0	0	1.64528664
Sc4mol	22.5595264	12.5484986	24.6307164	43.261177	160.554101
Fndc4	4.6826015	2.95497978	5.61287719	3.95615371	15.3549387
Ecel1	0	0	0	0	2.09184702
Skil	6.88478919	4.15955944	4.12764596	9.15924795	20.2565981
Gys1	4.30492816	3.68628689	2.72314779	5.62753282	23.5662447
Gjb5	2.44926534	0.93153929	5.17383062	1.0891805	12.6350143
Trim10	0.11619009	0	0.10759017	0.1333403	11.2661915
Rcan3	2.48634059	1.50720804	1.87286491	3.02778881	10.6228573
Rab31	3.41442103	2.50425315	2.39733243	7.40621717	13.2447649
Bean1	0	0.09060091	0.24930397	0.17655498	5.73275341
Pcgf5	8.25409722	4.03800449	7.8032283	7.77632829	24.1720951
Tagln	2.0969711	0.35513917	2.77938442	3.01991389	6.32624719
Slc22a5	13.4203767	15.6994493	42.4347925	20.9206844	158.303852
Amn	0.1161039	0	0	0	2.20194545
Pou5f1	0	0	0	0	2.46258196
Nab2	3.11496614	1.09369073	2.88440885	3.65031358	31.0500504
Acta2	0.53244381	0.53752897	1.38519205	2.63036026	5.52644753
Edn1	24.3152801	22.3255602	38.1270364	16.3211594	270.673017
Wipf1	3.81155429	2.48045992	5.21862478	11.2468612	22.117601
Cdk17	7.46452773	3.2890143	6.16613047	7.00096946	24.3571587
Dusp5	1.69381012	0.31033098	2.45722438	3.47296633	15.4736309
Fermt2	8.12381208	3.93152892	10.4272556	16.9658771	34.8866336
Igf2bp2	12.8449801	12.6074448	10.3610036	19.7928092	37.2999357
Gjb4	9.83816493	1.06307152	6.81159313	2.15448389	16.9972289
Timd2	3.68068342	3.66179551	4.12202448	6.05067055	47.0687559
Tns4	0.28800115	0.18306591	0.21588742	0.13850005	9.08872786
Ptrh1	1.2856631	1.00951041	3.76992769	5.96730684	24.1540402
Nipal1	0.12406505	0.23136448	0.25848506	0.95393011	15.0247901
Sparc	22.465271	19.310308	21.8268465	33.4635423	81.2388353
Gm16065	0	0	0.04466345	0.39854152	1.8939456
Serpine2	37.8576864	117.758148	79.4260859	30.6778062	255.362136
Plin2	73.9549632	78.8827799	123.170003	108.666688	361.924989
Cxcl5	1.73372468	3.34144897	14.7040168	6.94561946	52.794221
Cdh5	0.78528879	1.92691714	2.59052482	1.99766243	16.1124915
Dnmt3l	0.10378518	0	0.06406895	0.12704465	3.49307011
1700012D01Rik	1.31361677	0.1473514	0.7819638	1.2921538	3.60914678
Cldn6	1.99185772	0.10200122	0.24057762	0.23852507	4.78853015
Fam167a	0.41575339	0	0.14258555	0.07068453	8.61291417
Syt7	0.04680713	0	0	0	3.96094274
Ncf2	3.08976803	1.63712741	0.46521558	1.4529264	8.73641293
S1pr5	0	0	0	0	1.65464432
Ell2	5.02178261	5.55687207	7.14889346	8.84346898	20.2499361
Snord89	5.54993102	1.08945982	5.78154083	5.09530151	11.674534
4930502E18Rik	2.87300045	0.55333329	0.75292995	0.89580737	6.16837585
Pvr	5.64505623	2.37583724	8.00811916	13.4333884	23.7764104
Usp50	0	0.35151907	0.13818074	0.03425045	2.27206251
Mif	148.079974	36.6935574	58.5320133	176.151195	449.659216
Efna5	5.87760807	5.54984381	5.46553555	13.7180263	48.0939339
Sprr2g	446.46467	69.1380239	953.35921	102.449999	790.203098
Plekhf1	12.0468035	1.05102474	2.55638795	5.03075231	18.7040733
Hesx1	0	0.09006968	1.22150799	0.05265593	3.67686457
Ccdc134	5.452909	3.93111199	6.96863362	11.88613	46.8911483
Abhd5	14.5006934	11.7276127	15.0386995	31.0569796	75.9791272
Egr3	0	0	0.12793428	0	2.472633
Mxd1	3.45575785	3.89994359	2.29310058	8.516128	49.2975657
Rab27b	2.92170351	0.81265746	1.15342101	4.97793783	11.3689285
Ppp1r18	2.09925403	1.76932651	1.8012059	3.18268243	14.4919474
Parp16	0.52526532	0.53617394	1.24028759	3.11043225	6.9452092
Acpp	18.1392932	15.300354	14.090167	12.9347697	67.9337918
Tceal8	11.8089022	7.70892158	11.6775029	15.7672355	52.9222906
Mvp	9.74694292	12.7555972	17.9220908	28.1878064	79.1858255
Pttg1ip	51.6005269	49.3132084	53.1291097	106.790445	266.38037
Rcan1	6.11522228	8.0096599	12.8673505	17.4162326	69.7113385
1600014K23Rik	0.07763296	0	0	0	3.79488536
Pkp1	0.13083041	0	0	0.01334592	2.94719834
Gm12070	1.90167746	1.33766604	1.21063451	3.20081517	7.93703489
Fasl	0	0.23828413	1.09279957	0.46434689	2.70203918
Pitpnc1	3.69704067	1.85465522	1.91139864	4.25216953	20.7548438
Rdh10	64.7570547	104.595738	60.9112128	87.3916365	347.335492
1700071M16Rik	0	0	0.12057704	0.40646425	6.53217971
Fam25c	0.35537706	0	0	0	1.99347174
Pxdn	10.5892583	5.62058482	3.69759869	6.97002426	20.5268859
Dcbld1	3.92348016	1.65886014	1.51711029	5.36354177	10.0196079
Vasn	2.53566933	3.43612006	3.14327595	6.28923819	13.4371586
Sertad1	35.7895805	37.4121236	35.7836931	53.4695708	128.488195
Piga	3.21248791	1.59613596	4.38644039	5.39877898	16.187734
Notum	6.94428591	2.5107759	2.36276057	3.08393187	18.0920995
Tnfrsf26	0.15333619	0.06880028	0.20283837	1.56864086	7.1794733
Mapk13	26.2162268	6.41902191	7.87441048	14.7949128	40.6614069
Slc3a2	27.6455236	13.6407146	20.8632359	18.0945216	61.1436754
Pgm1	37.2809475	15.8367204	10.9336778	18.1865501	65.8981659
Galnt18	8.12930188	12.9453212	12.6867089	5.58009719	73.2821777
Tpi1	179.220134	138.166097	178.590187	310.156191	654.311428
Ucn2	0.52065221	0	0	0	10.1177075
Cnn1	0.09845751	0	0.18234016	0.12052299	3.10336067
Cdx1	0	0.02925978	0	0	1.85140636
Krt6b	0	0	0	0	2.24260797
Sulf1	2.11830735	1.06325365	1.44010237	0.87392173	6.10243105
Tfap2c	0.2125544	0.25034849	0.05904659	1.54162765	11.4632515
Slc7a8	2.69964109	1.39625862	0.86820252	6.69342502	18.6670718
Ypel2	8.9263655	9.08772175	5.05055949	17.9536098	53.0569701
Folr1	1.11235291	0.03639276	0.17167017	0.29785967	6.75966856
Pdk1	2.81818086	3.44886034	1.77499169	0.87409664	11.8105675
Slc12a4	1.27632482	1.40304912	1.41822766	4.84332875	9.0414095
Gm13154	0.71509104	0.50715569	0.93984408	0.91064772	3.5676284
Ptx3	0	0.31969581	0.06283557	0.03114974	4.86684774
Btbd11	0.08760596	0.12897887	0.04056086	0.97520905	11.4606572
Manf	87.2346763	35.3279323	46.97218	75.9092522	161.415313
Akr1b8	2.20039443	1.72776297	1.66707006	2.80065748	31.6991052
Sqle	9.37358711	1.90060785	1.91175162	3.72552204	15.1371617
Gnptab	4.87458632	5.01813065	5.9062175	11.2169577	23.8693177
Prr5	3.37608202	1.39522102	10.1683703	9.78797702	19.1658069
Prkch	9.99912573	6.82728154	4.40993354	6.31354178	28.2506875
Bag2	23.749271	11.7643653	11.0788975	28.4010422	92.9692927
Gng4	0.26365912	0	0.02034534	0.04034353	2.58822215
Plekhg4	0	0.02457625	0.01449124	0	6.15752386
Lyve1	1.27571775	0.58923604	0.34743948	0.34447522	3.76846885
4930500J02Rik	1.25297569	0.8944037	0	0.41830423	3.78809374
Medag	0.31744216	0.35608214	2.03662839	1.16575396	21.3317913
Nvl	3.13271842	2.17535931	3.31525392	5.08697586	10.2294788
Klf6	39.4332009	19.1121789	23.58408	46.5527746	101.330109
Tcl1	0	0	0	0.04297903	1.68814408
Hsd17b7	1.38293264	0.22389424	1.21456457	3.14139709	8.79716376
Rpl39l	0.56705817	0	0	0	6.4266911
Ffar4	0.32769693	0	0.99702411	2.83661617	15.5309255
Mir3057	0	0	0	0	1.72277267
Fkbp11	1.94926246	1.18271521	1.80498588	6.18220686	13.9873848
Sowahc	4.71166019	7.16092861	9.48868623	9.8854157	39.6331897
Eva1a	2.54502635	0.86885653	0.40985282	0.98202714	7.35978619
Metrnl	7.42483175	2.80090055	3.49880569	4.63335917	15.2876297
S100a14	9.58820275	1.02876845	0.38602272	1.80429513	26.5343702
Opn3	1.28906828	0	0.49150567	0.62654434	3.03822312
Pmaip1	50.1741786	17.228982	24.072084	25.3329982	262.074791
Serpinb9b	1.82552164	0.34508033	0	0.37244039	13.8161188
Nampt	17.7837025	12.3197372	18.6453738	29.6781423	71.565222
Il1f9	0.1583769	0	0	0.61796386	14.0241575
Fbxw13	0	0	0.24397973	0	4.85624674
Pfkp	1.60329794	0.6294596	0.90895608	2.31308281	20.2422187
Cyp2j6	4.17209697	3.14776611	3.10743301	8.94299825	17.6468788
Thsd1	0.32385038	0.15026184	0.34077304	2.79752756	12.1147604
Vasp	33.1707617	29.3670162	36.4026746	64.3380847	105.977434
Rap2a	6.59619678	6.68900127	7.43956874	9.62910617	28.0201074
Apoc1	1.81143582	1.42235032	0.5591202	0.55434993	6.1934741
Sfrp5	0.30889748	0.0269498	0.09534471	0.12604167	3.93305979
Gas7	0.27205544	0.5981348	0.49544068	1.08233529	3.70620126
Rhoq	17.4973626	3.52792569	4.58527399	7.84320429	42.7491703
P2ry2	0.30432122	0	0.46295153	1.31713544	15.9036802
Lims2	20.8229269	22.8722021	15.4182034	11.6334668	64.0215528
Mpp6	15.8480254	2.76532556	7.55491675	14.6037028	28.6922025
Ppp1r15a	10.2583326	11.5226837	19.4381147	32.796226	72.887142
Apoa1	0	0	0	0.60781516	3.6606733
Igfbp4	57.8887893	30.6087586	45.9755938	43.9315614	176.096942
Sfrp1	0.25356787	0.09369554	0.95301092	1.97192364	8.88089575
Slc15a1	2.58925697	2.2298518	2.4942884	0.36424146	13.9219303
Nsfl1c	32.268709	26.9637282	26.535498	69.8628306	159.666927
Lif	0.58109426	0.21546495	0	0.02963851	3.93224284
Egr1	4.2455527	1.71682129	4.32444533	2.18275286	32.2356264
Rgs4	0.02217331	0	0.36957827	0.44785292	8.36900821
Tnc	2.58091335	1.47123109	2.97672447	1.42506972	30.4417975
Wnt9a	0.74684877	0.47840354	0.43678106	0.50523032	6.52036745
Tgm1	0.21693842	0.10220481	0.0803526	1.03567173	6.6929852
Cd1d1	4.52908099	2.05596378	1.83481226	2.53382737	18.8274219
Cd47	103.779941	68.9683848	51.2041932	98.5317906	221.534404
Gem	2.08030201	2.26870233	4.49476162	4.03199313	14.0773248
Mall	6.36535048	0.3384138	1.1051649	0.15218554	13.4429403
Pspn	1.65092885	0.32407982	0.63697238	2.39984401	6.50461777
Snord83b	0.84690506	0	0	0	2.71467209
Mgat5b	0	0	0.32648204	0.11259011	2.39543967
Cd68	19.925794	9.14368063	9.46510631	13.0668199	34.7346064
Dkkl1	2.38315145	0.90727994	0.93620127	0.13260198	5.96070441
Adamts4	0.31957811	0.12546733	0.70693009	1.64629705	5.23570299
Cgref1	0.24497254	0.38470783	0.77125822	0.53977274	5.69295903
Gm15698	0	0.06313762	1.04240413	0	3.80171248
Tpm2	0.24866231	0.48812785	0.54686113	0.68487847	5.57944044
Fgfbp1	1.04505913	0	0.09677081	0.43175331	3.14047102
Derl3	0.09762229	0.07665361	0.4519834	1.47881973	5.90634214
Rpe65	0	0	0.6880546	0.16242483	3.23241883
C1galt1	18.7898089	26.4825788	31.3475548	16.6417729	65.5432269
Ier5	4.98560318	2.20790527	4.43192529	8.42205036	19.944123
Atp10a	1.01352964	0.72092797	0.76185112	1.69680342	9.07744849
Lrrc8c	0.80679008	0.47059735	0.36286486	0.50790917	9.14366619
AA467197	1.32461244	0.72006485	0.47175767	0.28063965	16.7387105
Cdc42ep2	7.21658536	1.99186282	1.13399027	8.43236518	50.9606401
Nlrp12	0	0	0	1.39682353	3.38001445
Panx1	10.3395884	1.55464604	3.31753636	12.147076	37.9779354
Csrp1	96.4543521	50.741745	99.7205099	96.1975654	258.954794
Chit1	0.22867922	0.20948701	0.63525989	1.64458227	13.4995836
Trim6	0.64751612	0.24083695	0.6469256	0.2346608	3.04505059
Lamc1	9.35140077	9.5462809	18.6573908	25.2298313	77.734824
Mast2	4.7824183	2.88791506	3.4373152	6.31418803	24.3357057
Edem1	7.69226314	4.11699762	4.27787087	8.34988404	22.9797131
Fhl3	2.32207797	0.30388494	0.68089891	1.1369931	4.40389089
Gm17455	0	0	0	0	5.0611054
Map1lc3a	89.61147	105.643199	102.798931	269.786463	613.637338
Il13ra1	19.094549	7.32288474	36.2473759	42.4693727	140.620014
1300002K09Rik	0.09042109	0.05679935	0.20094836	0.86334698	4.41999958
H2‐Q10	0.04427134	0.1390485	0.16397823	0.28451361	2.23504316
Sqrdl	7.98066867	7.97328951	10.6680135	23.1211438	40.6114944
Paqr4	8.29777781	5.12658855	4.52827149	3.65380068	32.8094164
E130012A19Rik	1.14750183	0.08790491	0.31099562	0.92502684	9.3300833
Rab23	2.79042665	1.04988283	1.84371351	4.64334475	8.54846429
Plec	5.31299349	6.51057207	7.01934476	8.93859595	20.5359945
Slc39a10	12.4219001	5.30521474	7.01103601	8.54420748	42.6402152
Prss32	0.17866216	0	0	0.04100671	1.96860211
Bend4	3.13936552	2.67154495	2.60260414	4.43647614	14.5412001
Rell1	26.2054382	7.3330061	24.5267396	30.1566364	67.7895095
Elf5	1.42332864	3.37562695	2.07111074	0.26668059	7.37888987
Eps8	10.6220013	4.62667276	6.22689543	10.4311227	71.0176037
Golm1	1.39499688	1.26174375	1.60241761	3.58278478	8.29213607
Ube2j1	19.6264597	16.3267401	14.5733205	24.3082918	62.0649807
Limk1	0.49060856	0.69341183	0.34526472	0.43240296	3.3810861
Fabp4	1.72948319	0.31952956	0.09420434	0.28020184	14.0222766
Cd1d2	0.05242097	0	0.04854098	0	2.56246278
Bcl2l11	7.89738547	2.14496189	3.20087159	4.84948887	12.7297749
Smurf1	4.02299754	3.40851999	3.78184587	8.72282561	16.5405128
Kctd11	5.1508131	3.81871276	4.2815215	8.8419018	25.4375845
Dio3	0	0	0.32326008	1.50635988	6.29439159
Prr9	0	0	0	0	3.53389266
Tdgf1	0	0	0.31336265	0	6.31862116
Plekho2	1.07787917	1.05010988	0.81326575	1.79591053	5.67841763
Fam115c	1.28677048	0.39693497	1.41848678	2.78464153	22.7467776
Ankrd10	21.2831503	26.6259379	32.2134619	30.5266014	143.484932

**TABLE 9:** Gene list of cluster 9

	D3AM	D4AM	D4AM	D5AM_mesometrial	D5AM_antimesometrial
Plekhs1	327.915397	717.55072	980.295058	37.4562271	6.67442148
Drd4	3.41678014	30.5464636	8.31649453	0.04481272	0.66495243
Ganc	18.3526653	25.5170358	8.86165952	0.90976163	0.67442988
Cdkl1	42.7022668	30.5287412	15.6950901	1.02795973	0.06187974
Pla2g4a	268.133236	269.804763	149.944016	27.7050306	10.6865175
Pex11a	119.666047	142.545716	56.1944429	15.8699223	9.09600451
Fgf18	20.3748966	24.0213339	12.9674662	0.60697486	1.34857904
Hnmt	46.4603429	39.2943814	39.2981629	4.49571704	3.95599651
Slc2a3	39.4169167	113.402888	147.034915	2.08689856	38.859787
Sycp3	24.8176394	35.1860566	30.6768625	4.21543104	0.79177936
Galm	268.021496	257.870529	104.416259	7.28038015	1.09403099
Kif5c	11.9173409	15.4587022	34.1916719	1.83810767	0.91679715
Slc51a	45.5299231	38.7019311	2.26834777	0.62041236	0
Meig1	37.2242364	61.7211394	30.5346156	0.25440422	0
Bcat1	196.496294	124.813611	36.3934667	11.5276323	3.16692198
Ripply3	11.387522	47.7887382	49.287415	0.46987411	0.649373
Lrrc26	11.3998894	6.36345628	3.20185489	0.0992043	0
Capsl	52.8035457	21.5427435	13.9153553	4.55668613	0.44192336
Pdxk	101.171571	54.2302472	9.35291536	7.26335531	1.62086281
Calb1	2159.92398	2558.94466	1539.93288	28.3373941	5.5775291
Ccdc11	7.08419208	10.1534761	4.98433913	0	0
Rnf128	1046.47538	764.57159	1028.32288	191.496095	79.1793781
Wfdc13	46.8139782	125.293966	75.5655641	16.8906399	0.72985248
Fgf1	21.8206421	51.2570082	7.38399115	0.09189531	1.32348077
1600029D21Rik	511.201711	362.426573	290.116146	55.5563137	23.014787
Gcnt3	164.583327	169.257373	40.2238953	7.1548648	18.9298042
Ifi47	54.9747825	34.7214579	69.8041993	13.0862935	1.06435914
Tspan15	12.2825901	9.70117883	12.9480142	0.73072701	0.3334039
Insl6	351.056262	372.164427	243.095299	11.2482641	13.1435374
Gstm1	1589.9255	3057.3585	1704.03246	230.225726	15.9114609
B3gnt5	55.5477241	83.3441234	13.2247077	0.81104399	4.89109023
Aldh1a1	134.274463	5.26661605	3.06085293	0.20624437	0.28288967
Ctf1	75.1025332	142.230802	78.7025082	10.9946757	3.80760513
Pla2g10	206.673838	339.393384	185.409074	5.50661499	11.6582151
Lyz1	22.8885298	35.9849009	15.8480743	1.46716488	0.12393068
Ces1d	80.7351233	88.1886138	18.1830668	0.12181036	0.05316118
Suox	43.7798358	24.0873119	6.7608558	2.01350091	1.00110034
Pla2g5	17.4566677	9.65572675	6.10485533	0.42106228	0
Dync1i1	65.1418697	61.8037103	52.906899	9.6791635	2.21935585
Akr1c19	29.7180485	16.4085631	11.4254998	1.25331289	0
Krt19	713.484228	510.853577	307.506346	30.8499369	103.906346
Ces1e	12.1426494	14.2523207	6.29192288	0.20216524	0
Efemp1	29.1787078	29.2992988	15.4818078	2.91115397	0.92400185
Mab21l3	124.485047	212.848849	69.5496505	8.06004237	1.13092263
Il17rb	80.3043513	206.333964	332.542235	17.4293429	3.16942247
Plgrkt	121.914143	105.264047	53.8665557	11.7182868	5.7650554
Ang4	17.9214859	14.73514	5.82128604	0	0
Isg15	109.37622	64.0734954	112.463035	20.1149833	3.80179772
Gm266	47.8219057	45.262348	16.5996576	4.23769727	0.34408189
Chdh	34.5817889	56.5849637	37.9879042	8.72995281	2.55476864
Cbr2	214.029747	205.821475	128.044551	3.40102643	2.50794755
Plat	336.746778	78.6472094	128.234616	15.0856745	19.6894137
Tmem158	495.917401	1426.69698	1222.43873	12.7478093	4.97546291
Acot7	255.727991	982.082426	498.046242	16.2549706	15.0591326
Mettl20	100.904478	146.160382	94.3292027	31.2456552	4.23625383
Ifit1	74.1109345	120.790863	142.88213	46.6385232	2.37713894
Anpep	77.1185858	88.0037189	85.4351353	6.10416526	5.90815904
Pfkfb3	63.4715593	126.228477	68.5216412	5.22331084	16.8818028
Bcl2l15	18.6630683	13.2109366	6.63571231	0.54349071	1.07361196
Kctd14	59.6009849	127.080354	60.8809088	1.03021935	1.0831616
Arl14	27.7693111	27.7358312	44.332641	0.09978299	0.95805302
Ctf2	4.70973313	12.1753187	3.22053238	0	0
E130008D07Rik	9.20771591	18.5034388	19.7160891	1.30869378	0.69978451
Rab40b	38.9172174	112.12849	34.2235528	4.17322952	1.93338509
Ndufc2	1421.8255	1177.78126	867.734852	209.148659	94.3856804
Pcx	34.9413758	76.4113761	55.9748429	12.0384251	2.69116018
Rims3	17.3541856	32.5990464	20.8288014	2.20605173	1.01626542
St6gal1	25.930951	10.4188043	13.2482353	0.96564182	1.1886514
Gstm2	1881.23579	3772.65992	1791.9402	47.2897026	8.23541282
Ptgs1	118.651576	84.3338522	43.1803224	45.8848447	1.0582861
Acyp2	205.83958	191.769283	144.078027	28.7303626	10.2801517
Gpr82	14.6772005	15.502878	2.46666727	0.2157902	0.16742471
Akr1c13	24.6661098	11.0555411	5.58757139	0.09719122	1.01800203
Dcxr	144.843829	226.125812	247.72314	17.347892	17.077594
Lrpap1	146.933803	305.871372	265.760392	31.1808966	15.3159054
Sla	35.8144587	39.8849504	10.8174067	6.41017532	0.81482491
Slc15a2	21.9860107	24.3325681	16.6152287	1.68446246	1.12140424
Mcoln2	12.3036309	18.8416069	8.60701169	0.85335789	0.88206478
Gstm7	730.670747	208.389036	83.6135345	10.9646594	0.36039612
Eppin	21.9387671	35.334573	30.9523021	1.0308706	0.48450605
Serpina1e	676.672207	597.486585	442.705921	229.057554	20.1722083
Bche	26.2254129	18.176029	5.24198829	0.60747253	0.02524921
Slc31a2	132.961773	204.144701	190.19283	41.7841262	9.99881895
Gm9895	32.9004759	53.0553806	38.19532	2.97545657	3.14803842
Fam189a2	194.841677	385.180059	251.564594	36.0910525	27.8514915
Fxyd4	997.244465	1837.1734	1112.10825	18.2635288	13.0171957
Thrsp	1319.12317	1299.79799	486.453969	11.8239957	0.85585399
Slc25a48	112.052663	143.837787	98.0752078	4.31638025	0.06976961
Slc39a2	26.1237917	20.7538828	13.7638434	0	1.65683393
Ifi27l2a	63.8032297	15.8148152	7.43400427	2.97409338	0.90293629
Rbm24	37.6019016	63.3069422	67.3899396	25.2181075	0.71319084
Ly6f	36.2121925	38.4180552	16.8003827	0.95573215	0.35751953
Atp2b2	19.5960314	21.6793146	16.9158262	0.41472564	0.40543316
Acbd7	374.814851	418.7293	204.631724	4.58815234	0.87909708
Myd88	218.159631	633.216923	447.641321	34.2876346	42.8757875
Pga5	15.7042275	15.6217459	2.47339616	0.12684278	0.44285964
Nipsnap1	119.531565	170.335938	92.4463786	21.6604693	6.66463586
AU021092	48.3859329	43.5910979	38.2467561	3.70913439	1.63780599
Chpt1	193.95268	318.441344	270.006375	28.2762346	10.0041357
Stx18	120.153164	154.367808	343.67637	29.6665939	13.9509293
4833423E24Rik	59.4838972	99.3399852	57.2671818	0.74615172	0.24810653
Ank	58.1980753	106.056233	67.6740159	3.04829037	10.3588113
BC048679	417.921871	122.668439	41.3943428	1.21474942	0.908825
Spink8	270.354795	104.143694	48.2901992	0.08817348	0.30784941
H60c	18.8898611	10.0155275	4.86595347	0.23378336	0
Rln1	8.0462056	8.37492929	6.06448887	0	0.29989921
Npl	733.782568	1135.33361	567.136784	3.28579761	2.34655636
Slc16a12	17.423485	44.9859636	14.3082761	0.21658323	0.04050964
Plscr2	21.7944331	12.396906	13.8567643	2.52083338	0.46756655
Ano2	8.48565657	14.0170505	3.98261022	0.03049136	0.02661443
Sptlc3	12.8816132	17.3342179	6.01880031	0.28209761	0
Il6	39.414977	11.2113133	14.8323737	0	1.87492187
Rasd1	94.5954527	111.447203	120.657376	3.42325726	1.4939976
Tsku	17.7468955	28.8940308	4.39947726	0.98357517	0.89584179
Sbspon	68.7000878	106.527207	80.8429217	6.42707019	0.96984328
Crip1	123.566669	72.1675733	56.4673881	11.65251	4.32555972
Gpr30	14.3730826	34.1636189	20.746056	0.47724028	0.49987186
Irf7	100.85186	104.17205	80.8814445	14.4280082	2.83916149
Alox12e	35.2747511	83.2047217	74.8470414	14.2608861	2.62531952
Lpar3	121.062599	251.875772	240.547232	26.4540945	15.0234895
Lrp2	36.2928961	63.1855703	63.64492	7.95064036	0.85141749
Oasl2	46.3094492	35.1052024	44.3259658	11.3783152	1.06647832
Ovgp1	49.9999331	66.6775298	61.2459164	5.90399145	1.42802503
1810007I06Rik	13.5939776	24.2873837	14.5945576	0.18087551	0.31575491

**TABLE 10:** Gene list of cluster 10

	D3AM	D4AM	D4AM	D5AM_mesometrial	D5AM_antimesometrial
Rgs19	15.9784305	6.85305371	9.82240271	23.4219505	17.5387008
Snora7a	8.59378854	2.77854481	3.27670445	10.2103523	5.67134983
Stambpl1	1.21983444	0.98370841	1.00743398	5.32714031	3.98931906
Rcsd1	5.32440012	2.51228618	3.14363764	14.4853506	5.75418977
Zfp422	35.6243321	19.7052818	26.6151817	79.42915	45.8841767
Elovl7	4.36583686	0.58390398	2.99870244	11.7272999	15.5801557
Mirlet7f‐1	0.73271561	0.57533271	2.03544883	3.3634715	4.69729777
Atg9b	0.51808364	0.02624532	0.2011801	9.00655263	3.68293064
Snora33	9.93701936	1.95065187	10.9268063	25.0882941	8.95841789
Efemp2	4.24831854	2.55745074	5.11403757	8.3206661	12.2274461
Sbno2	4.25843576	8.38696223	18.3367327	19.2125262	28.2009966
Fam199x	7.1601602	5.25191327	8.6752921	14.3090031	13.7870955
Lepr	0.87486288	0.1852443	0.32768456	2.77960449	0.7325809
Pdk4	0.07554207	0.02965804	0.01748769	3.86648242	3.20839177
2310042D19Rik	0.61104134	0.50644853	0.26719019	5.45404151	4.71976337
Nt5dc2	2.54371839	1.08945982	1.1991344	7.43064804	4.29919346
Snora65	22.1056575	7.81087295	11.7699541	35.0086076	24.8001399
Mir3079	0	0	0	9.57916685	0
Gar1	31.4514286	8.50052953	12.6376123	34.9044189	21.7910637
Gm2a	30.2615336	8.95118209	7.27854694	45.8870451	53.485291
Plekhn1	0.82621145	0.9267803	0.49182338	4.95754393	3.52323901
Myc	23.6219084	5.72023172	14.1208733	55.1281252	23.8523945
Tmem180	7.17900617	5.97387135	6.38279854	20.166667	14.8750338
Igsf11	1.93035775	0.58528132	0.10618696	3.38653869	6.14162735
Snrnp35	8.718779	6.14267776	7.18868834	16.3929195	12.2029731
Fam117a	6.36074313	7.69063652	2.606171	26.8729325	7.46534824
Fam110c	8.37738184	5.12046115	13.2078013	41.6846235	36.1104766
8‐Sep	5.28514882	2.95475065	4.3980417	12.9909816	11.3166096
Ankrd44	0.91338849	0.38361761	0.35404876	2.95448652	0.88514227
Ptgdr	0	0	0.11368368	5.22240427	1.06580905
Itprip	1.62769485	1.67237579	1.23463718	5.45282499	9.51901282
Blnk	14.9890483	5.95799963	5.70158629	42.7967665	57.6401781
Sh3d19	5.28770912	3.5211654	2.24101446	7.78596713	11.5629665
Fuom	74.3413261	23.1602397	20.159294	247.400405	66.8091242
Il7	1.7916747	1.79991968	3.95247155	8.3938659	3.3782586
F2r	8.68565889	8.26482201	13.6305906	37.6729218	25.954316
Oas2	8.84340717	3.39243803	7.42310209	18.267724	9.18291931
Jag1	3.94143108	3.86854429	13.3675839	16.2508394	8.638222
Cyp2s1	0.52248969	0.03907258	0.06911673	10.781588	1.63491411
Ang	2.35273547	0.76304911	1.70498773	5.72871743	8.93499719
Rin1	2.17059982	1.42235032	2.67510098	8.50162527	10.4489848
Mad2l1bp	32.5033107	26.1658785	33.8478712	67.6359216	67.0883615
Slc43a3	1.12822992	0.59059529	1.13758713	4.00513031	4.90227405
Zfp597	2.71039704	2.80124695	4.06499257	8.57105737	6.62731981
Slc12a2	47.2584713	60.2518067	51.9848013	184.751569	77.3682586
Mmp7	17.5284268	0.75815484	3.78265833	26.5254549	9.16588315
Serpina3n	0	0	0	3.53188706	1.50381116
Snx18	7.00948676	6.28687158	8.6768616	21.6349802	15.5467934
Nipa1	1.79894316	0.84209176	0.76882736	4.38304053	1.88514895
Anxa7	23.929281	22.7096338	39.489538	72.0254809	99.1921954
Prr18	0.55061939	0.08646993	0.16995492	1.73560052	1.61787733
Pam	19.9624377	11.5063738	24.6072849	56.163498	32.4523401
Dennd5a	3.82978843	2.74882476	5.17933751	10.4636207	11.1791895
Adam28	1.82315881	1.37853471	2.23532292	12.7395728	11.8096669
Tmbim6	136.123295	157.577945	121.416254	326.539205	243.986117
Neu1	30.2175725	18.46026	20.7191068	78.4372038	77.1509807
Ipmk	8.35460819	9.90630107	16.9773542	39.6663626	52.4021417
Snord96a	11.8566708	22.6098285	8.62642601	32.6562506	41.3987493
9530026P05Rik	0.32769693	0.66900498	3.15579806	3.48989747	1.83820133
Trp53	52.8884955	21.819524	22.5958793	65.2221779	34.1295899
Slc25a37	5.59360239	4.29370721	3.38051437	12.0257441	11.4007096
Irak2	6.95564158	7.46366874	6.27082078	30.0378942	11.5074245
Lgmn	27.5253183	22.4600804	65.4116935	168.523563	149.633624
Large	3.55473914	2.65164246	2.9460103	12.5809785	9.25792709
Mxra8	3.22744553	2.17217972	1.41423069	5.37055587	7.34328996
Mmp2	14.9465793	7.35824243	14.4558589	37.7606914	16.9608981
Tfrc	21.5912533	2.70923871	6.83232601	34.9301376	45.0047774
Cpeb1	2.70144764	0.97015802	1.84218796	5.71012475	2.04733699
Gabarapl1	26.187661	16.0862767	18.3232489	113.222456	32.215104
Snord12	16.7969503	6.20661958	20.1283274	47.1701398	21.3780427
Smyd2	14.5456603	6.38063695	10.9483052	28.3495592	16.6051671
Mogs	21.3527085	14.9177334	16.2843255	44.0486057	33.4862642
Snhg9	1.42539212	0.27980662	0.32997258	2.29010136	1.14223908
Tmcc3	23.5422061	11.5121253	13.6991571	39.8725297	49.9325186
Ftx	1.14910466	0.59468712	0.54008728	3.38071317	3.71822377
Aprt	60.844163	24.7153899	31.4476061	83.3061204	105.420027
Igfbp1	0	0	0.15890785	1.89062504	0.27503915
1700020L24Rik	0.14008956	0.10999917	1.10262588	3.92272541	0.78582613
Pank1	4.27443902	1.53946692	0.60515827	6.98913345	3.60933007
Fdft1	14.7825311	8.7099706	11.8323349	22.6890413	28.7114738
Mfsd5	22.685582	20.7522708	23.2249931	48.3563711	45.1477863
Capg	13.7771175	11.3520918	18.3643425	38.2579531	37.7823728
Slc20a2	9.50186728	5.15125671	6.88368986	17.9052907	10.7857751
Pusl1	3.88291463	1.87945148	3.25074128	7.56918261	3.79356603
Sema3c	57.4994695	15.1288701	17.9144145	81.9328082	111.116925
Gipc1	66.3819488	54.9752481	61.6554581	141.820277	129.867494
Cdkn2b	5.57084785	3.1612323	2.68763021	11.1315695	5.73968986
Vpreb3	0	0	0	4.17696229	3.99309325
2010204K13Rik	4.13604796	1.13667742	2.29794858	6.51859266	2.76202102
Ankrd13b	1.79840391	0.85954983	0.36202028	2.92529263	1.75444635
Raph1	4.54200097	3.92023899	5.75568163	12.9760569	15.7454405
Erbb3	3.48768986	3.45185317	2.68878403	13.6568657	6.73366983
Unc119	9.01051448	7.35491688	10.22915	22.8075401	14.4819597
BC048546	1.81837619	0.16348854	0	2.7271468	3.49272785
Pmm1	22.3688201	19.9197924	22.4676971	68.0360518	72.7976896
Nr1d1	3.12777803	2.35849181	5.51670944	15.8163823	7.49944957
Nptn	103.170733	50.6375889	69.1360074	168.77113	131.184536
Limk2	9.4193227	13.6863525	21.0575725	51.6847012	27.3453325
Dusp6	16.1805193	17.2817852	31.455459	45.913731	66.6810136
Nek6	1.87344096	1.48668646	3.21118181	14.3453293	11.2915674
Mppe1	5.8160899	5.79145332	7.04040149	14.5871095	12.2897412
Fxyd3	0.74669874	1.07490597	0.46095406	2.62787258	3.49047771
Comt	23.9652959	18.3056486	23.0720952	83.318795	62.796021
Ldlrap1	8.03243948	5.04186179	5.92319929	27.5253109	28.9166952
Fam46a	2.5705607	1.12547829	3.52108615	12.0500873	2.80984588
Rnf185	6.8464313	10.5909783	10.9522814	21.0703247	17.37054
Rhbdf2	6.00219466	3.68444065	6.03389218	17.3078959	18.6016814
Tcf4	10.7907899	9.43295473	12.7436605	36.4861997	34.0105365
Mafk	7.73306761	7.61272101	11.7563682	23.3121317	24.7506024
Apbb1	3.26469101	3.38569535	9.54447764	13.1389312	6.30182577
Prkaa1	26.7290878	21.0978399	31.1718822	88.9236836	79.4245697
Myo1d	6.03022349	5.19411621	10.4683847	42.2097138	32.4107404
Abcb1b	0.18014279	0.02357487	0.18071012	1.59873296	0.86614538
Anxa3	39.3230163	8.17606508	17.4311063	76.1399807	40.2483709
Ctsl	55.8484688	33.6428067	45.434261	116.823553	166.97988
Itga3	9.84961229	5.42327845	4.3425425	14.7546409	23.4205496
Tmem2	3.19847345	2.60362432	4.30221486	8.66571922	8.5515021
Trpv6	11.0913324	7.25748422	17.8803892	73.6512956	45.6360206
Glipr1	5.36895501	1.563138	2.6812943	9.30446363	6.6228205
Slc39a13	7.60277067	2.74833375	3.00198107	10.7728752	12.2539438
Ppm1a	33.693096	39.6014152	40.0329264	94.4306986	68.6148214
Eda	1.25039045	4.12762668	4.46596032	9.27741653	6.20627366
1810008I18Rik	0	0	0.34955127	3.72561663	0.37812907
Mb21d2	1.96905426	0.49874622	0.8626426	4.52911095	5.37844408
Zfand4	3.57571	3.27224736	4.40679356	10.0137247	12.2655524
Ttc5	19.4046281	15.3207338	16.1500486	53.4288323	35.3199052
Syt11	6.11136482	1.95086543	2.02296962	8.16704202	6.91120919
Sat1	980.573262	401.508447	664.668058	1259.57453	1526.05778
Gpatch4	9.11561252	3.88308882	7.10813597	15.3825048	7.92585925
Disp1	2.29472546	1.99524928	3.49344528	7.2843565	6.58672276
Cyba	89.4405386	43.8511166	46.7009018	140.248342	84.2040136
Mtus1	10.9284806	10.1675916	15.8071951	30.5367271	26.3302072
Atf6	4.92823166	2.36708977	3.33359408	8.15859296	10.2932378
Runx2	0.44216483	0.09577669	0.31060781	1.66577165	0.87971371
Vps37d	7.22227909	4.21177828	3.55895943	39.9778215	5.44912401
Dpagt1	16.0205096	8.35938802	11.4430333	24.0107725	20.409204
Ifitm5	1.47163192	0.36110445	0.51101536	3.12437565	2.57970426
Mybbp1a	8.10988221	4.31576395	6.33624295	15.4000754	14.0385538
Prom1	41.2038798	19.125365	42.6826401	93.5729729	36.7977867
Ptpn9	10.7560701	6.71618672	7.87565471	20.945571	16.7878307
Ddr1	20.6411439	22.1256072	34.9406861	75.8609756	59.5364318
Id3	407.692808	37.9870518	29.1470008	306.259433	230.579775
Ndfip1	119.218382	109.731974	98.5364328	329.443598	446.837344
Dusp7	8.79099053	3.77817339	3.81636094	16.2722071	28.3088237
Ppdpf	51.2202229	60.3918881	69.7041198	131.232783	82.8119747
Tmem132a	12.2797593	4.37345206	2.78646664	23.3198763	9.19635046
Diap2	2.37948115	1.08584593	1.12314207	3.96483997	2.37111701
Snord55	5.85233111	2.62587751	0	13.816106	6.6996715
Arhgef37	0.10082203	1.04499207	0.70953288	9.81168527	2.97321229
Gas5	32.5803116	17.4825455	33.6444125	85.3273476	44.9119326
Ephb6	0.97123793	0.29960145	0.35331638	2.67503329	0.8894883
Slc1a1	46.7455962	113.732789	167.084659	273.014651	207.479503
Abcc1	7.6583905	8.1099132	8.78049528	21.9102258	25.1205509
Abca3	0.77813422	0.82524091	0.78604347	2.93179212	1.39288382
Snora62	1.01893265	0	0	1.87093102	0.81652246
Arid5b	4.68699949	9.18355961	11.3003493	22.7675736	19.7680561
Mir3068	0.82546442	0.64815964	0	3.78922739	2.64594621
Ptger2	26.9292433	57.8769747	92.5165874	261.612563	173.026484
Mir1247	0	0	0	9.49155251	0.63728583
Snx7	25.5620211	6.17946026	9.95754195	48.1495432	41.2812094
Vamp5	0.791404	0.59034443	1.39237216	4.32312217	1.17325558
D030056L22Rik	70.6493289	58.9590806	114.216842	450.656926	362.533972
Cst3	180.07918	116.072987	117.758853	354.700282	244.595797
Hhat	1.44914866	1.13788026	0.29819744	2.8931787	2.69121196
Cmtm3	8.23216873	6.27383235	6.83815081	16.1159405	13.0643594
Cfc1	0.06282436	0	0	2.076409	0.25172176
Slc2a12	45.2252254	90.7582003	86.2436227	172.861693	146.076346
Trib2	1.14951525	0.64646127	2.54600806	4.76334226	2.21577511
Xrcc6bp1	2.7231255	1.31293876	1.19442822	7.63175381	2.33531407
Mir1904	0	0	0	5.23860687	3.91930783
Fam213a	112.646087	42.1763424	53.9811095	191.061375	165.44222
Prob1	4.23627185	7.99379066	7.01108371	26.8296679	11.994747
Frmd8	17.3430962	13.407651	14.8959795	33.8493143	43.1957405
Slc43a2	0.53200908	0.66554667	0.18369325	5.37271785	2.18943634
Tuft1	8.72371666	3.29514856	7.65410264	51.8606902	26.9643487
C2cd4a	19.0522318	5.98397087	4.24302758	165.306166	24.2734252
Tmem204	0.87975298	1.23766065	1.79899047	10.2643546	2.23247064
Cd164l2	0.13227523	0.5712482	0.12248475	1.82159613	0.79499145
Fgd3	0.41936778	0.37868362	0.31066229	2.86835985	2.89012196
Lifr	7.28921914	6.30828128	16.7661389	65.9825027	42.1427686
Gng12	34.4816337	15.1865155	20.8018859	58.360355	54.0181169
Maml3	0.19026752	0.10222051	0.03709151	3.39249901	1.40433839
Nefh	0.01633559	0.02565361	0.06050599	1.81469061	0.35344459
Slc25a45	11.171015	6.89991219	6.3251045	44.4532395	30.7614704
Mocos	2.44204663	1.2605851	1.82159967	5.6880455	5.09165742
Kremen1	7.04344721	7.81582516	6.28830769	33.4426538	31.5014267
Snx33	2.94338476	4.75695778	2.40799008	13.8393145	4.19067095
Birc6	4.03230577	3.20191191	3.14409375	8.82023901	5.11041153
Agpat6	13.9385026	8.5427572	9.12511392	34.838026	31.9984057
Tmem28	0	0	0.08528952	2.57913655	1.99286839
Crlf3	6.31617044	5.69906891	6.15649774	12.9200742	14.8958117
Ccdc107	13.6992616	3.83720062	7.34411944	16.9165262	8.9877657
Gpr125	4.22600893	1.41885404	2.01058376	5.15080899	8.99178258
Tm4sf1	99.2918259	286.901969	419.742296	1347.76284	521.667702
Igf1r	3.4571233	4.7323013	4.64810097	17.9039571	10.0344053
Rnaseh1	11.3684071	7.83708193	13.4280948	30.2841711	25.4293228
Wipi1	20.5289413	28.2090348	23.5624393	53.8399788	56.4851996
Ppp1r14b	166.612674	56.3863622	88.507694	192.769006	149.550665
Pdia6	121.40115	42.8913922	58.6515779	122.669684	179.54426
2200002D01Rik	23.4093245	12.5234158	27.6317867	96.5946558	121.006375
Rassf1	21.36984	12.9333714	21.800963	62.7930127	72.2771885
Prkab1	33.0668733	21.5703483	37.3615515	84.2438917	57.2768015
Dennd2c	0.17043666	0.06176672	0.81338838	2.58785795	3.86625192
Agpat5	14.084635	16.0206646	16.6851165	90.6724806	54.5775642
Fam210a	3.41157187	1.6125766	3.31545017	10.884853	5.62472576
Mpzl2	29.0167686	5.63632851	34.3420809	71.5980171	40.1680305
Snora61	1.05180144	0.82588083	3.89580529	9.65641819	5.90003329
1300002E11Rik	8.07116137	3.27223206	2.54718161	13.7009427	21.4626405
Map3k6	0.07525005	0.25998187	0.50169844	2.50090757	2.23116224
Ltk	0.87628208	0.14401297	0.2641843	2.22640792	3.21709851
Zfp398	1.29373392	0.92751309	0.98963312	6.09370519	2.14859505
Gnl3	9.48407799	3.85937093	9.42313414	16.7152394	10.2351351
Ninj1	27.5939923	15.6056457	16.4298361	47.8112126	38.8699316
Rras	15.6480481	13.2071091	11.6812386	36.2636271	53.472727
Yars	15.225137	15.7675996	19.4559824	124.280857	47.5117205
Pop1	2.5561906	1.34312126	1.67291138	6.2640072	2.74147478
Eps8l2	6.74456952	3.32454085	5.00417143	24.6901853	6.66644638
Ado	22.2262564	18.8230226	20.2822898	52.1924515	40.9594855
Gabbr1	3.24168675	3.43570844	4.46897326	13.5861456	6.26716484
Csnk1g3	19.4121279	15.4755858	18.2226869	36.052679	38.0097339
Zadh2	4.59486159	4.3325793	13.0734123	18.532414	13.3279283
Mir17hg	4.20990386	1.35728342	3.27870574	10.6736655	12.0869063
Pnpla2	14.1127985	22.3325229	18.9355925	51.0116195	27.0423104
Kif3a	4.97661846	5.09409615	18.7995172	30.6505155	24.9082895
Cdr2	5.55487847	4.50373366	5.3111989	34.6077764	12.4639372
S100pbp	2.69957835	3.64327766	4.2964735	32.1422562	11.729192
Gpr137b	0.19688199	0.51530974	0.42538901	2.8117313	2.68211606
Tpra1	12.6216173	12.5701862	10.4988651	33.0609572	26.5430069
Cyb561	33.6074795	14.3844806	21.6009334	74.4621473	54.9188917
Fam117b	7.71840561	4.92245272	7.46355759	22.3619194	18.6121087
Hspb6	13.3868525	4.25094888	7.70193356	25.0323511	12.3051476
Tmem40	5.13240149	0.85933665	1.11824041	6.09784927	6.16928084
Hpdl	0.56988992	0	0.18846749	3.9987939	5.61066123
Sri	52.5713053	43.1896156	58.4505078	171.257712	153.424107
5330417C22Rik	0.92151215	1.12385347	0.83515008	5.85017518	3.12664766
Zbtb2	5.13286927	5.6329833	5.68857593	13.061151	13.8295171
Btd	22.1625721	14.7654905	9.11060749	45.9967718	31.6719898
Pld3	13.8318695	11.4760798	10.7431692	35.4128164	47.9831464
Fkbp1a	335.317633	319.272142	411.324393	1629.61855	1692.23659
6430548M08Rik	0.65377728	0.29334278	0.46124748	2.49616259	2.45320562
Upk3bl	6.75250477	0.44184229	1.9684462	19.057307	7.71586328
Cebpb	108.830679	77.4537841	159.594617	408.499876	613.01727
Myo10	8.18165925	7.65097307	11.8339055	19.6731932	31.6267847
Zfp948	14.2124822	6.26995243	21.69395	32.0673399	36.6425853
Atg13	7.26399452	9.28647867	6.18553133	18.2809807	20.8707987
Tmem159	17.562326	6.27809908	6.50497326	30.7622961	22.8880911
Tmprss13	0.46235168	1.01020195	0.72596002	5.66585277	2.52910534
Reep4	18.118697	7.08279357	6.36392625	41.6579037	24.8457518
Mtr	3.49036697	4.88750529	11.9719556	15.9688386	8.88050123
Olfm2	1.53942349	0.57080551	0.31677368	7.4591873	7.09330466
Tmem184c	2.84480525	2.82560869	2.9719678	7.18794678	7.11184368
Rorb	1.29729638	0.94238274	1.45180914	3.98070431	2.45165213
Soga2	0	0.01407107	0.36506447	8.76905731	1.90993108
4921507P07Rik	0.25145896	0.13163139	0.69854092	5.8741142	1.70161322
Tnfrsf1a	12.0437366	12.6170064	18.0734633	33.6159857	40.9087227
Snora43	2.81489307	2.5786495	7.81963796	9.90651247	8.27096136
Snord45b	2.89829731	1.13788026	0	5.32175936	5.80638197
Hmgb3	59.3835917	21.3970961	16.5183556	55.7844748	50.490278
Rnu73b	0	0	0	1.68647304	1.4720405
Ifitm1	1454.45358	373.976538	406.87976	2932.69329	1464.20364
Ddx52	23.3517207	16.1835055	18.7414831	90.3912769	74.4552873
Ugcg	15.3253608	8.30233416	7.74499501	29.105831	32.0795188
Abhd12	18.7976756	20.2527645	17.9589296	58.9262424	44.6592541
Sowaha	0.45060593	0.19813835	5.3074694	10.4581838	1.45881736
Calhm2	0.28643495	0.2998806	0.38311604	3.27253138	0.56108523
Slc19a1	4.36563612	1.71396189	2.57709965	11.4228559	8.83347482
Nop56	37.8537512	13.942786	23.4848189	57.6898373	33.7811932
Trex2	0.1925542	0	0	1.59102796	2.05737944
Abcf2	16.9808908	11.0449123	15.2546593	29.8999937	32.5771201
Tmtc1	0.64070915	0.66873983	3.00260814	9.00270668	6.66210325
Pip5k1b	3.87242812	1.52032695	2.22320005	18.6767208	13.8815284
Atp11a	20.6332835	8.03533337	16.6397418	43.372017	17.6157344
Ctr9	7.18845136	5.25144969	5.23804612	13.8257452	8.28827268
Arhgef5	8.85248384	7.41505656	10.2521571	18.8788601	24.9641135
Gpx8	239.023949	84.8706356	138.243571	299.761453	168.807983
Tspan9	5.74128361	9.01618096	5.59290928	25.9131088	31.1018185
Ubiad1	4.63288434	7.03416886	8.27497902	27.8989203	17.3663606
Tfcp2l1	32.4721854	52.1546863	56.1638959	199.908274	112.260476
Gm8615	1.04212385	1.2398211	2.45633825	6.32039459	2.65715785
Atp5g1	156.117386	92.4986531	183.450689	594.973309	692.170232
Lrrc8e	4.97747811	6.39067354	7.31844806	15.9632196	15.1058761
Ptprk	3.55776904	1.97291526	2.52215078	9.98314498	8.08776274
Ccdc85b	3.23020866	2.82724849	3.44390604	9.76744177	6.21009769
Klhl21	6.278404	7.13840926	6.46483633	26.0691062	31.0056325
Atp8a1	3.34439193	1.54582302	2.57705064	15.2577964	17.9175599
Fam108c	51.2252682	23.7719354	31.4606296	70.1691092	74.7902471
Ept1	3.73794291	2.20129147	2.60487735	9.074665	13.10103
Smad5	8.67065531	5.89477479	12.4893817	39.4431374	36.1614186
Ftsj3	8.05757002	5.61195393	8.34640589	17.4907562	12.5126011
Tgfb1i1	1.01287158	0.60917582	0.87803675	6.90500915	7.08048561
Tspan4	1.70406582	1.27277237	2.30917713	14.9579087	8.06010193
Zswim4	2.32470374	1.87247774	2.69427013	6.65066925	7.05499446
Fyttd1	27.6448918	27.7741639	33.3442657	71.2936627	57.1896271
Zmiz1	5.06094707	1.86446027	3.04934129	7.31962915	9.99314988
Svopl	0.22049599	0.15149293	0.94431291	2.45450968	1.08225463
Med13l	2.75720215	2.0933569	5.21669076	8.8113907	7.27499993
Wtip	9.33978162	12.0219523	22.48607	30.103328	20.4485626
Lrfn4	14.3872313	10.8934785	11.4191539	45.7381425	31.6695469
Slc16a11	3.19373878	2.36194393	6.60245817	18.0359456	21.9921677
Fam131c	3.03725677	1.33272277	1.11670857	4.34671098	3.72244762
Tenc1	0.18010452	0.35898708	0.30789028	5.26579439	1.39886067
Ctps	11.6440836	3.50197898	11.8984087	58.8074083	14.508442
Fam115a	1.36075757	0.93149106	1.77697914	5.2213891	2.96377121
Snord42a	7.24574328	5.68940128	4.02566547	22.6174773	9.29021115
Umps	12.9653561	3.3404686	7.76619527	20.3843718	10.0164147
Slc10a2	0	0	0	1.10257445	1.50773454
Ogfrl1	2.26167709	1.38476551	1.63303729	5.58652897	2.49204544
Bin1	4.70161672	1.77286842	2.43507667	6.68200539	7.08827974
Lphn3	2.50210027	1.18937071	0.93507371	5.346253	5.30486721
Tcn2	8.65614828	8.04575275	8.18539391	27.576049	18.4812889
Wls	174.651459	101.41065	95.691333	285.489615	285.556984
St6galnac3	0.13428405	0.05272032	0.24868976	2.20370151	2.09836816
Mcoln1	4.23452529	6.08726151	6.15311505	13.0684812	9.9711994
Ugdh	16.5566124	28.9152987	30.4150401	66.2196193	59.6830441
Zdhhc13	25.4127814	13.6148531	31.5346583	98.3735066	103.62474
Mboat7	17.6388563	16.5348225	17.9477586	42.428558	37.0519749
Gpc1	16.376359	6.87629496	6.47710534	24.3895243	19.449418
Atp2b1	44.7152805	16.8048795	24.1455237	54.5965488	76.2533495
Ncdn	4.81026513	4.08142675	5.89002392	11.3398338	7.69529953
Map9	1.0697318	0.20587251	0.50498859	4.54463528	1.61361017
Serping1	50.9152807	88.9891133	183.079303	417.443775	111.520818
Ydjc	4.54505715	2.32748234	4.43738126	20.5008684	8.35327224
Deptor	1.21429972	0.53632994	1.38795856	10.2947476	4.40926882
Cadm1	16.2769091	3.3498344	6.40050528	40.9139819	34.5444653
Cisd1	113.799467	84.36822	101.559405	256.354387	146.862353
Srm	14.1913993	4.78115349	12.0951847	31.242294	29.7096126
Gnai1	3.43111927	4.08931285	7.24317198	30.8630376	11.6036716
Gcsh	47.2626232	15.9320257	31.7055029	77.7366943	32.0892718
Nle1	2.21431883	0.6954786	1.09355938	3.7270386	2.12933532
Agpat9	5.07730916	2.84766696	9.08818117	30.3198777	18.7632719
Slc44a4	12.5973967	12.5991731	11.5877364	55.5040211	81.628569
Snrnp25	18.1056494	12.8626969	24.4588209	89.3729359	47.9235877
Tlr4	10.1225634	4.36690395	7.28513564	14.5393621	9.93244591
Lrrc20	2.01531738	0.13675402	0.9676342	3.6090909	4.32654101
Afg3l2	8.89250312	7.41780344	9.2018972	19.9109154	12.6798623
Ric8	13.6709373	13.0298219	14.1447801	27.6849868	36.1416455
Rhbdf1	3.66108499	1.13245994	2.87645372	10.2668852	7.1300553
Snord100	12.4669407	3.76504497	3.55205777	17.6087626	13.8328512
Zscan29	2.49361038	2.43266405	2.08163756	6.94607532	4.66259873
Apbb2	6.1949695	5.59508369	15.0418407	33.0116704	39.4962874
Snord87	5.28743429	4.15172526	3.26405308	14.5629226	10.5927239
Atxn10	55.9072588	43.2863726	47.4613508	188.571331	199.569538
Smg8	9.61675024	8.90238512	12.8793799	24.3122288	19.5661192
Snora52	0.4866544	0	3.15443936	2.68073699	1.55992351
Smtnl2	2.90768585	5.21022534	5.68410464	21.2647282	17.5253602
Arhgef28	3.76121104	4.36863118	3.0390269	12.836757	6.16168542
Cblc	9.14219234	10.9372064	11.2268107	24.5314855	13.8661432
Stat3	23.8348943	46.1035504	58.0298735	89.1041846	102.837793
Plekha5	1.80402492	2.38416173	2.05871262	7.09153205	2.84041011
Serpina3g	0.83935838	1.34348733	0.44840333	8.44697571	0.90545065
Cgnl1	3.99728153	4.8065922	10.3890064	24.3414941	14.3758246
Mxi1	11.2806126	15.8926575	12.573488	31.6556601	27.7050911
Rhpn2	12.742732	22.3340847	31.0089789	45.9425365	36.4388879
Herc2	2.38817734	1.83183447	3.55714999	6.09387569	7.61080238
Swt1	7.55588006	8.27817193	21.6977662	29.5758082	18.7630985
Grwd1	11.3829027	5.46505581	10.9213637	18.4771653	15.3859964
1700021K19Rik	3.83239201	4.54257643	2.52027906	10.6674233	4.71422141
Snord4a	8.38436008	6.58345005	10.3517112	23.0926344	13.4376268
Grasp	4.079778	1.01697342	1.6490437	6.30208345	2.33523803
Rabggta	4.01972331	5.38784291	5.26323754	15.0438427	13.2336267
Sf3b3	11.4829609	6.39610988	9.01771539	18.8007026	16.5804013
Galnt3	13.0989822	2.82153533	5.35990476	15.9912802	21.2916433
Bcap29	27.7994055	13.4641608	27.4258484	49.0169021	24.6713401
Csdc2	0.41903094	0.55523073	0.46076894	2.71698256	2.18263997
Trp53i13	3.77139347	1.49940297	1.90084497	6.8810816	4.47592988
Chmp4c	2.04014224	0.88678378	2.15901612	5.11736218	6.36431364
Cds2	2.52086744	3.80415995	3.18046052	13.5173523	19.3235101
Tcp11l1	0.6870252	0.26243821	0.34387803	1.92633445	1.17549476
Grb7	22.4717795	23.498153	24.3101826	52.626472	34.9036954
Snord52	18.7898089	11.282372	13.3051655	65.9582463	36.3144906
Tpbg	22.4263736	10.7581962	18.3166697	44.7173627	31.6324576
Fam174b	5.47895076	1.49139645	0.87939294	17.6613653	7.20052073
Wnt11	37.7750641	78.4598639	133.548709	215.084593	315.812614
Fry	0.37072815	0.76830778	1.40691943	3.38127627	1.74840821
Pinlyp	1.69729055	0	0.90991069	9.26751587	0.64426978
Rab11fip1	7.02468671	6.64727788	5.54192365	14.7475874	17.4060241
Rhou	32.6155287	10.0979691	11.1731048	39.545714	47.1201606
Sema4a	4.89702163	6.4284131	10.452257	21.4899401	13.075723
Xkr5	0.71864267	0.29020246	0	3.33657221	2.05673165
Samd1	21.0969734	7.81296825	12.5376834	26.2779535	32.9794645
Prss29	0	0	0	1.7742953	3.27438644
Plekha7	2.07189273	0.67686987	0.58816541	5.7342821	3.81750762
Gla	2.7745079	1.68880645	4.89930923	9.35959162	7.56629787
Rdh11	3.19015254	1.23619774	2.4936619	6.46624191	7.27088873
Wnt16	1.25256547	0.36882073	0.90613719	7.40286754	3.45037104
Sfxn1	24.7618764	12.227151	17.2129433	37.7754956	21.2005263
Arhgap17	11.6631398	12.3725429	14.354912	34.4284718	15.9513496
9430008C03Rik	2.59037607	1.17841634	1.92272484	4.73748991	2.45471571
Etv4	0.36290752	0.13151869	0	1.66589749	0.76059627
Cpeb2	1.87625279	0.88245132	2.19597788	4.16211792	3.32762419
Rab34	5.23571151	4.28122721	4.41352028	15.2823779	5.4109307
Ptp4a1	24.7643326	12.5748307	15.6483242	42.7744947	42.3349792
Tm9sf4	11.4685513	11.2137442	11.2708298	24.8240604	22.1372971
Fam196a	0.54847011	0.70672744	2.07056548	2.93086974	3.62883221
Zfp169	1.19954613	1.33332601	0.53374208	12.4573792	4.31941554
Slc39a6	20.9644999	13.7442569	23.7215604	85.3321905	60.32086
Gadd45b	15.504517	3.27612443	7.67929077	36.1772445	34.6319038
Pde4d	2.87712235	0.62471224	1.51626356	10.1920487	7.04278136
Snord35b	0.75827546	0.59540246	1.40430191	7.65776419	1.82293387
Rab5b	16.5287815	17.4443922	21.0662973	38.8135716	38.8804551
Pkib	4.04940944	1.50078096	15.4087195	13.9487992	15.7923206
Chp1	66.0915697	75.365924	124.587673	465.110279	389.573759
Arhgdig	3.60180843	0.6946362	2.10645286	7.77379092	7.03855023
0610007N19Rik	18.7324206	11.3596205	15.9207876	30.3184183	30.8329454
Mid1ip1	114.207553	125.387883	108.833739	432.260485	216.491473
Igsf8	8.58971179	3.76234601	4.65332299	21.5391772	9.92704014
Spop	45.8654041	38.1280695	40.7944344	121.276839	125.36708
Heatr2	3.31264025	2.34842631	1.70024478	6.1520774	6.12830329
Fmo2	0.50824458	0.3683785	0.14480811	14.1060123	0.0783235
Fam20c	11.3865129	1.38206382	2.21337822	12.0098685	19.8512093
Rrp15	17.3790229	6.46394849	10.5623896	35.4674185	25.1801515
S1pr2	3.26637592	3.29237467	1.7804453	7.29496942	7.98248604
Rasgef1b	2.13309265	0.56400273	0.66512163	6.07490554	1.02910174
Mir1949	0	1.4629889	0	11.9739586	5.22574377
Slc7a6	2.98959756	0.85112223	1.58652232	5.28074044	3.79672
Tgfa	6.22972868	1.70722398	2.64157524	11.580395	5.73361341
Map1lc3b	160.286676	150.323819	150.609739	570.301741	503.710419
Zfp57	0.72726791	0.444154	0.54249209	4.1359863	1.60269093
Lpar1	10.276118	8.66494846	7.92102831	19.395637	19.1254507
Lasp1	38.3899868	16.80967	7.40520753	47.8191004	66.7945344
Ckap4	52.1022384	40.0151304	33.2479918	96.7364405	97.2723351
Map6	2.90066714	2.03478669	2.37984966	7.34298358	3.08502617
Nipal2	10.5361632	4.60506833	5.98324397	18.6887667	27.3241504
Ngfrap1	193.201797	67.9088878	65.7282161	261.882473	166.521835
Myh9	15.8804752	14.1505622	13.2714246	30.4587875	38.9998867
Rora	0.75366308	3.45724567	6.97879963	9.68696161	5.49910993
Epas1	2.79026101	2.11439072	3.1930026	10.9067728	8.12372724
4922501L14Rik	0	0	0.3606868	1.19203171	1.61272331
Dusp22	9.87127815	8.1949577	5.84652283	37.1158108	11.572908
C3	4.42113149	0.21946259	1.65873417	8.15292912	11.3616405
Tnip1	7.37882351	13.048863	18.2798749	30.3767037	32.3074025
Sema3b	2.9986749	1.04316729	1.19504621	4.61743163	2.73758405
Cers6	2.2235029	3.06185336	3.85664898	9.8411924	8.44363179
Gpr160	14.8100891	11.5825481	32.5192016	74.8779503	76.2541668
Rdh12	2.45420337	0.85646901	1.8757581	14.9495659	9.20904667
Enah	3.1328377	1.40908213	2.09826547	11.1557753	5.93502149
9930104L06Rik	1.57376953	1.53161313	1.88831351	4.82295747	2.48675774
Slc22a4	7.90619599	4.98451971	25.3296296	23.6300244	30.3833067
4930523C07Rik	1.65751419	0.27549791	0.48173548	5.2094495	5.60385881
Spint1	46.2561369	37.5140206	37.6808661	153.728989	123.680532
Selenbp1	21.4792212	32.6676305	21.5737443	116.328606	46.302881
Cmtm7	26.1535008	10.4030477	13.2772608	40.1764748	19.6252646
Grk6	10.4966087	4.4052071	5.3812087	14.1782303	10.7318697
Zfand2a	7.30639664	8.19339727	5.54079842	20.3742341	20.3242063
Bri3bp	4.50733344	2.30083687	2.60101311	7.68506069	3.95595085
Cryz	3.04338559	2.3896847	3.27577086	11.5106662	4.31483431
Tyro3	4.97100141	4.69931992	7.58597693	17.774783	24.9493885
Cluh	3.35781621	2.17185702	2.91914805	10.2573733	4.89762243
Nfe2l1	23.5683916	26.8069345	30.8066387	85.8129596	56.2731109
3632451O06Rik	0.0237306	1.50930624	2.10951902	20.0873177	1.73050467
Dtnbp1	24.9895177	10.5927097	12.3535688	37.7956631	43.2420324
Cxcl17	97.3027052	71.1474602	203.560953	430.589852	260.187032
Stx3	16.4535721	10.6153393	14.5807048	29.2734182	23.976695
Kank3	0.47039563	0.75815484	2.22374459	23.8206313	4.97973305
Aim1	3.85149946	1.97471938	3.46871661	9.01760498	11.2674815
Ibtk	5.77094598	3.03602564	5.38655415	15.6297247	16.3154195
Cxx1b	13.3916862	8.56181005	9.85177061	26.1930343	35.3755707
Tbx3	1.89912044	1.71286579	1.84173007	6.03174614	6.31366918
Tirap	15.4589749	14.4841311	23.6677374	35.4815843	31.8611494
Tnfaip2	6.64903501	2.16579729	0.93030084	8.82115154	7.59708968
Pip4k2a	1.7660325	1.56372481	1.61792087	5.07108011	3.01108831
Haus4	10.6426135	3.22650356	6.54456012	27.1998239	5.99297647
F5	0.10566301	0.09679511	0.30168031	5.9821289	0.59271196
Fbxo31	3.35417186	2.36328976	1.81640245	9.71939001	3.86381055
Frzb	2.38960643	0.96447042	1.48894476	8.48838843	4.59937037
Tmem160	34.4061675	16.0210748	19.6343807	132.448143	56.7458066
Fam13c	0	0.05835283	0.20644438	2.52442431	0.84862506
Steap2	4.69097058	0.71124058	0.55546851	3.60727755	5.5377213
Prss28	0	0	0	1.05652576	1.93220778
Cry1	5.96764798	4.07463752	6.72723938	12.1089435	10.8638639
Slc15a3	0.38932352	0.17468524	0.18025368	1.94034296	0.71310789
Mrps6	107.181264	128.996233	164.665201	410.108081	322.053209
Tm4sf4	0.8862819	6.33647911	19.9987458	53.3603446	12.4849672
Met	7.27406399	2.22461406	3.76725309	18.0635409	27.2128329
Casd1	18.4882077	13.7135755	14.1487152	35.3486543	18.9711177
Ethe1	3.60091484	1.0344366	1.42321507	6.97473007	3.16711744
Awat2	1.10528287	0.57858318	0.75812909	6.99044662	5.11748919
Plekhg2	2.24600831	1.41897182	2.59373339	5.28541718	6.64079078
Ets1	3.84053033	4.22994617	5.28666938	18.5407046	10.6993489
Slc13a2	0	0	0	2.40462883	0.47701906
Mrm1	3.50147546	2.00518664	2.53534038	6.04257093	6.22363509
Chodl	0.15465223	0.24286772	0.11933791	8.6610056	0
Cited2	26.4291154	13.4187809	6.624254	48.041784	45.6224151
Il15	4.97334286	3.70585904	4.60523598	17.1455905	5.8560864
Map3k5	3.89359615	3.77886805	4.01969378	12.7110908	11.9379127
Nin	2.14976664	1.27219501	2.90716561	8.40399643	3.87990257
Prdx5	127.01114	106.651466	154.297672	432.576657	594.884087
Cxcl14	28.0549469	4.73649663	16.0960516	83.5293387	57.4631595
Camsap3	2.71715373	3.15857124	1.56022649	10.8841499	9.01367815
Fads1	0.69775376	2.23594458	1.78116488	4.951637	3.05263228
Golim4	4.33906131	1.83931705	1.74691366	9.04974929	3.38892255
Frat2	12.6976864	14.4806736	22.283934	48.1844971	68.4894652
Mgat1	7.02915856	10.0955952	11.4680416	29.9348964	20.3538933
Adpgk	7.49498981	4.45527781	4.2765568	15.1673372	7.27501359
Acat3	0.16376617	0.12859019	0.12131589	6.55530629	1.25984782
Sdc2	36.1390468	7.85147681	7.72811119	64.6405822	21.3730239
Dhrs1	20.6584958	18.9014983	17.7747772	39.4383611	60.7479391
Polg2	2.06758686	3.40931148	6.47118704	18.9821791	6.06411611
Fyn	4.35334481	2.83466506	5.52231722	12.656328	15.358156
Nfe2l3	22.8907385	5.49483449	9.4707578	97.0060086	55.5851519
Rab11a	144.818587	129.449121	133.116143	486.708311	447.783299
Chpf	6.08776824	2.184308	3.09858022	10.8635451	11.0814845
Myof	7.63897857	2.29532936	4.8859571	9.1821729	13.6205247
Cp	5.05872005	3.13030586	7.43125637	45.5450012	4.06215038
Acat2	12.9418189	8.58343199	10.9658662	48.8821285	15.3801996
St3gal1	4.63381819	6.46254606	9.57348349	25.9083837	20.8252756
Tspan14	49.0667923	50.3980769	55.2878652	160.434602	107.385236
Ypel4	0	0	0.56209425	6.13028463	2.91863349
Ntn4	1.83923471	0.16990332	0.1502737	9.03882396	0.4551664
Stk24	6.94172608	8.79271107	8.77388628	18.119102	26.5956657
Klf3	18.4869785	18.8974668	21.0851623	45.9760682	46.5438976
Uck2	13.8519969	5.01705618	7.09083185	15.6727206	25.3274642
Zbtb42	3.07496178	3.15060002	2.30914394	8.48643124	5.49920132
Cadps2	2.48199381	1.4150538	0.59955302	4.30967398	2.21378521
Cebpg	26.4480417	23.3263033	37.3970795	74.7926761	53.3577113
Chst14	3.18715493	2.57617835	2.54618036	9.40933974	3.68080658
Kctd15	2.00069031	0.40335308	1.40197305	3.99628385	2.40488208
Trim27	18.1022935	14.615969	18.0838945	61.5502484	41.2689565
Fjx1	7.22783044	0.69623081	6.81725796	29.0098378	18.1512237
Tek	0.32227522	0.550114	0.62279311	6.05901857	2.76221093
Snai1	1.11016117	1.61426896	2.36057307	7.88952503	4.77763459
Bok	19.9915724	15.9120476	16.361294	38.9655355	25.6543147
Gnpda1	29.4870248	35.8209652	38.3838451	208.086575	87.4744066
Fbxl5	51.3973142	38.3265286	61.8728332	110.888643	169.538328
Lypd1	0.09571187	0	0	2.66543598	4.09060178
Stbd1	2.36536103	1.23819716	2.55533425	17.282313	7.66091404
Pros1	4.84600629	3.35100516	3.02848228	9.70366672	9.33282679
Flot2	17.8245285	15.3803481	31.2660018	57.741089	49.4510198
Chrnb1	3.42899996	1.45345803	2.95040629	30.8681854	11.7694741
B630019K06Rik	0.39945905	0.49009008	1.0403232	4.21747392	4.02134188
Mycn	19.21685	3.39260138	7.62953548	59.4085463	44.426874
2700038G22Rik	3.71278232	1.98090833	5.20104225	14.9018973	14.5329079
Rap1gap	0.74845671	0.28631184	0.23101965	3.41811059	1.72243467
Cebpz	3.502909	3.41615265	4.75437313	9.58915249	5.67820576
Tmx4	4.19371852	2.7855627	2.11177322	12.1903134	4.09165483
Itpr3	1.90775021	1.53784707	1.32994732	4.47525036	6.93471893
Lama3	3.29778705	2.53993093	1.96184045	10.0091734	3.01660127
8430410A17Rik	29.1065679	28.3135663	38.2395302	111.782477	79.2218299
Syne2	2.70539333	2.12193344	3.30312914	9.03918641	13.0799996
Pelo	6.9691882	4.69049877	8.22034743	14.2819162	10.7706169
Pink1	25.880717	62.3068794	103.899942	191.046887	94.3770208
Slc29a2	1.2393649	0.26917103	0.39068326	3.41352235	0.73959172
Dtnb	2.93390966	0.75500183	0.61640624	5.83985124	3.45748643
Vat1	3.62817599	2.19287685	2.40921048	6.50853897	10.3673248
Dgkd	2.16456082	1.86149536	2.88456535	7.94899251	5.93792803
Il6ra	0.17556243	0.22975446	0.39738846	1.98789919	1.82893216
Sh3bgrl3	294.605568	200.617785	157.752563	717.167291	510.821997
Gbp2	16.7876936	4.5260288	15.2991686	62.6742841	35.3349681
Snhg4	20.169732	13.4795806	21.877096	64.4471531	20.3399931
Cdk5rap1	5.19707373	3.95081267	8.18415746	13.2503704	7.21524013
2810459M11Rik	0.97886055	0	0.2356664	4.70906206	0.86285172
Setd8	60.9722152	46.678857	38.418787	106.641702	101.384773
Itga1	0.39244748	0.20543475	0.42396678	4.2535376	0.81242757
Pigf	9.04978549	3.18722582	3.08086643	9.83575167	10.7181071
Khdrbs3	1.10236348	1.19017586	3.06231288	7.4955842	6.87379925
Pcolce	2.28812946	1.16449734	2.23648082	7.74144819	2.81830236
Rap1gap2	0.63075269	0.19970597	0.19782911	4.27774806	2.7555404
Spire1	1.64215941	1.41031785	1.82948795	4.87627272	7.01152322
Amot	1.54704872	0.84469508	1.19536649	3.47084895	3.18552802
D1Ertd622e	14.8619775	11.6510128	10.9610431	27.2672139	24.6781837
Lpcat4	14.0229133	5.11507686	4.88921516	14.2592093	13.9023467
Npas2	6.17794954	16.9538714	18.5922182	51.1328135	39.5056228
Ubl7	11.90448	15.0535552	12.3957441	35.5092833	22.4890879
Tlcd1	6.5059207	5.02866801	6.07144402	30.9381704	15.6405737
Zranb1	12.5088713	9.50908395	15.4156301	32.0600348	23.36475
Prdx6	192.086703	113.071366	106.799146	284.009574	181.378536
Dos	3.84063988	2.89133508	5.31622489	11.323279	8.99513272
Impdh1	3.68305223	1.93505799	1.85568466	7.85666716	6.35856696
Ccdc125	1.63933268	0.7003958	0.44646937	6.10871083	1.0625357
Acot9	22.4533789	11.8418934	20.9084951	44.8249934	62.1147838
Hpgds	1.30502472	0.97813539	1.51969482	8.15086021	5.67257814
Pqlc2	3.79100687	2.30133086	3.27441769	7.80910341	8.8840197
Sgol2	12.9556459	1.52214595	4.32595514	13.1766605	6.98566929
Rbp4	0.1508366	0.07895854	0.60524654	2.72345241	4.47230192
Zfp52	19.1883954	7.68716513	40.4793132	56.7287101	61.3413791
Chn1	3.50245191	2.57372186	1.88473596	6.28547885	8.36712116
Pus1	11.2314781	5.64417552	8.00013011	16.6886651	10.5511838
1700011H14Rik	11.8465109	2.23773367	3.98427045	116.199726	5.8213084
Frmd4b	10.9701908	18.6633631	29.7182006	54.1078163	38.3332049
Slc26a11	1.30810967	0.26629441	1.0094072	4.9817361	2.48475187
Tgfbr1	12.729285	5.15118754	16.1353447	40.4485646	62.3750532
Lipg	0.30995786	0.59493079	0.71754006	3.54128164	3.62918038
Bcl2l1	80.4213329	108.564143	57.6598901	179.558877	248.459762
Tbc1d30	0.74429496	0.2247788	0.39231683	4.32071727	1.04606636
Foxp1	5.57662765	6.06874538	6.72916556	31.2388483	25.4234809
Uap1	27.4500366	14.8926246	21.5830685	47.3084127	76.2688491
Acsl4	128.817788	143.402006	97.2678546	295.380322	291.157065
Bag1	124.525806	97.3608811	123.052866	418.955407	220.612385
Nhsl1	3.65465538	1.46164794	0.80650362	8.31764438	5.17989791
Slc44a1	3.52119251	2.40558578	2.48687388	7.99968555	6.93471184
Gstm5	47.6739684	9.61140348	32.4130145	281.473461	42.2189617
Ngef	5.51138984	7.88345231	7.31614735	31.2794898	17.2183933
Sh3gl2	3.67193405	0.21897909	1.57095641	5.39809607	1.50850052
Zdhhc18	5.45148652	3.12947046	5.89639094	10.0098424	11.4903955
Ube2f	33.060365	23.5173051	49.7522633	69.2258206	60.7305806
Sytl2	0.10400588	0.07424186	0.07004203	4.34895841	3.833879
Zyx	18.6701228	43.7906542	59.3447157	80.2274869	75.685403
S100a11	2058.95559	1236.63201	1212.85153	2965.45537	4286.97623
Maged2	17.0834598	16.6056509	13.8279972	37.7228369	25.5150362
Rsph9	3.73824972	2.66350952	13.9103409	40.9300919	18.4176957
Ttc22	0.23152079	0.06059717	0.4049486	2.03108567	3.85488791
Pcdhb22	0.17009896	0.03339068	0.61034706	1.79589858	1.19270318
Slc16a13	2.47639327	3.86870285	7.83476033	16.1042164	15.5242625
BC017158	3.35129626	2.25553544	2.9165853	13.718233	4.58363151
Dhrs13	6.91752441	3.34709043	7.82511809	22.8974494	36.3464862
Ctsz	48.4055183	29.259778	32.3382603	113.421668	142.254274
Scarb1	4.41756607	1.71783213	2.25956707	15.1026381	13.4015042
Snord73a	6.71296804	0	4.44007221	7.92394316	6.14793385
Mrpl21	14.3965323	11.5107141	15.4614773	47.1353913	30.1323129
Ocm	0.08872339	0.13933228	0.65725151	2.93239801	0.63988699
Cpxm1	0.91581885	0.35955324	0.54872764	4.22872142	1.35985897
Lgr5	5.95224507	1.17386925	20.7264946	19.1135997	6.4559178
Epb4.1l4b	27.6589027	23.1163098	45.2174798	116.631039	60.8201871
Emp2	64.5292416	51.3534621	47.2020979	115.493007	179.1944
Acss1	3.26602786	2.0800983	4.68764886	68.9485454	9.46566276
Amdhd1	0.51178112	2.00926811	3.96580427	25.0261174	3.75580097
Pkhd1l1	0.71666823	1.1214449	0.48823716	2.90912958	1.81725763
Slc35g2	12.1371914	10.4670626	10.6292808	27.9518275	38.7397409
Klc3	0.76762639	0.51663846	1.62470804	5.73864045	3.95445857
Hdac11	9.56995925	5.37915326	3.41390636	29.190725	14.6253775
Snord49a	2.10360289	4.95528499	6.81765926	26.0723291	16.0143761
B4galnt1	5.65075449	2.28856123	1.3586162	14.5806336	19.8289092
Tmem97	10.3223305	2.53526209	6.43260874	15.674837	22.6200612
Snora34	1.10528287	3.47149909	1.53521141	13.1916493	3.10001749
Osbpl3	6.77035566	1.68343928	1.27997006	10.2732593	11.1748782
Ide	9.93151436	4.56962992	6.54454479	13.9784629	19.6018028
Slc24a6	1.69763163	1.42185662	1.17374488	8.16689641	3.59145181
Ppp4r1	6.69054997	2.9126779	4.06226243	15.7683039	15.6772313
Pstpip2	1.4991193	2.89752002	7.66157818	13.1279475	14.8778912
Lacc1	3.98980159	2.2437738	0.99851149	6.38545124	5.72477098
Gjc1	0.83029336	1.26866209	1.80780917	4.01741555	2.10396428
Ugt1a7c	2.09849396	1.89646709	1.44821299	7.72454825	5.79051754
Tmem176a	195.227131	186.880816	272.083181	696.608313	261.75419
Pcbd1	17.9533417	3.79293419	3.13107314	34.6653492	9.61278792
Mapkapk2	45.9555693	31.3171952	32.180232	76.1025064	96.5144184
Cyp4v3	0.89026197	1.5728379	1.19533412	6.06871278	3.90593135
Cxx1a	9.76896682	5.86343003	6.01481782	23.7131336	24.3048318
Flot1	12.4726018	17.1085066	27.6972438	40.2194948	55.4965351
Tmem173	16.4587279	9.14209181	22.7427365	48.2183301	53.0746696
Mir1192	0	0	0.99809888	3.46354173	3.4550372
Mrto4	20.404948	10.6813921	11.1679752	30.0636486	22.6448897
Ipo4	2.76305414	2.52407113	3.3277794	7.11275024	5.52820305
Snord65	20.5931651	9.8815917	27.5440269	44.1145842	33.0046975
Shoc2	30.4465686	23.0195904	19.7938904	52.0396822	55.891767
Cep85	0.82653202	0.78144648	1.04650641	2.89592635	3.41984784
Irgq	3.42883149	2.57528092	2.12984261	6.75539301	4.95780067
Specc1	2.06106448	0.3988376	1.89946768	4.44808526	5.21321952
Epha1	4.36338528	6.10137443	7.69341201	29.1209014	9.86712389
Ampd3	2.06124516	0.56347852	2.2338626	4.07916406	5.05322448
Dsc2	5.75918454	0.8348578	1.0802567	6.86008042	12.3543399
Pcbp3	0.77954204	0.7533552	0.99947557	3.22058196	4.97346648
Emilin1	1.31778818	1.18255454	1.16795433	4.89120998	4.13352712
Ppm1m	5.73358816	4.00731742	4.84246716	30.7736519	14.4402195
Olfml2b	0.84280051	0.0496329	1.26818218	10.2716834	6.07840955
Gyg	25.7243055	9.87366995	17.1463529	35.7599703	18.5251234
Dock1	6.96612032	6.66448869	12.484584	21.0400813	17.667078
Mcph1	2.49891081	1.3191922	0.78439033	4.19956979	2.14958068
Rin2	18.7359331	23.4307552	32.9478719	143.11156	40.5087718
Naaa	34.7042407	37.2416378	75.5310501	149.970867	64.9768635
Snord47	12.0390811	14.1797386	24.1539928	46.9747605	32.9623838
Fth1	936.743694	737.163411	719.984008	3721.15923	3281.63567
Snord58b	0.59827238	0	0.55399066	4.94337739	1.4382781
Rnase1	8.40356824	0.10557652	2.92587026	10.4309227	16.3775887
Prkar1b	1.66660032	1.04689969	2.79988781	7.95640912	7.28818591
Moxd1	1.03644917	0.26573915	0.45048803	7.92308643	6.28852072
Snord72	29.3452603	22.188665	33.2117401	73.8394111	70.5475409
Thsd7a	0.8536803	0.8565135	0.86185838	4.0003907	2.44184754
Eif4a1	179.852452	118.424282	180.673151	304.444265	288.472175
Snord32a	0	1.2643114	0.74549361	2.95653298	3.87092131
F2rl1	10.4348168	7.04156305	11.6563173	21.6799903	15.4914102
Cnn2	19.3951714	14.6545185	20.1622134	50.9329999	71.3481178
Rhobtb1	0.46539484	0.05536831	0.03917711	1.87740483	3.52602088
B230208H11Rik	0.60687698	0.21660157	0.25543563	1.87409673	1.28211988
Rpp25	0.58138208	0	0.35890034	6.09373077	5.39657046
Psmd1	17.0439643	11.9676626	11.7394637	34.2191905	32.5004027
Mir744	0	0	0	2.39479171	4.70316939
Eva1b	9.47315813	6.08594796	9.61374989	22.9727818	16.6779057
Snord2	3.83598174	0	0.88801444	5.28262878	5.37944212
Klf11	2.53837566	3.84484652	4.64033262	15.6064402	6.04989422
St14	35.7633438	27.9031353	37.9416716	65.2352709	96.6573966
Gypc	1.13567562	0.1877346	0.99626918	3.10049798	1.22141582
Hpse	2.68717864	0.08307043	0.07837116	2.99154708	4.54412502
Susd3	1.40973666	0.78556587	0.75797049	3.42351674	1.38478566
Ifngr1	30.664096	15.9053867	14.4417632	37.992646	41.8756599
Tac4	0	0	0	3.74486274	0.29334568
Krt23	0.46610045	0.53234164	0.51007457	4.43479947	1.2223962
Plk3	2.9203376	3.98041763	4.23485721	8.57450771	12.5179414
Ece2	4.62966529	2.04171074	3.43546944	8.57362216	9.71965471
Nrbp2	2.53426838	1.66168811	3.48374896	8.9468881	4.14542174
Srgn	73.7629026	30.7336847	49.8272666	100.655291	127.579459
Nradd	30.701036	25.6023058	47.2600676	83.9411528	91.9892524
Snord35a	1.46543123	1.15066543	4.07089767	7.39963731	5.87162221
D230025D16Rik	5.13334791	5.83208219	4.98475818	14.0968234	10.3204344
Chchd10	34.7382945	7.61587577	14.5866676	76.9844698	75.3543432
Ppan	9.79541237	4.62710733	9.17880637	19.2758519	10.1950477
Icam1	1.4383043	1.33103756	0.57079148	3.63133048	3.00495703
Tulp3	6.06890057	3.47535745	4.70695029	15.2247428	17.8889569
Las1l	23.2181728	36.9154109	194.891484	148.614432	122.577685
Snord68	4.0757306	4.26705096	7.54812276	14.9674482	8.70957295
Pik3ca	7.03528601	4.59962923	7.51680274	13.1932424	10.2733305
Exoc6	7.49230535	6.48254504	6.65052309	17.3935659	11.9505666
Tmem38a	5.41356583	2.22310098	1.9014355	17.5096293	5.85901615
Hexb	8.55796451	7.95620211	5.99095097	30.8620139	11.7133469
Mospd1	24.1800332	11.4502041	8.23274998	31.796222	38.9620485
Smad6	13.8281267	10.2374753	5.89419212	25.9146932	37.9222604
Klf9	6.31532145	12.6010736	13.82396	34.8246603	37.0910897
Dhx32	6.64886234	4.9268448	3.85305929	11.0158802	11.8205548
Nucb2	5.13508272	3.63798306	4.54049303	15.8447587	20.2965537
Prps2	8.85502326	2.66016261	3.23668815	15.1237877	7.06725106
Noc2l	5.10454159	3.40138353	3.88111734	9.39429064	5.72298689
Nr1h3	0.73667429	0.85508683	0.53385544	3.82272744	2.56667179
Slc35d3	0.0496095	0.01947684	0.04593761	1.63964438	1.37153412
Mfsd7b	1.48389702	0.82689121	2.02416015	6.41619017	3.18377832
Oxr1	12.2247815	8.56942937	11.248533	40.7629143	19.1291271
Snx30	1.65048901	3.41727859	8.20469984	21.2698465	20.5145423
Gpr56	8.20209139	2.84911788	1.75028933	26.107816	6.58627644
Slc8a1	0.56449712	0.28356766	0.16233395	4.12995022	1.82629897
Atp6v1b2	35.496476	35.1202735	61.6847682	98.7709606	89.3962515
Prrx2	1.84833841	0.72566323	3.04866606	12.6209129	3.61034556
Dhrs7	39.1070101	14.2147771	12.1325654	81.4945416	104.955697
Tmem139	1.81933223	0.98212856	2.47436282	4.69771696	3.4625678
Bid	18.0702937	8.96283787	14.3697736	52.3029602	16.3321765
B4galt2	3.98199849	2.23334943	5.50695713	9.06830327	4.55853937
Dcun1d4	2.7145831	2.61594042	3.0278184	10.4786298	4.09110972
Eif2b3	1.54030594	1.2476509	3.1378571	5.07598193	4.54751447
Pkd2	5.98065764	2.76064771	3.18608405	9.76387641	7.78046271
Fscn1	5.74606188	2.11192624	4.91321376	9.20383016	11.109849
Lad1	14.2759548	6.02407195	12.3111093	23.3916403	23.1420805
Smpdl3b	0.37263823	0.10639919	0.06273764	1.52395836	1.3030426
Snord69	1.10528287	0	8.18779418	9.13268026	1.77143857
AI413582	20.8841204	10.7714567	9.28999724	41.2603439	13.7821814
Rexo2	60.965347	24.2924204	39.9945183	158.279024	87.5980491
Cdan1	1.28707282	0.48124635	0.62428083	3.57305624	1.56346657
Nnmt	0.54684855	1.61020791	0.6962629	4.58123152	6.73759417
Tmem222	53.7270398	46.2292647	35.8561297	103.176418	60.2265206

**FIGURE 3:**
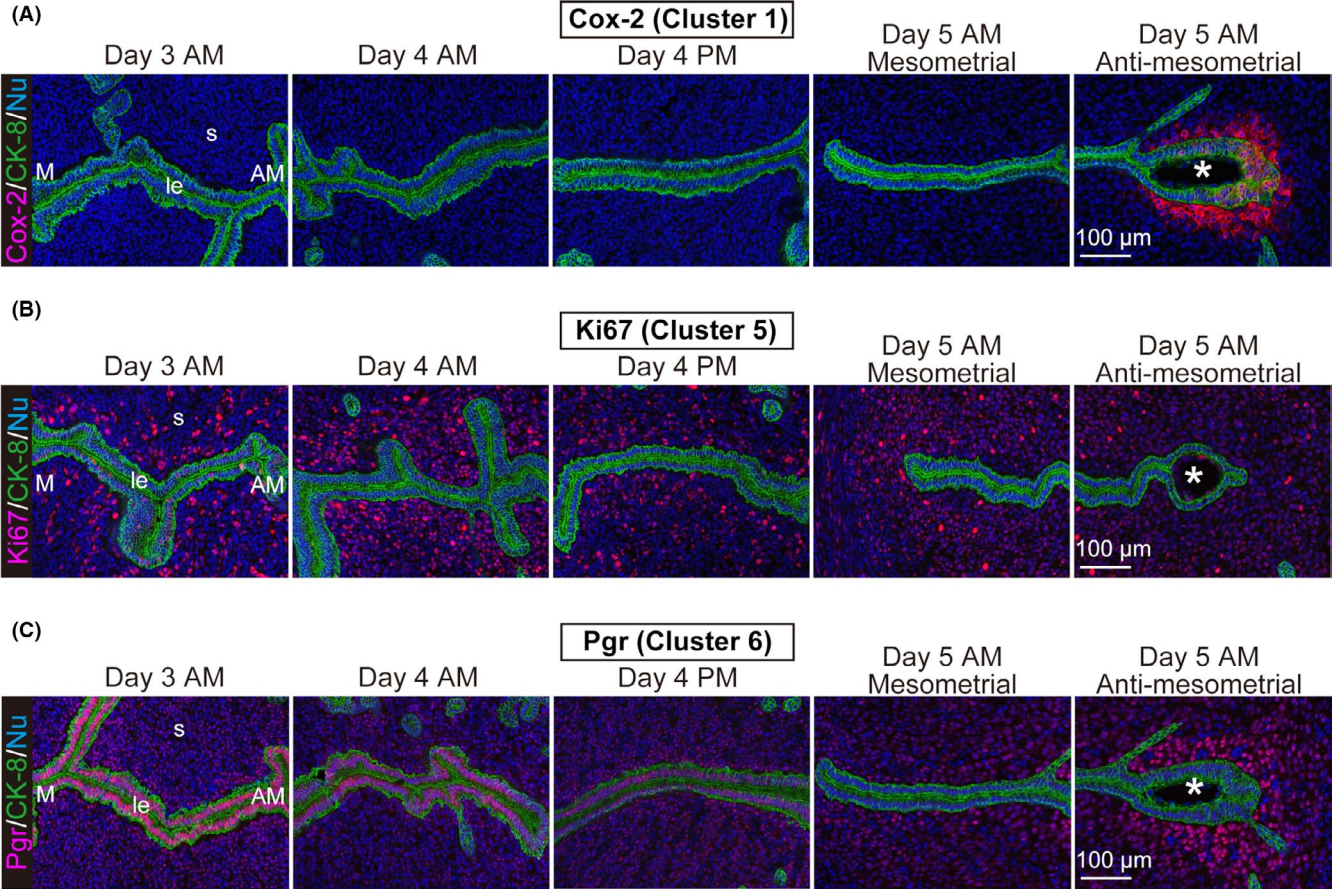
Validation of DEGs by immunostaining. The expression patterns of (A) Cox‐2 (*Ptgs2* in cluster 1), (B) Ki67 (*Mki67* in cluster 5), and (C) Pgr (cluster 6) were confirmed by immunostaining on uterine sections throughout day 3 to day 5. At least three different tissues were tested for each time point. CK8 (cytokeratin 8), an epithelial marker; Nu, nuclei; *, a blastocyst

For the validation of our RNA‐seq analyses, we performed the immunostaining of implantation‐related molecules on days 3, 4, and 5 of pregnancy and compared the variation patterns of these molecules between the results of RNA‐seq and immunostaining. Cox‐2 encoded by *Ptgs2*, which belongs to cluster 1, is highly expressed in the luminal epithelium at the antimesometrial side on day 5 a.m. (Figures 1C and 2, Table 1). These findings are in accordance with the previous studies.^19^, ^24^ Nonetheless, we confirmed by immunostaining that Cox‐2 localizes at the luminal epithelium surrounding the blastocysts on day 5 a.m., while its expression looks unclear on days 3 and 4 (Figure 3A). A previous study showed that uterine epithelial cells proliferate on the morning of day 3, while the epithelial proliferation ceases and stromal proliferation starts by the morning of day 4.^8^ We then examined cell proliferation status by the immunostaining of Ki67 encoded by *Mki67* which belongs to cluster 5 and is highly expressed in the luminal epithelium on day 3 a.m. (Figures 1C and 2, Table 5). We found that Ki67 is expressed in the luminal epithelium on day 3 a.m. and disappears by day 4 a.m. (Figure 3B). We also performed the immunostaining of P_4_ receptor (Pgr) which is classified as cluster 6 and is highly expressed on days 3 and 4 (Figures 1C and 2, Table 6). We confirmed that the epithelial expression of Pgr is high on days 3 and 4 and is decreased on day 5 (Figure 3C). Taken together, the variation patterns of implantation‐related molecules are comparable between the results of RNA‐seq and immunostaining, proving the reliability of our RNA‐seq data. These findings also assure us that the contamination of stromal cells in the RNA‐seq specimens dissected by LMD is negligible since the epithelial expression levels of *Mki67* and *Pgr* are relatively low on day 5 when these molecules are highly expressed in the stroma (Figure 3B,C).

### 3.2 Genes expressed at each time point show unique gene ontologies

**FIGURE 4:**
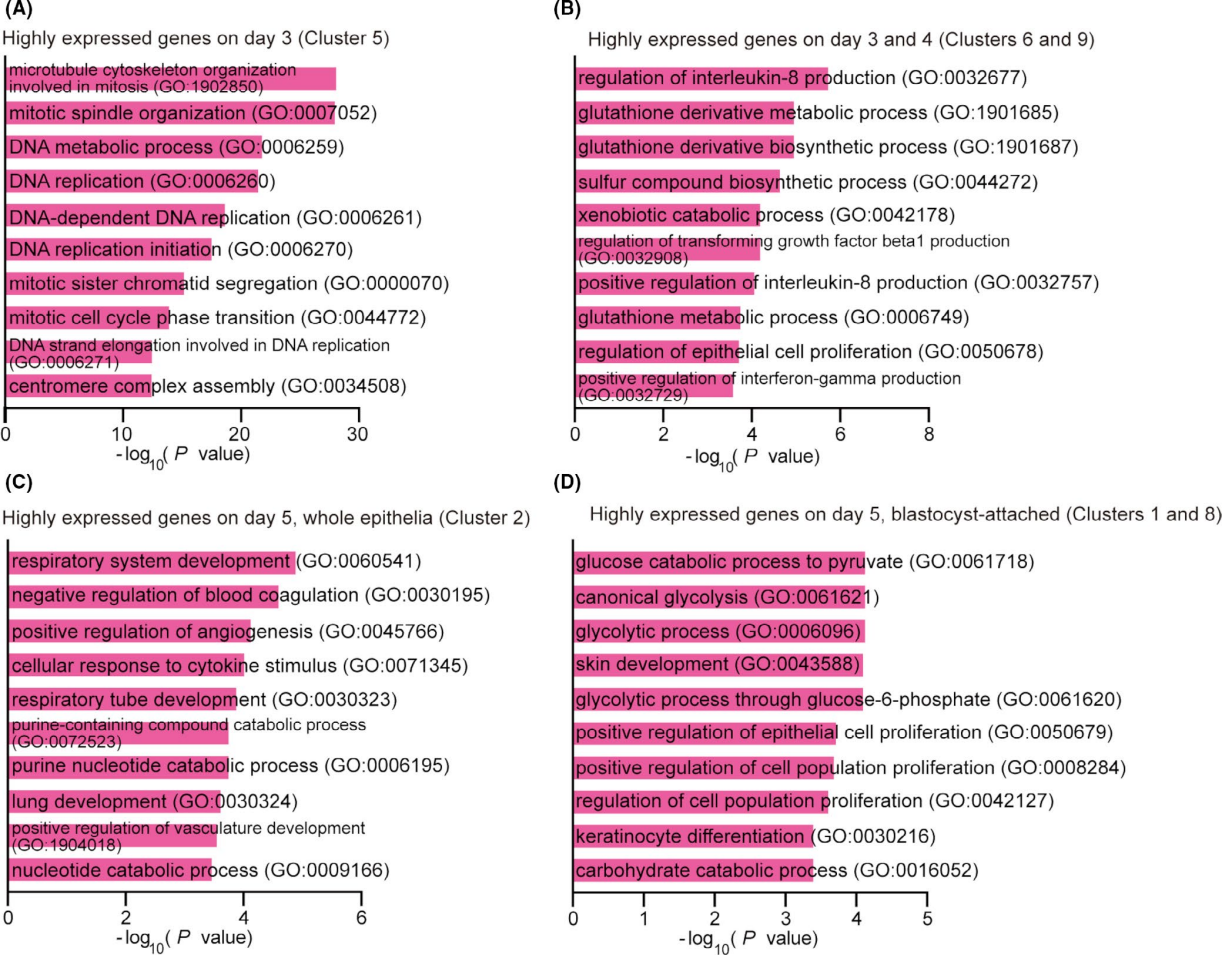
Unique GO terms among genes expressed on each day of early pregnancy. GO terms were analyzed by Enrichr for day 3 (A), days 3 and 4 (B), and day 5 with blastocyst attachment (C) and day 5 without attachment (D)

With DEGs identified, we then analyzed GO terms at each time point in early pregnancy. We first focused on cluster 5, which contains highly expressed genes on day 3. On day 3, newly formed corpus lutea start to secrete P_4_, and the luminal epithelium ceases apoptosis and proliferation, preparing to be receptive for implantation‐competent blastocysts.^4^, ^25^ We found that highly expressed genes in the day 3 epithelium are related to DNA replication and cell cycle (Figure 4A). These GO terms correspond to our previous observations that epithelial cells proliferate on day 3, but not on day 4, and this transition is critical to blastocyst implantation.^4^

We next examined 756 genes in clusters 6 and 9, which contain genes that are highly expressed on day 3 and 4 but are downregulated on day 5. These clusters show relationships with glutathione metabolism (Figure 4B). Glutathione safeguards cells from oxidative or chemical stresses,^26^ indicating that the endometrium before implantation is protective against these stimuli. This notion agrees with the fact that mouse uteri are resistant to low doses of paraquat, a chemical compound causing oxidative stress, in a P_4_‐dependent manner.^27^

We then investigated features of day 5 epithelial cells. Analysis of cluster 2 revealed that the day 5 whole luminal epithelium expresses genes related to angiogenesis and cytokine pathways (Figure 4C), which would reflect the increased permeability of blood vessels surrounding implantation sites.^18^ In contrast to the oxidative stress‐protective genes expressed on days 3 and 4 (Figure 4B), day 5 epithelia with attached blastocysts seem to take advantage of oxidative pathways for their function (Figure 4D); that is, glycolysis processes are upregulated in the day 5 epithelium surrounding blastocysts (clusters 1 and 8). This may explain why females missing glycolysis‐related genes, such as *Hif* genes or *p53*, have pregnancy defects from day 5 onward.^19^, ^28^ These data suggest that specific signaling pathways regulate uterine functions on each day of pregnancy.

### 3.3 Transcriptional regulation in the luminal epithelium changes before and after blastocyst implantation

**FIGURE 5:**
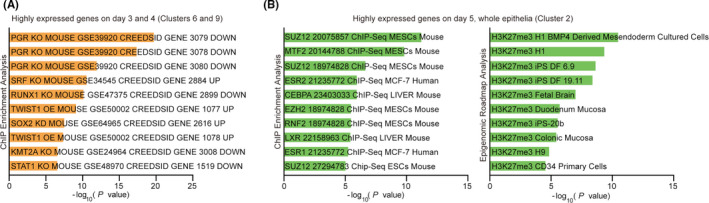
Gene expression is differently governed before and after blastocyst attachment. Gene regulatory systems were predicted in Enrichr by comparing our data and publicly available datasets. Genes expressed on days 3 and 4 (A) are progesterone receptor (Pgr)‐related, while the day 5 epithelium expresses the genes regulated by PRC2 and the trimethylation of histone H3 at lysine 27 (H3K27me3) (B)

Last, we sought to identify how these dynamic changes in gene expressions are regulated during early pregnancy. We performed comparative analyses between our data and the publicly available chromatin immunoprecipitation‐sequencing results. We first examined genes highly expressed on days 3 and 4 (clusters 6 and 9). We found that genes in these clusters are highly correlated with downregulated genes in uteri with deficiency of Pgr (Figure 5A), indicating that Pgr is a major regulator for gene expression in the uterine luminal epithelium during the pre‐implantation period. This corresponds to the fact that Pgr is highly expressed in the luminal epithelium on days 3 and 4 but is reduced on day 5 and later.^29^ On the other hand, in the analysis of upregulated genes on day 5, we observed that the expression of Polycomb repressive complex 2 (PRC2)‐targeting genes is increased (Figure 5B). PRC2 is composed of multiple enzymes, such as Suz12 and Ezh2, catalyzing trimethylation of histone H3 at lysine 27 (H3K27me3). H3K27me3 is a hallmark of gene silencing, and genes enriched with H3K27me3 modifications are transcriptionally suppressed.^30^ In the same vein, we also revealed that genes expressed on day 5 are related to H3K27me3 (Figure 5B) suggesting that PRC2‐H3K27me3 axis regulates epithelial functions upon embryo implantation. Taken together, our analyses indicate that these unique features support time point‐specific gene expression in the uterine luminal epithelium.

## 4 DISCUSSION

Appropriate communication between blastocysts and the endometrium upon blastocyst implantation is critical for successful pregnancy.^1^, ^2^ Among the cells in the uterus, epithelial cells dramatically change function during early pregnancy.^4^, ^6^, ^19^, ^25^ In mice, epithelial cells undergo massive apoptosis and proliferation after mating in response to E_2_ stimulus.^25^ Once serum P_4_ levels start to increase on day 3, epithelial cells cease cell death and growth, becoming receptive to blastocysts.^4^, ^25^ After blastocysts attach to the surrounding luminal epithelium on day 4 around midnight, trophoblast cells initiate invasion into the endometrium for placentation.^19^ Since any adverse conditions during the peri‐implantation period give rise to the termination of pregnancy, signaling pathways controlling uterine functions need to be strictly regulated.^2^

In this study, we examined gene expression dynamics in the luminal epithelium from day 3 to day 5 of pregnancy. Previously, several groups have reported comprehensive analyses of genes expressed in early pregnant uteri.^31^, ^32^, ^33^, ^34^ However, these studies only examined limited time points or compared whole endometria before or after implantation. In this paper, we focused on luminal epithelial cells that directly communicate with blastocysts. By combining LMD and RNA‐seq data, we found that luminal cells on each day of pregnancy show unique gene expression patterns. Furthermore, GO analysis revealed that DEGs govern specific signaling pathways. Analyses comparing our data with published datasets also indicate that the expression of these genes is regulated by different transcriptional regulations before and after blastocyst attachment.

It has been reported that there is a window of uterine receptivity that is strictly regulated by P_4_ and E_2_.^5^ If blastocysts try to implant into the luminal epithelium outside of this window, implantation or subsequent pregnancy processes and outcomes are compromised.^2^ This indicates that epithelial properties change before and after this receptive phase. From this perspective, our analyses clearly show dynamic transitions in the gene network during the peri‐implantation period. Intriguingly, genes expressed in the receptive (day 4) and postattachment (day 5) epithelia show contrasting features regarding the relationship with oxygen. Day 4 epithelium expresses genes that are protective against oxidative stresses, while day 5 epithelium seems to use oxygen for their functions. This may explain why females missing receptive genes are sensitive to oxidative stress^27^ and have easily induced inflammatory reactions,^35^ resulting in abnormal blastocyst implantation. After blastocyst attachment, stromal cells surrounding the blastocyst differentiate into epithelial‐like cells to form a primary decidual zone (PDZ).^36^ At this moment, the PDZ can protect the blastocyst from the external pathogens and oxygen stresses instead of the luminal epithelium.^37^ Hence, the uterine luminal epithelium may need to acquire the special gene network to combat excessive oxidative stresses prior to blastocyst implantation. It is also intriguing that unique gene expression is observed in day 5 embryo‐attached epithelium compared to the nonattached one. Once the blastocyst attaches, cellular growth‐related genes such as *Ptgs2* and *Hb*‐*egf* are induced specifically in the luminal epithelium around the blastocyst.^24^ The blastocyst attachment site‐specific epithelial gene expression may be involved in the initiation of PDZ.

We also found that transcriptional regulation is altered before and after blastocyst attachment. In contrast to the Pgr‐dependent gene expression on days 3 and 4, genes expressed in the day 5 epithelium seem to be under the control of H3K27me3. Our previous studies demonstrated that epithelial Pgr controls pre‐implantation epithelial cell differentiation to provide subsequent blastocyst attachment,^4^, ^12^, ^38^ suggesting that P_4_‐epithelial Pgr signaling induces the epithelial genes involved in blastocyst attachment. Since the current study revealed that H3K27me3‐related genes are upregulated in the postattachment luminal epithelium, H3K27me3 may participate in blastocyst invasion by regulating the epithelial genes such as programmed cell death‐related genes in the postattachment uterus. As the role of H3K27me3 in the uterus remains to be elucidated, further investigations should be planned in the future.

In conclusion, our study presents the dramatic changes of epithelial gene expression during the peri‐implantation period. This may provide hints to improve fertility treatment in humans. Further analyses in women are required in future studies.

## CONFLICT OF INTEREST

The authors declare no conflicts of interest. Human rights: This article does not contain any studies using human participants. Animal studies: The animal studies were approved by the Animal Experiment Committee of The University of Tokyo (Approval number: P16‐066; P20‐076) and performed according to the institutional and national guidelines for the care and use of laboratory animals.
